# Bifacial ladder polymers enabled by chirality-assisted synthesis that exhibit self-assembly and chirality-induced spin selectivity

**DOI:** 10.1038/s41467-026-76059-5

**Published:** 2026-08-03

**Authors:** Fumitaka Ishiwari, Kazuharu Murotani, Akinori Saeki

**Affiliations:** 1 Department of Applied Chemistry, Graduate School of Engineering, Osaka University, Osaka, Japan; 2 Innovative Catalysis Science Division, Institute for Open and Transdisciplinary Research Initiatives (ICS-OTRI), Osaka University, Osaka, Japan; 3 PRESTO, Japan Science and Technology Agency (JST), Saitama, Japan; 4 Graduate School of Urban Environmental Sciences, Tokyo Metropolitan University, Tokyo, Japan

**Keywords:** Polymer synthesis, Polymers, Self-assembly

## Abstract

The development of structural concepts in polymers has repeatedly enabled advances in molecular organization and emergent functions. Here we introduce “bifacial ladder polymers” —rigid double-stranded backbones bearing two deliberately differentiated molecular faces— realized through a chirality-assisted synthetic strategy. To implement this architecture, we designed a *C*_2_-chiral 2,8-dibromoindenofluorene monomer bearing two different substituents with *syn* stereochemistry across the sp^3^-carbon pair, such that only one ladderization geometry is accessible. Polymerization of the enantiopure monomers with a diboronate comonomer under Suzuki-Miyaura conditions affords precursor polymers, which undergo regioselective ladderization to yield well-defined bifacial ladder polymers with fully preserved facial differentiation. The homochiral bifacial ladder polymers display emergent chiroptical behaviour in the solid state: thin films form one-handed supramolecular helices with circular dichroism intensities over 100-fold stronger than those of their monomeric or non-ladder analogues. Strikingly, these films also exhibit robust chirality-induced spin selectivity, with spin-polarization values exceeding ±90% and opposite signs for the two enantiomers, far surpassing their precursor polymers. These results demonstrate that bifacial ladder polymers provide a versatile platform for emergent self-assemblies and functions in organic polymeric materials.

## Abstract (web summary)

Ladder polymers are known for their high stability and optoelectronic properties. Here, the authors report the chirality-assisted synthesis of homochiral bifacial ladder polymers, where facial orientation is retained along the chain. These display high circular dichroism intensities and chirality-induced spin selectivity.

## Introduction

Organic polymeric materials are ubiquitous in our daily lives, and the emergence of new structural concepts in polymers has repeatedly triggered paradigm shifts in their functions and applications. Examples include π-conjugated polymers^1–3^ as active components in organic electronics, helical polymers with well-defined one-handed chirality for chiral recognition and enantioselective separation^4–6^, supramolecular polymers such as polyrotaxanes based on mechanical bonds for tough elastomers^7–9^, and extended two- and three-dimensional polymeric frameworks such as covalent and metal–organic frameworks (COFs and MOFs) for gas storage and separation^10–12^. These developments illustrate how controlling not only composition but also topology and higher-dimensional architectures can transform the role of polymers in modern materials science.

**Fig. 1:**
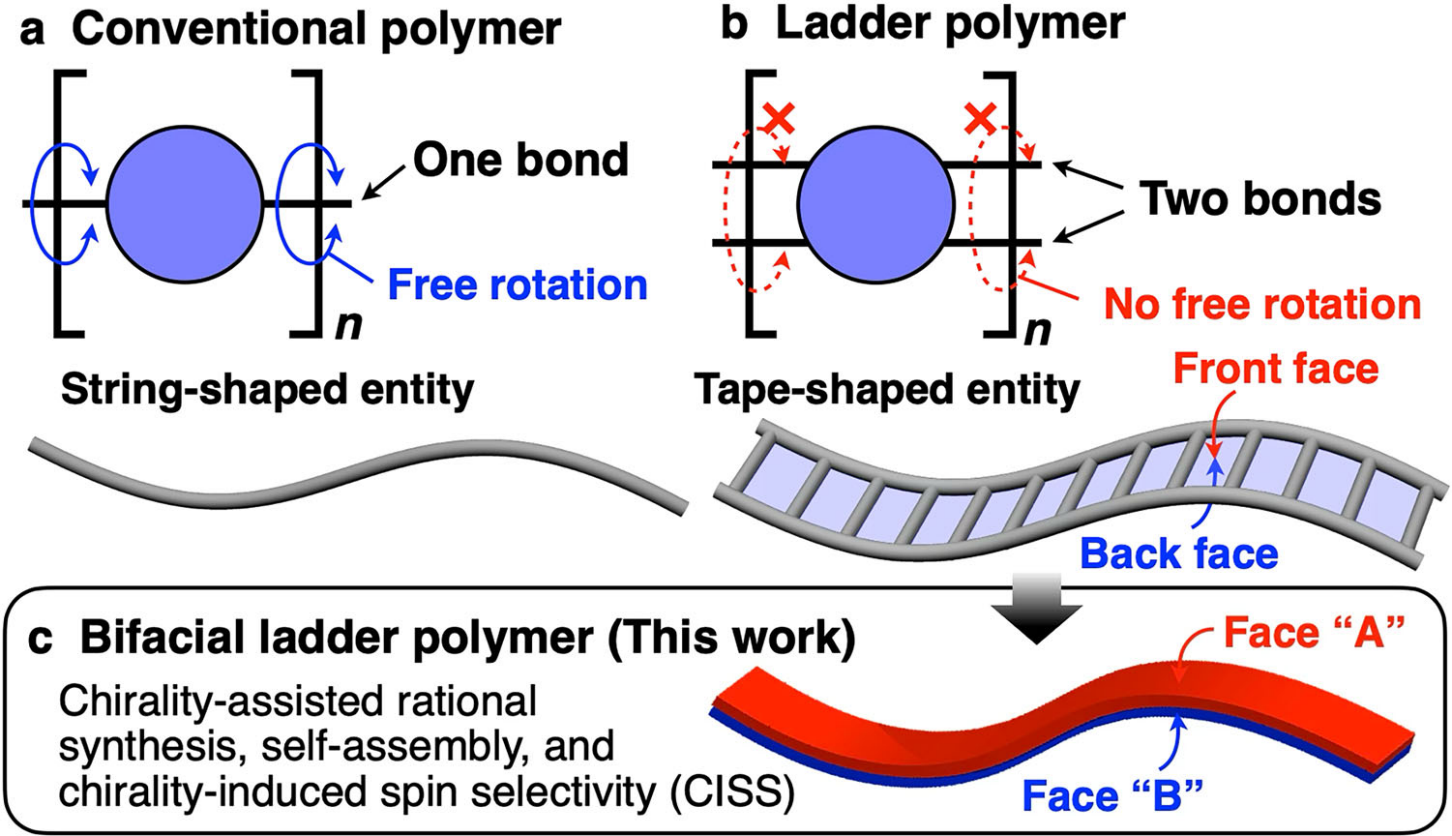
Concept of bifacial ladder polymers. **a–c** Schematic structures of (**a**) ordinary single-bond-based polymers, (**b**) ladder polymers and (**c**) bifacial ladder polymers developed in this work.

In this context, designing polymer backbones whose effective dimensionality is increased— by fixing both their conformation and the orientation of their faces —represents a promising yet largely unexplored structural concept. Ladder polymers offer a particularly attractive platform in this regard^3,13–16^. Unlike conventional single-stranded polymers (Fig. 1a), whose main chains can freely rotate around σ-bonds, ladder polymers (Fig. 1b) consist of double-stranded backbones connected by two covalent bonds, resulting in exceptional thermal and mechanical stability, intrinsic microporosity and remarkable photo- and electronic properties. Beyond these outstanding properties, ladder polymers are also highly effective building blocks for constructing special polymer architectures owing to their inherent rigidity, and several types of helical ladder polymers with one-handed helicity have been developed^17–20^. Owing to their conformationally locked double-stranded backbones, ladder polymers inherently possess two geometrically distinct faces, giving rise to the concept of “bifacial ladder polymers” (Fig. 1c). Unlike π-conjugated polymers incorporating bifacially asymmetric monomer units^21,22^, which typically retain rotational freedom along the backbone or lack control over the orientation of repeat units, the present ladder polymer architecture enforces a uniform facial orientation along the polymer chain, thereby ensuring persistent facial asymmetry even in solution and providing a unique platform for the face-selective arrangement of substituents and functional groups^23^. Such bifacial ladder polymers are expected to enable face-selective intermolecular interactions arising from the differentiation of the two faces along the polymer backbone. In addition, the rigid ladder structure, fixed by covalent bonds, may facilitate the contribution of otherwise weak interactions, thereby promoting the formation of higher-order supramolecular organization and the emergence of associated functional properties. In this work, the term “bifacial” refers to a ladder polymer architecture in which two different substituents are persistently oriented on opposite faces of the rigid ladder backbone, thereby generating permanent facial asymmetry along the polymer chain.

**Fig. 2:**
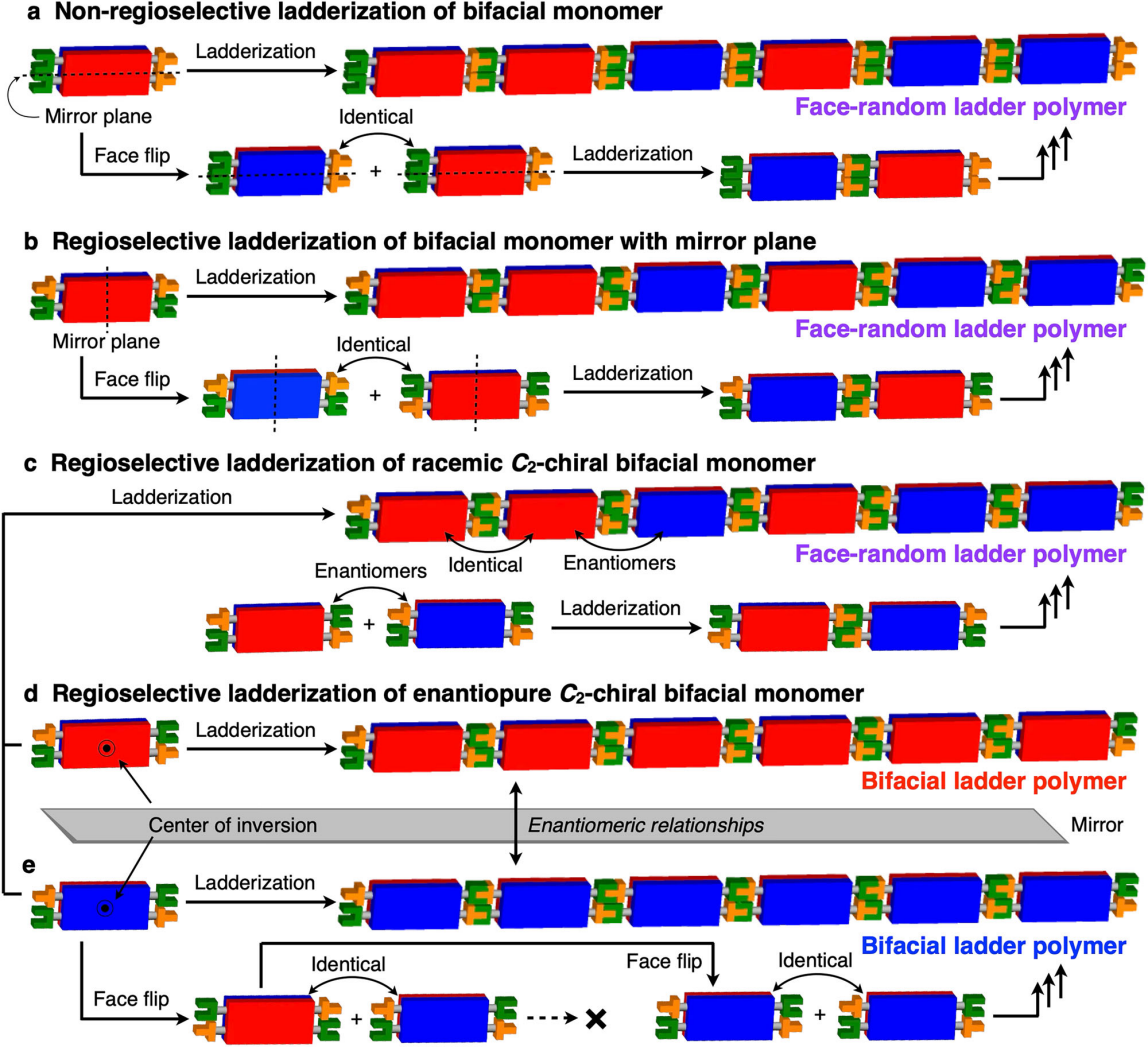
Synthetic pathways toward bifacial ladder polymers. **a–c** Unsuccessful pathways that fail to control facial orientation and give face-random ladder polymers: non-regioselective ladderization of a bifacial monomer (**a**), symmetry-allowed flipping in a mirror-symmetric bifacial monomer (**b**), and ladderization of a racemic *C*_2_-chiral bifacial monomer (**c**). **d**, **e** Successful pathways in which regioselective ladderization of an enantiopure *C*_2_-chiral bifacial monomer achieves facial control and yields bifacial ladder polymers (**d**) and regioselective ladderization of the opposite enantiomer affords the enantiomeric counterpart (**e**).

However, no synthetic approach has yet enabled their controlled differentiation. One may naturally ask, then, how such bifacial ladder polymers can be rationally constructed. If a bifacial monomer is simply subjected to ladder-forming reactions without structural control, the resulting polymer would contain randomly oriented faces within a single chain, leading to a face-random ladder polymer (Fig. 2a–c, discussed in detail later). In this study, we report a chirality-assisted synthetic strategy, which was rationally designed based on geometrical considerations of monomer symmetry and two-bond-based connectivity, to achieve the regioselective ladder polymerization of bifacial monomers and thereby realize well-defined bifacial ladder polymers (Fig. 2). These polymers were found to form thin films exhibiting chiral higher-order organization and remarkably high chirality-induced spin selectivity (CISS)^24–28^ with spin-polarization values exceeding 90%, which in turn suggests that bifacial ladder polymers offer a promising structural motif for accessing emergent molecular organization and function in organic polymeric materials.

## Results

### Geometrically guided synthetic strategy for bifacial ladder polymers

Based on geometrical considerations of monomer symmetry and connectivity, the possible ladder polymer structures that can be obtained when a bifacial monomer is subjected to ladder formation reaction, hereafter referred to as “ladderization”, are summarized in Fig. 2. In this figure, the two different faces of the bifacial monomers are depicted in red and blue, while the bond connection sites are highlighted in orange and green. The orange and green sites indicate mutually reactive positions that can be connected during the ladderization process. Each monomer possesses two reactive sites on both sides (left and right in the figure), giving a total of four reaction points available for forming ladder linkages along the polymer main chain.

Figure 2a illustrates the case of non-regioselective ladderization of a bifacial monomer. In this case, the monomer possesses two orange reactive sites on one side and two green reactive sites on the opposite side. The cyclization between the monomers occurs without distinguishing the red and blue faces of the bifacial units, leading to the random incorporation of the monomers with inverted orientations along the polymer backbone. As a result, the resulting polymer consists of randomly oriented faces along the main chain, as illustrated for the face-random ladder polymer in Fig. 2a. Such non-regioselective ladder polymerizations include, for example, Diels-Alder ladder polymerizations^15,29^, dioxane-forming ladderizations^30,31^, *o*-quinone–*o*-diamine condensations^32,33^ and catalytic arene norbornene annulation (CANAL) ladder polymerizations^34^, in which intrachain cyclization proceeds between symmetry-equivalent pairs of reactive sites without facial differentiation of the monomer units. Consequently, these ladderization methods are not straightforwardly applicable to the synthesis of bifacial ladder polymers with a uniform facial orientation along the main chain.

Figure 2b illustrates the case of regioselective ladderization of a bifacial monomer with *C*_s_ symmetry. This monomer possesses one orange and one green reactive site on each side in a mirror-symmetric arrangement, i.e., it has a mirror plane in its structure. In this case, the presence of a mirror plane allows the monomer to rotate and polymerize with the faces flipped, leading to a face-random ladder polymer.

Figure 2c–e illustrate the ladderization of a bifacial monomer in which the reactive sites on one side are inverted relative to those in panel b. In this case, the monomer adopts a *C*_2_-symmetric structure, thereby becoming chiral, *i.e*., two enantiomeric forms of the monomer can be envisioned. In this monomer symmetry, the monomer flipped horizontally or vertically with respect to the molecular plane cannot polymerize, because the reactive sites (orange and green points) no longer match between neighbouring units (Fig. 2e, below). However, the ladder polymerization between the two enantiomeric monomers can proceed because their reactive sites (orange and green points) are geometrically matched. Consequently, when a racemic mixture of the monomer is polymerized, incorporation of the opposite enantiomer into the growing chain leads to the inversion of facial orientation within the ladder polymer. In contrast, polymerization of the homochiral *C*_2_-bifacial monomer yields a ladder polymer that retains a uniform facial orientation along the entire main chain. Therefore, as shown in Fig. 2d, e, ladder polymerization of the enantiomeric *C*_2_-chiral bifacial monomers affords the corresponding enantiomeric bifacial ladder polymers if the difference between the orange and green reactive sites remains distinguishable in the resultant ladder polymer. If this distinction is lost during the ladder-forming step —for example, when ladderization proceeds via an *o*-haloamine Buchwald-Hartwig coupling that renders the two types of reactive sites chemically equivalent^35,36^— the resulting ladder polymer becomes effectively achiral, even though it is constructed from homochiral bifacial monomers.

This strategy —regioselective ladder polymerization of a homochiral *C*_2_-chiral bifacial monomer to yield a ladder polymer with a uniform facial orientation— represents a “chirality-assisted synthesis” of bifacial ladder polymers. In line with chirality-assisted synthesis^35–37^ as introduced by Schneebeli et al.^35^, the target architecture itself does not need to be chiral; rather, an enantiomerically pure chiral building block is used to enforce a single geometric mode of covalent connection. This chirality-assisted approach to the synthesis of bifacial ladder polymers is conceptually related to strategies that have been used to construct one-handed helical ladder polymers, which have long relied on concave–convex, *C*_2_-chiral, face-differentiated monomers that enforce a single bending direction of the backbone, effectively applying the principle of chirality-assisted synthesis to fix the helical sense^17–20^. A closely related precedent toward bifacial ladder polymers was reported by Scherf et al.^38^, who synthesized an optically active ladder polymer in which one face of the π-system is capped by a cyclophane unit, and a *C*_2_-chiral monomer is regioselectively linked so that the resulting backbone may be viewed as an early example of a bifacial ladder polymer. However, in that system, LiAlH_4_ reduction and BF_3_-mediated cyclization steps introduce structural irregularities along the main chain, preventing the formation of a fully regular ladder backbone. In contrast, the bifacial ladder polymers reported here are structurally well-defined, with fully regular ladder backbones and a uniform facial orientation along the main chain.

### Synthesis of bifacial monomers

**Fig. 3:**
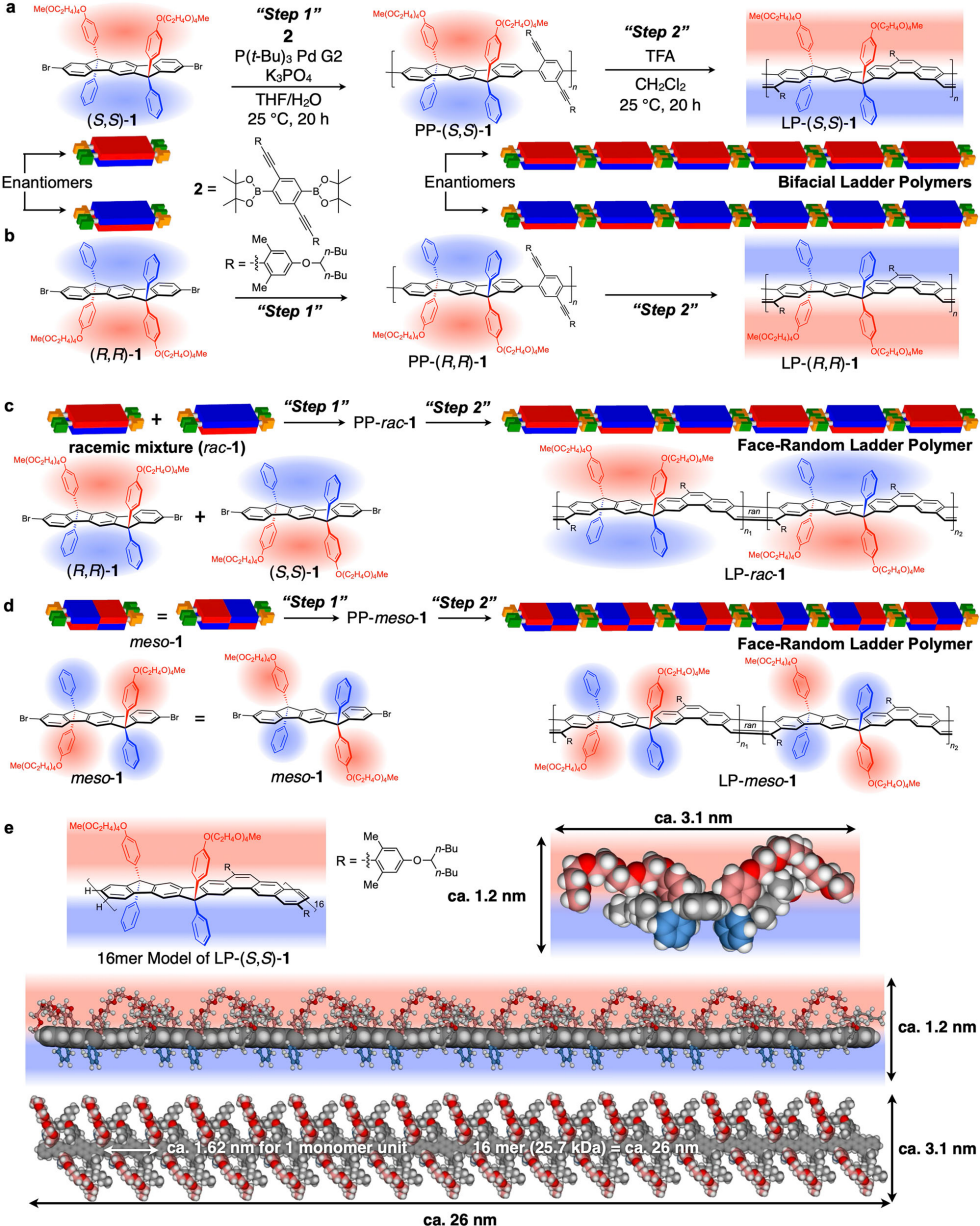
Schematic and chemical structures illustrating the formation and molecular features of bifacial ladder polymers. **a**, **b** Synthesis of bifacial ladder polymers using (**a**) (*S*,*S*)−**1** and (**b**) (*R*,*R*)-**1**. **c**, **d** Synthesis of face-random ladder polymers using (**c**) *rac*−**1** and (**d**) *meso*−**1** as control samples. **e** Energy-minimized 3D structure model of a 16-mer segment of LP-(*S*,*S*)-**1** ( ≈ 25 kDa) used for visualization. Substituents (R) are omitted for clarity in the model structure shown in the middle of panel e.

To realize this chirality-assisted synthesis of bifacial ladder polymers, we employed 2,8-dibromoindeno[1,2-*b*]fluorene as the core structure for designing a bifacial monomer, in which different substituents were introduced on its two sp^3^ carbon atoms so that the front and back faces of the molecular skeleton are distinguishable (Fig. 3). When different substituents are introduced with *syn* stereochemistry, the two sp^3^-hybridized carbon atoms become chiral centres, giving rise to a pair of enantiomers, (*S*,*S*)- and (*R*,*R*)-isomers [(*S*,*S*)−**1** and (*R*,*R*)−**1**, Fig. 3a]. The *anti*-isomer is achiral, corresponding to a meso form, and can be used as a reference compound (*meso*-**1**, Fig. 3d). Hydrophobic phenyl groups (blue) and hydrophilic tetraethylene glycol (TEG)-appended phenyl groups (red) were employed as the substituents to facilitate both the separation of *syn* and *anti* isomers and the chiral resolution of the (*S,S*)- and (*R,R*)-enantiomers of the *syn* isomer.

**Fig. 4:**
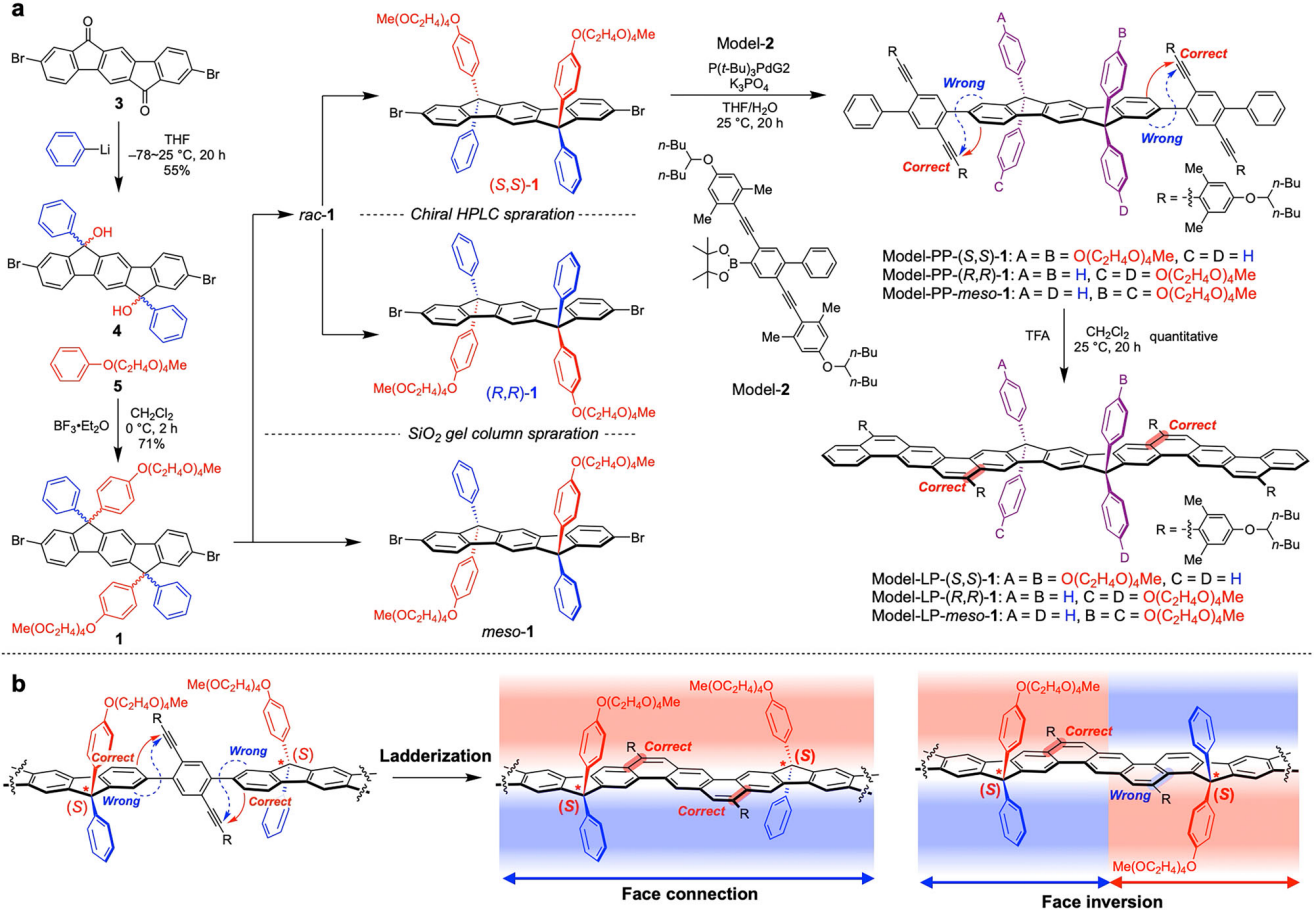
Synthesis of bifacial monomers and regioselectivity in model reactions. **a** Synthesis of bifacial monomers **1 **s and Model-PP−**1**s and model reaction using Model-PP−**1**s. **b** Schematic illustration of face connection and face inversion in regioselective and non-regioselective ladderization pathways.

The monomers **1** were synthesized as shown in Fig. 4a. While *meso*-**1** and *rac*−**1** have been prepared previously^39^, the chiral resolution of *rac*−**1** and the chiroptical properties of these isomers are reported here. Nucleophilic addition of phenyllithium to 2,8-dibromoindeno[1,2-*b*]fluorene-6,12-dione (**3**) afforded the corresponding diol (**4**). Subsequent Friedel–Crafts-type reaction with tetraethylene glycol methyl phenyl ether (**5**) gave a mixture of the desired (*S*,*S*)−**1**, (*R*,*R*)-**1** and *meso*-**1** isomers. The *meso*−**1** and *rac*−**1** [a mixture of (*S,S*)−**1** and (*R,R*)-**1**] were separated by silica gel column chromatography. Importantly, the *rac*-**1** was resolved by chiral HPLC to afford the enantiomerically pure (*S*,*S*)−**1** and (*R*,*R*)-**1**. All isolated compounds were unambiguously characterized by ^1^H NMR, ^13^C NMR, 2D NMR, FT-IR and high-resolution mass spectrometry (Analytical Data, Suppl. Figs. 12–99). The mirror-imaged circular dichroism (CD) spectra and chiral HPLC traces (Suppl. Fig. 1) clearly demonstrate the successful chiral resolution with high enantiomeric purity. The absolute configurations of (*S*,*S*)-**1** and (*R*,*R*)-**1** were determined based on the agreement between the experimentally observed and theoretically simulated CD spectra, which were calculated by TD-DFT at the B3LYP/6-311 G(d,p) level of theory (Suppl. Fig. 1a).

### Model reaction for ladderization

As a regioselective ladderization reaction for monomer **1**, we employed an intramolecular Friedel–Crafts-type cyclization toward the inner alkyne moiety under strong acid conditions, which was originally developed by Swager^40^ and subsequently refined by Ikai^18^ to enhance regioselectivity. In the original version of the reaction, an inner alkyne with relatively less bulky substituents was employed; however, Scherf et al. reported that, in fluorene-based systems, the Friedel–Crafts-type cyclization can take place not only at the “correct” site but also at the “wrong” one, thereby generating undesired regioisomers (Fig. 4b)^41^. Subsequently, Ikai and co-workers demonstrated that employing a more sterically bulky inner phenylacetylene bearing methyl groups at both ortho positions relative to the ethynyl group improved the regioselectivity, allowing the reaction to proceed exclusively at the “correct” site even for fluorene-based substrates^18^. Because the occurrence of the Friedel–Crafts-type cyclization at the “wrong” site during the ladder polymerization leads to inversion of facial orientation within the ladder backbone (Fig. 4b), special care must be taken to suppress this undesired side reaction.

**Fig. 5:**
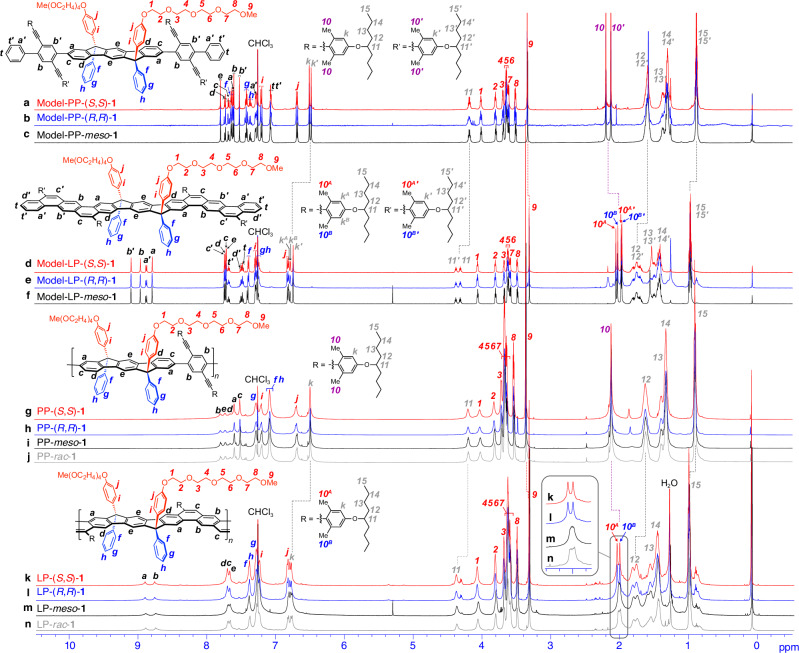
Structural characterization of bifacial ladder polymers. **a–n** ^1^H NMR spectra (600 MHz, CDCl_3_, 25 °C) of Model-PP-(*S*,*S*)-**1** (**a**), Model-PP-(*R*,*R*)-**1** (**b**), Model-PP-*meso*−**1** (**c**), Model-LP-(*S*,*S*)−**1** (**d**), Model-LP-(*R*,*R*)-**1** (**e**), Model-LP-*meso*-**1** (**f**), PP-(*S*,*S*)−**1** (**g**), PP-(*R*,*R*)−**1** (**h**), PP-*meso*-**1** (**i**), PP-*rac*−**1** (**j**), LP-(*S*,*S*)−**1** (**k**), LP-(*R*,*R*)-**1** (**l**), LP-*meso*−**1** (**m**) and LP-*rac*−**1** (**n**).

To examine whether the Friedel–Crafts-type cyclization proceeds selectively at the “correct” site even in the case of the indeno[1,2-*b*]fluorene framework of monomer **1**, a model reaction was carried out using a small molecular analogue. As shown in Fig. 4a, the bifunctional dibromo monomers (*S*,*S*)-**1**, (*R*,*R*)-**1** and *meso*-**1** were subjected to Suzuki-Miyaura cross-coupling with a monofunctional boronate bearing a sterically bulky inner phenylacetylene (Model-**2**) to afford the corresponding model precursor compounds (Model-PP-**1**) for ladderization. Treatment of Model-PP-**1** (Fig. 5a–c) with trifluoroacetic acid (TFA) quantitatively yielded a single compound whose ^1^H NMR spectrum (Fig. 5d–f) exhibited a characteristic downfield-shifted singlet proton signal (*a*), indicative of the “correct” cyclization product (Model-LP-**1**). Additional spectroscopic characterization, including 2D NMR, FT-IR (Supplementary Fig. 2) and high-resolution mass spectrometry, unambiguously corroborates that the cyclization proceeds solely at the “correct” site, with no detectable formation of regioisomeric products (Supplementary Figs. 74–99). These results clearly demonstrate that even with the indeno[1,2-*b*]fluorene skeleton, the Friedel–Crafts-type ladderization developed by Ikai et al.^18^ proceeds in a geometrically selective manner.

**Fig. 6:**
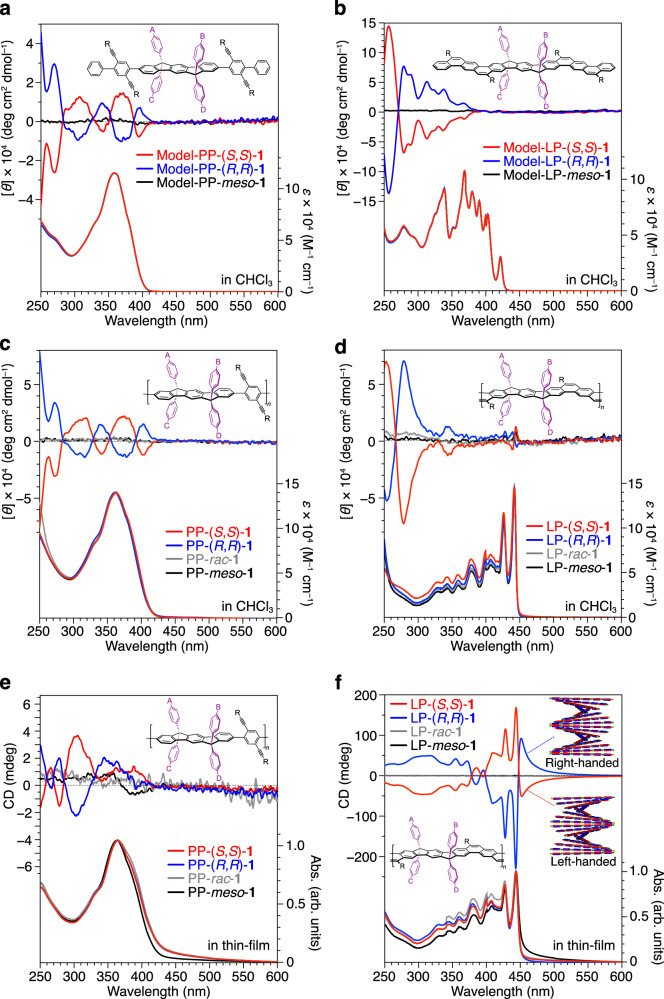
UV-vis/CD spectra. **a–d** UV-vis/CD spectra (25 °C, 15 µM for monomer units, CHCl_3_) of Model-PP-**1**s (**a**) and Model-LP-**1**s (**b**), PP−**1**s (**c**) and LP−**1**s (**d**). **e**, **f** UV-vis/CD spectra in thin-film states of PP-**1**s (**e**) and LP-**1**s (**f**).

Upon ladderization, the UV-vis absorption spectra of the model compounds exhibited a red shift of the absorption edge from 400 nm to 425 nm, indicative of an extended effective π-conjugation length induced by their ladder backbones (Fig. 6a, b). In addition, the spectra evolved from broad, featureless bands to well-resolved vibronic progressions, consistent with the increased backbone rigidity imparted by the ladder architecture (Fig. 6a, b). The chiroptical response changed in parallel. Upon ladderization, the CD spectra displayed markedly altered line shapes with a modest increase in intensity. The enantiomeric pair, Model-LP-(*S*,*S*)−**1** and Model-LP-(*R*,*R*)−**1** (Fig. 6b), showed mirror-image spectra of identical amplitude. Taken together, these observations demonstrate that the intramolecular Friedel–Crafts cyclization proceeds while preserving the absolute configuration of the *C*_2_-chiral bifacial monomer —and thus its facial differentiation— throughout the reaction.

### Synthesis and characterization of bifacial ladder polymers

Having confirmed that the cyclization proceeds exclusively at the “correct” site on the indeno[1,2-*b*]fluorene scaffold (Fig. 4b), we proceeded to the polymer system to achieve bifacial ladder polymers. The dibromo monomer **1** and the bulky phenylethynyl diboronate monomer **2**, which was synthesized from the dibromo precursor (compound **6** in Supplementary Information) reported by Ikai and co-workers^18^, were polymerized by Suzuki–Miyaura coupling to afford the corresponding precursor polymers. The diboronate monomer **2** contains a branched alkyl chain introduced to enhance the solubility of the resulting polymers. To avoid introducing additional chirality at the branching point, an achiral 5-nonyl substituent was employed, consisting of two identical *n*-butyl chains extending from the branching carbon. As the dibromo monomer **1**, we used the bifacial enantiomeric monomers (*S*,*S*)-**1** and (*R*,*R*)−**1**, as well as the racemic mixture *rac*−**1** and the non-bifacial *meso*-**1** as control compounds (Fig. 3a–d). For intuitive comparison, the stereochemical regularity of the polymers derived from these monomers can be discussed in relation to the concept of tacticity commonly used for polylactide (PLA).^42^ In this context, polymers derived from enantiopure (*S*,*S*)−**1** and (*R*,*R*)−**1** are conceptually analogous to optically active isotactic PLA derived from L- and D-lactide, respectively. Polymers derived from *meso*-**1** are analogous to optically inactive atactic PLA obtained from *meso*-lactide, whereas polymers derived from *rac*-**1** are analogous to optically inactive atactic PLA obtained from a racemic mixture of L- and D-lactide. The polymerization proceeded smoothly to give well-defined precursor polymers with number-average molecular weights (*M*_n_) of 25.6–30.7 kg/mol and dispersities (*Ð*) of 1.9–2.7, as determined by size-exclusion chromatography (SEC) (Suppl. Fig. 3). The ^1^H NMR spectra confirmed the formation of structurally well-defined precursor polymers (Fig. 5g–j, Supplementary Figs. 100–119).

Thus obtained precursor polymers PP-(*S*,*S*)−**1**, PP-(*R*,*R*)−**1**, PP-*rac*-**1** and PP-*meso*-**1** were subsequently subjected to intramolecular Friedel–Crafts-type cyclization with TFA in CH_2_Cl_2_, similar to those employed in the model reaction, to afford the corresponding ladder polymers LP-(*S*,*S*)−**1**, LP-(*R*,*R*)−**1**, LP-*rac*-**1** and LP-*meso*−**1** (Fig. 3a–d). The resulting ladder polymers were readily soluble in common organic solvents such as CHCl_3_ and THF, facilitating comprehensive characterization by NMR, IR and SEC (Fig. 5k–n, and Supplementary Figs. 120–139). Despite the line broadening inherent to polymer samples, all the LPs exhibited essentially identical ^1^H NMR spectra consistent with assignment to a single, well-defined chemical structure. Two pronounced singlets are observed in the downfield aromatic region at ca. 8.8 and 8.9 ppm, assignable to the bay-region protons (*a* and *b*) of the phenanthrene motif generated upon regioselective ladder formation. In addition to the bay-region singlets, the LP−**1**s exhibit systematic chemical-shift changes that closely parallel those observed in the model compounds upon ladderization. In particular, the aromatic protons (*k*) on the phenylethynyl side group and the aliphatic protons on the adjacent alkyl substituents (*11*–*15*) undergo characteristic downfield shifts (grey dotted lines in Fig. 5), while the methyl protons (*10*) shift upfield (purple dotted lines in Fig. 5). These shifts reflect the same local electronic reorganization induced by the intramolecular Friedel–Crafts cyclization in the model system, demonstrating a one-to-one correspondence between the ladderization-driven NMR signatures of the small-molecule analogues and those of the polymer backbones. In addition to the global chemical-shift trends, a particularly diagnostic signature of bifaciality appears in the methyl protons (*10*). Because the substituents (R) project nearly perpendicular to the rigid ladder backbone, these methyl protons are subjected to distinct magnetic environments on the two faces of the chain. As a result, LP-(*S*,*S*)-**1** and LP-(*R*,*R*)−**1** display two cleanly resolved resonances (*10 *^A^ and *10*^B^), arising from magnetically nonequivalent methyl protons on the hydrophilic and hydrophobic faces of the bifacial backbone (Fig. 5k,l). In stark contrast, LP-*rac*−**1** and LP-*meso*-**1**, which lack a uniform facial orientation, show a broadened multiplet for the same methyl group (Fig. 5m, n). This face-sensitive splitting observed exclusively for the homochiral LPs provides direct spectroscopic evidence that the ladderization generates well-defined bifacial polymers with differentiated front and back faces.

Together with these NMR signatures, the disappearance of the internal alkyne stretching band in the IR spectra (∼2200 cm^–1^) further corroborates complete intramolecular cyclization of the precursor backbones (Suppl. Fig. 2). SEC analysis shows no discernible change in elution profiles upon ladderization (Supplementary Fig. 3). In the ladder polymers, a minor early-eluting shoulder attributable to aggregation becomes more apparent —presumably reflecting reduced solubility and increased intermolecular association after backbone rigidification— although the number-average molecular weights and dispersities remain essentially unchanged (precursors: *M*_n_ = 25.6–30.7 kg/mol, *Ð* = 1.9–2.7; ladder polymers: *M*_n_ = 25.1–30.1 kg/mol, *Ð* = 2.2–3.1). These observations indicate neither chain scission nor intermolecular crosslinking during the Friedel–Crafts cyclization. Considered alongside the model study, where the Friedel–Crafts ladderization proceeded without detectable side products, these data strongly indicate that the same regioselective transformation occurs along the polymer backbone. Taken together, these spectroscopic and chromatographic analyses indicate that ladderization proceeds to near-completion along the polymer backbone, yielding structurally uniform ladder polymers with no detectable defects within the sensitivity of the employed characterization methods.

In addition to spectroscopic and chromatographic characterization, bulk thermal properties were examined by thermogravimetric analysis (TGA) and differential scanning calorimetry (DSC). TGA revealed two distinct classes of decomposition behaviour: the homochiral ladder polymers LP-(*S*,*S*)-**1** and LP-(*R*,*R*)−**1** exhibit essentially identical TGA profiles, whereas LP-*rac*−**1** and LP-*meso*-**1** show a second, likewise mutually similar profile. These two profile types differ markedly from each other (Suppl. Fig. 4). This difference may originate from the sequence regularity of the backbones: the homochiral LPs are perfectly uniform polymers, whereas LP-*meso*-**1** and LP-*rac*-**1** are statistical copolymers of two configurationally distinct repeat units. Such sequence heterogeneity could influence thermal decomposition pathways and contribute to the observed variations in TGA behaviour. In contrast, DSC measurements showed no discernible thermal transitions for any polymer (Suppl. Fig. 5), indicating the absence of cooperative phase transitions in the bulk. In addition, time-resolved microwave photoconductivity (TRMC) measurements^43^ likewise reveal a clear dichotomy between the homochiral and the meso/racemic polymers (Supplementary Fig. 6). The homochiral samples exhibit markedly larger TRMC signals, with peak *φ*Σ*µ* values of approximately 8 × 10^−^^9^ m^2^ V^−1^ s^–1^, whereas the meso and racemic polymers show values around 5 × 10^−9^ m^2^ V^−1^ s^−1^. This enhancement in the homochiral polymers may reflect the uniformity of their repeat-unit sequences: identical configurational units along the backbone minimize intrachain trap sites and support more efficient short-range charge migration. By contrast, the statistical mixture of two configurationally distinct repeat units in the meso and racemic polymers likely introduces intrachain trapping sites, suppressing their effective TRMC response.

To visualize the overall molecular scale, we constructed a 16-mer model of LP-(*S*,*S*)-**1** (*M*_n_ ≈ 25.1 kg/mol) by molecular-mechanics energy minimization, in which a single repeat unit was optimized and then concatenated 16 times with hydrogen end-capping (Fig. 3e), retaining all side chains to preserve realistic steric envelopes. The resulting repeat spacing (~1.62 nm) gives a contour-length estimate of ~26 nm for a 16-mer. Owing to the flexible TEG side chains, the overall molecular thickness is estimated to be approximately 1–2 nm, while the lateral width is roughly 2–4 nm. The ladder backbone features two chemically distinct faces, one TEG-decorated, hydrophilic face and one phenyl-appended, hydrophobic face, yielding a Janus-like, amphiphilic bifacial architecture.

### CD spectra of bifacial ladder polymers in solution and thin-film state

Figure 6c, d display the UV–vis absorption and CD spectra of the precursor polymers (PP-**1**s) and the corresponding ladder polymers (LP-**1**s) in CHCl_3_ solution. The precursor PP-**1**s exhibit UV-vis and CD features closely resembling those of the small-molecule models (Model-PP-**1**s, Fig. 6a), indicating that the precursor backbones can be regarded as a straightforward concatenation of the model motifs. As observed for the small-molecule models, upon ladderization, the absorption edge (*λ*_edge_) undergoes a pronounced red shift from 400 nm to 450 nm, reflecting an extension of the effective π-conjugation length induced by their ladder backbones (Fig. 6d). In parallel, the absorption profiles sharpen and reveal clearly resolved vibronic structure, consistent with increased backbone rigidity. Relative to the ladderized model (*λ*_edge_ ≈ 425 nm), the LP-**1**s exhibit an additional ~25 nm bathochromic shift, which we attribute to intrachain electronic delocalization across multiple repeat units, *i.e*., an increase in exciton coherence length beyond a single repeating ladder motif. In the CD spectra (Fig. 6d), the LP-**1**s show pronounced changes in line shape relative to the precursors (Fig. 6c), while closely matching those of the corresponding model compounds (Fig. 6b), supporting both the assigned chemical structures and the absolute configurations. Moreover, LP-(*S*,*S*)-**1** and LP-(*R*,*R*)-**1** exhibit mirror-image spectra with comparable amplitude (Fig. 6d), indicating that the stereochemistry is retained throughout the ladderization. By contrast, the LP-*meso*-**1** and LP-*rac*-**1** controls behave as expected: their UV-vis spectra are essentially identical to those of LP-(*S*,*S*)-**1** and LP-(*R*,*R*)-**1**, indicating comparable electronic structures, whereas almost no CD signal was observed (Fig. 6d), consistent with the lack of chirality in the meso backbone and enantiomeric cancellation in the racemate.

To investigate the behaviour of the bifacial ladder polymers in the solid state, thin films were prepared by spin-coating CHCl_3_ solutions of the polymers (1.0 mg/mL) onto quartz substrates at 1000 rpm. For comparison, thin films of the ladderized small-molecule models (Model-LP-**1**s, Suppl. Fig. 7a) and of the precursor polymers (PP-**1**s, Fig. 6e) were fabricated under similar conditions and subjected to the same characterization. The UV-vis spectra of Model-LP-**1**s, PP-**1**s, and LP-**1**s in thin films are essentially unchanged from their solution counterparts (Fig. 6e, f, Supplementary Fig. 7a). In the CD spectra, Model-LP-**1**s and P−**1**s exhibit signals of only a few millidegrees (mdeg), broadly similar to those in solution (Fig. 6e, Suppl. Fig. 7a, c); however, the film spectra do not perfectly reproduce the solution profiles, and the (*S*,*S*)/(*R*,*R*) pairs are not exact mirror images, both effects attributable to film-induced orientational anisotropy. By contrast, the bifacial ladder polymers LP-(*S*,*S*)−**1**s and LP-(*R*,*R*)-**1**s display dramatically stronger CD responses, reaching ~100–200 mdeg (Fig. 6f, Suppl. Figs. 7, 8), together with pronounced spectral-shape change relative to solution. The two enantiomers give mirror-image spectra of comparable amplitude, whereas the racemic and meso controls show negligible net CD. These observations indicate that, upon film formation, homochiral LP-(*S*,*S*)-**1** and LP-(*R*,*R*)−**1** develop chiral higher-order organization that amplifies the molecular chirality; such a behaviour is not observed for the model or precursor analogues. More specifically, the thin-film CD spectra of LP-(*S*,*S*)−**1** and LP-(*R*,*R*)−**1** are characterized by a strong bisignate couplet on the lowest-energy absorption band, indicative of exciton splitting associated with one-handed packing of identical chromophores. TD-DFT calculations on a model monomer model show that the transition-dipole moment of the first excited state is oriented predominantly along the ladder backbone (Suppl. Fig. 9); applying the Frenkel–Kasha exciton-coupling helix rule^44,45^ to a helical stack of such approximately collinear dipoles, the observed couplet signs are consistent with a left-handed supramolecular helix for LP-(*S*,*S*)-**1** and a right-handed helix for LP-(*R*,*R*)−**1** (Fig. 6f, insets). To evaluate the thermal stability of the chiral supramolecular structure in the thin-film state, UV–vis/CD spectra of an LP-(*S*,*S*)−**1** thin film were measured after thermal annealing at 50–300 °C for 60 s (Suppl. Fig. 7d). Remarkably, the CD spectral shape and intensity remain essentially unchanged even after annealing at 300 °C. These results indicate that the supramolecular chirality responsible for the strong chiroptical response is highly stable against thermal treatment, consistent with the intrinsic structural stability of the ladder polymer backbone.

We note an intriguing observation. Although the *M*_n_ ≈ 28 kg/mol, *Ð* = 2.3 sample exhibited the strong CD with a markedly reshaped spectral profile characteristic of homochiral ladder polymers, a lower-molecular-weight batch of LP-(*R*,*R*)-**1** with *M*_n_ ≈ 14.3 kg/mol, *Ð* = 2.8 (Suppl. Fig. 129) gave a thin-film CD spectrum that closely resembled that in solution —showing neither the characteristic spectral-shape change nor an intensity increase (Suppl. Fig. 7b, c). This suggests a critical chain-length requirement for the emergence of chiral supramolecular ordering in thin films, highlighting the importance of employing sufficiently high-molecular-weight ladder polymer systems for chiral self-assembly. This behavior may arise from the rigid ladder backbone and polymeric nature of the system, which together can facilitate the amplification of otherwise weak chiral interactions, as described in the Introduction.

Whereas DSC indicates no cooperative phase transitions in the bulk, thin films of the ladder polymers exhibit clear signatures of supramolecular ordering. To assess solid-state order, 2D grazing-incidence wide-angle X-ray scattering (GI-WAXS) patterns were collected for the thin films. Qualitatively, LP-(*S*,*S*)−**1** and LP-(*R*,*R*)−**1** (Suppl. Fig. 10a–d) exhibit closely similar diffraction images, while LP-*meso*-**1** and LP-*rac*-**1** (Suppl. Fig. 10e–h) share a different, but internally consistent, pattern. A main distinction is that the films of LP-*meso*−**1** and LP-*rac*−**1** display a clear out-of-plane diffraction at *q* ≈ 4.8 nm^–1^ (*d* ≈ 1.3 nm), which is absent in the films of LP-(*S*,*S*)-**1** and LP-(*R*,*R*)-**1**. The enantiomeric pair and the meso/racemic pair are thus clearly distinguishable in reciprocal space. While these qualitative differences are clear, a definitive structural assignment is not possible at this stage because only a few characteristic diffractions are present in the patterns under our measurement conditions; full 2D WAXS data are provided in the Supplementary Information (Suppl. Fig. 10).

### Chirality-induced spin selectivity

**Fig. 7:**
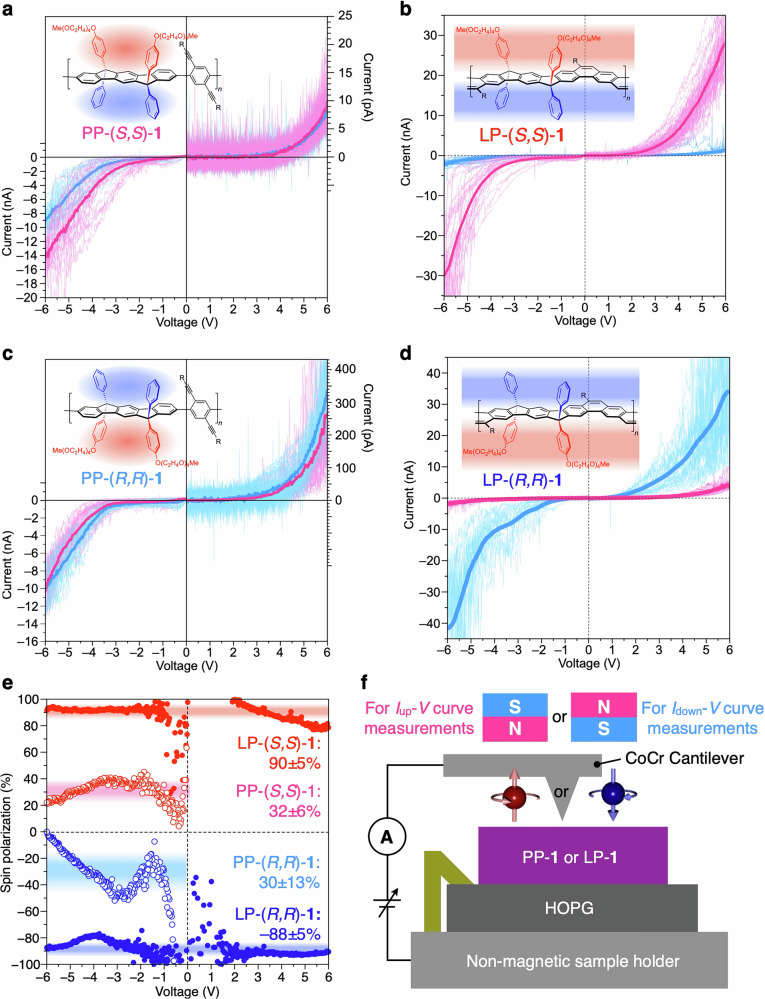
Chirality-induced spin selectivity. **a–d** *I*_up_-*V* and *I*_down_-*V* curves of PP-(*S*,*S*)−**1** (**a**) and LP-(*S*,*S*)−**1** (**b**), PP-(*R*,*R*)-**1** (**c**) and LP-(*R*,*R*)−**1** (**d**). **e** Spin-polarization of PP-(*S*,*S*)−**1** (open red circles) and LP-(*S*,*S*)-**1** (red circles), PP-(*R*,*R*)−**1** (open blue circles) and LP-(*R*,*R*)−**1** (blue circles). **f** Experimental setup for *I*_up_-*V* and *I*_down_-*V* curve measurements using magnetic conductive-AFM equipped with a CoCr ferromagnetic cantilever.

We next investigated chirality-induced spin selectivity (CISS) in thin films of the bifacial ladder polymers [LP-(*S*,*S*)−**1** and LP-(*R*,*R*)−**1**] and their non-ladder precursors [PP-(*S*,*S*)-**1** and PP-(*R*,*R*)-**1**] (Fig. 7). We employed the magnetic-conductive AFM (mc-AFM) protocol on highly oriented pyrolytic graphite (HOPG) that we previously validated for chiral bifacial indacenodithiophene (IDT)-based systems (Fig. 7f)^21,46^. Thin films were prepared by spin-coating CHCl_3_ solutions of the polymers onto freshly cleaved HOPG (see Methods). Tapping-mode AFM confirmed the flatness of the spin-coated films. For both the bifacial ladder polymers and the precursor polymers, the root-mean-square roughness (*R*_a_) was on the order of ~0.5 nm, indicating that uniform films were formed across the substrate (Suppl. Fig. 11). In AFM height images, fibrous features were observed for both classes of samples; however, the spatial resolution was insufficient to resolve their supramolecular organization unambiguously (Suppl. Fig. 11). Film thicknesses were determined by AFM line scans across step edges formed by locally removing the coating; the average value was obtained from approximately eight step measurements per sample. The resulting films had comparable thicknesses of about 15 ~ 20 ± 5 nm for both the bifacial ladder polymers and the precursor polymers (Suppl. Fig. 11).

To investigate CISS behaviour, a neodymium (Nd) magnet was mounted above the paramagnetic CoCr cantilever during mc-AFM measurements, with the magnet oriented so that either the N or S pole faced the cantilever to set its magnetization “up” or “down,” respectively (Fig. 7f). This configuration enabled acquisition of spin-selective current-voltage traces (*I*_up_ and *I*_down_) under well-defined magnetization states. For each state, more than 50 *I*-*V* curves were collected at distinct locations across the film and averaged to obtain representative *I*_up_-*V* and *I*_down_-*V* characteristics. Spin polarization (SP) was defined as SP = (*I*_up_ − *I*_down_)/(*I*_up_ + *I*_down_). Interestingly, LP-(*S*,*S*)−**1** preferentially conducted up-spin current (*I*_up_ > *I*_down_, Fig. 7b), while LP-(*R*,*R*)−**1** showed the opposite preference (*I*_down_ > *I*_up_, Fig. 7d). Opposite spin preferences were thus observed for the two enantiomers, confirming a clear CISS effect in these ladder polymers. The *I*-*V* characteristics of both spin channels were highly symmetric; the favoured channel carried ~30–40 nA, whereas the disfavoured one remained at ~2–3 nA under a bias of ±6 V. Interestingly, the spin-polarization values reached as high as +90 ± 5% for LP-(*S*,*S*)−**1** and –88 ± 5% for LP-(*R*,*R*)-**1** (Fig. 7e). These values are substantially larger than the ~70% spin polarization we previously observed for chiral bifacial IDT-based systems under comparable conditions^21,46^. We attribute this enhancement to the structural fixation of the ladder backbone and the emergence of chiral supramolecular organization in the films, whereas the IDT-based analogues formed nearly amorphous films. Such amplification is consistent with prior reports indicating that CISS signals are highly sensitive to supramolecular packing and long-range chiral organization in condensed phases^47,48^. While it has been experimentally established that chiral organization can enhance spin polarization, the detailed mechanism remains under active investigation^25,26^.

By contrast, the non-ladder precursor polymers exhibited much weaker conduction and spin dependence under the identical mc-AFM/HOPG conditions (Fig. 7a, c). Their *I*-*V* characteristics were strongly bias-asymmetric: in the negative-bias regime, currents were ~10 nA, whereas under positive bias they dropped to the pA level (Fig. 7a, c), too small to reliably determine SP; we therefore restricted quantitative analysis to the negative-bias regime (Fig. 7e). Although the origin of this bias asymmetry is unclear, in the negative-bias regime, PP-(*S*,*S*)−**1** and PP-(*R*,*R*)−**1** still retained enantiospecific asymmetry —*I*_up_ > *I*_down_ and *I*_down_ > *I*_up_, respectively— though the magnitudes were only ~30% of those of the ladder analogues (Fig. 7e). Taken together, the ladder polymers outperform their precursors in both spin polarization and electrical conduction, highlighting how structural fixation of the bifacial, chiral backbone translates molecular chirality into enhanced spin-selective transport.

## Discussion

In this work, we introduced bifacial ladder polymers as an architectural motif in polymer science, enabled by a chirality-assisted synthetic strategy that enforces complete control over facial orientation along a rigid π-conjugated backbone. By designing a *C*_2_-chiral bifacial indenofluorene monomer whose geometry dictates a single ladderization pathway, we realized structurally uniform ladder polymers that preserve monomer-level facial asymmetry throughout the macromolecular architecture. The homochiral bifacial ladder polymers display emergent chiroptical and supramolecular properties that are inaccessible to either their non-ladder precursors or control polymers lacking uniform facial orientation. In solution, ladder formation extends π-conjugation and rigidifies the backbone, while in thin films the homochiral ladders spontaneously assemble into one-handed supramolecular structures, giving rise to CD signals enhanced by two orders of magnitude relative to their molecular counterparts. This amplification reveals a clear chain-length threshold for the onset of chiral supramolecular organization in rigid bifacial systems. Most strikingly, thin films of the homochiral bifacial ladder polymers exhibit robust CISS, with spin-polarization values exceeding ± 90% and opposite signs for the two enantiomers. These values surpass those of their non-ladder counterparts and fall among the highest reported for organic chiral materials. Structural fixation and uniform facial asymmetry thus synergistically enhance not only charge transport but also spin selectivity, underscoring the critical role of molecular and supramolecular order in transducing chirality into macroscopic spin functionality.

Together, these findings establish bifacial ladder polymers as an architectural motif in which facial differentiation and backbone rigidity cooperate to direct supramolecular organization and enable spin-selective transport. Bifacial ladder polymers thus provide a versatile platform for emergent molecular assemblies and functions in organic polymeric materials.

## Methods

### Materials

Unless otherwise stated, all commercial reagents were used as received. Compounds *meso*-**1** and *rac*−**1** were prepared according to ref. ^39^, and compound **6** was prepared according to ref. ^18^. Enantiopure (*S*,*S*)−**1** and (*R*,*R*)-**1** were obtained by chiral-stationary-phase HPLC separation of *rac*-**1** using a Daicel CHIRALPAK IE semi-preparative column (2 cm i.d., 25 cm length), providing each enantiomer in >99% ee. Detailed synthetic procedures and characterization data for the monomers, diboronate comonomer, model compounds, precursor polymers and ladder polymers are provided in the Supplementary Information.

### Synthesis of precursor polymers

A THF/H_2_O (2:1, 2 mL) solution of (*S*,*S*)−**1** (50 mg, 44.3 *µ*mol), bis(boronate) **2** (38.6 mg, 44.3 *µ*mol), K_3_PO_4_ (188 mg, 886 *µ*mol) and P(*t*-Bu)_3_ Pd G2 (2.3 mg, 4.3 *µ*mol) was degassed by three freeze–pump–thaw cycles and purged with N_2_. The mixture was stirred at room temperature for 24 h. The reaction was quenched with water, extracted with CHCl_3_, and the combined organic layers were washed with brine, dried over anhydrous MgSO_4_, and concentrated under reduced pressure. The crude product was purified by preparative GPC (CHCl_3_) to afford PP-(*S*,*S*)−**1** as a brown solid (87%, 67 mg, 38.7 *µ*mol; *M*_n_ = 26.0 kg/mol, *Ð* = 2.7, polystyrene standards). The other stereochemical variants were obtained analogously. ^1^H NMR (600 MHz, CDCl_3_, 25 °C): *δ* (ppm) 7.78 (br, 1H), 7.71 (br, 1H), 7.64 (br, 1H), 7.57 (br, 1H), 7.49 (s, 1H), 7.30 (br, 1H), 7.19 (d, *J* = 4.2 Hz, 2H), 7.06 (s, 3H), 6.67 (d, *J* = 4.2 Hz, 2H), 6.47 (br, 2H), 4.18 (br, 1H), 4.01 (br, 2H), 3.80 (br, 2H), 3.69 (m, 2H), 3.65 (m, 8H), 3.52 (m, 2H), 3.34 (s, 3H), 2.10 (s, 6H), 1.31 (m, 12H), 0.88(br, 6H). FT-IR (KBr): *v* (cm^−1^) 3434 (w), 3056 (m), 2928 (w), 2868 (s), 2200 (s) 1600 (m), 1508 (s), 1456 (m), 1314 (s), 1246 (s), 1127 (m), 829 (m), 700 (s). The other stereochemical variants were obtained analogously.

### Synthesis of ladder polymers

A CH_2_Cl_2_ solution (3 mL) of (*S*,*S*)-PP-**1** (20 mg, 12.3 µmol) and TFA (200 *µ*L) was stirred at 25 °C for 20 h. The reaction mixture was diluted with CH_2_Cl_2_, washed with saturated aqueous NaHCO_3_ and water, dried over anhydrous MgSO_4_, and concentrated under reduced pressure to afford (*S*,*S*)-LP-**1** as a brown solid (87%, 17.4 mg, 10.7 *µ*mol; *M*_n_ = 25.1 kg mol^−^^1^, *Ð* = 3.1, polystyrene standards). The other stereochemical variants were obtained analogously. ^1^H NMR (600 MHz, CDCl_3_, 25 °C): *δ* (ppm) 8.90 (s, 1H), 8.76 (s, 1H), 7.69 (s, 1H), 7.69 (s, 1H), 7.66 (s, 1H), 7.38 (br, 4H), 7.28 (Br, 10H), 6.79 (m, 8H), 4.37 (br, 2H), 4.07 (br, 4H), 3.81 (br, 4H), 3.68 (br, 16H), 3.49 (br, 4H), 3.32 (s, 6H), 2.04 (br, 6H), 2.00 (br, 6H), 1.78 (m, 8H), 1.45 (m, 16H), 0.99 (br, 12H). FT-IR (KBr): *v* (cm^−1^) 3414 (w), 3056 (w), 2929 (m), 2868 (w), 1603 (m), 1508 (m), 1485 (m), 1309 (s), 1257 (s), 1106 (br), 1040 (br), 867 (m), 803 (br), 745 (s), 700 (s). The other stereochemical variants were obtained analogously.

### Spin-polarized conductive-AFM measurement

Spin-polarized conductive-AFM measurements were performed on a Bruker Innova atomic force microscope equipped with a nonmagnetic sample holder (Bruker APSH-0020) and a CoCr-cantilever (Bruker MESP-V2). Samples were fabricated by spin coating 1.0 mg/mL polymer CHCl_3_ solution onto highly oriented pyrolytic graphite (HOPG) at 1000 rpm. A disc-shaped Nd magnet was placed above the CoCr cantilever at a distance of ca. 6 mm, with either the north or south pole facing the cantilever to set the magnetization direction. Spin-selective current–voltage (*I*–*V*) curves were recorded under the two opposite magnetization directions while the cantilever was in contact with the film surface.^21,46^ More than 50 *I*–*V* curves were recorded at different locations for each magnetization direction for each sample. HOPG substrates were purchased from Bruker (ZYB grade) and freshly cleaved just before use. The cantilever was replaced when changing thin films to ensure that a fresh cantilever was used for each sample. The film thicknesses on HOPG were determined using the same AFM facility operated in tapping mode with a silicon cantilever tip (Bruker OLTESPA-R3), by measuring the step height of partially scratched films.

## Supplementary information

Supplementary Information

Transparent Peer Review file

## Source data

Source Data

## Data Availability

The data that support the findings of this study are provided in the Supplementary Information and Source Data files. All data are available from the corresponding authors upon request. Source data are provided with this paper.
