# Supplementary material for: Bifacial ladder polymers enabled by chirality-assisted synthesis that exhibit self-assembly and chirality-induced spin selectivity
Source: Nat Commun. 2026 Aug 3;17:7602. doi: 10.1038/s41467-026-76059-5 (PMC13433944; doi:10.1038/s41467-026-76059-5)
Supplement: Supplementary file 1 [file 41467_2026_76059_MOESM1_ESM.pdf]

Supplementary Information

Bifacial ladder polymers enabled by chirality-assisted synthesis that exhibit self-assembly and chirality-induced spin selectivity

*Fumitaka Ishiwari,[1,2,3,4] Kazuharu Murotani[1] and *Akinori Saeki[1,2]

[1] Department of Applied Chemistry, Graduate School of Engineering, Osaka University, 2-1 Yamadaoka, Suita, Osaka 565-0871, Japan.
[2] Innovative Catalysis Science Division, Institute for Open and Transdisciplinary Research Initiatives (ICS-OTRI), Osaka University, 1-1 Yamadaoka, Suita, Osaka 565-0871, Japan.
[3] PRESTO, Japan Science and Technology Agency (JST), 4-1-8 Honcho, Kawaguchi, Saitama 332-0012, Japan.
[4] Graduate School of Urban Environmental Sciences, Tokyo Metropolitan University, 1-1 Minamiosawa, Hachioji, Tokyo 192-0297, Japan.

*To whom correspondence should be addressed.
E-mail: ishiwari@chem.eng.osaka-u.ac.jp (F.I.)
saeki@chem.eng.osaka-u.ac.jp (A.S.)

Table of Contents

1. Materials S2
2. General S2
3. Theoretical Calculations S3
4. Synthesis S3
5. Supplementary Figures (Supplementary Figs. 1–11) S12
6. Analytical Data (Supplementary Figs. 12–139) S19
7. Supplementary References S112

1. Materials.

Unless otherwise stated, all commercial reagents were used as received. Compounds *meso*-**1**,[1] *rac*-**1**[1] and **6**[2] were prepared according to previously reported procedures and unambiguously characterized by NMR spectroscopy and matrix assisted laser desorption/ionization time of flight (MALDI-TOF) mass spectrometry. Enantiopure (*S*,*S*)-**1** and (*R*,*R*)-**1** were obtained by chiral-stationary-phase HPLC separation of *rac*-**1** using a Daicel CHIRALPAK IE semi-preparative column (2 cm i.d., 25 cm length), providing each enantiomer in >99% ee (Supplementary Fig. 1b). The enantiomeric separation was typically performed using ca. 20 mg scale of racemate per batch, with the sample dissolved in 4 mL of solvent. Their NMR, IR and HR-MS spectra were confirmed to be identical to those of *rac*-**1**, consistent with the expected structures. NMR, IR and HR-MS spectra of (*S*,*S*)-**1** and (*R*,*R*)-**1** are shown in Supplementary Figs. 12–25.

2. General.

NMR spectroscopy measurements in solution were carried out at 25 °C on a JEOL JNM-ECS400 spectrometer (400 MHz for ^{1}H and 100 MHz for ^{13}C) or on a Bruker AVANCE-III spectrometer (600 MHz for ^{1}H and 151 MHz for ^{13}C). Chemical shifts (δ) are expressed relative to the resonances of the residual non-deuterated solvents for ^{1}H [DMSO-d_6: ^{1}H(δ) = 2.50 ppm] and ^{13}C [DMSO-d_6: ^{13}C(δ) = 39.52 ppm]. Absolute values of the coupling constants are given in Hertz (Hz), regardless of their sign. Multiplicities are abbreviated as singlet (s), doublet (d), triplet (t) and multiplet (m). ^{1}H NMR spectra of LP-**1**s were recorded with a relaxation delay of 10 s. Infrared (IR) spectra were recorded at 25 °C on a JASCO model FT/IR-4700AC Fourier-transform infrared spectrometer. High-resolution fast atom bombardment mass spectrometry (FAB-MS) was performed on a JEOL JMS-700. High-resolution matrix assisted laser desorption/ionization time of flight (MALDI-TOF) mass spectrometry measurements were performed on a JEOL model JMS-S3000 mass spectrometer. The molecular weight (weight-averaged: M_w, number-averaged: M_n) and molar-mass dispersity ($Đ$) were measured using size exclusion chromatography (SEC, a Shimadzu Corp. LC-20AT/CBM-20A/CTO-20A/SPD-M20A) with polystyrene standards in chloroform (40 °C) as an eluent. UV-vis absorption spectra were recorded at 25 °C on a JASCO model Jasco V-730 UV-vis spectrophotometer. Circular dichroism (CD) and linear dichroism (LD) spectra were recorded on a JASCO J-820 spectropolarimeter. Thermogravimetric analysis (TGA) and differential Scanning Calorimetry (DSC) were performed on a SHIMADZU model TGA-50 analyzer and a Netzsch DSC 204F1, respectively. Atomic force microscopy (AFM) measurements of the thin-films of the (*S*,*S*)-**1** and (*R*,*R*)-**1** were performed on a Bruker Innova atomic force microscope operated in tapping mode using a silicon cantilever tip (a Bruker OLTESPA-R3). 2D GI-XRD measurements were performed on the BL13XU beamline at SPring-8 (Japan Synchrotron Radiation Research Institute, JASRI), using 12.39 keV (λ = 1 Å) X-rays at the grazing incidence angle of 0.12°. 2D diffraction patterns were recorded with a 2D detector, Dectris PILATUS 300K. Time-resolved

microwave conductivity (TRMC) was performed for the films prepared on a quartz substrate at 25 °C. The microwave frequency and its power were ~9 GHz and ~3 mW, respectively. A third harmonic generation (355 nm) of a Nd:YAG laser (Continuum Inc., Surelite II, 5–8 ns pulse duration, 10 Hz) was used for the excitation (incident photon density I_0 = 4.6 × 10^{15} photons cm^{-2} $pulse^{-1}$). The photoconductivity ($\Delta\sigma = A^{-1}\,\Delta P_r\,P_r^{-1}$ where A is the sensitivity factor, P_r is the reflected microwave power, and ΔP_r is the change in P_r upon exposure to light) was converted into the product of the quantum yield (φ) and sum of the charge carrier mobilities $\Sigma\mu$ ($= \mu_+ + \mu_-$) using the relationship $\varphi\Sigma\mu = \Delta\sigma(eI_0F_{light})^{-1}$, where e and F_{Light} are the electron charge and correction (or filling) factor, respectively. The experiments were performed at room temperature in the air.

3. Theoretical Calculation.

Theoretical calculations were conducted using DFT and TD-DFT methods at the B3LYP/6-31G(d,p) level of theory with the Gaussian 16 program package.[3] To simplify the calculations, simplified models of (*S*,*S*)-**1** and (*R*,*R*)-**1** were used, in which the tetraethylene glycoloxy moieties were replaced by methoxy groups. The optimized structure of the (*R*,*R*)-**1** model was obtained by mirror inversion of the optimized (*S*,*S*)-**1** model. Cartesian coordinates of the optimized geometries of the simplified models of (*S*,*S*)-**1** and Model-LP-(*S*,*S*)-**1**, calculated in vacuum in the singlet state at the B3LYP/6-31G(d,p) level, are provided in the Source Data files. Optimized geometries were verified by frequency computations as minima with no imaginary frequencies. Transition electric dipole moments (**μ**) in atomic units, obtained from Gaussian, were converted to CGS-Gaussian units (esu·cm) by multiplying each component by 2.5435 × 10^{-18}, following the literature method[4] (Supplementary Fig. 9). Molecular mechanics simulations of LP-(*S*,*S*)-**1** (Fig. 3e) were performed using PerkinElmer ChemBio3D with the Molecular Mechanics 2 (MM2) force field.

4. Synthesis.

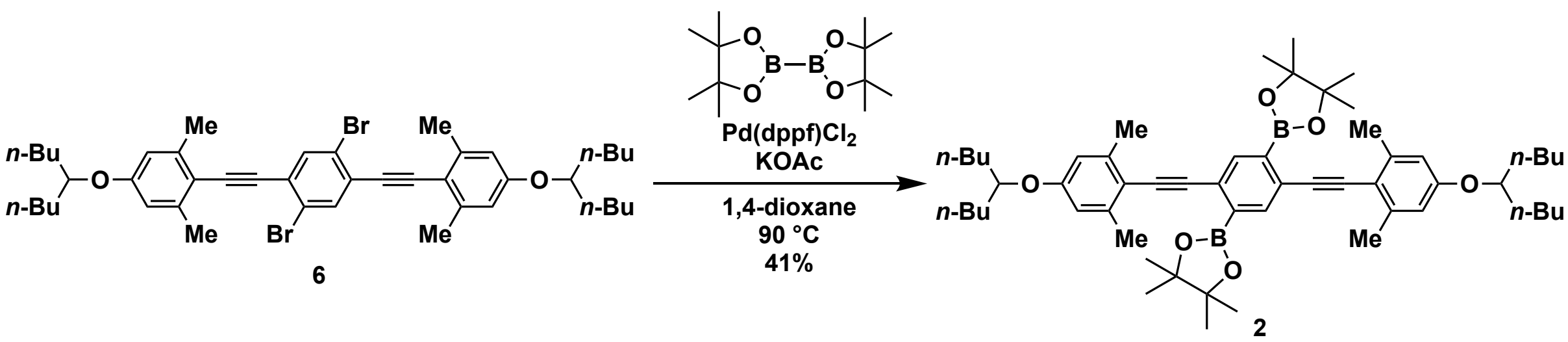

2,2'-(2,5-bis((2,6-dimethyl-4-(nonan-5-yl)phenyl)ethynyl)-1,4-phenylene)bis(4,4,5,5-tetra methyl-1,3,2-dioxaborolane) (2). Under nitrogen, 1,4-dioxane solution (6 ml) of **6**[3] (2.00 g, 2.58 mmol), bis(pinacolato)diboron (2.62 g, 10.3 mmol), Pd(dppf)Cl_2 (188 mg, 0.257 mmol) and KOAc (1.52 g, 15.5 mmol) was stirred for 120 h at 90 ºC. The resultant mixture was diluted

with $CHCl_3$, washed with water and brine, dried over anhydrous $MgSO_4$ and then evaporated under a reduced pressure. The residue was purified by silica gel column chromatography (*n*-hexane/ethyl acetate = 50/1) to afford **2** as a yellow powder (41%, 887 mg, 1.06 mmol): ^{1}H NMR (600 MHz, $CDCl_3$, 25 ºC): δ (ppm) 7.95 (s, 2H), 6.59 (s, 4H), 4.23 (q, J = 8.8 Hz, 2H), 2.55 (s, 12H), 1.64 (m, 8H), 1.34 (m, 40H), 0.90 (t, J = 6.8 Hz, 12H). ^{13}C NMR (151 MHz, $CDCl_3$, 25 ºC): δ 158.27, 142.51, 140.07, 132.35, 127.25, 115.76, 114.11, 97.20, 91.22, 84.17, 77.71, 33.78, 27.69, 24.96, 22.94, 21.84, 14.21, FT-IR (KBr): ν (cm^{-1}) 3467 (w), 2952 (w), 2204 (s), 1598 (s), 1491 (m), 1389 (m), 1321 (s), 1135 (m), 1092 (s), 855 (s). High-resolution FAB-MS: calced. for $C_{56}H_{80}B_2O_6$ [M]: *m/z* = 870.6141; found:870.6149. NMR, IR and HR-MS spectra of **2** are shown in Supplementary Figs. 26–33.

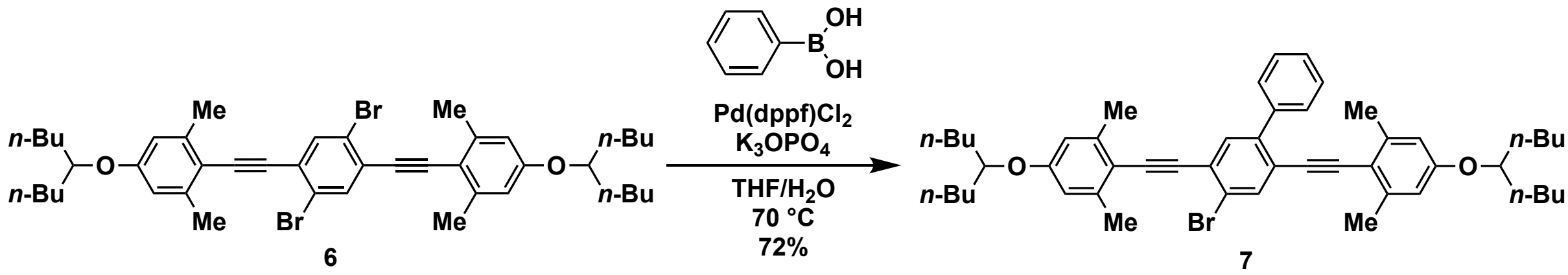

4-bromo-2,5-bis((2,6-dimethyl-4-(nonan-5-yloxy)phenyl)ethynyl)-1,1'-biphenyl (7). Under nitrogen, degassed THF/H_2O solution (4/1, v/v, 15 ml) of **6** (1.00 g, 1.29 mmol), phenylboronic acid (162 mg, 1.33 mmol), $Pd(PPh_3)_2Cl_2$ (90 mg, 0.129 mmol) and K_3PO_4 (547 mg, 2.58 mmol) was stirred for 20 h at 70 ºC. The resultant mixture was diluted with $CHCl_3$, washed with water and brine, dried over anhydrous $MgSO_4$ and then evaporated under a reduced pressure. The residue was purified by silica gel column chromatography (Hexane/$CHCl_3$ = 4/1) to afford **7** as a yellow solid (72%, 723 mg, 0.93 mmol). ^{1}H NMR (400MHz, $CDCl_3$, 25 ºC): δ (ppm) 7.85 (s, 1H), 7.58 (dd, J = 4.1 Hz 2H), 7.52 (s, 1H), 7.40 (m, 3H), 6.61 (s, 2H), 6.52 (s, 2H), 4.22 (m, 2H), 2.52 (s, 6H), 2.21 (s, 6H), 1.62 (m, 8H), 1.34 (m, 16H), 0.89 (q, J = 7.5 Hz 12H). ^{13}C NMR (151 MHz, $CDCl_3$, 25 ºC): δ 158.89, 158.70, 142.75, 142.53, 142.26, 139.67, 135.91, 133.59, 129.21, 128.31, 127.85, 125.62, 123.55, 123.03, 114.57, 114.55, 114.28, 114.13, 94.84, 94.50, 93.97, 93.23, 77.71, 77.66, 77.33, 33.73, 33.71, 27.62, 22.89, 22.87, 21.74, 21.29, 14.17. FT-IR (KBr): ν (cm^{-1}) 3442 (w), 3055 (m), 2934 (m), 2202 (s), 1598 (m), 1486 (m), 1319 (m), 1125 (m), 1041 (m), 698(s). High-resolution FAB-MS: calced. for $C_{56}H_{80}B_2O_6^{+}$ $[M]^{+}$: *m/z* = 772.3855; found:772.3848. NMR, IR and HR-MS spectra of **7** are shown in Supplementary Figs. 34–41.

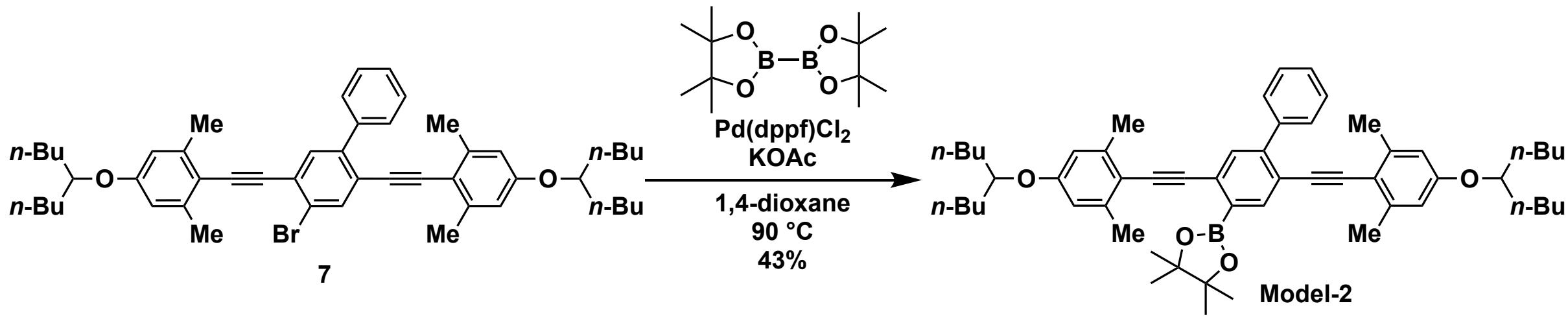

2-(2,5-bis((2,6-dimethyl-4-(nonan-5-yloxy)phenyl)ethynyl)-[1,1'-biphenyl]-4-yl)-4,4,5,5-tetramethyl-1,3,2-dioxaborolane (Model-2). Under nitrogen, 1,4-dioxane solution (1.5 ml) of **7** (100 mg, 0.129 mmol), bis(pinacolato)diboron (68 mg, 0.258 mmol), Pd(dppf)Cl_2 (9.5 mg, 12 μmol) and KOAc (40 mg, 0.387 mmol) was stirred for 72 h at 90 ºC. The resultant mixture was diluted with $CHCl_3$, washed with water and brine, dried over anhydrous $MgSO_4$ and then evaporated under a reduced pressure. The residue was purified by silica gel column chromatography (*n*-hexane/ethyl acetate = 10/1) to afford **Model-2** as a brown solid (43%, 46 mg, 0.055 mmol). ^{1}H NMR (600 MHz, $CDCl_3$, 25 ºC): δ (ppm) 8.06 (s, 1H), 7.60 (dd, J = 4.2 Hz 2H), 7.40(m, 3H), 6.59 (s, 2H), 6.51 (s, 2H), 4.21 (m, 2H), 2.55 (s, 6H), 2.22 (s, 2H), 1.61 (m, 8H), 1.34 (m, 28H). ^{13}C NMR (151 MHz, $CDCl_3$, 25 ºC): δ 158.39, 158.29, 145.25, 142.56, 142.28, 140.54, 140.31, 134.06, 129.30, 128.44, 128.17, 127.65, 121.45, 115.51, 115.17, 114.08, 114.04, 97.15, 95.60, 91.86, 91.60, 84.09, 77.66, 77.61, 77.29, 33.72, 33.69, 27.61, 27.60, 24.92, 22.86, 22.84, 21.74, 21.34, 14.13. FT-IR (KBr): ν (cm^{-1}) 3467 (w), 2952 (w), 2204 (s), 1598 (s), 1491 (m), 1389 (m), 1321 (s), 1135 (m), 1092 (s), 855 (s). High-resolution FAB-MS: calced. for $C_{56}H_{80}B_2O_6^+$ [M]$^+$: m/z = 870.6141; found:870.6149. NMR, IR and HR-MS spectra of **Model-2** are shown in Supplementary Figs. 42–49.

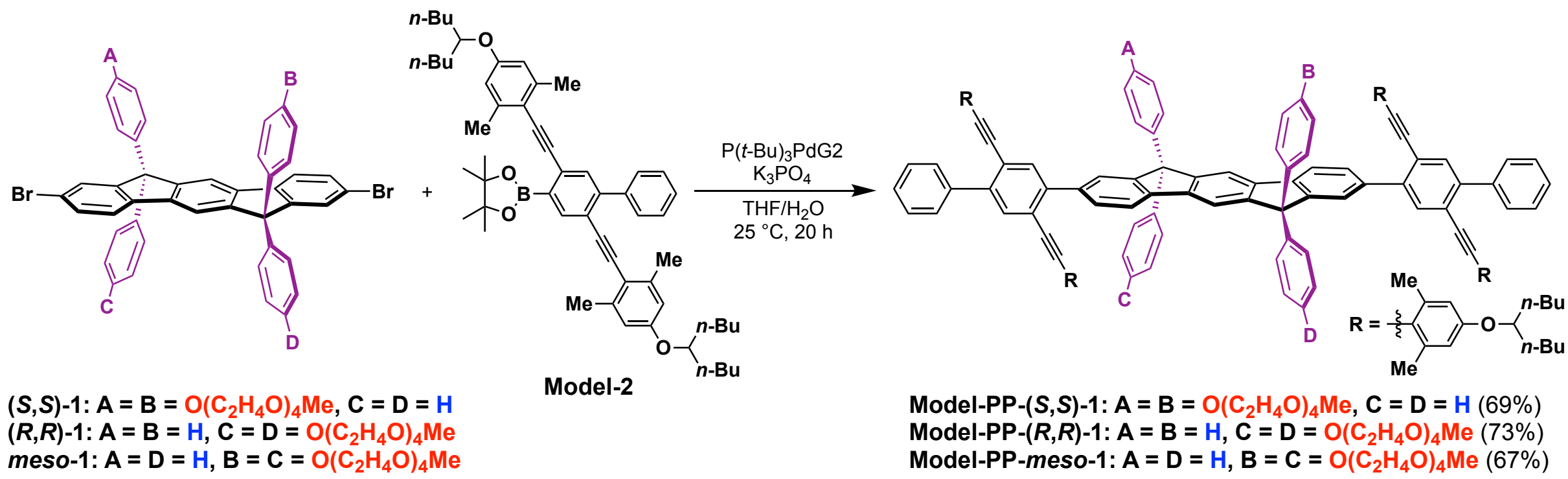

Model-PP-(*S,S*)-1, Model-PP-(*R,R*)-1 and Model-PP-*meso*-1. A THF/H_2O (2:1, 2 mL) solution of (*S,S*)-**1** (28 mg, 24.4 μmol), Model-**2** (50 mg, 60.9 μmol), P(*t*-Bu)3 Pd G2 (1.3 mg, 2.4 μmol) and K_3PO_4 (31 mg, 146 μmol) was degassed by three freeze–pump–thaw cycles and purged with N_2. The mixture was stirred at room temperature for 24 h. The reaction was quenched with water and extracted with $CHCl_3$. The combined organic layers were washed with brine, dried over anhydrous $MgSO_4$ and concentrated under reduced pressure. The residue was purified by silica gel column chromatography (*n*-hexane/ethyl acetate = 2/1) to afford Model-PP-(*S,S*)-**1** as a brown solid (69%, 40 mg, 16.8 μmol). The other stereochemical variants were

obtained analogously.

Model-PP-(*S,S*)-1 (69%) : ^{1}H NMR (600 MHz, $CDCl_3$, 25 ºC): δ (ppm) 7.80 (s, 2H), 7.74 (d, J = 4.0 Hz, 2H), 7.68 (dd, J = 4.0 Hz, 2H), 7.63 (m, 8H), 7.53 (s, 2H), 7.39 (m, 6H), 7.29 (m, 4H), 7.21 (d, J = 4.4 Hz, 4H), 7.07 (m, 4H), 6.69 (d, J = 4.4 Hz, 4H), 6.51 (s, 4H), 6.48 (s, 4H), 4.18 (m, 4H), 4.01 (t, J = 5.2 Hz, 4H), 3.80 (t, J = 4.4 Hz, 4H), 3.69 (m, 4H), 3.64 (m, 16H), 3.52 (m, 4H), 3.34 (s, 6H), 2.20 (s, 12H), 2.12 (s, 12H), 1.60 (m, 16H), 1.30 (m, 32H), 0.88 (m, 24H). ^{13}C NMR (151 MHz, $CDCl_3$, 25 ºC) : δ 158.51, 158.42, 157.67, 151.78, 151.69, 146.28, 142.42, 142.33, 141.95, 140.28, 140.14, 139.68, 139.67, 138.29, 133.99, 133.86, 129.56, 129.40, 128.85, 128.39, 128.27, 127.66, 127.37, 126.69, 122.34, 119.83, 118.03, 115.07, 115.00, 114.31, 114.20, 114.16, 95.70, 95.63, 92.22, 92.20, 77.66, 77.36, 72.05, 70.91, 70.76, 70.65, 69.83, 67.38, 64.77, 59.16, 33.74, 27.64, 22.92, 21.35, 21.33, 14.21. FT-IR (KBr): ν (cm^{-1}) 3486 (w), 3056 (m), 2930 (s), 2868 (s), 2200 (s), 1601 (s), 1508(s) 1487 (s), 1457 (m), 1315 (m), 1248 (s), 1165 (s), 1128 (br), 1041 (s), 831 (m), 700 (s). High-resolution MALDI-TOF-MS: calced. for $C_{162}H_{186}O_{14}Na^+$ $[M+Na]^+$: m/z = 2355.3837; found: 2378.3728. NMR, IR and HR-MS spectra of Model-PP-(*S,S*)-**1** are shown in Supplementary Figs. 50–57.

Model-PP-(*R,R*)-1 (73%) : ^{1}H NMR (600 MHz, $CDCl_3$, 25 ºC): δ (ppm) 7.80 (s, 2H), 7.74 (d, J = 4.0 Hz, 2H), 7.68 (dd, J = 4.0 Hz, 2H), 7.63 (m, 8H), 7.52 (s, 2H), 7.39 (m, 6H), 7.29 (m, 4H), 7.21 (d, J = 4.4 Hz, 4H), 7.07 (dd, J = 4.4 Hz, 4H), 6.69 (d, J = 4.4 Hz, 4H), 6.51 (s, 4H), 6.48 (s, 4H), 4.18 (m, 4H), 4.01 (t, J = 5.2 Hz, 4H), 3.80 (t, J = 4.4 Hz, 4H), 3.69 (m, 4H), 3.64 (m, 16H), 3.52 (m, 4H), 3.34 (s, 6H), 2.20 (s, 12H), 2.12 (s, 12H), 1.60 (m, 16H), 1.30 (m, 32H), 0.88 (m, 24H). ^{13}C NMR (151 MHz, $CDCl_3$, 25 ºC) : δ 158.51, 158.42, 157.67, 151.78, 151.69, 146.28, 142.42, 142.33, 141.95, 140.28, 140.14, 139.68, 139.67, 138.29, 133.99, 133.86, 129.56, 129.40, 128.85, 128.39, 128.27, 127.65, 127.37, 126.69, 122.34, 119.83, 118.03, 115.07, 115.00, 114.31, 114.20, 114.16, 95.70, 95.63, 92.22, 92.20, 77.66, 77.36, 72.05, 70.91, 70.76, 70.65, 69.83, 67.38, 64.77, 59.16, 33.74, 27.64, 22.92, 21.35, 21.33, 14.21. FT-IR (KBr): ν (cm^{-1}) 3477 (w), 3056 (m), 2930 (s), 2858 (s), 2201 (s), 1601 (m), 1487 (m), 1377 (s), 1315 (br), 1248 (s), 1165 (s), 1128 (br), 1041 (s), 831 (m), 700 (s). High-resolution MALDI-TOF-MS: calced. for $C_{162}H_{186}O_{14}Na^+$ $[M+Na]^+$: m/z = 2355.3837; found: 2378.370. NMR, IR and HR-MS spectra of Model-PP-(*R,R*)-**1** are shown in Supplementary Figs. 58–65.

Model-PP-*meso*-1 (67%) : ^{1}H NMR (600 MHz, $CDCl_3$, 25 ºC): δ (ppm) 7.80 (s, 2H), 7.74 (d, J = 4.0 Hz, 2H), 7.68 (dd, J = 4.0 Hz, 2H), 7.63 (m, 8H), 7.53 (s, 2H), 7.39 (m, 6H), 7.29 (m, 4H), 7.21 (d, J = 4.4 Hz, 4H), 7.07 (m, 4H), 6.69 (d, J = 4.4 Hz, 4H), 6.51 (s, 4H), 6.48 (s, 4H), 4.18 (m, 4H), 4.01 (t, J = 5.2 Hz, 4H), 3.80 (t, J = 4.4 Hz, 4H), 3.69 (m, 4H), 3.64 (m, 16H), 3.52 (m, 4H), 3.34 (s, 6H), 2.20 (s, 12H), 2.12 (s, 12H), 1.60 (m, 16H), 1.30 (m, 32H), 0.88 (m, 24H). ^{13}C NMR (151 MHz, $CDCl_3$, 25 ºC): δ 158.50, 158.41, 157.65, 151.77, 151.69, 146.29, 142.43, 142.34, 141.93, 140.27, 140.13, 139.67, 139.67, 138.29, 134.01, 133.86, 129.56, 129.40, 128.86, 128.38, 128.27, 127.67, 127.37, 126.70, 122.34, 119.83, 118.02, 115.06, 114.99, 114.31, 114.18, 114.15, 95.70, 95.62, 92.22, 92.18, 77.68, 77.37, 72.04, 70.89, 70.74, 70.63, 69.13, 67.36, 64.76, 59.16, 33.74, 27.64, 22.92, 21.35, 21.33, 14.21. FT-IR (KBr): ν (cm^{-1}) 3470 (w), 3056 (m), 2932 (s), 2869 (s), 2201 (s), 1602 (s), 1507(s) 1487 (s), 1465 (m), 1315 (m), 1248 (s), 1165 (s), 1128 (br), 1040 (s), 829 (m), 700 (s). High-resolution MALDI-TOF- MS:

calced. for $C_{162}H_{186}O_{14}Na^+$ $[M+Na]^+$: *m/z* = 2355.3837; found:2378.3785. NMR, IR and HR-MS spectra of Model-PP-*meso*-**1** are shown in Supplementary Figs. 66–73.

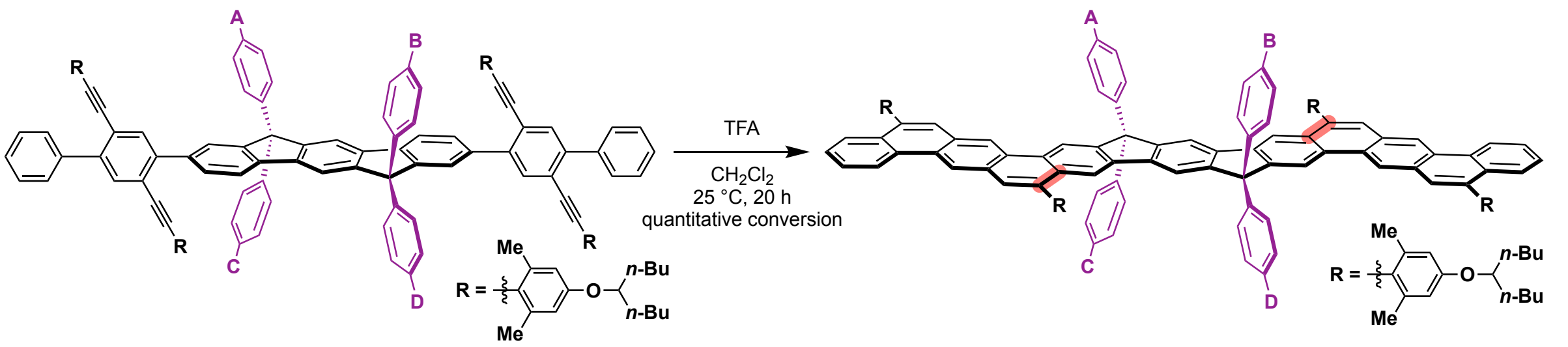

Model-LP-(*S,S*)-1, Model-LP-(*R,R*)-1 and Model-LP-*meso*-1. A CH_2Cl_2 solution (3 ml) of Model-PP-(*S,S*)-**1** (20 mg, 8.48 μmol) and TFA (200 μL) was stirred for 20 h at 25 °C. The resultant mixture was diluted with CH_2Cl_2, washed with saturated aqueous $NaHCO_3$ and water, dried over anhydrous $MgSO_4$ and then evaporated under a reduced pressure to yield Model-LP-(*S,S*)-**1** as a brown solid (80%, 16.9 mg, 6.79 μmol).

Model-LP-(*S,S*)-1 (80%) : ^{1}H NMR (600 MHz, $CDCl_3$, 25 ºC): δ (ppm) 9.10 (s, 2H), 8.97 (s, 2H), 8.88 (d, *J* = 4.2 Hz, 2H), 7.74 (m, 8H), 7.68 (t, *J* = 7.2 Hz, 2H), 7.49 (m, 4H), 7.41 (d, *J* = 3.9 Hz, 4H), 7.28 (m, 8H), 7.24 (m, 2H), 6.81 (m, 8H), 6.75 (s, 4H), 4.38 (t, *J* = 5.7 Hz, 2H), 4.32 (t, *J* = 5.7 Hz, 2H), 4.06 (t, *J* = 4.8 Hz, 4H), 3.80 (t, *J* = 4.8 Hz, 4H), 3.68 (t, *J* = 4.8 Hz, 4H), 3.62 (m, 16H), 3.48 (t, *J* = 4.8 Hz, 4H), 3.31 (s, 6H), 2.04 (d, *J* = 6.0 Hz, 12H), 1.97 (d, *J* = 3.0 Hz, 12H), 1.75 (m, 16H), 1.44 (m, 32H), 0.99 (m, 24H). ^{13}C NMR (151 MHz, $CDCl_3$, 25 ºC): δ 157.79, 157.68, 152.25, 151.01, 146.50, 140.65, 139.50, 138.51, 138.48, 138.46, 138.38, 137.80, 137.38, 131.98, 131.88, 131.63, 130.67, 130.62, 130.47, 130.42, 129.61, 129.15, 128.88, 128.52, 128.42, 128.17, 127.09, 126.77, 126.63, 126.37, 123.07, 121.97, 121.78, 120.63, 118.32, 117.36, 115.06, 114.83, 114.62, 114.57, 114.35, 77.73, 77.68, 71.91, 70.77, 70.63, 70.60, 70.50, 69.70, 67.27, 64.5560.41, 59.00, 33.79, 33.69, 33.66, 27.70, 27.68, 27.66, 22.95, 22.92, 21.10, 21.07, 20.70, 20.68, 14.21, 14.17. FT-IR (KBr): *ν* (cm^{-1}) 3434 (w), 3058 (m), 2928 (s), 2863 (s), 2355 (m), 1602 (m), 1507 (s), 1482 (m), 1372 (s), 1309 (s), 1247 (s), 1144 (m), 1042 (s), 904 (s), 864 (m), 765 (s). High-resolution MALDI-TOF-MS: calced. for $C_{162}H_{186}O_{14}^+$ $[M]^+$: *m/z* = 2355.3837; found:2355.3848. NMR, IR and HR-MS spectra of Model-LP-(*S,S*)-**1** are shown in Supplementary Figs. 74–81.

Model-LP-(*R,R*)-1 (85%) : ^{1}H NMR (600 MHz, $CDCl_3$, 25 ºC): δ (ppm) 9.10 (s, 2H), 8.97 (s, 2H), 8.88 (d, *J* = 4.2 Hz, 2H), 7.74 (m, 8H), 7.68 (t, *J* = 7.2 Hz, 2H), 7.49 (m, 4H), 7.41 (d, *J* = 3.9 Hz, 4H), 7.28 (m, 8H), 7.24 (m, 2H), 6.81 (m, 8H), 6.75 (s, 4H), 4.38 (t, *J* = 5.7 Hz, 2H), 4.32 (t, *J* = 5.7 Hz, 2H), 4.06 (t, *J* = 4.8 Hz, 4H), 3.80 (t, *J* = 4.8 Hz, 4H), 3.68 (t, *J* = 4.8 Hz, 4H), 3.62 (m, 16H), 3.48 (t, *J* = 4.8 Hz, 4H), 3.31 (s, 6H), 2.04 (d, *J* = 6.0 Hz, 12H), 1.97 (d, *J* = 3.0 Hz, 12H), 1.75 (m, 16H), 1.44 (m, 32H), 0.99 (m, 24H). ^{13}C NMR (151 MHz, $CDCl_3$, 25 ºC): δ 157.79, 157.68, 152.25, 151.01, 146.50, 140.65, 139.50, 138.51, 138.48, 138.46, 138.38,

137.80, 137.38, 131.98, 131.88, 131.63, 130.67, 130.62, 130.47, 130.42, 129.61, 129.15, 128.88, 128.52, 128.42, 128.17, 127.09, 126.77, 126.63, 126.37, 123.07, 121.97, 121.78, 120.63, 118.32, 117.36, 115.06, 114.83, 114.62, 114.57, 114.35, 77.73, 77.68, 71.91, 70.77, 70.63, 70.60, 70.50, 69.70, 67.27, 64.5560.41, 59.00, 33.79, 33.69, 33.66, 27.70, 27.68, 27.66, 22.95, 22.92, 21.10, 21.07, 20.70, 20.68, 14.21, 14.17. FT-IR (KBr): v (cm^{-1}) 3440 (w), 3055 (m), 2929 (s), 2855 (s), 2360 (m), 1722 (s), 1603 (m), 1508 (s), 1482 (m), 1454 (m), 1309 (s), 1249 (s), 1146 (m), 1044 (s), 905 (s), 853 (m), 765 (s). High-resolution MALDI-TOF-MS: calced. for $C_{162}H_{186}O_{14}^+$ $[M]^+$: m/z = 2355.3837; found:2355.3834. NMR, IR and HR-MS spectra of Model-LP-(*R,R*)-**1** are shown in Supplementary Figs. 82–89.

Model-LP-*meso*-1 (84%) : ^{1}H NMR (600 MHz, $CDCl_3$, 25 ºC): δ (ppm) 9.10 (s, 2H), 8.97 (s, 2H), 8.88 (d, J = 4.2 Hz, 2H), 7.74 (m, 8H), 7.68 (t, J = 7.2 Hz, 2H), 7.49 (m, 4H), 7.41 (d, J = 3.9 Hz, 4H), 7.28 (m, 8H), 7.24 (m, 2H), 6.81 (m, 8H), 6.75 (s, 4H), 4.38 (t, J = 5.7 Hz, 2H), 4.32 (t, J = 5.7 Hz, 2H), 4.06 (t, J = 4.8 Hz, 4H), 3.80 (t, J = 4.8 Hz, 4H), 3.68 (t, J = 4.8 Hz, 4H), 3.62 (m, 16H), 3.48 (t, J = 4.8 Hz, 4H), 3.31 (s, 6H), 2.04 (d, J = 6.0 Hz, 12H), 1.97 (d, J = 3.0 Hz, 12H), 1.75 (m, 16H), 1.44 (m, 32H), 0.99 (m, 24H). ^{13}C NMR (151 MHz, $CDCl_3$, 25 ºC): δ 157.93, 157.81, 152.39, 151.15, 146.62, 140.78, 139.63, 138.64, 138.61, 138.51, 138.64, 137.94, 137.52, 132.12, 132.02, 131.76, 130.81, 130.76, 130.60, 130.56, 129.73, 129.29, 129.02, 128.68, 128.56, 128.30, 127.22, 126.91, 126.77, 126.51, 123.21, 122.10, 121.92, 120.76, 118.46, 117.50, 115.21, 114.96, 114.71, 114.57, 114.48, 77.87, 77.82, 72.04, 70.90, 70.76, 70.74, 70.63, 69.82, 67.41, 64.68, 59.13, 33.92, 33.83, 33.80, 27.84, 27.82, 27.79, 23.08, 23.06, 21.24, 21.19, 20.84, 20.82, 14.34, 14.31. FT-IR (KBr): v (cm^{-1}) 3425 (w), 3056 (m), 2925 (s), 2863 (s), 2360 (m), 1728 (s), 1602 (m), 1508 (s), 1482 (m), 1375 (s), 1311 (s), 1260 (s), 1140 (w), 1107 (s), 1042 (s), 905 (s), 866 (m), 802 (w), 766 (s), 700 (s). High-resolution MALDI-TOF-MS: calced. for $C_{162}H_{186}O_{14}^+$ $[M]^+$: m/z = 2355.3837; found:2355.3866. NMR, IR and HR-MS spectra of Model-LP-*meso*-**1** are shown in Supplementary Figs. 90–99.

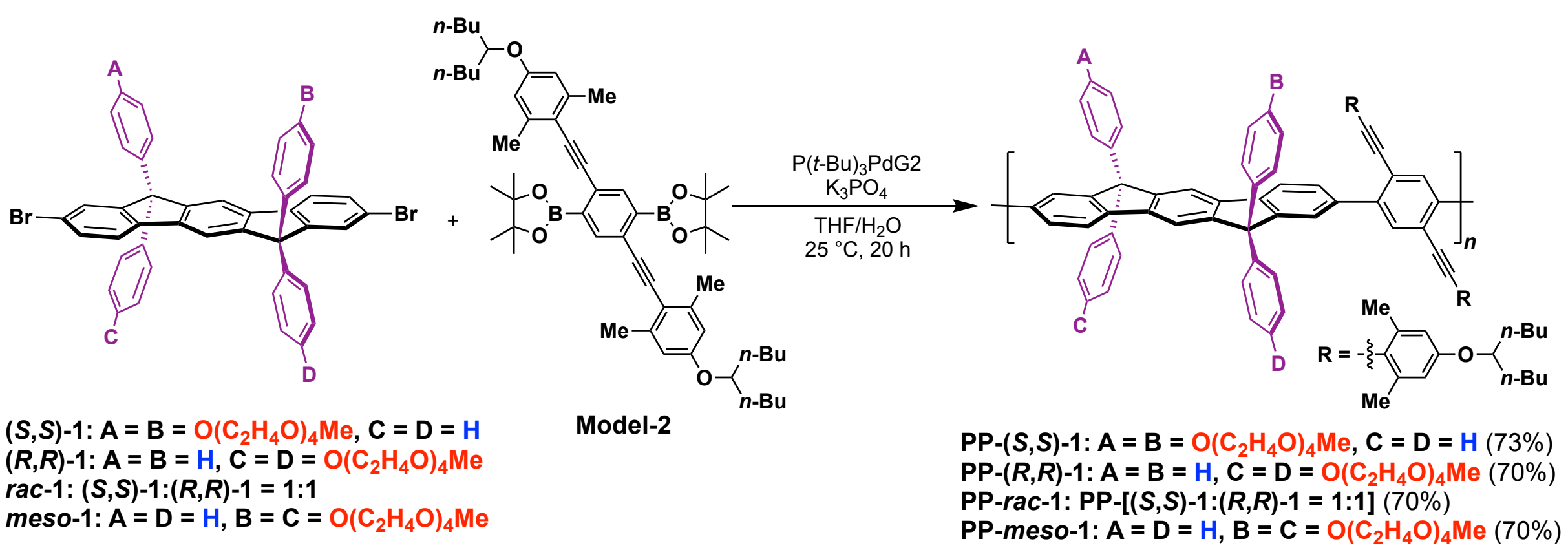

PP-(*S,S*)-1 and PP-(*R,R*)-1, PP-*meso*-1, PP-*rac*-1.

A THF/H_2O (2:1, 2 mL) solution of (*S,S*)-**1** (50 mg, 44.3 µmol), bis(boronate) **2** (38.6 mg, 44.3 µmol), K_3PO_4 (188 mg, 886 μmol), and P(t-Bu)$_3$ Pd G2 (2.3 mg, 4.3 μmol) was degassed by

three freeze–pump–thaw cycles and purged with N_2. The mixture was stirred at room temperature for 24 h. The reaction was quenched with water, extracted with $CHCl_3$, and the combined organic layers were washed with brine, dried over anhydrous $MgSO_4$, and concentrated under reduced pressure. The crude product was purified by preparative GPC ($CHCl_3$) to afford PP-(*S*,*S*)-**1** as a brown solid (73%, 67 mg, 38.7 μmol; M_n = 26.0 kg/mol, $Đ$ = 2.7, polystyrene standards). The other stereochemical variants were obtained analogously.

PP-(*S,S*)-1 (73%, M_n = 26.0 kg/mol, M_w/M_n = 2.7, polystyrene standards). ^{1}H NMR (400 MHz, $CDCl_3$, 25 ºC): δ (ppm) 7.78 (br, 1H), 7.71 (br, 1H), 7.64 (br, 1H), 7.57 (br, 1H), 7.49 (s, 1H), 7.30 (br, 1H), 7.19 (d, J = 4.2 Hz, 2H), 7.06 (s, 3H), 6.67 (d, J = 4.2 Hz, 2H), 6.47 (br, 2H), 4.18 (br, 1H), 4.01 (br, 2H), 3.80 (br, 2H), 3.69 (m, 2H), 3.65 (m, 8H), 3.52 (m, 2H), 3.34 (s, 3H), 2.10 (s, 6H), 1.31 (m, 12H), 0.88(br, 6H). FT-IR (KBr): ν (cm^{-1}) 3434 (w), 3056 (m), 2928 (w), 2868 (s), 2200 (s) 1600 (m), 1508 (s), 1456 (m), 1314 (s), 1246 (s), 1127 (m), 829 (m), 700 (s). NMR and IR spectra and SEC profile of PP-(*S,S*)-**1** are shown in Supplementary Figs. 100–104.

PP-(*R,R*)-1 (70%, M_n = 28.8 kg/mol, M_w/M_n = 1.9, polystyrene standards). ^{1}H NMR (600 MHz, $CDCl_3$, 25 ºC): δ (ppm) 7.78 (br, 1H), 7.71 (br, 1H), 7.64 (br, 1H), 7.57 (br, 1H), 7.49 (s, 1H), 7.30 (br, 1H), 7.19 (d, J = 4.2 Hz, 2H), 7.06 (s, 3H) 6.67 (d, J = 4.2 Hz, 2H), 6.47 (br, 2H), 4.18 (br, 1H), 4.01 (br, 2H), 3.80 (br, 2H), 3.69 (m, 2H), 3.65 (m, 8H), 3.52 (m, 2H), 3.34 (s, 3H), 2.10 (s, 6H), 1.31 (m, 12H), 0.88(br, 6H). FT-IR (KBr): ν (cm^{-1}) 3468 (w), 3033 (m), 2929 (w), 2857 (w), 2197 (s) 1600 (s), 1508 (m), 1456 (m), 1310 (w), 1246 (s), 1122 (m), 1040 (br), 832 (m), 697 (s). NMR and IR spectra and SEC profile of PP-(*R,R*)-**1** are shown in Supplementary Figs. 105–109.

PP-*meso*-1 (70%, M_n = 28.5 kg/mol, M_w/M_n = 2.0, polystyrene standards). ^{1}H NMR (600 MHz, $CDCl_3$, 25 ºC): δ (ppm) 7.78 (br, 1H), 7.71 (br, 1H), 7.64 (br, 1H), 7.57 (br, 1H), 7.49 (s, 1H), 7.30 (br, 1H), 7.19 (d, J = 4.2 Hz, 2H), 7.06 (s, 3H), 6.67 (d, J = 4.2 Hz, 2H), 6.47 (br, 2H), 4.18 (br, 1H), 4.01 (br, 2H), 3.80 (br, 2H), 3.69 (m, 2H), 3.65 (m, 8H), 3.52 (m, 2H), 3.34 (s, 3H), 2.10 (s, 6H), 1.31 (m, 12H), 0.88(br, 6H). FT-IR (KBr): ν (cm^{-1}) 3434 (w), 3057 (m), 2930 (w), 2869 (s), 2200 (s) 1600 (m), 1508 (s), 1455 (m), 1314 (s), 1249 (s), 1124 (m), 834 (m). NMR and IR spectra and SEC profile of PP-*meso*-**1** are shown in Supplementary Figs. 110–114.

PP-*rac*-1 (70%, M_n = 30.7 kg/mol, M_w/M_n = 2.0, polystyrene standards). ^{1}H NMR (600 MHz, $CDCl_3$, 25 ºC): δ (ppm) 7.78 (br, 1H), 7.71 (br, 1H), 7.64 (br, 1H), 7.57 (br, 1H), 7.49 (s, 1H), 7.30 (br, 1H), 7.19 (d, J = 4.2 Hz, 2H), 7.06 (s, 3H), 6.67 (d, J = 4.2 Hz, 2H), 6.47 (br, 2H), 4.18 (br, 1H), 4.01 (br, 2H), 3.80 (br, 2H), 3.69 (m, 2H), 3.65 (m, 8H), 3.52 (m, 2H), 3.34 (s, 3H), 2.10 (s, 6H), 1.31 (m, 12H), 0.88(br, 6H). FT-IR (KBr): ν (cm^{-1}) 3470 (w), 3055 (m), 2928 (w), 2869 (s), 2200 (s) 1600 (m), 1508 (s), 14554(m), 1314 (s), 1248 (s), 1127 (m), 1032 (s), 834 (m), 741 (s), 700 (s). NMR and IR spectra and SEC profile of PP-*rac*-**1** are shown in Supplementary Figs. 115–119.

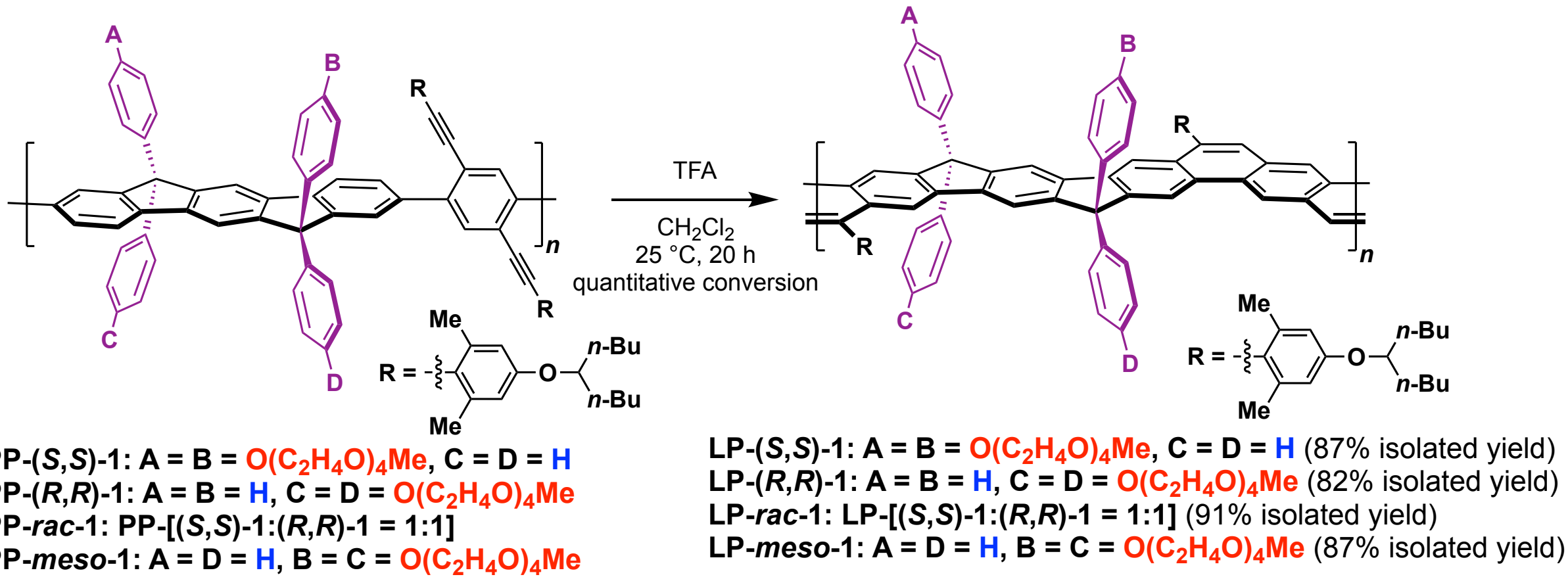

LP-(*R*,*R*)-1 and LP-(*S*,*S*)-1, LP-*meso*-1, LP-*rac*-1. A CH_2Cl_2 solution (3 mL) of (*S*,*S*)-PP-**1** (20 mg, 12.3 μmol) and TFA (200 μL) was stirred at 25 °C for 20 h. The reaction mixture was diluted with CH_2Cl_2, washed with saturated aqueous $NaHCO_3$ and water, dried over anhydrous $MgSO_4$ and concentrated under reduced pressure to afford (*S*,*S*)-LP-**1** as a brown solid (87%, 17.4 mg, 10.7 μmol; M_n = 25.1 kg mol^{-1}, $Đ$ = 3.1, polystyrene standards). The other stereochemical variants were obtained analogously.

LP-(*S*,*S*)-1 (87%, M_n = 25.1 kg/mol, M_w/M_n = 3.1, polystyrene standards). ^{1}H NMR (600 MHz, $CDCl_3$, 25 ºC): δ (ppm) 8.90 (s, 1H), 8.76 (s, 1H), 7.69 (d, J = 9.6 Hz, 3H), 7.38 (br, 4H), 7.28 (Br, 10H), 6.79 (m, 8H), 4.37 (br, 2H), 4.07 (br, 4H), 3.81 (br, 4H), 3.68 (br, 16H), 3.49 (br, 4H), 3.32 (s, 6H), 2.04 (br, 6H), 2.00 (br, 6H), 1.78 (m, 8H), 1.45 (m, 16H), 0.99 (br, 12H). FT-IR (KBr): v (cm^{-1}) 3414 (w), 3056 (w), 2929 (m), 2868 (w), 1603 (m), 1508 (m), 1485 (m), 1309 (s), 1257 (s), 1106 (br), 1040 (br), 867 (m), 803 (br), 745 (s), 700 (s). NMR and IR spectra and SEC profile of LP-(*S*,*S*)-**1** are shown in Supplementary Figs. 120–124.

LP-(*R*,*R*)-1 (82%, M_n = 27.9 kg/mol, M_w/M_n = 2.3, polystyrene standards). ^{1}H NMR (600 MHz, $CDCl_3$, 25 ºC): δ (ppm) 8.90 (s, 1H), 8.76 (s, 1H), 7.68 (d, J = 9.6 Hz, 3H), 7.38 (br, 4H), 7.28 (Br, 10H), 6.79 (m, 8H), 4.37 (br, 2H), 4.07 (br, 4H), 3.81 (br, 4H), 3.68 (br, 16H), 3.49 (br, 4H), 3.32 (s, 6H), 2.04 (br, 6H), 2.00 (br, 6H), 1.78 (m, 8H), 1.45 (m, 16H), 0.99 (br, 12H). FT-IR (KBr): v (cm^{-1}) 3430 (w), 3046 (w), 2925 (m), 2871 (w), 1603 (m), 1507 (m), 1479 (m), 1309 (s), 1250 (s), 1108 (br), 1043 (br), 867 (m), 808 (br), 746 (s), 700 (s). NMR and IR spectra and SEC profile of LP-(*R*,*R*)-**1** are shown in Supplementary Figs. 125–129.

LP-*meso*-1 (87%, M_n = 29.7 kg/mol, M_w/M_n = 2.3, polystyrene standards). ^{1}H NMR (600 MHz, $CDCl_3$, 25 ºC): δ (ppm) 8.90 (s, 1H), 8.76 (s, 1H), 7.69 (d, J = 9.6 Hz, 3H), 7.38 (br, 4H), 7.28 (Br, 10H), 6.79 (m, 8H), 4.37 (br, 2H), 4.07 (br, 4H), 3.81 (br, 4H), 3.68 (br, 16H), 3.49 (br, 4H), 3.32 (s, 6H), 2.04 (br, 6H), 2.00 (br, 6H), 1.78 (m, 8H), 1.45 (m, 16H), 0.99 (br, 12H). FT-IR (KBr): v (cm^{-1}) 3430 (w), 3056 (w), 2929 (m), 2868 (w), 1604 (m), 1506 (m), 1459 (m), 1308 (s), 1250 (s), 1111 (br), 1041 (br), 867 (m), 807 (br), 700 (s). NMR and IR spectra and SEC profiles of LP-*meso*-**1** are shown in Supplementary Figs. 130–134.

LP-*rac*-1 (91%, M_n = 30.1 kg/mol, M_w/M_n = 2.2, polystyrene standards). ^{1}H NMR (600 MHz, $CDCl_3$, 25 ºC): δ (ppm) 8.90 (s, 1H), 8.76 (s, 1H), 7.69 (d, J = 9.6 Hz, 3H), 7.38 (br, 4H), 7.28

(Br, 10H), 6.79 (m, 8H), 4.37 (br, 2H), 4.07 (br, 4H), 3.81 (br, 4H), 3.68 (br, 16H), 3.49 (br, 4H), 3.32 (s, 6H), 2.04 (br, 6H), 2.00 (br, 6H), 1.78 (m, 8H), 1.45 (m, 16H), 0.99 (br, 12H). FT-IR (KBr): *ν* (cm^{-1}) 3450 (w), 3056 (w), 2925 (m), 2855 (w), 2359 (m), 1604 (m), 1507 (m), 1456 (m), 1310 (s), 1259 (s), 1111 (w), 1045 (w), 867 (m), 807 (m), 748 (s), 700 (s). NMR and IR spectra and SEC profile of LP-*rac*-**1** are shown in Supplementary Figs. 135–139.

5. Supplementary Figure

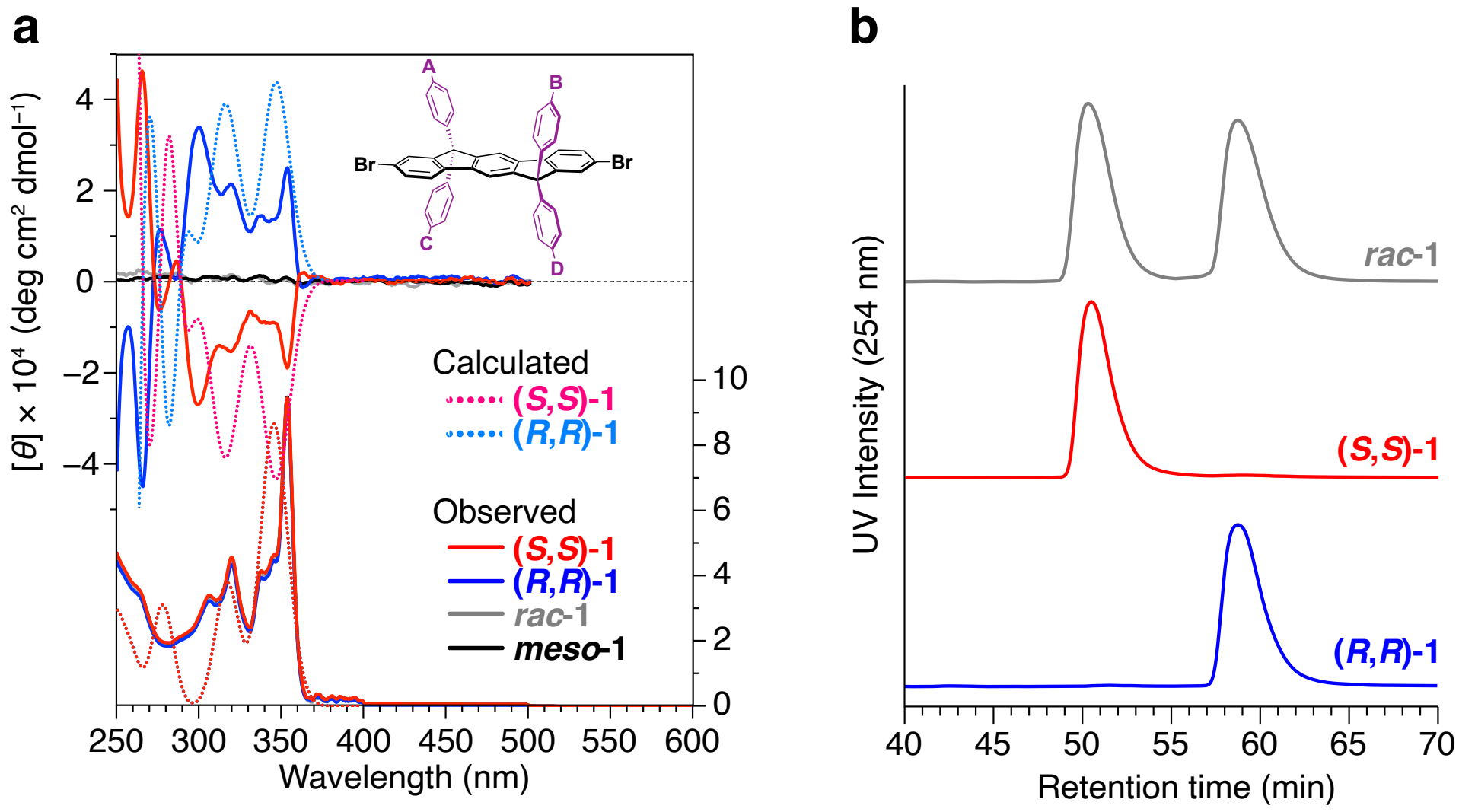

Supplementary Fig. 1 | Synthesis of enantiopure bifacial monomers. a, Experimentally observed (solid lines) and theoretically calculated (dotted lines) UV-vis/CD spectra (25 °C, 15 µM, THF) of (*S*,*S*)-**1**, (*R*,*R*)-**1**, *rac*-**1** and *meso*-**1**. **b,** Chiral HPLC charts (Daicel CHIRALPAK IE-3, *n*-hexane:EtOAc = 65:35) of *rac*-**1**, (*S*,*S*)-**1** and (*R*,*R*)-**1**.

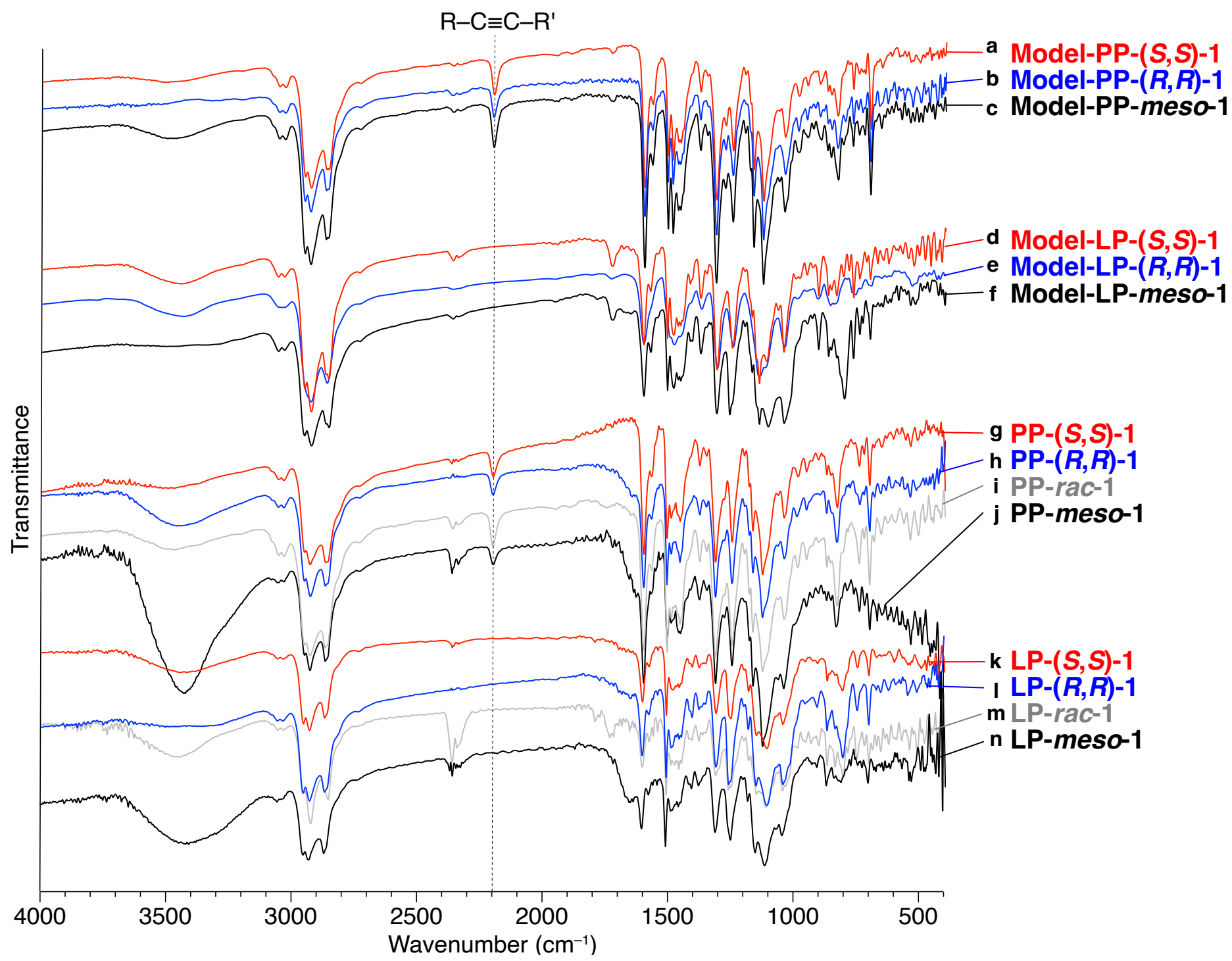

Supplementary Fig. 2 | Structural characterization of bifacial ladder polymers. a–n, FT-IR spectra (KBr, 25 °C) of Model-PP-(*S*,*S*)-**1** (a), Model-PP-(*R*,*R*)-**1** (b), Model-PP-*meso*-**1** (c), Model-LP-(*S*,*S*)-**1** (d), Model-LP-(*R*,*R*)-**1** (e), Model-LP-*meso*-**1** (f), PP-(*S*,*S*)-**1** (g), PP-(*R*,*R*)-**1** (h), PP-*meso*-**1** (i), PP-*rac*-**1** (j), LP-(*S*,*S*)-**1** (k), LP-(*R*,*R*)-**1** (l), LP-*meso*-**1** (m) and LP-*rac*-**1** (n).

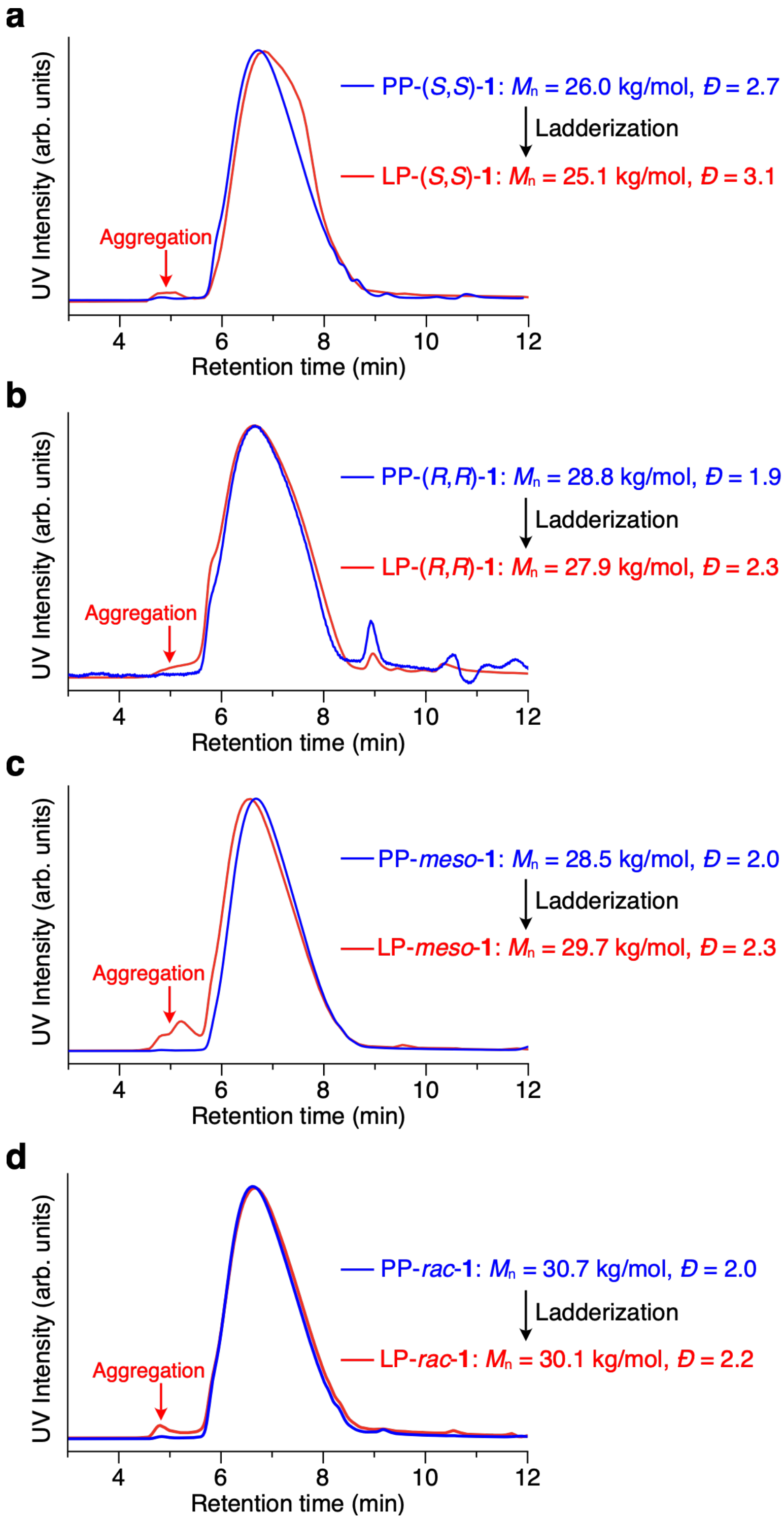

Supplementary Fig. 3 | Size-exclusion chromatography (SEC) traces of precursor and ladder polymers. a–d, SEC profiles (eluent; $CHCl_3$) of PP-(*S*,*S*)-**1** (a), PP-(*R*,*R*)-**1** (b), PP-*meso*-**1** (c) and PP-rac-**1** (d) (blue lines) and their corresponding ladder polymers LP-(*S*,*S*)-**1** (a), LP-(*R*,*R*)-**1** (b), LP-*meso*-**1** (c) and LP-*rac*-**1** (d) (red lines). Minor early-eluting features correspond to aggregation, which are more prominently observed in the ladder polymers. The slightly higher apparent dispersity values of the ladder polymers might be attributed to non-ideal SEC behavior associated with aggregation and stronger interactions with the SEC column due to increased backbone planarity upon ladderization.

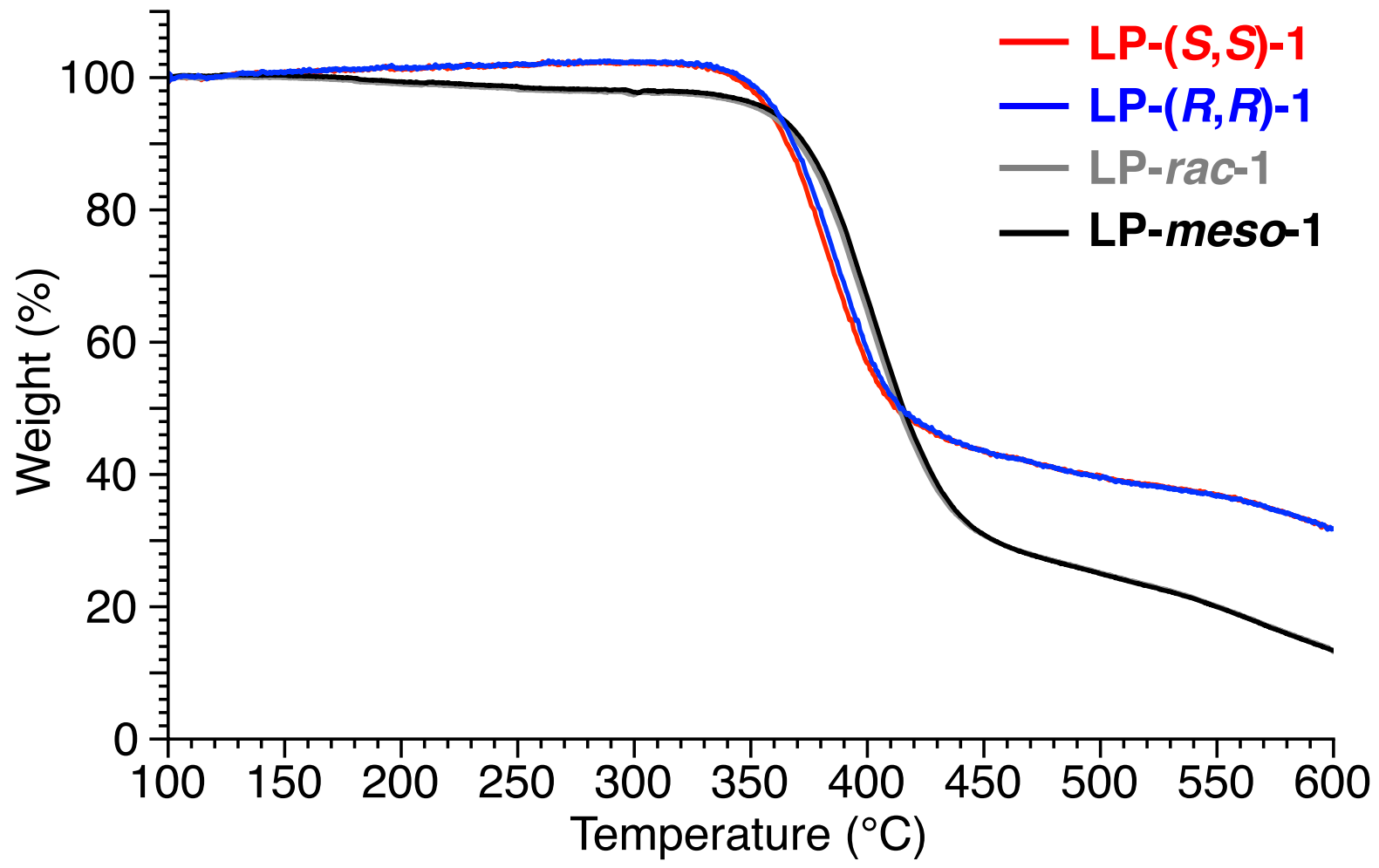

Supplementary Fig. 4 | TGA profiles of LP-(*S*,*S*)-**1** (red), LP-(*R*,*R*)-**1** (blue), LP-*rac*-**1** (gray) and LP-*meso*-**1** (black) measured at a scan rate of 10 °C min^{-1} under N_2 flow (20 mL min^{-1}).

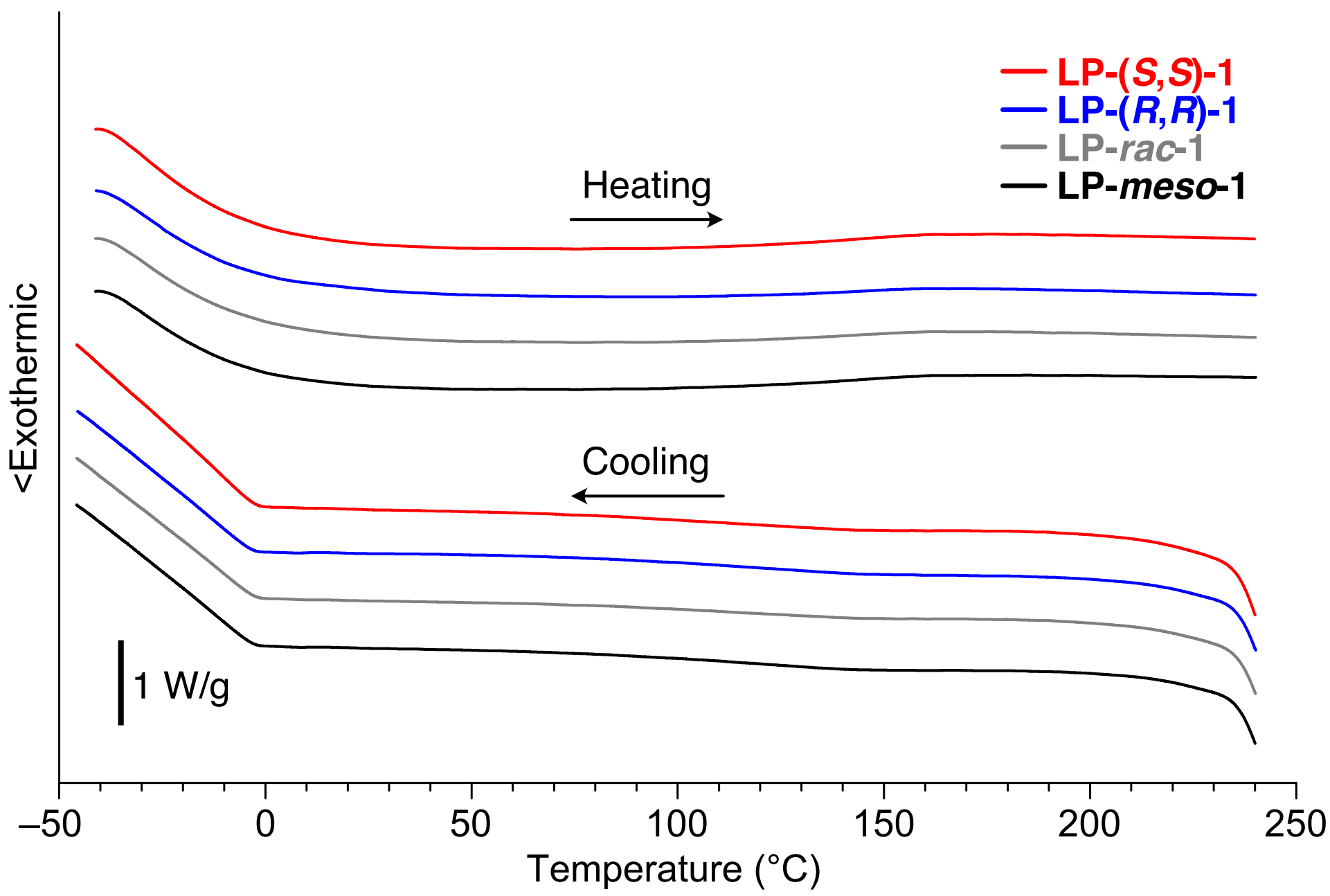

Supplementary Fig. 5 | DSC profiles (2nd cycle) of LP-(*S*,*S*)-**1** (red), LP-(*R*,*R*)-**1** (blue), LP-*rac*-**1** (gray) and LP-*meso*-**1** (black) measured at a scan rate of 10 °C min^{-1} under N_2 flow (20 mL min^{-1}).

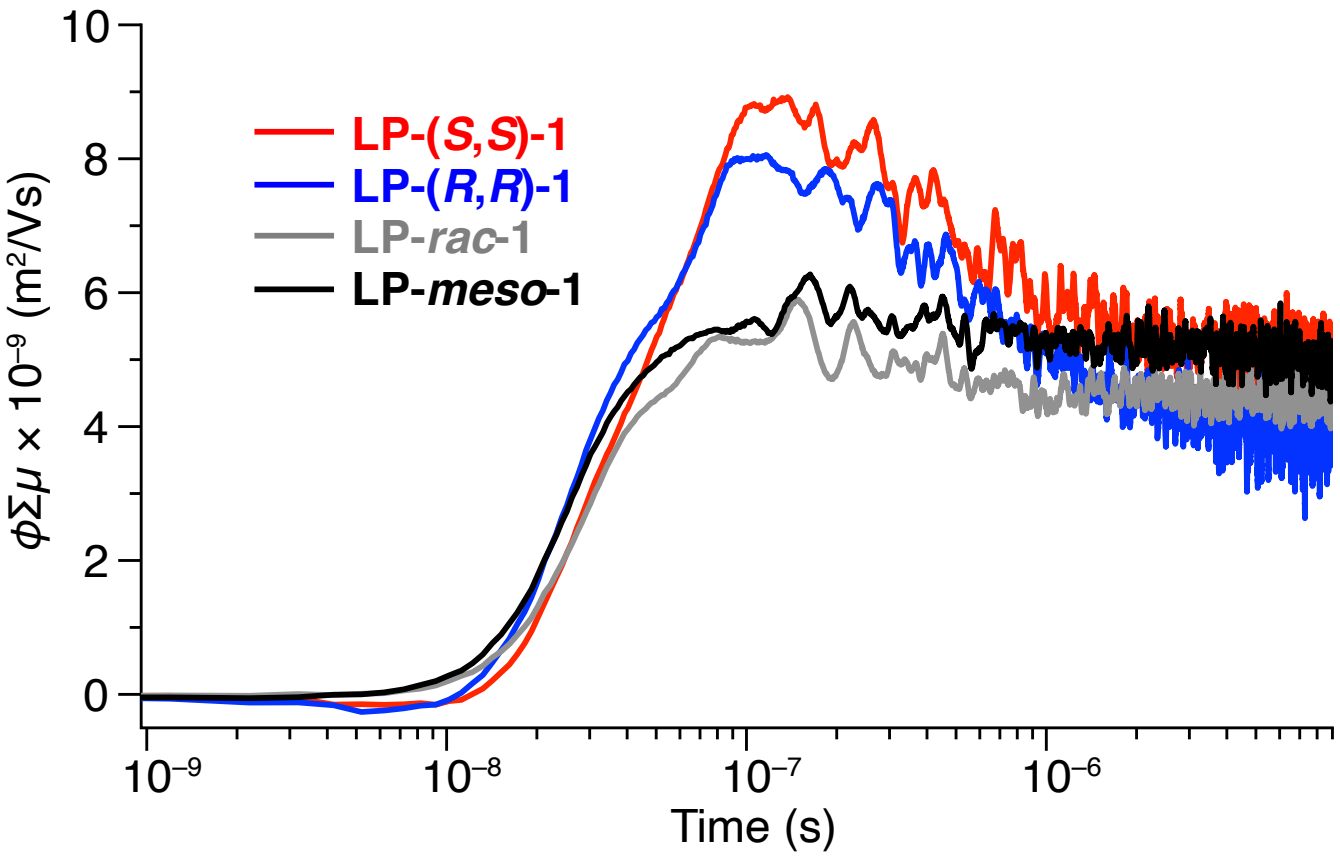

Supplementary Fig. 6 | TRMC profiles of thin films of LP-(*S*,*S*)-**1** (red), LP-(*R*,*R*)-**1** (blue), LP-*rac*-**1** (gray) and LP-*meso*-**1** (black) on quartz.

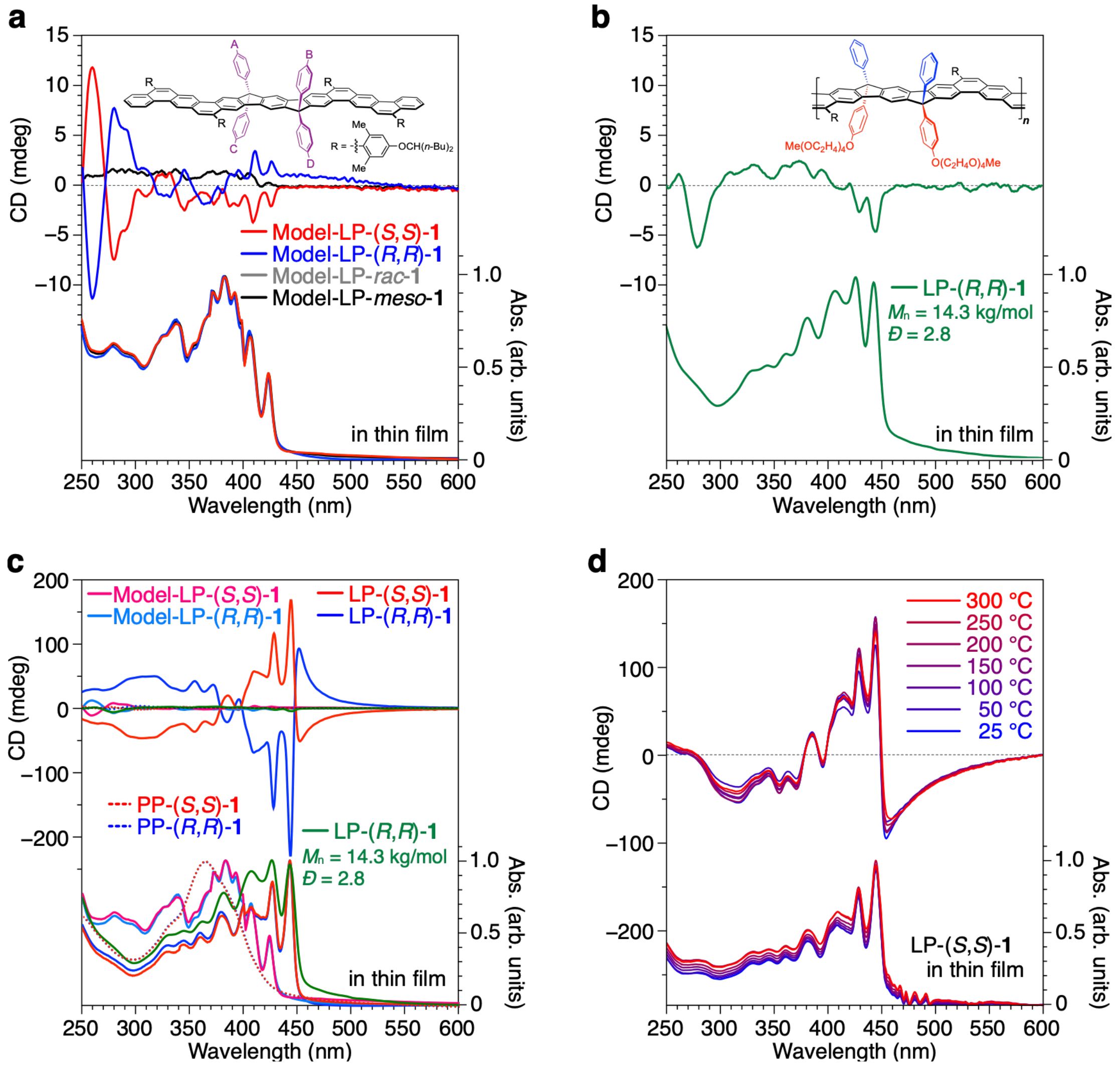

Supplementary Fig. 7 | UV-vis/CD spectra in the thin-film state. a,b, UV-vis/CD spectra in thin-film states of Model-LP-**1**s (a), LP-(*R*,*R*)-**1** with M_n = 14.3 kDa (b). **c,** Comparison of thin-film UV-vis/CD spectra of Model-LP-**1**s, PP-**1**s and LP-**1**s. **d,** UV-vis/CD spectra of an LP-(*S*,*S*)-**1** thin film after thermal annealing at 50–300 °C for 60 s in air.

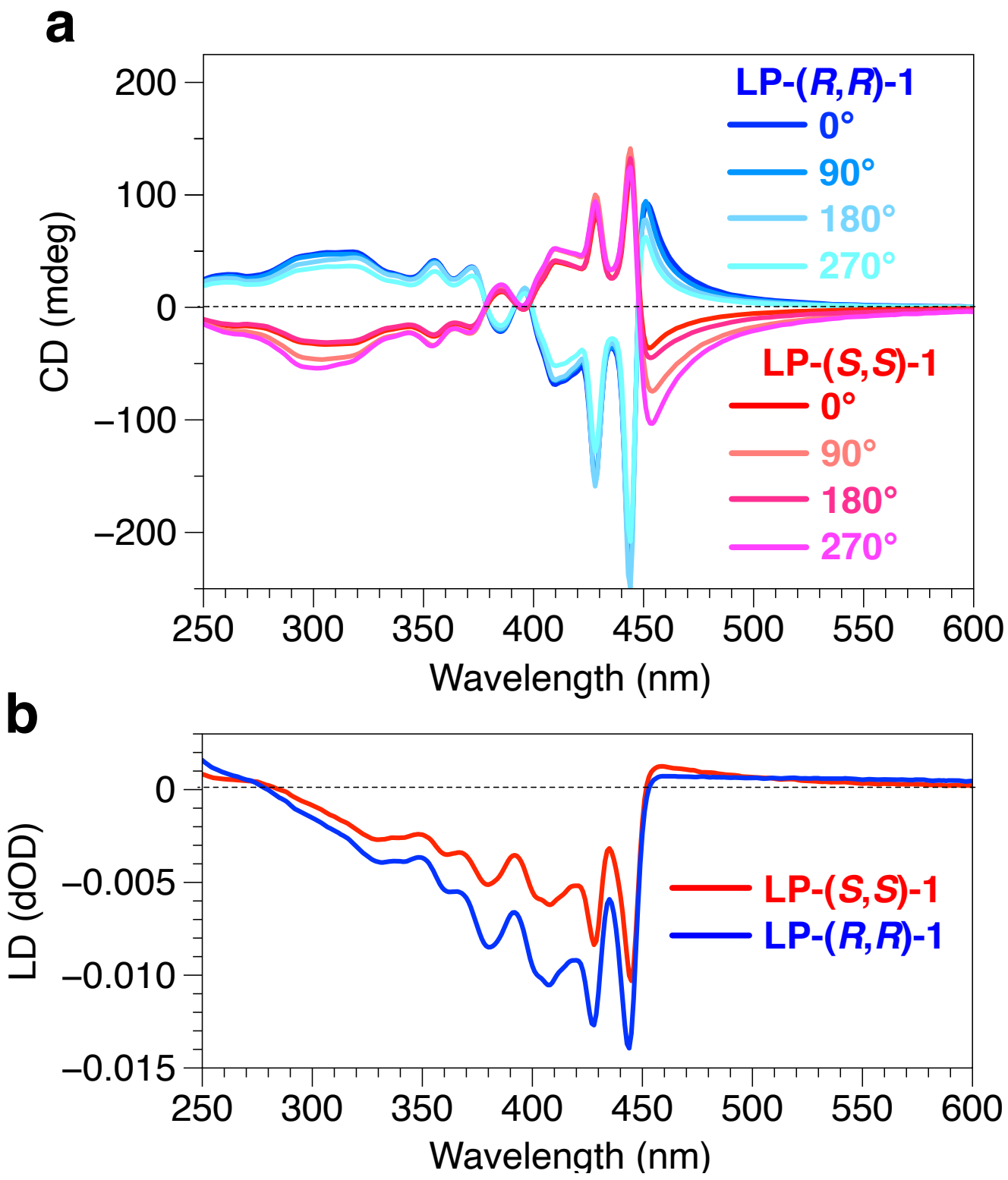

Supplementary Fig. 8 | Angle-dependent CD (a) and LD (b) spectra of thin films of LP-(*S*,*S*)-**1** and LP-(*R*,*R*)-**1** on quartz.

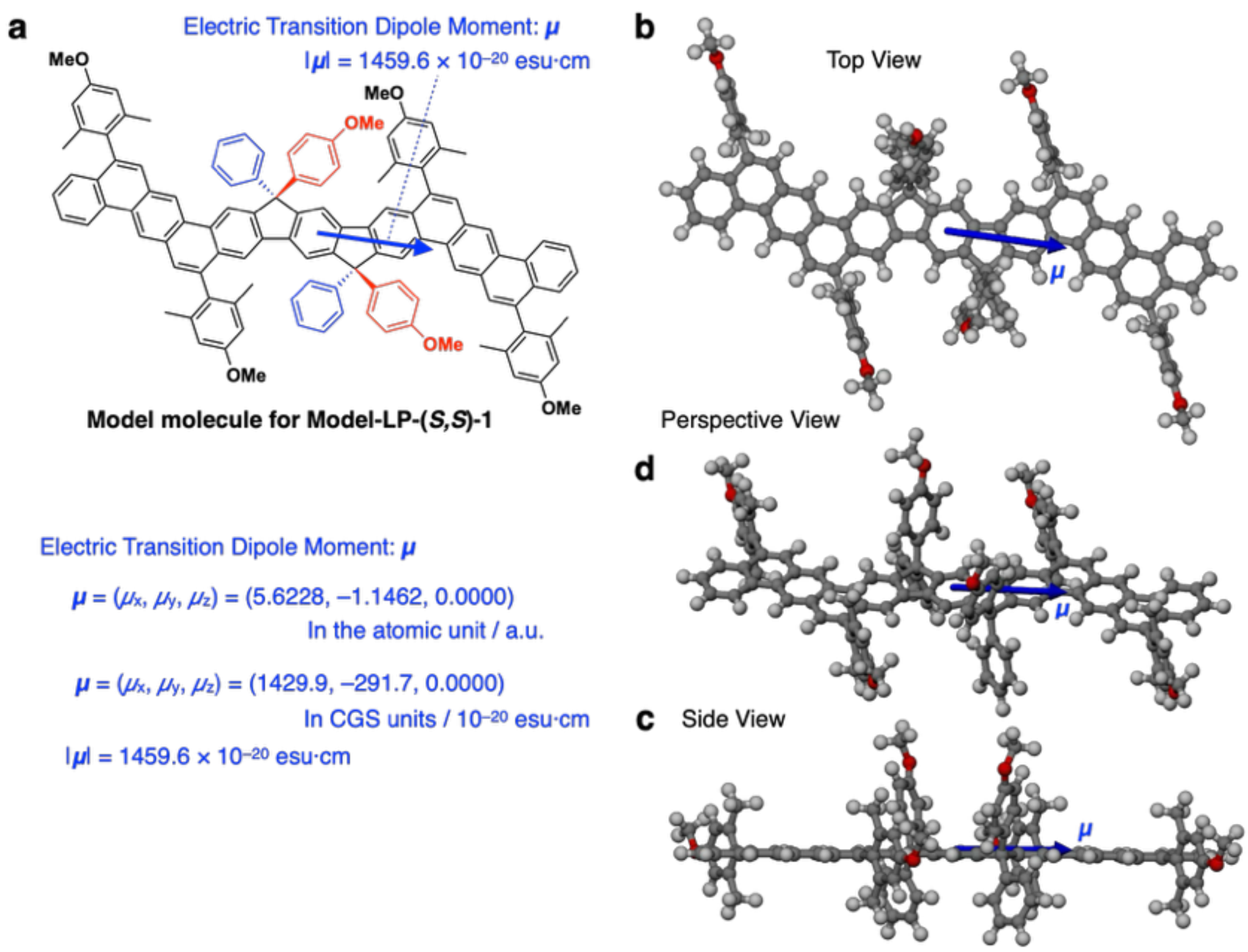

Supplementary Fig. 9 | (a) Simplified calculated model of Model-LP-(*S*,*S*)-**1** used for TD-DFT calculations. Direction of the corresponding electric transition dipole moment (***μ***) in (b) top, (c) perspective and (d) side views. Conversion of ***μ*** from a.u. to CGS followed ref. 4.

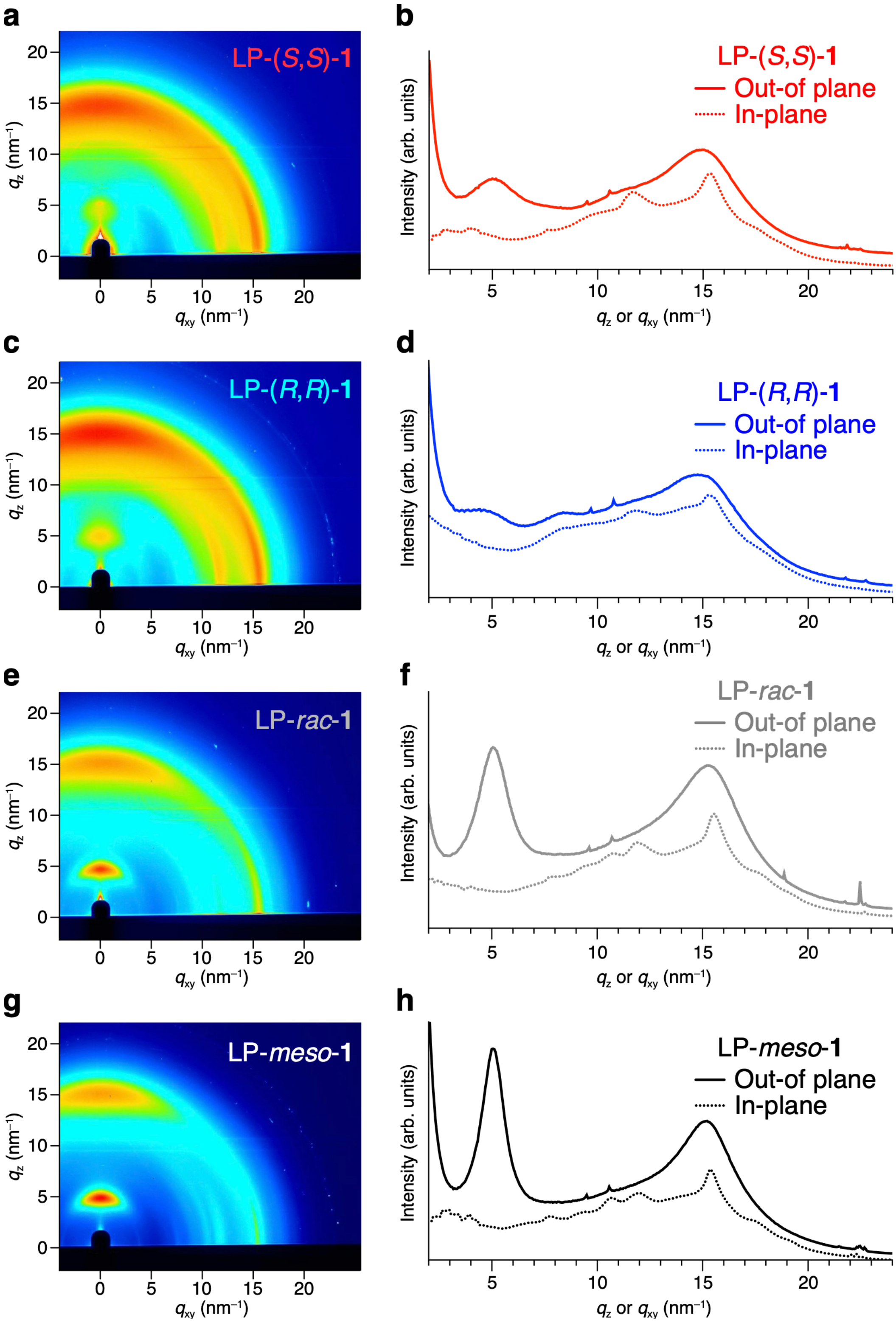

Supplementary Fig. 10 | 2D GI-XRD images (a,c,e,g) and in-plane and out-of-plane profiles (b,d,f,h) of thin films of LP-(*S*,*S*)-**1** (a,b), LP-(*R*,*R*)-**1** (c,d), LP-*rac*-**1** (e,f) and LP-*meso*-**1** (g,h) on quartz.

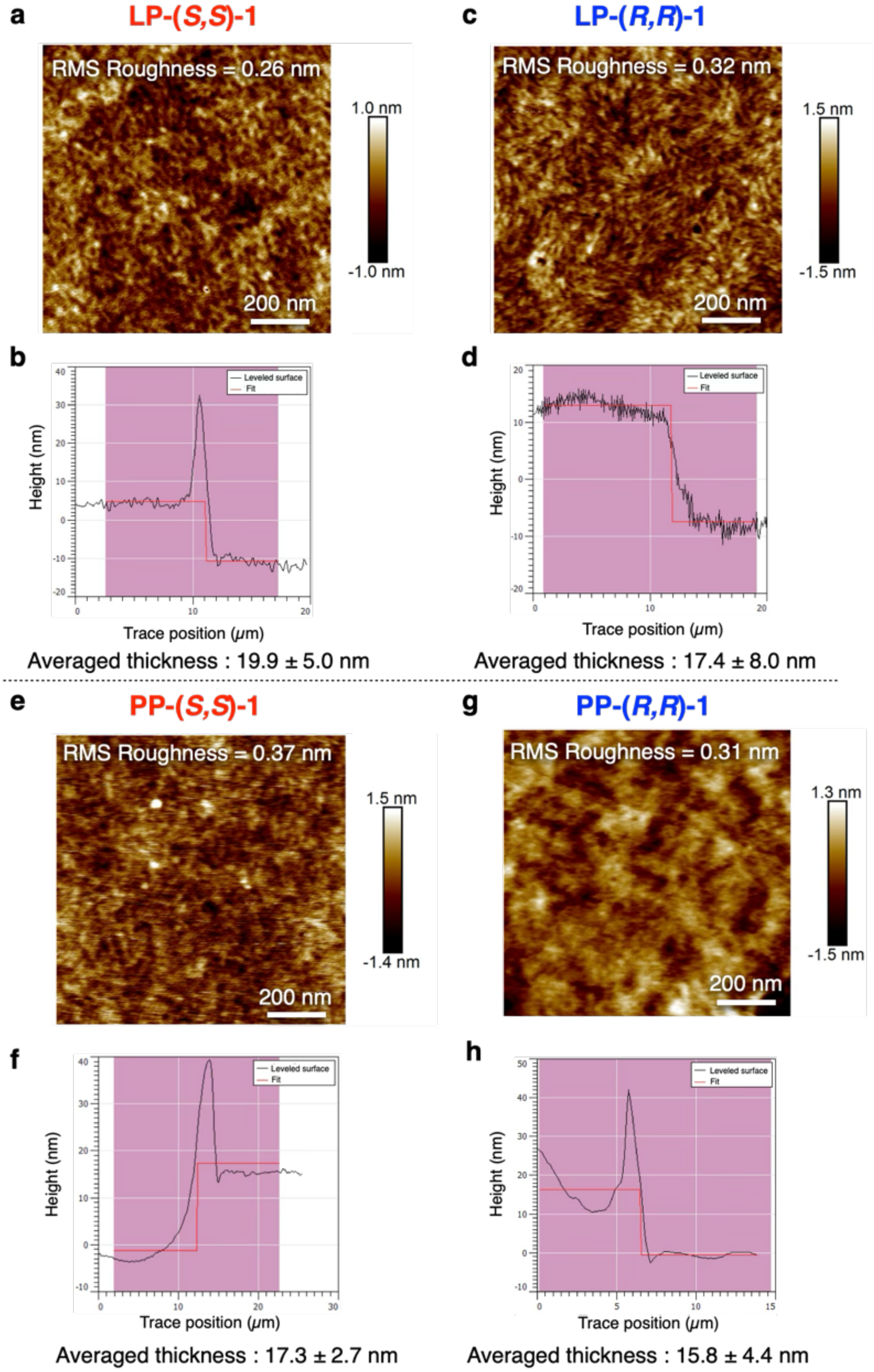

Supplementary Fig. 11 | AFM images (a,c,e,g) and representative film thickness measurements (b,d,f,h) of thin film of LP-(*S*,*S*)-**1** (a,b) and LP-(*R*,*R*)-**1** (c,d), PP-(*S*,*S*)-**1** (e,f) and PP-(*R*,*R*)-**1** (g,h) on HOPG.

6. Analytical Data.

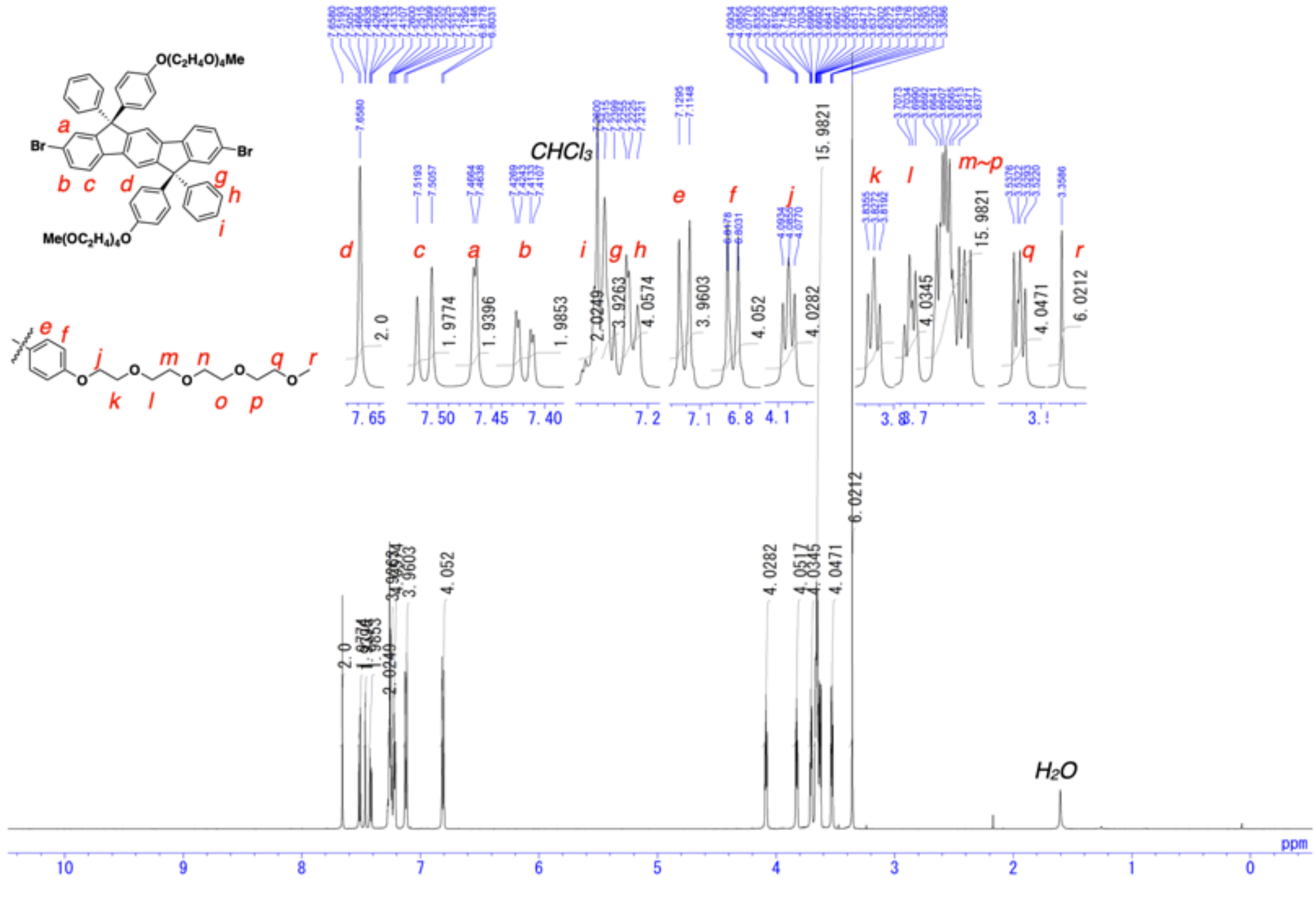

Supplementary Fig. 12 | ^1H NMR spectrum of (*S,S*)-**1** (600 MHz, $CDCl_3$, 25 ºC).

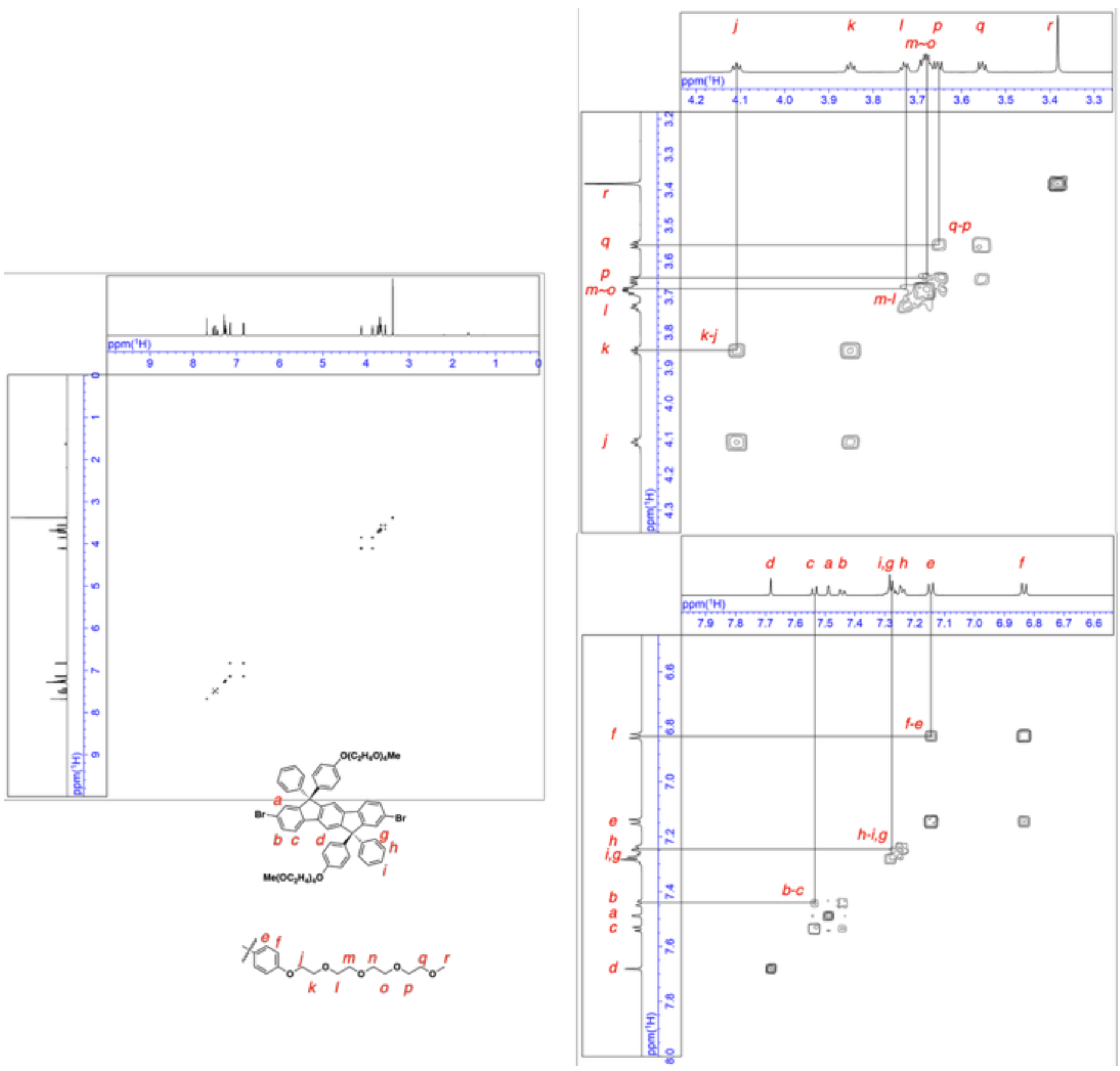

Supplementary Fig. 13 | HH-COSY correlation of (*S,S*)-**1** (600 MHz, $CDCl_3$, 25 ºC).

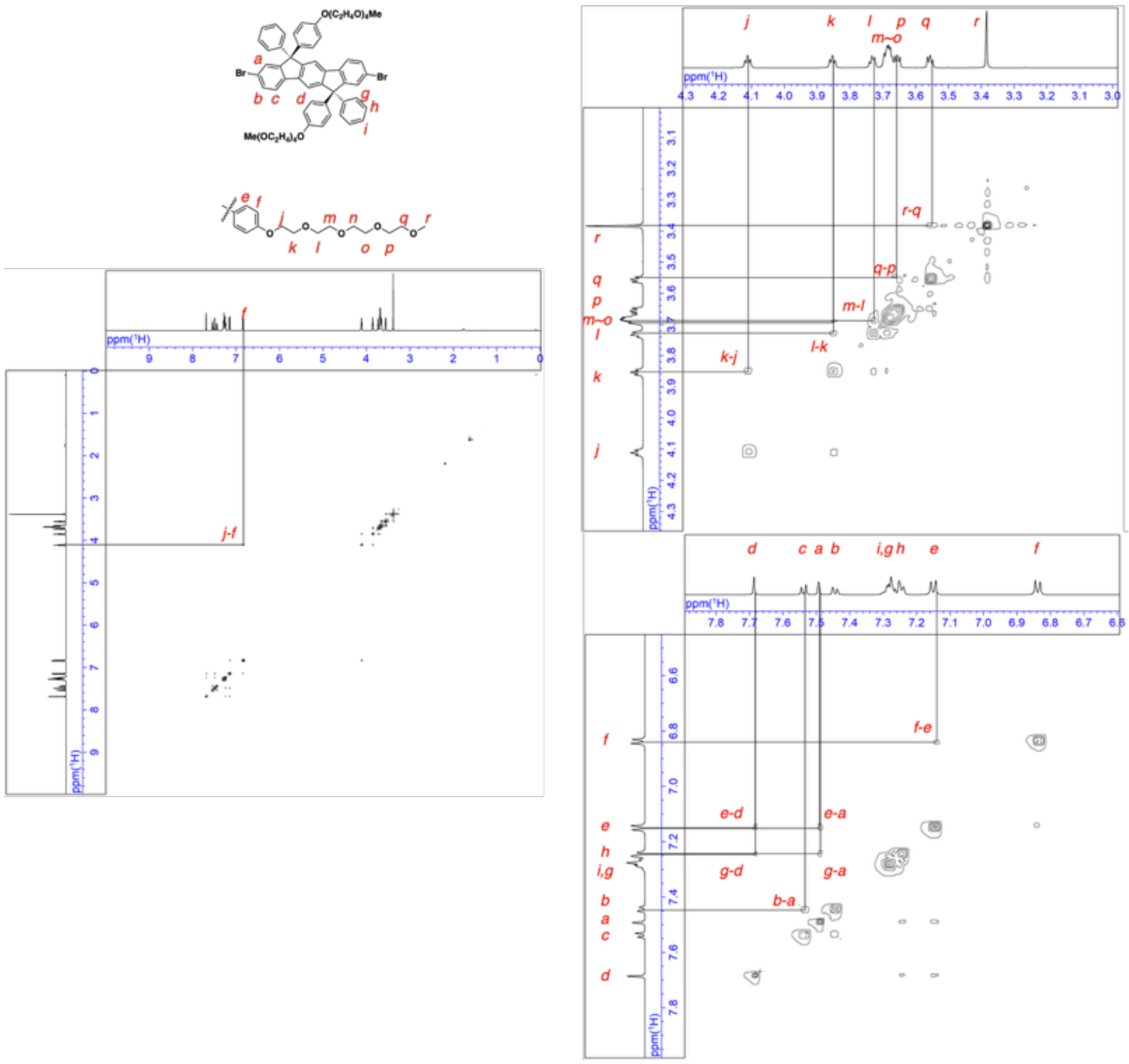

Supplementary Fig. 14 | NOESY correlation of (*S*,*S*)-**1** (600 MHz, $CDCl_3$, 25 ºC).

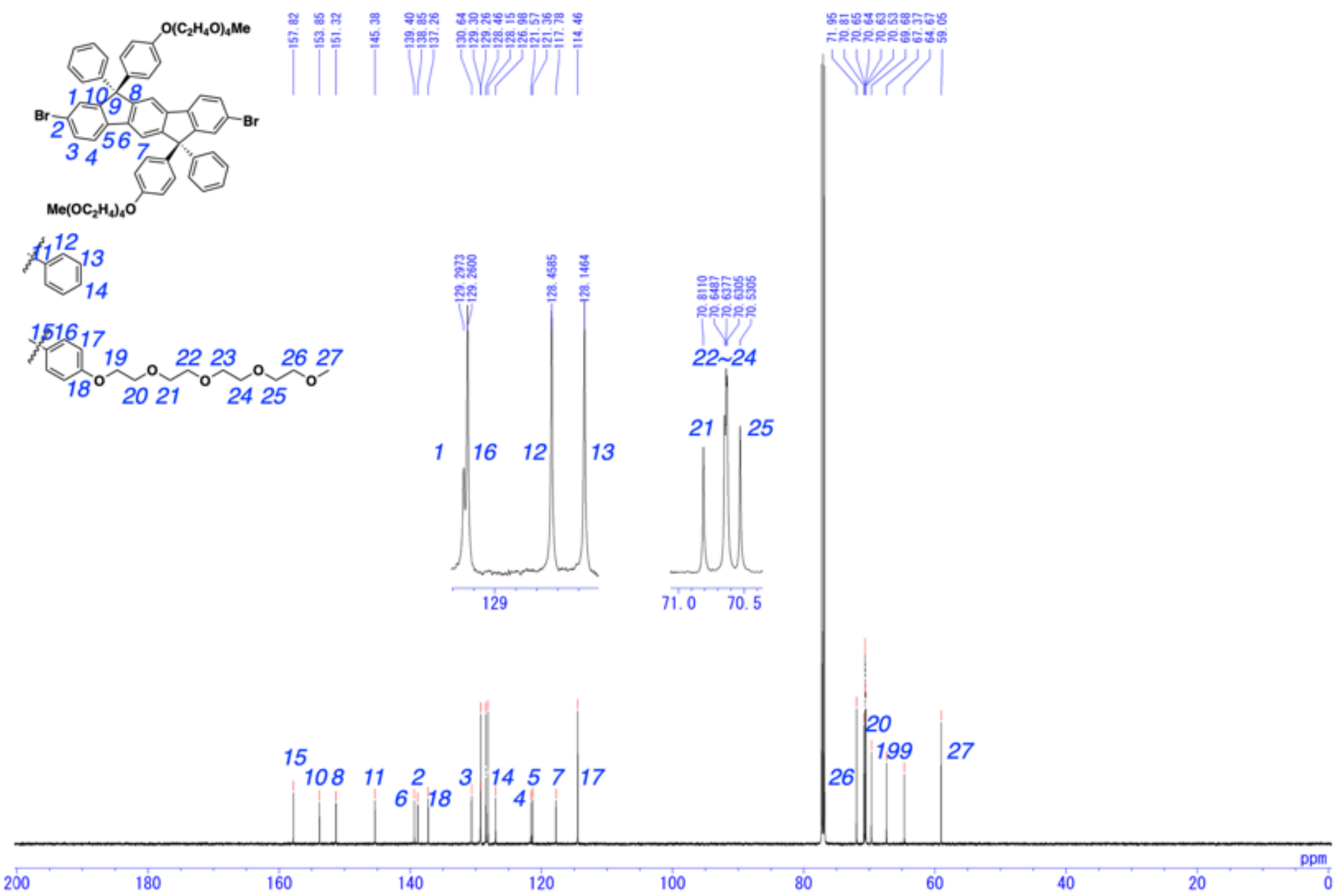

Supplementary Fig. 15 | ^{13}C NMR spectrum of (*S*,*S*)-**1** (151 MHz, $CDCl_3$, 25 ºC).

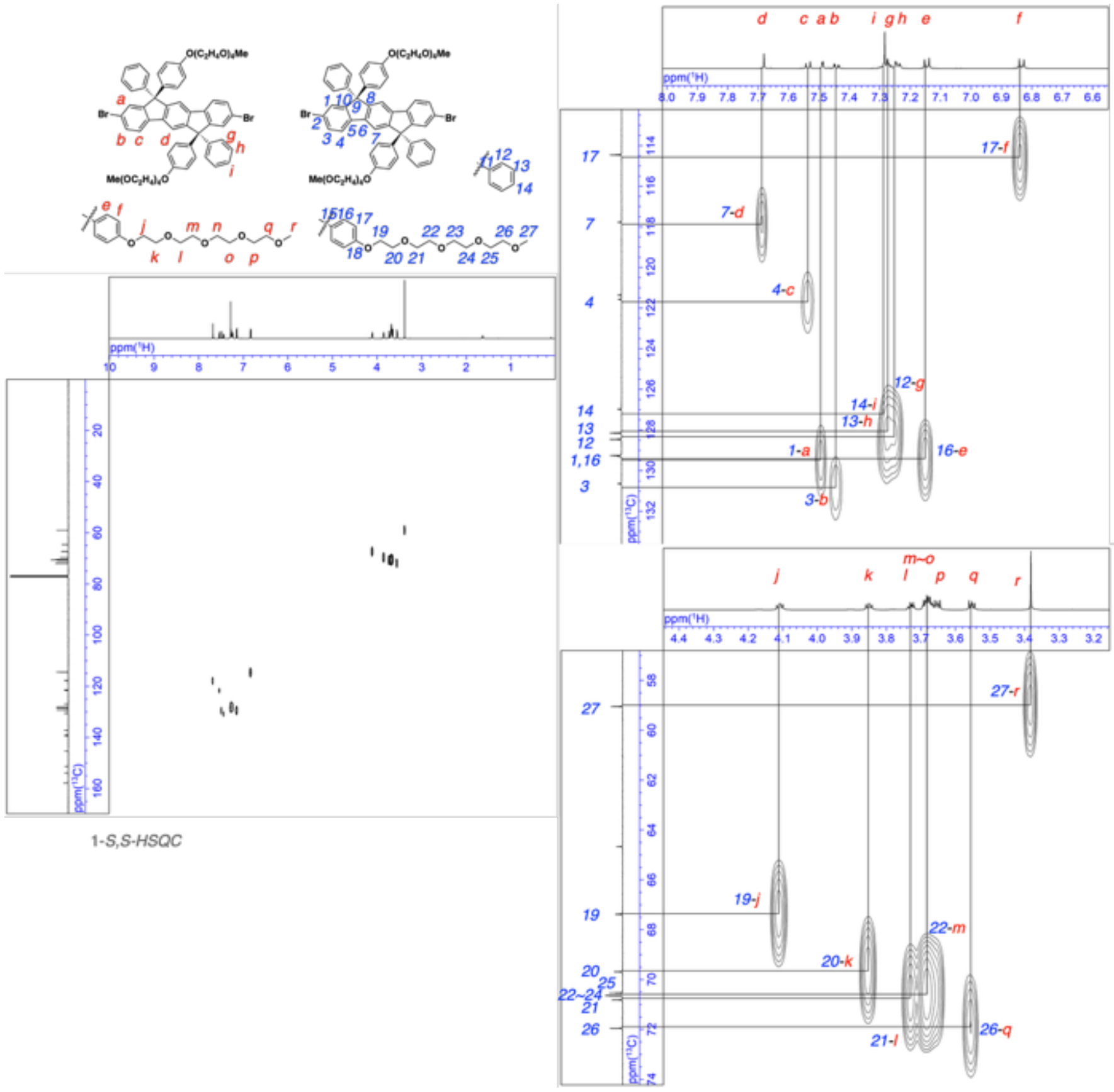

Supplementary Fig. 16 | HSQC correlation of (*S,S*)-**1** (600 MHz for ^{1}H and 151 MHz for ^{13}C, $CDCl_3$, 25 ºC).

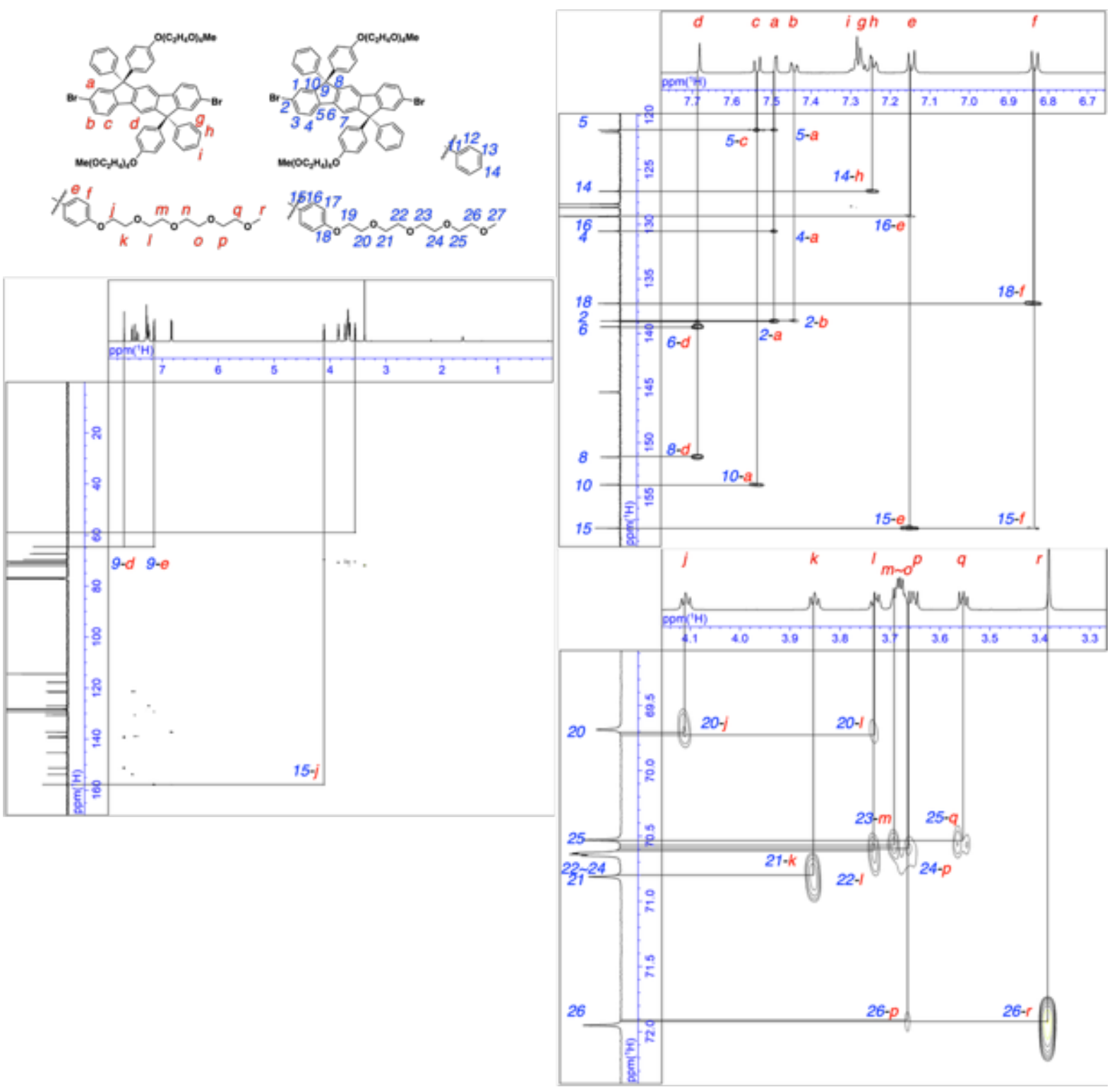

Supplementary Fig. 17 | HMBC correlation of (*S,S*)-**1** (600 MHz for ^{1}H and 151 MHz for ^{13}C, $CDCl_3$, 25 ºC).

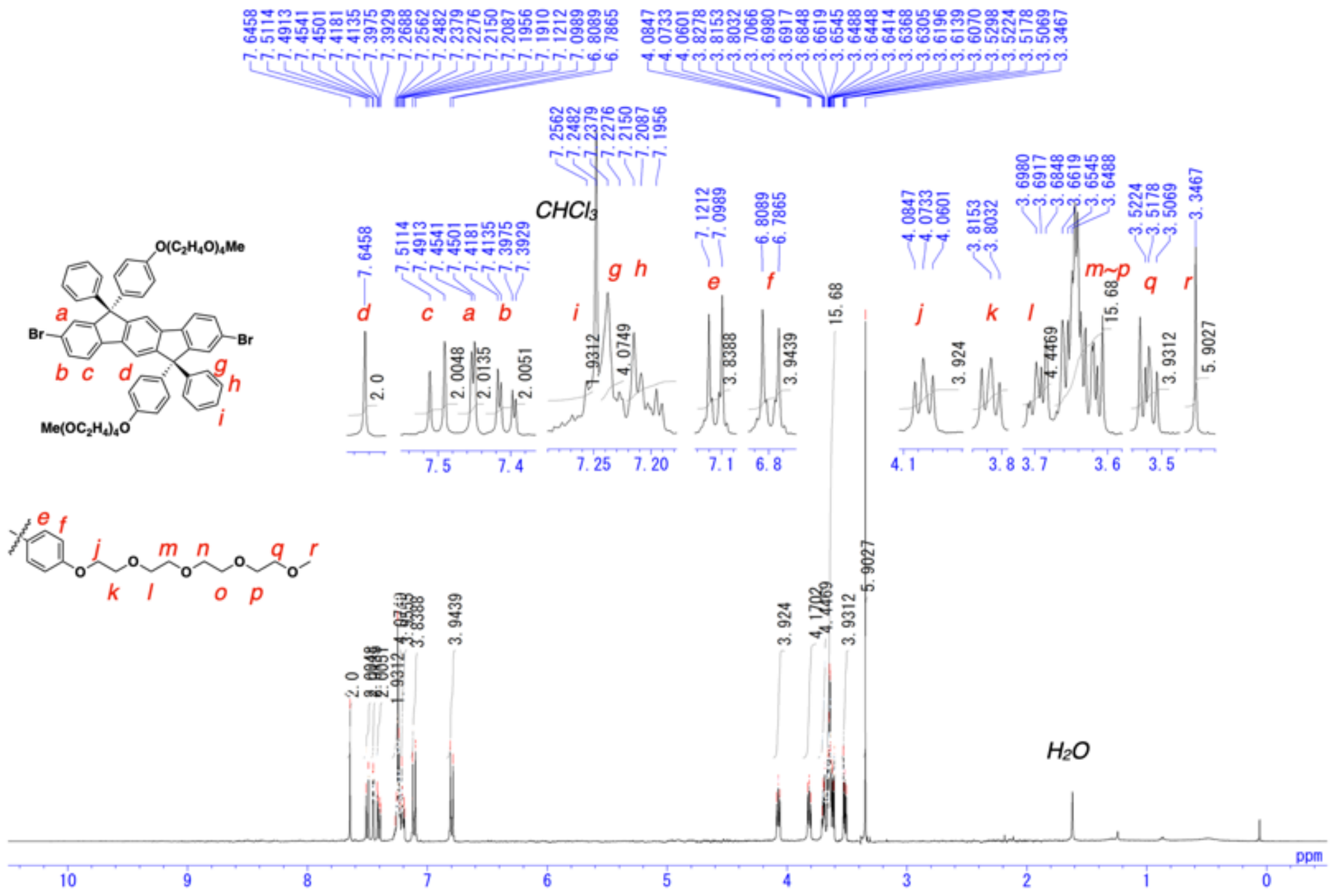

Supplementary Fig. 18 | ^{1}H NMR spectrum of (*R,R*)-**1** (600 MHz, $CDCl_3$, 25 ºC).

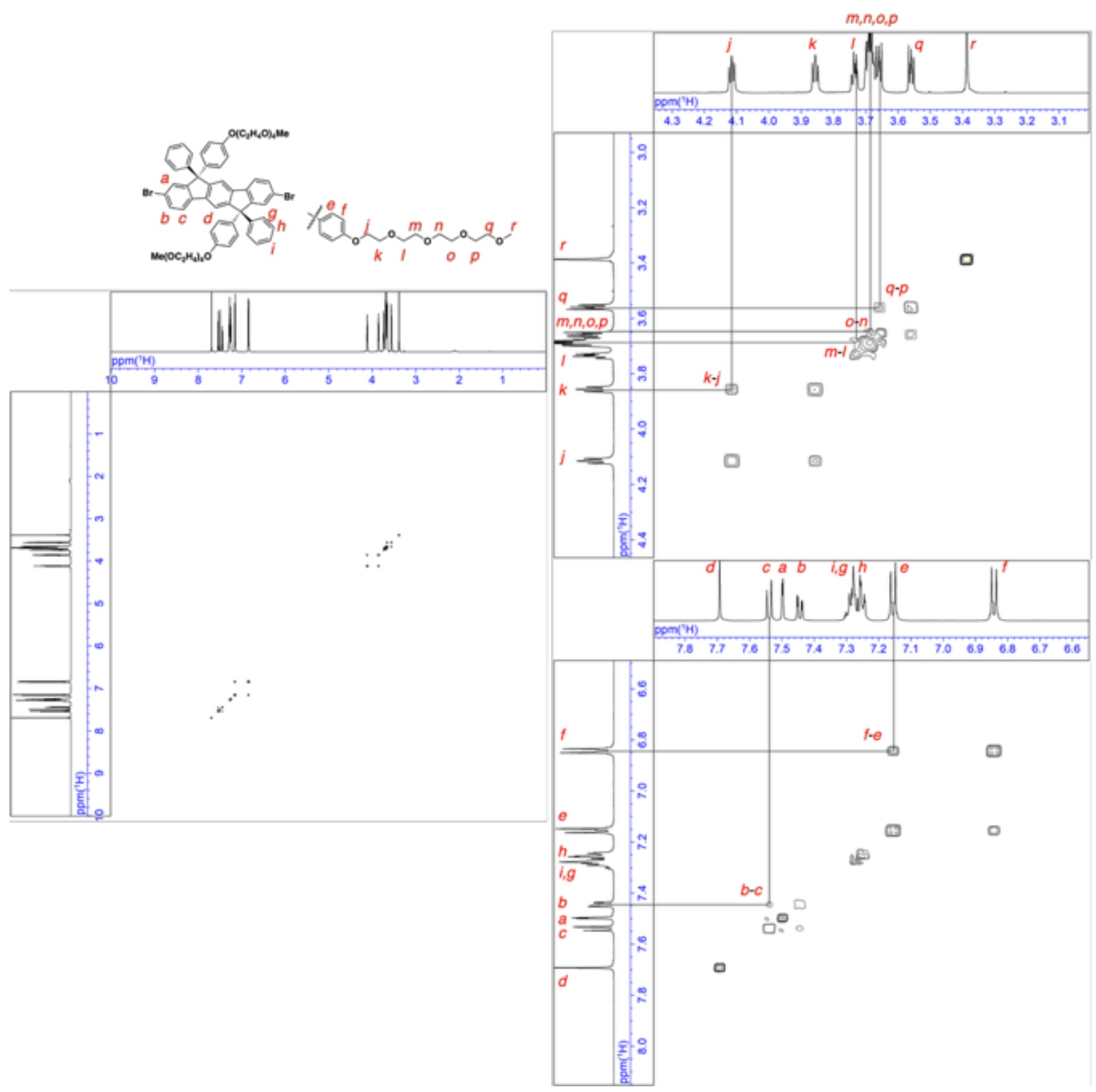

Supplementary Fig. 19 | HH-COSY correlation of (*R,R*)-**1** (600 MHz, $CDCl_3$, 25 ºC).

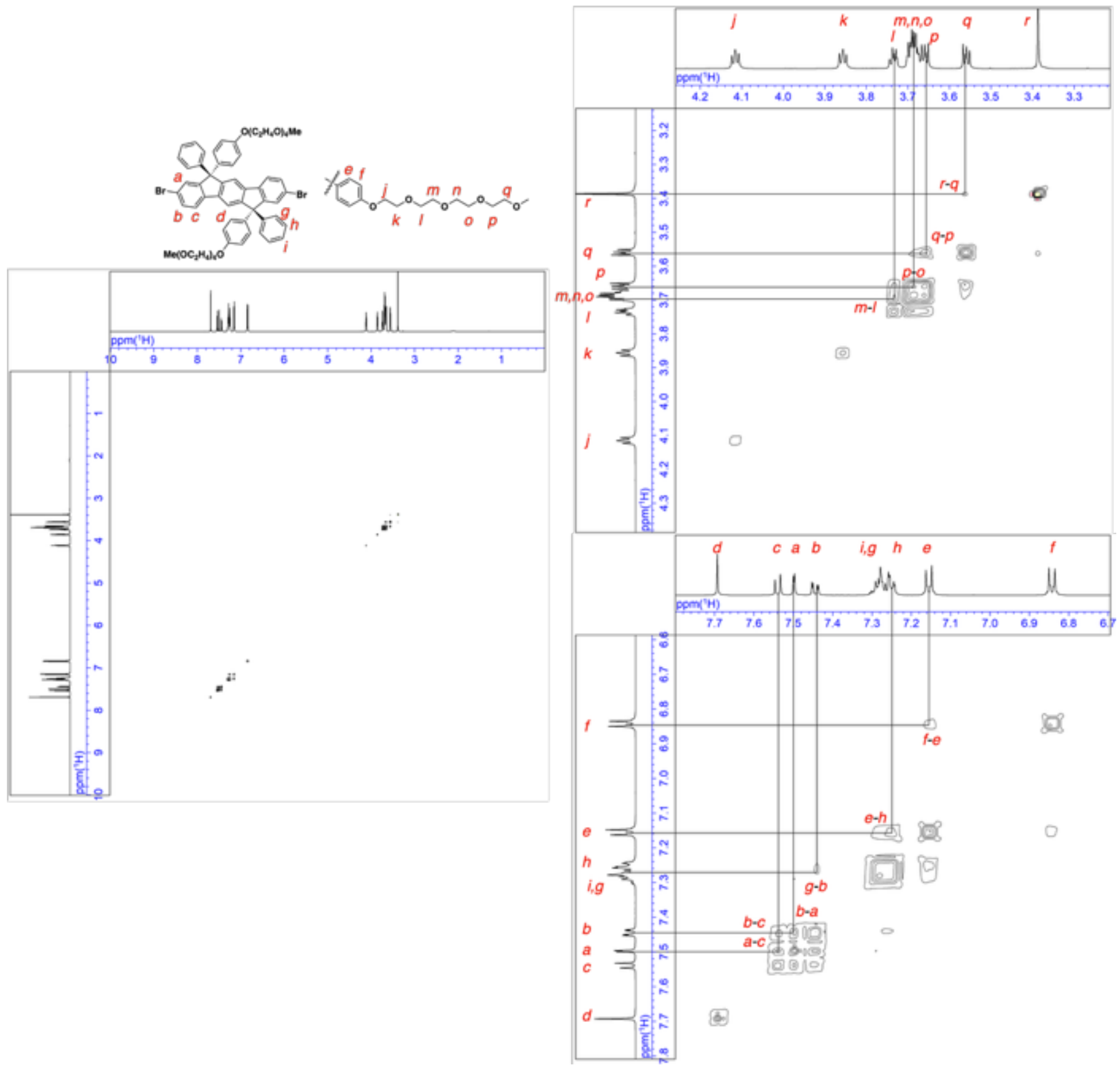

Supplementary Fig. 20 | NOESY correlation of (*R,R*)-**1** (600 MHz, $CDCl_3$, 25 ºC).

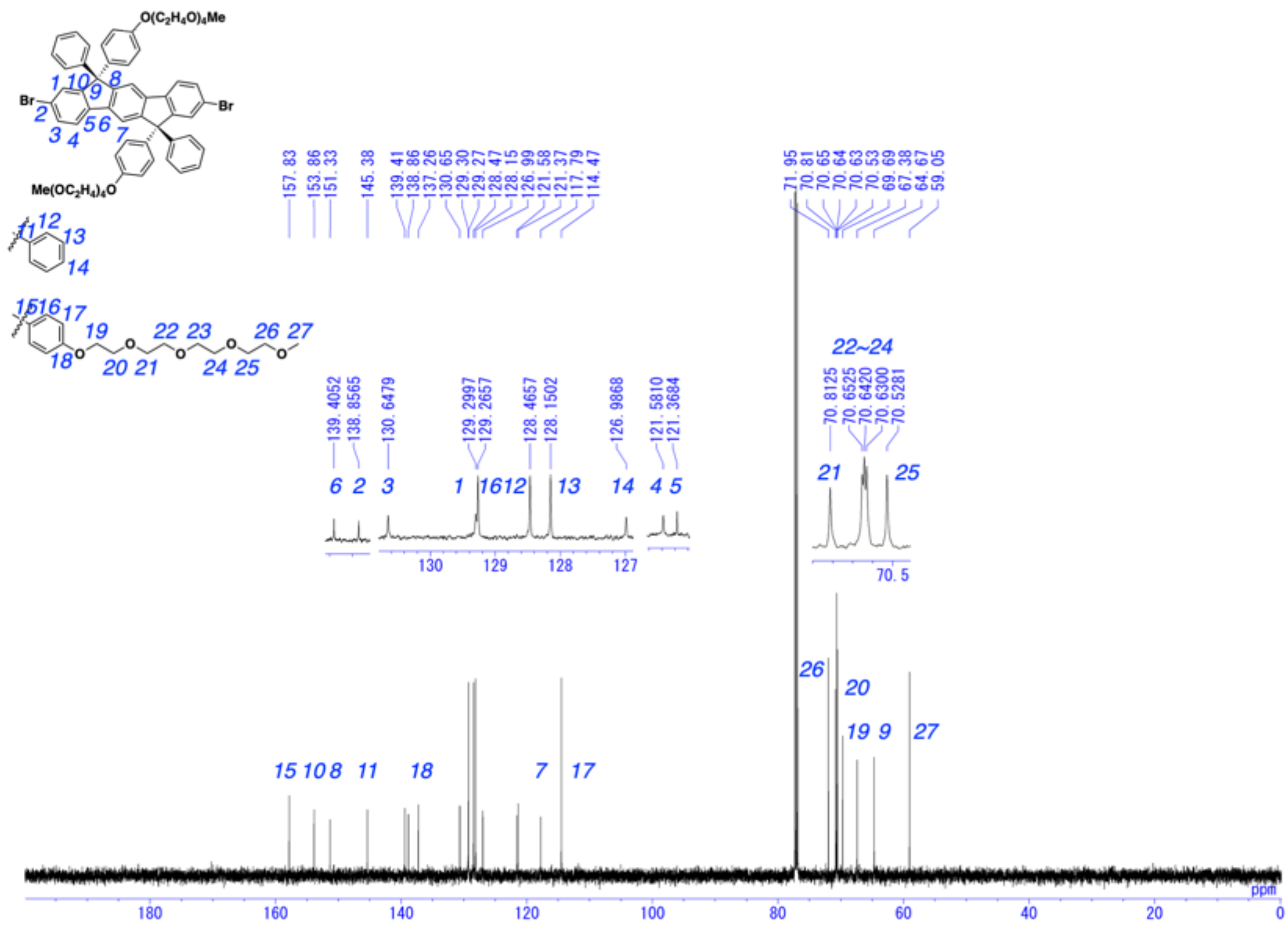

Supplementary Fig. 21 | ^{13}C NMR spectrum (*R,R*)-**1** (151 MHz, $CDCl_3$, 25 ºC).

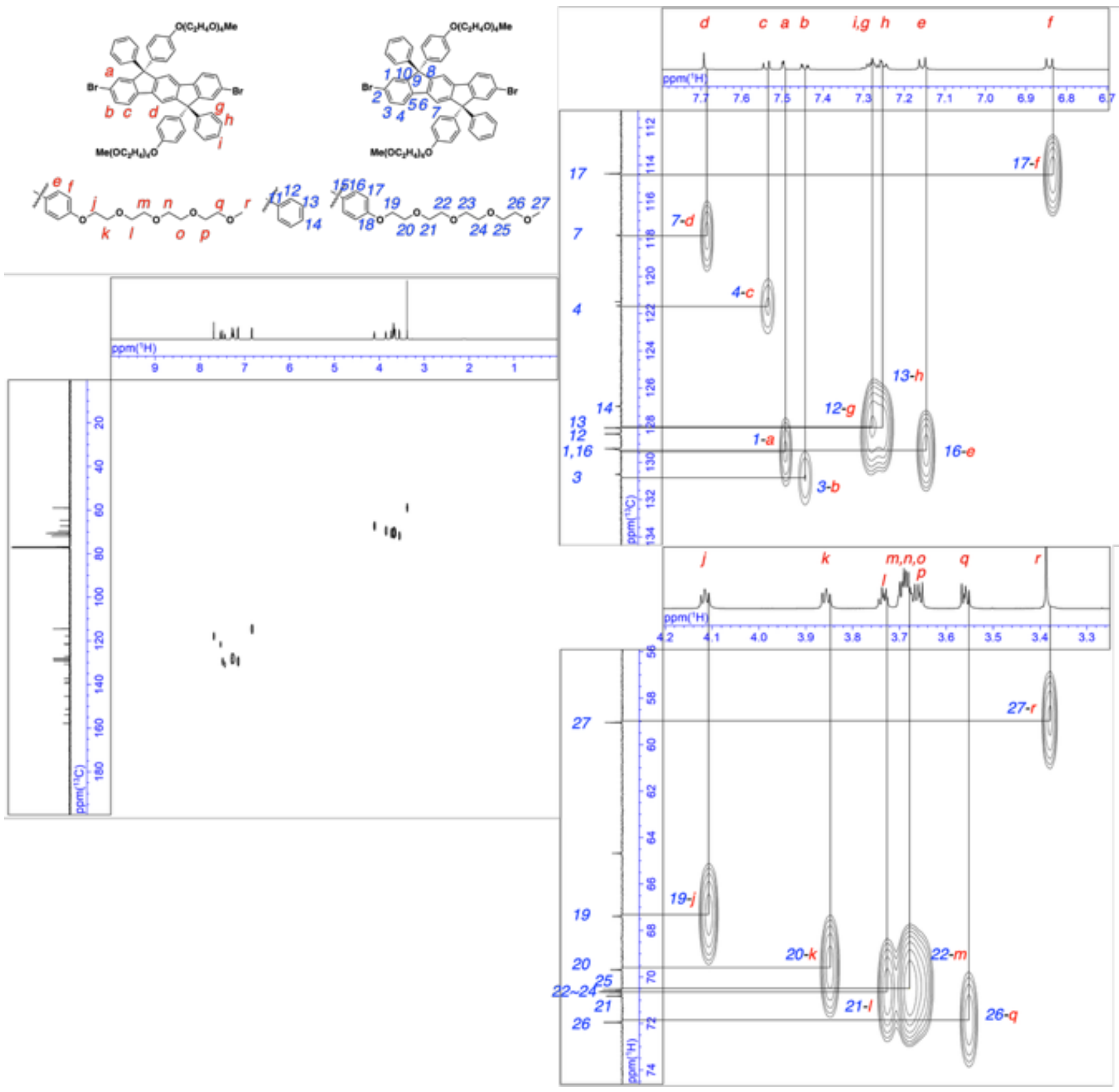

Supplementary Fig. 22 | HSQC correlation of (*R*,*R*)-**1** (600 MHz for ^{1}H and 151 MHz for ^{13}C, $CDCl_3$, 25 ºC).

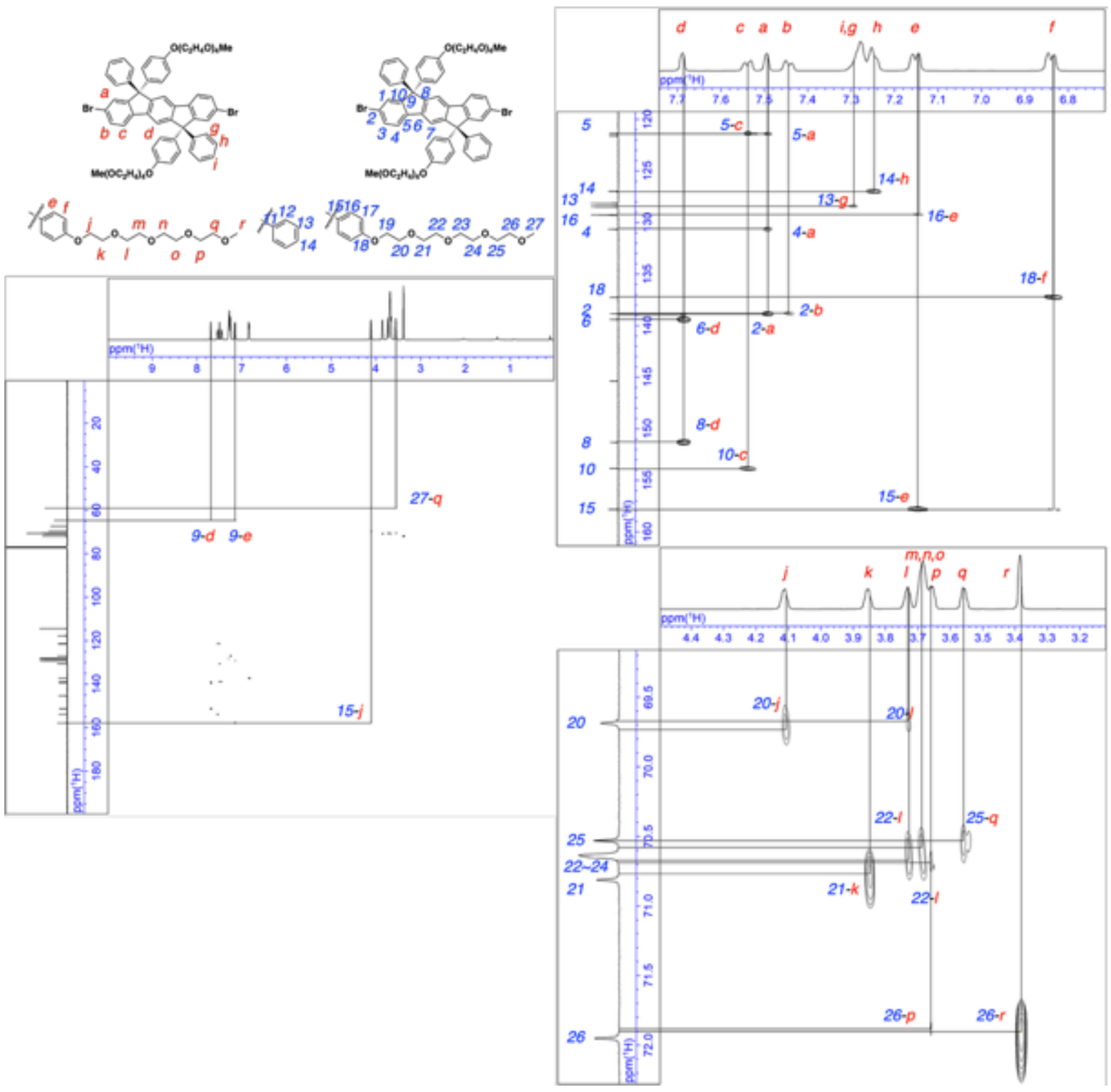

Supplementary Fig. 23 | HMBC correlation of (*R*,*R*)-**1** (600 MHz for ^{1}H and 151 MHz for ^{13}C, $CDCl_3$, 25 ºC).

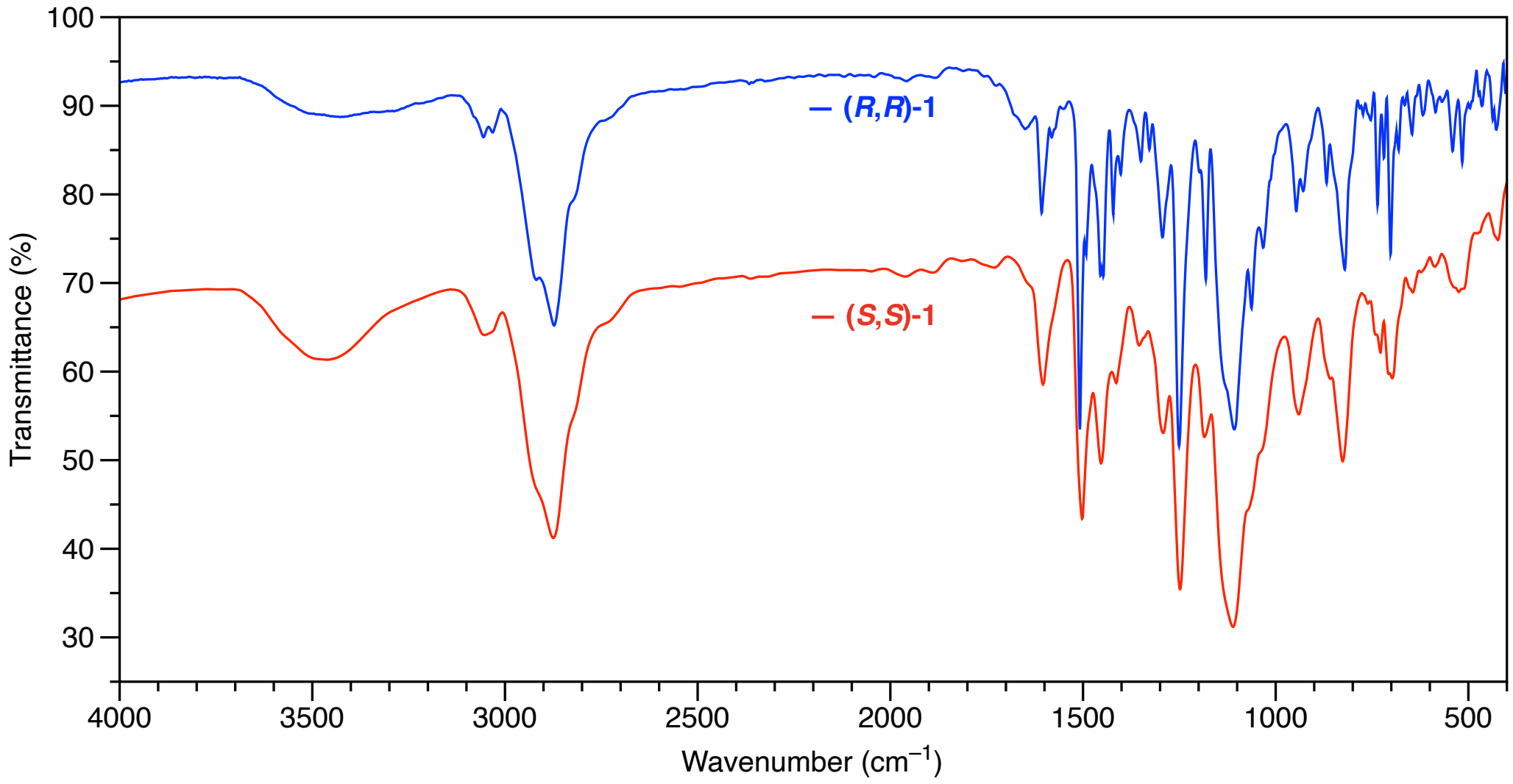

Supplementary Fig. 24 | FT-IR spectra of (*S,S*)-**1** and (*R,R*)-**1** (KBr, 25 °C).

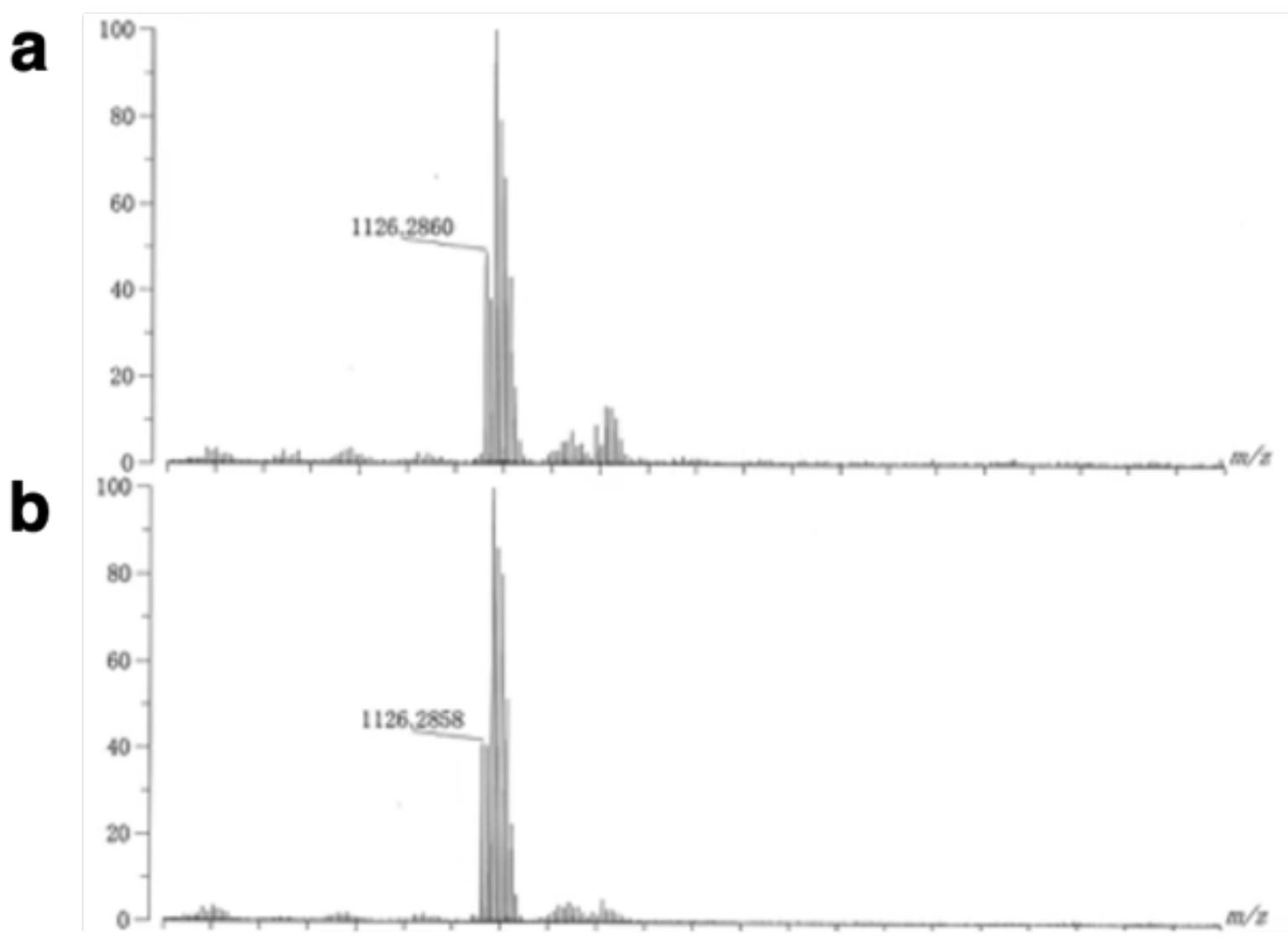

Supplementary Fig. 25 | High-resolution FAB-MS spectra of (*S,S*)-**1** (a) and (*R,R*)-**1** (b).

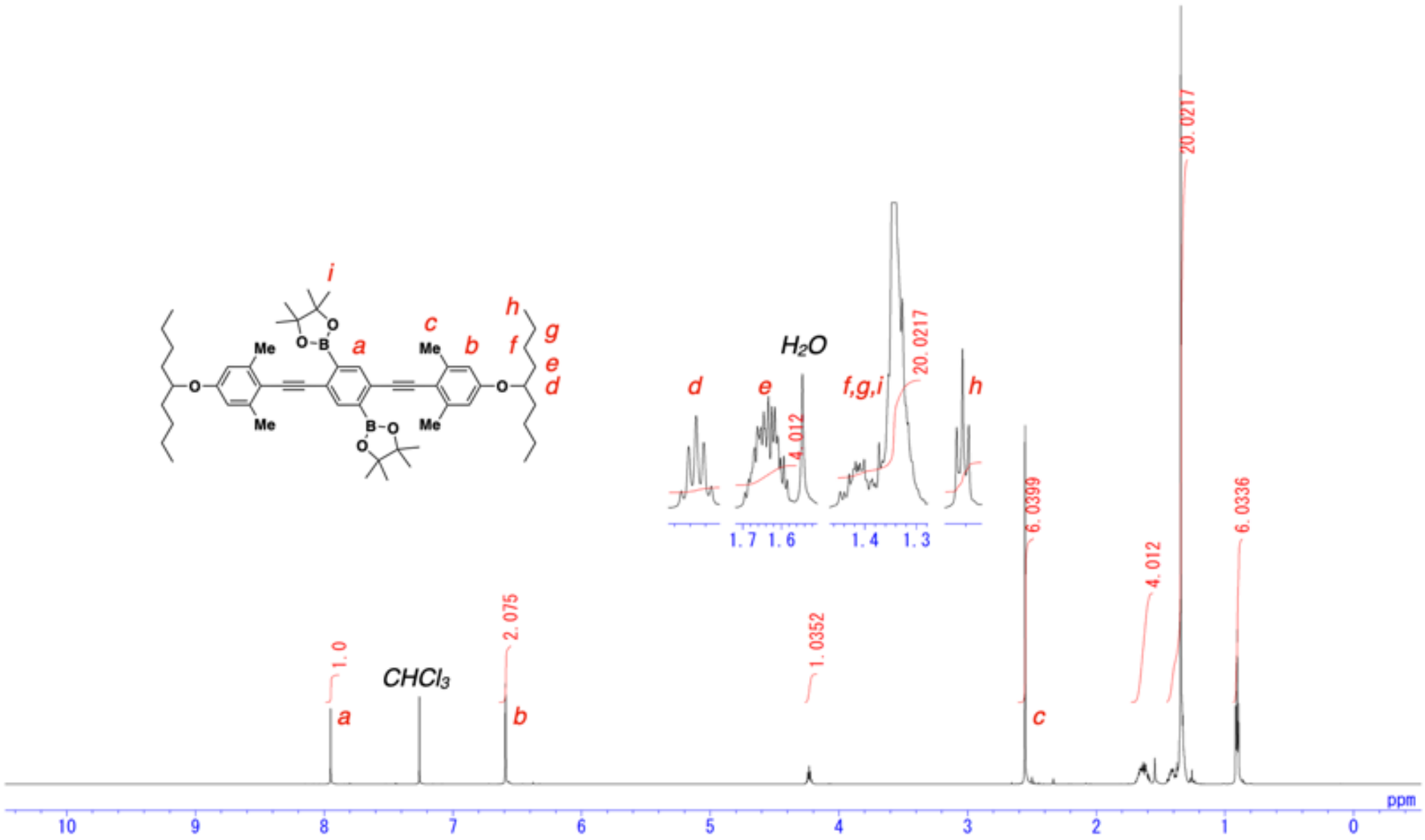

Supplementary Fig. 26 | ^{1}H NMR spectrum of **2** (600 MHz, $CDCl_3$, 25 ºC).

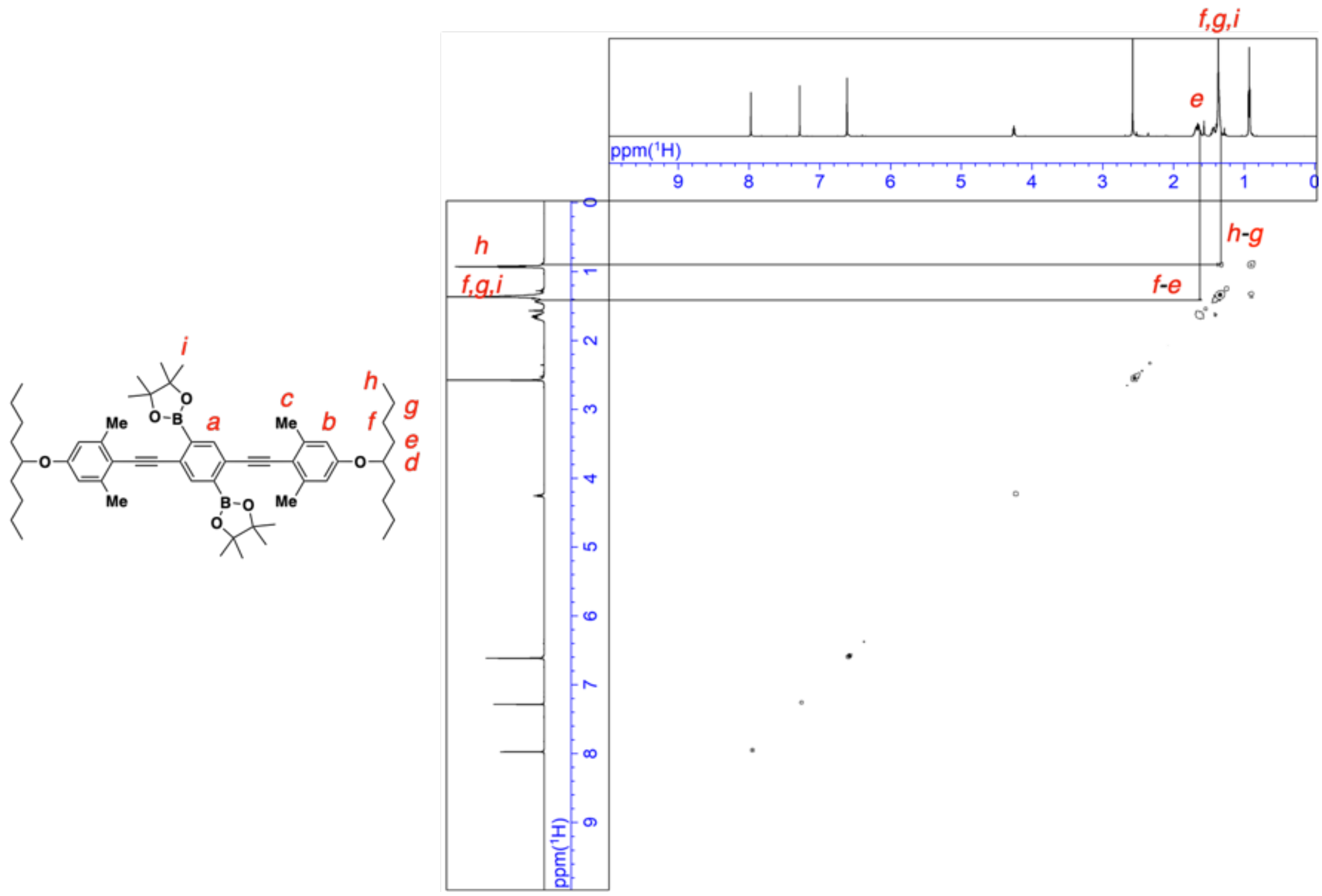

Supplementary Fig. 27 | HH-COSY correlation of **2** (600 MHz, $CDCl_3$, 25 ºC).

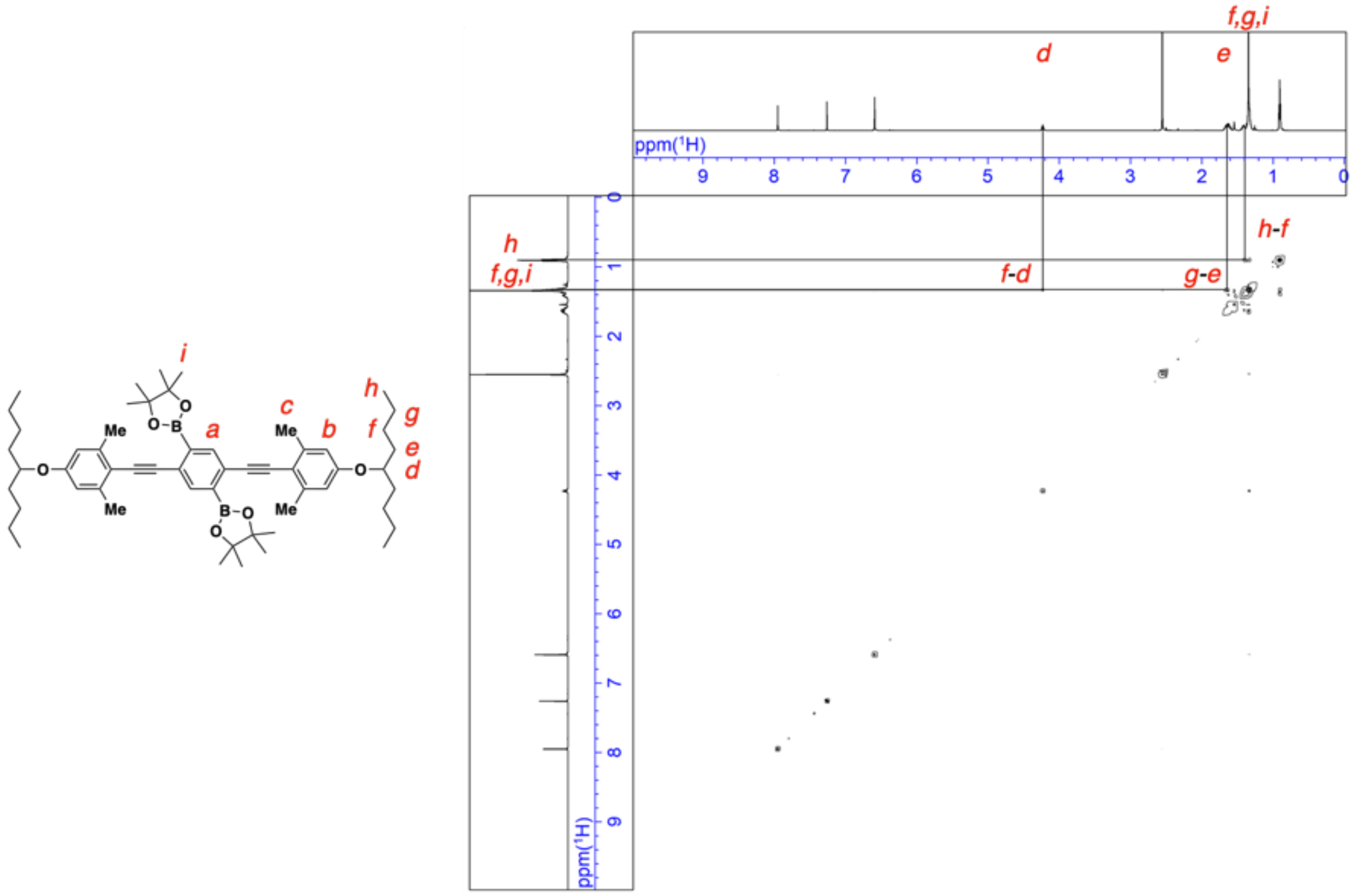

Supplementary Fig. 28 | NOESY correlation of **2** (600 MHz, $CDCl_3$, 25 ºC).

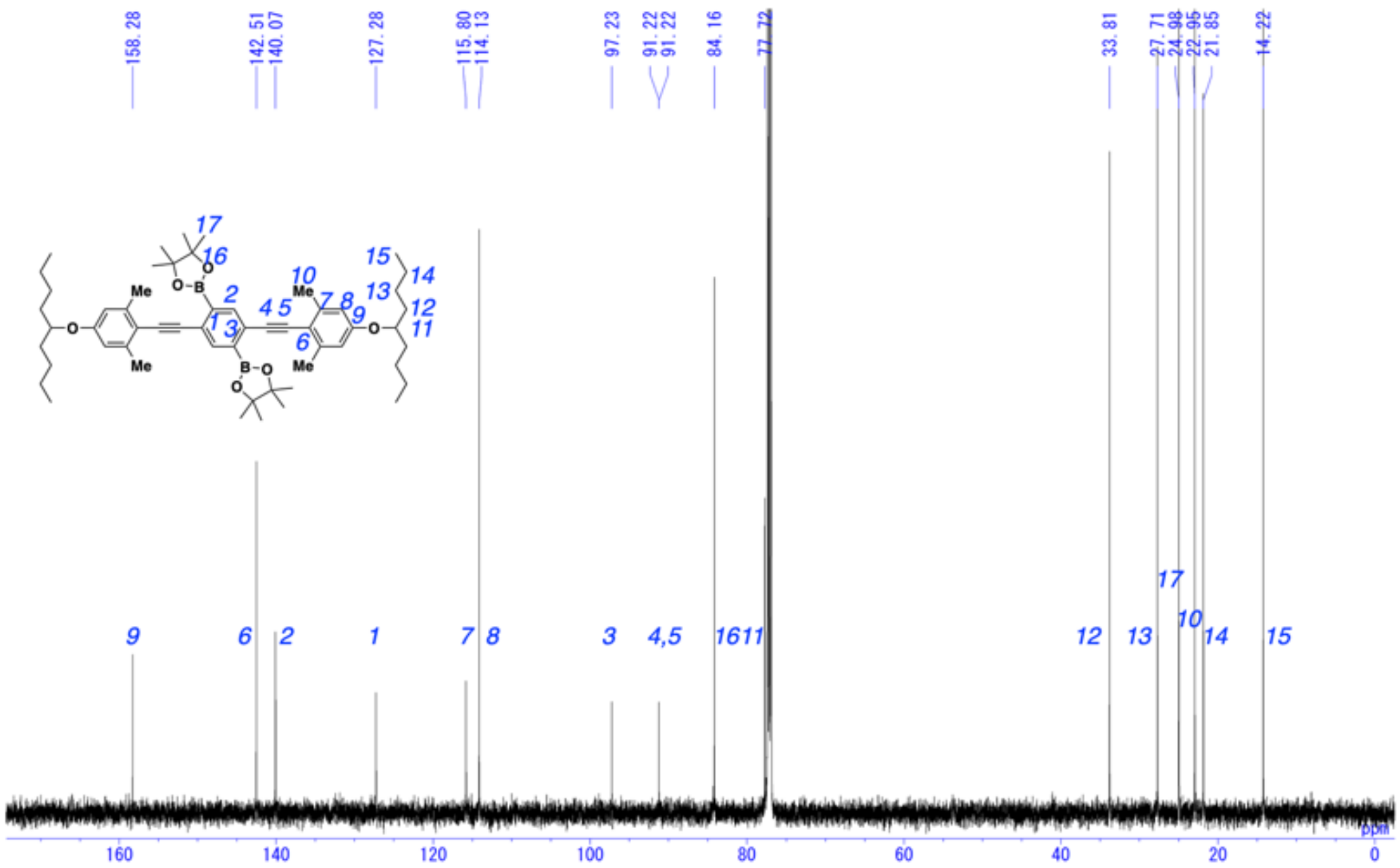

Supplementary Fig. 29 | ^{13}C NMR spectrum of **2** (151 MHz, $CDCl_3$, 25 ºC).

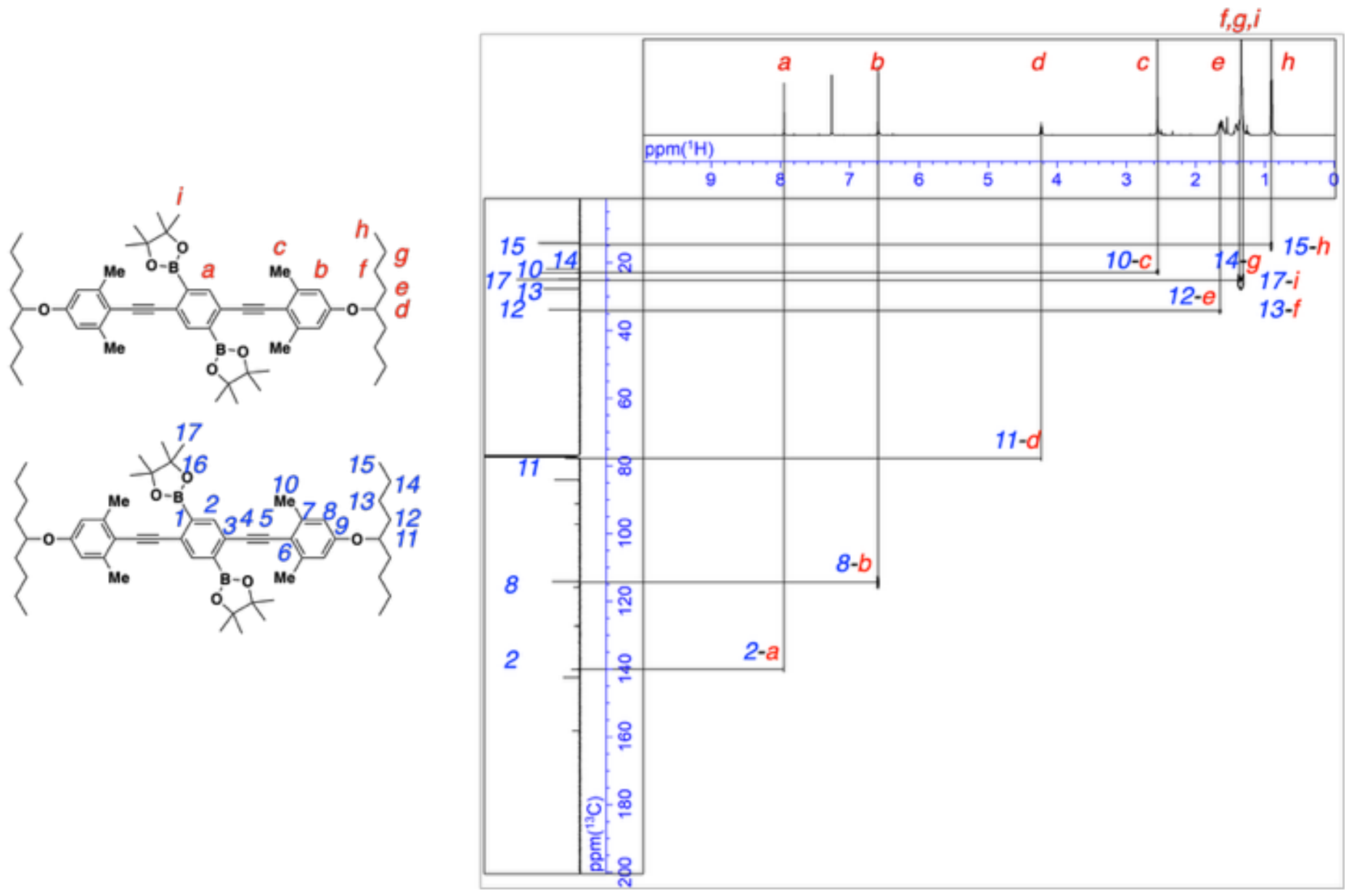

Supplementary Fig. 30 | HSQC correlation of **2** (600 MHz for ^{1}H and 151 MHz for ^{13}C, $CDCl_3$, 25 ºC).

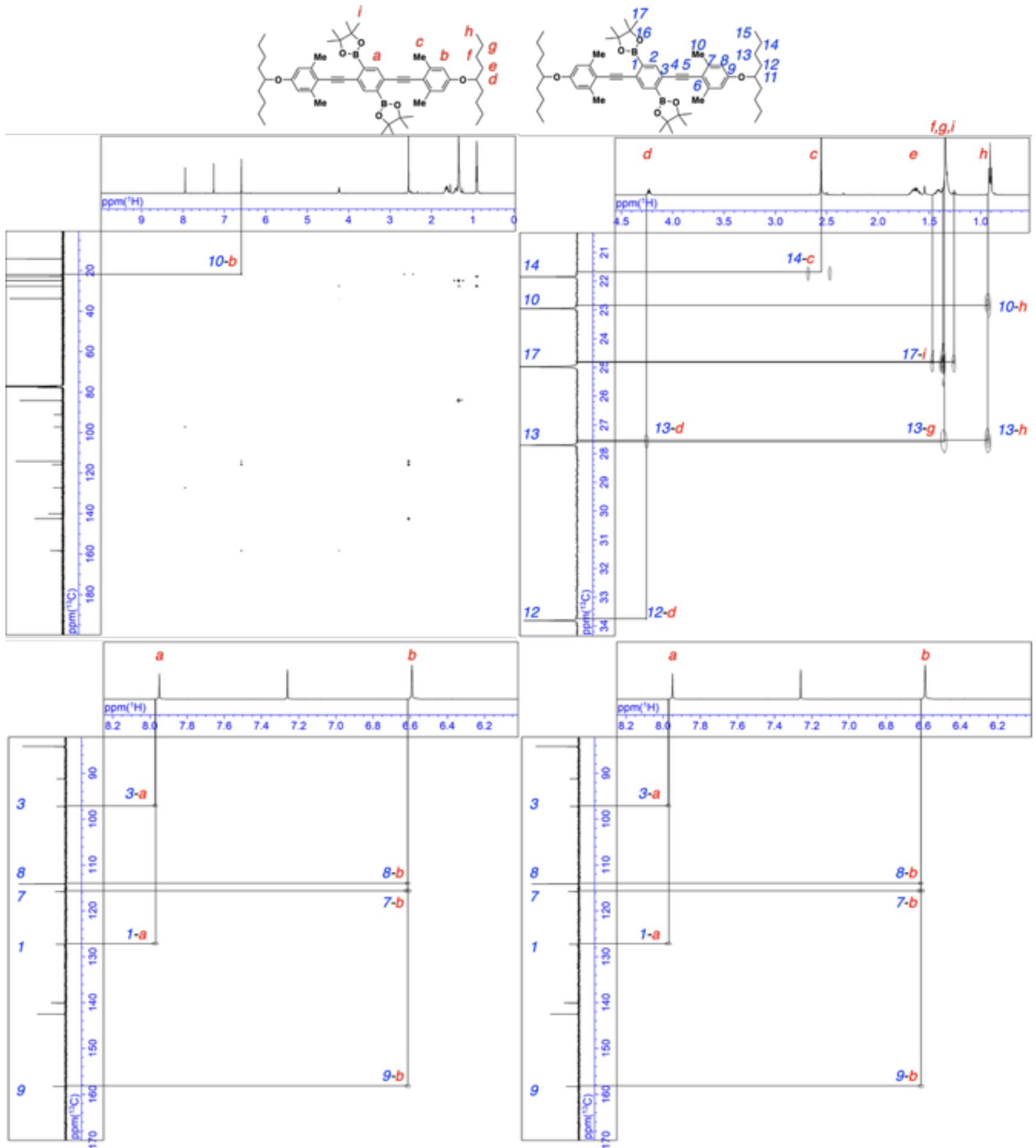

Supplementary Fig. 31 | HMBC correlation of **2** (600 MHz for ^{1}H and 151 MHz for ^{13}C, $CDCl_3$, 25 ºC).

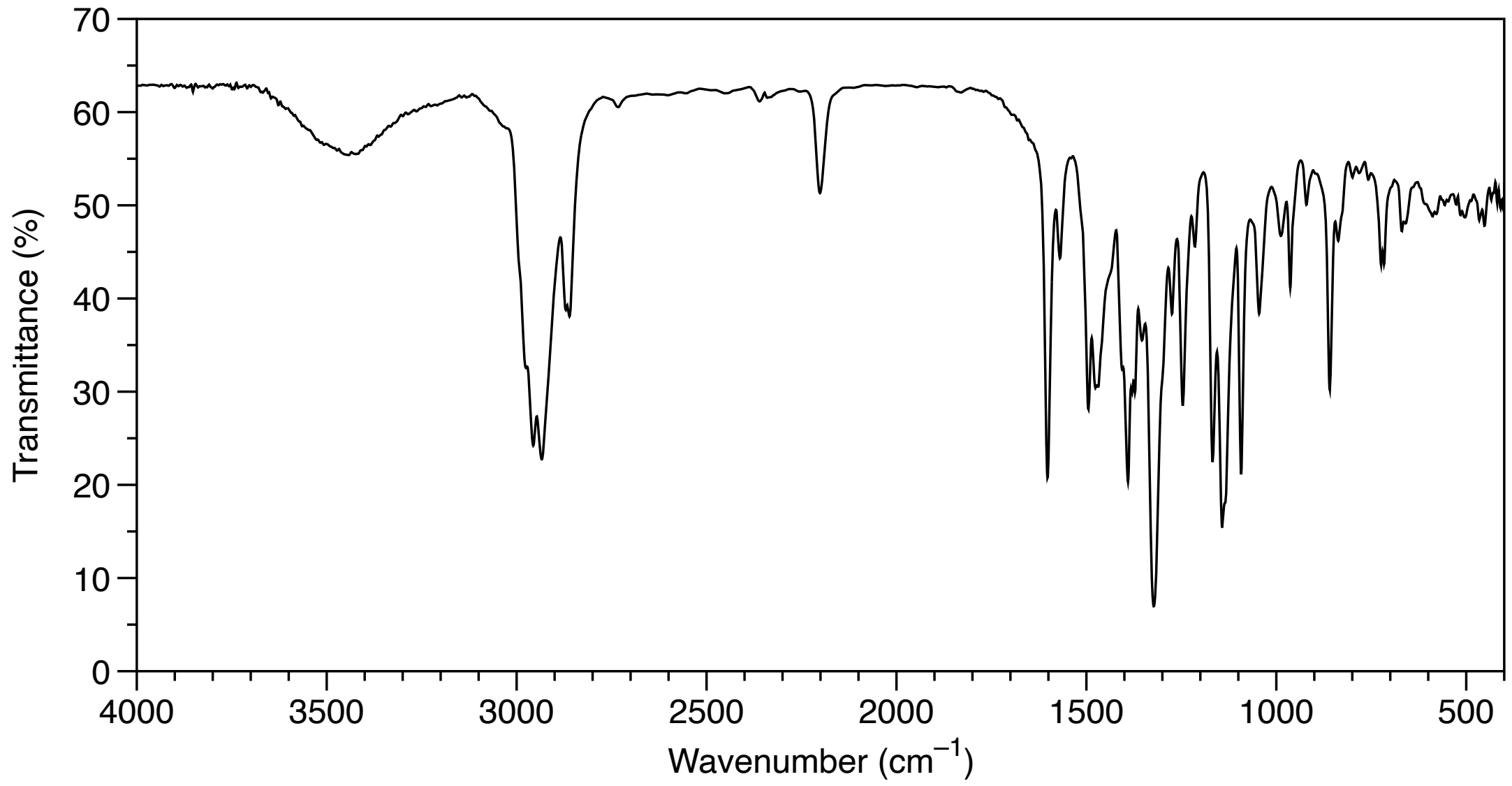

Supplementary Fig. 32 | FT-IR spectrum of **2** (KBr, 25 °C).

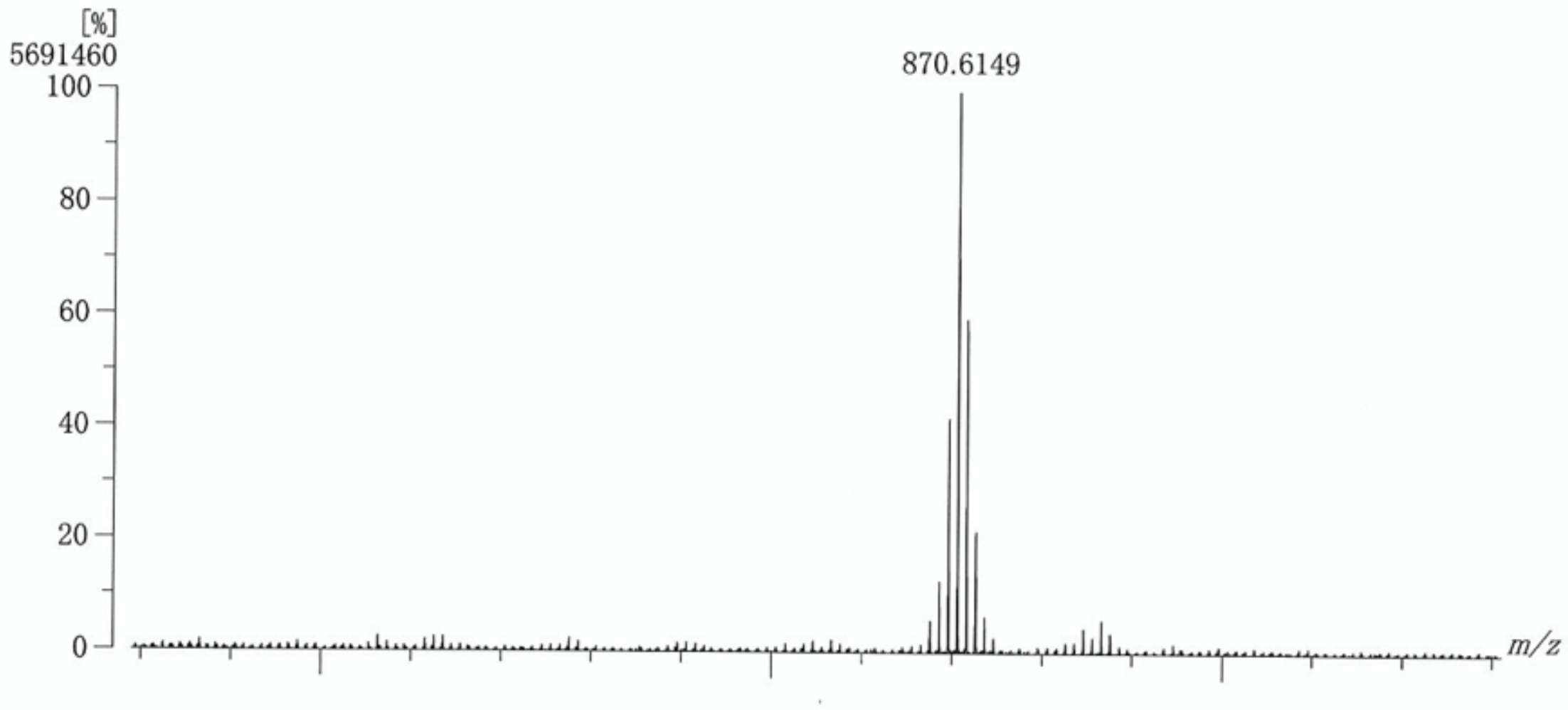

Supplementary Fig. 33 | High-resolution FAB-MS spectra of **2**.

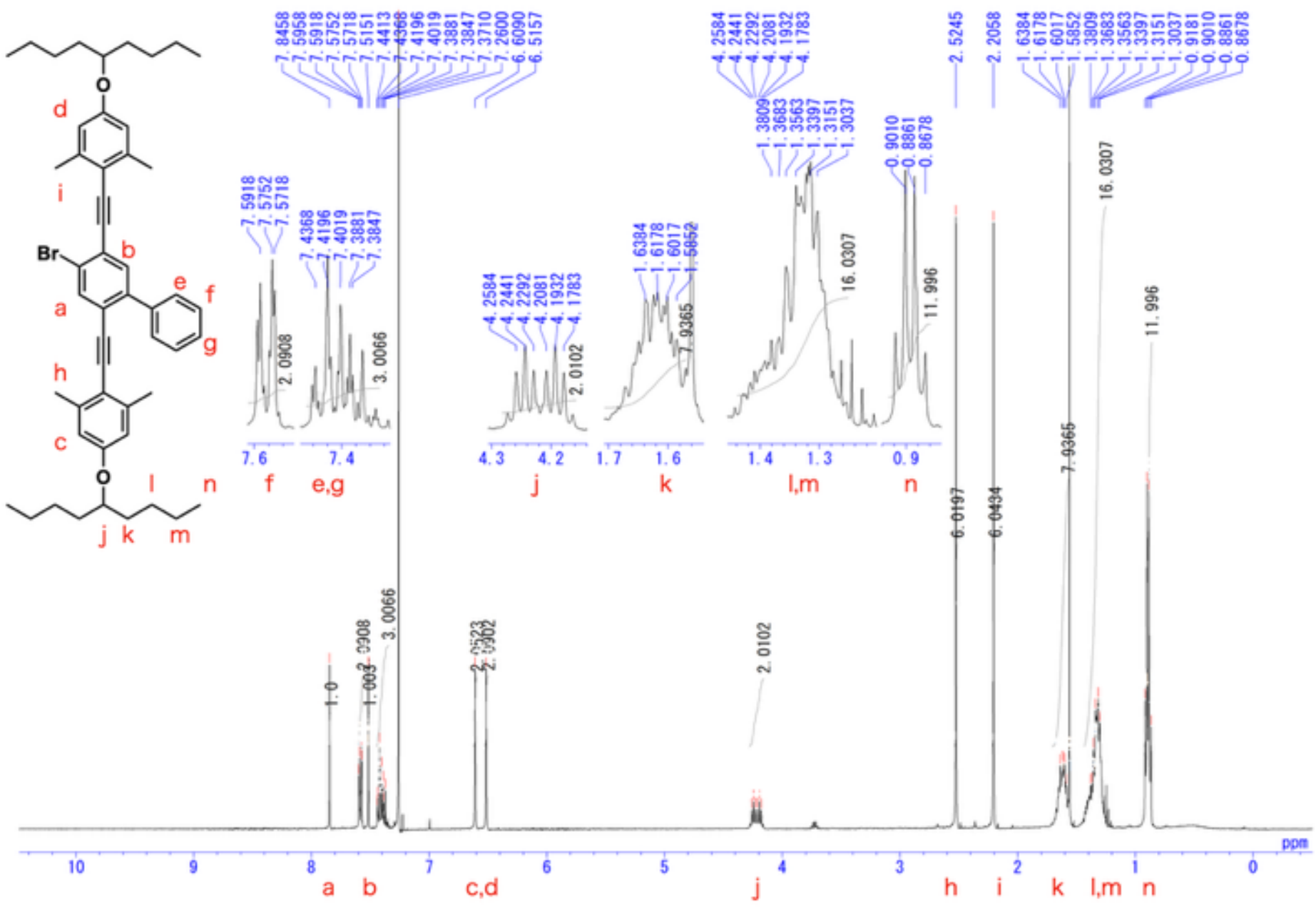

Supplementary Fig. 34 | ^{1}H NMR spectrum of **7** (400 MHz, $CDCl_3$, 25 ºC).

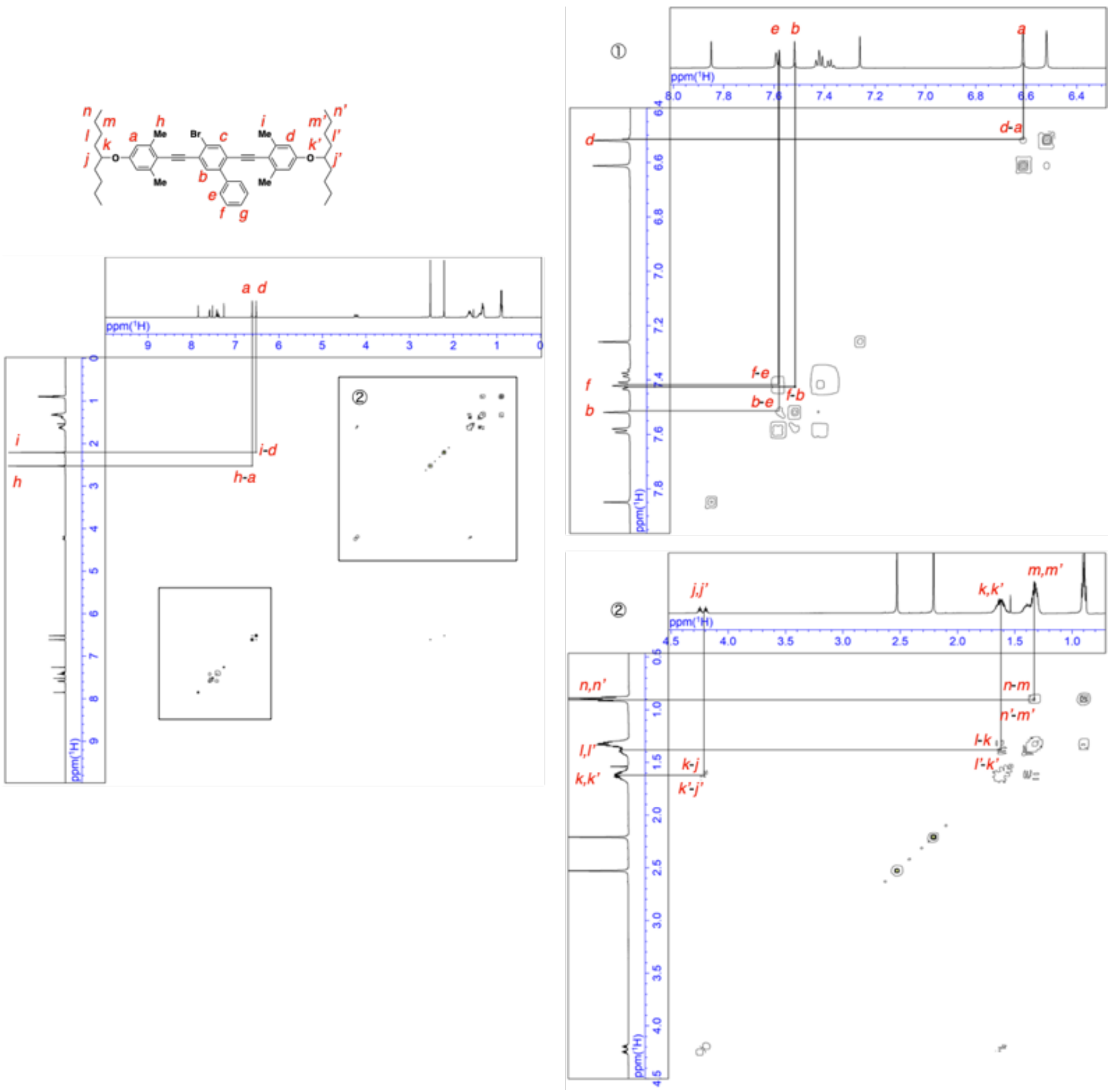

Supplementary Fig. 35 | HH-COSY correlation of **7** (600 MHz, $CDCl_3$, 25 ºC).

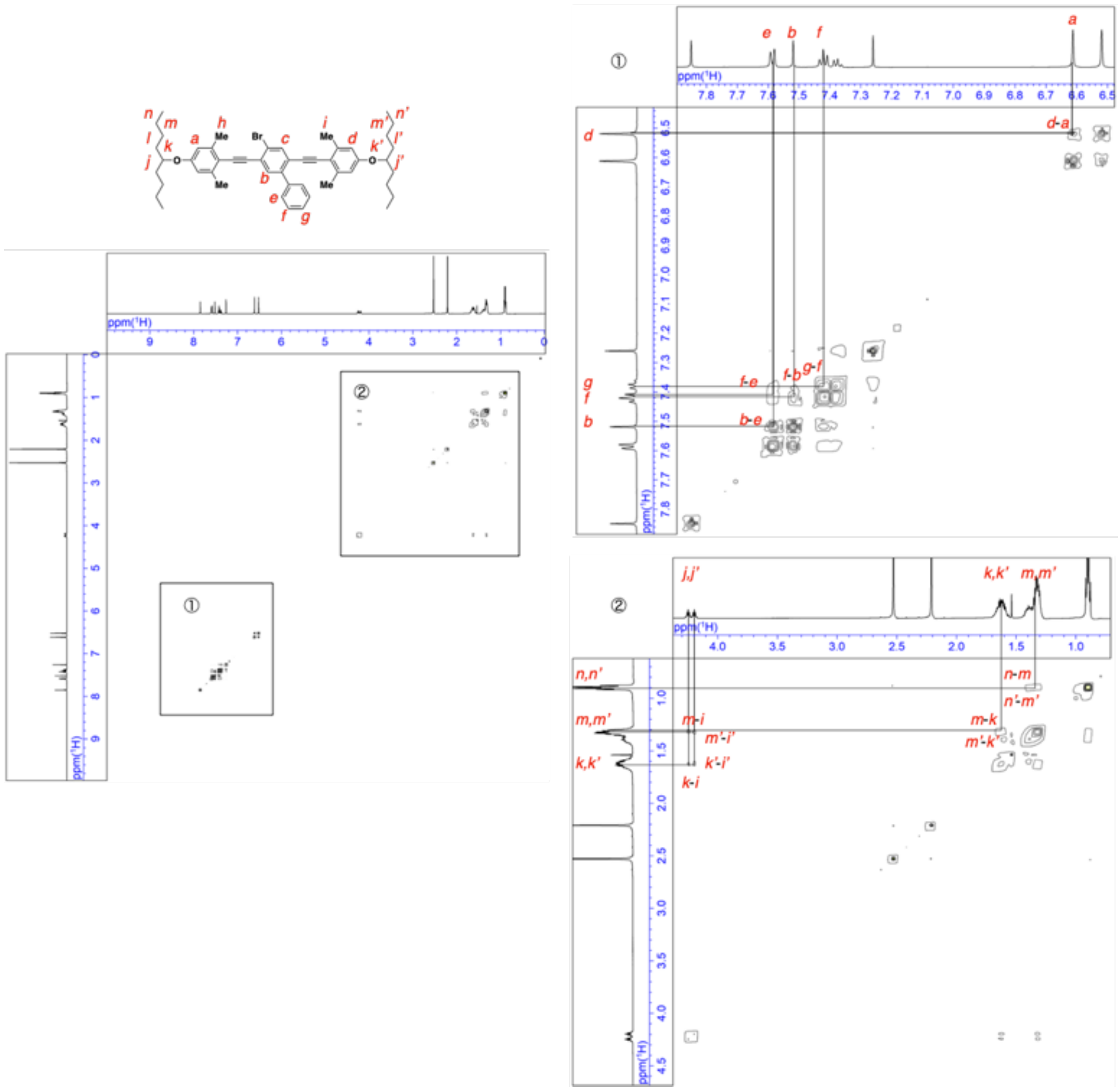

Supplementary Fig. 36 |NOESY correlation of **7** (600 MHz, $CDCl_3$, 25 ºC).

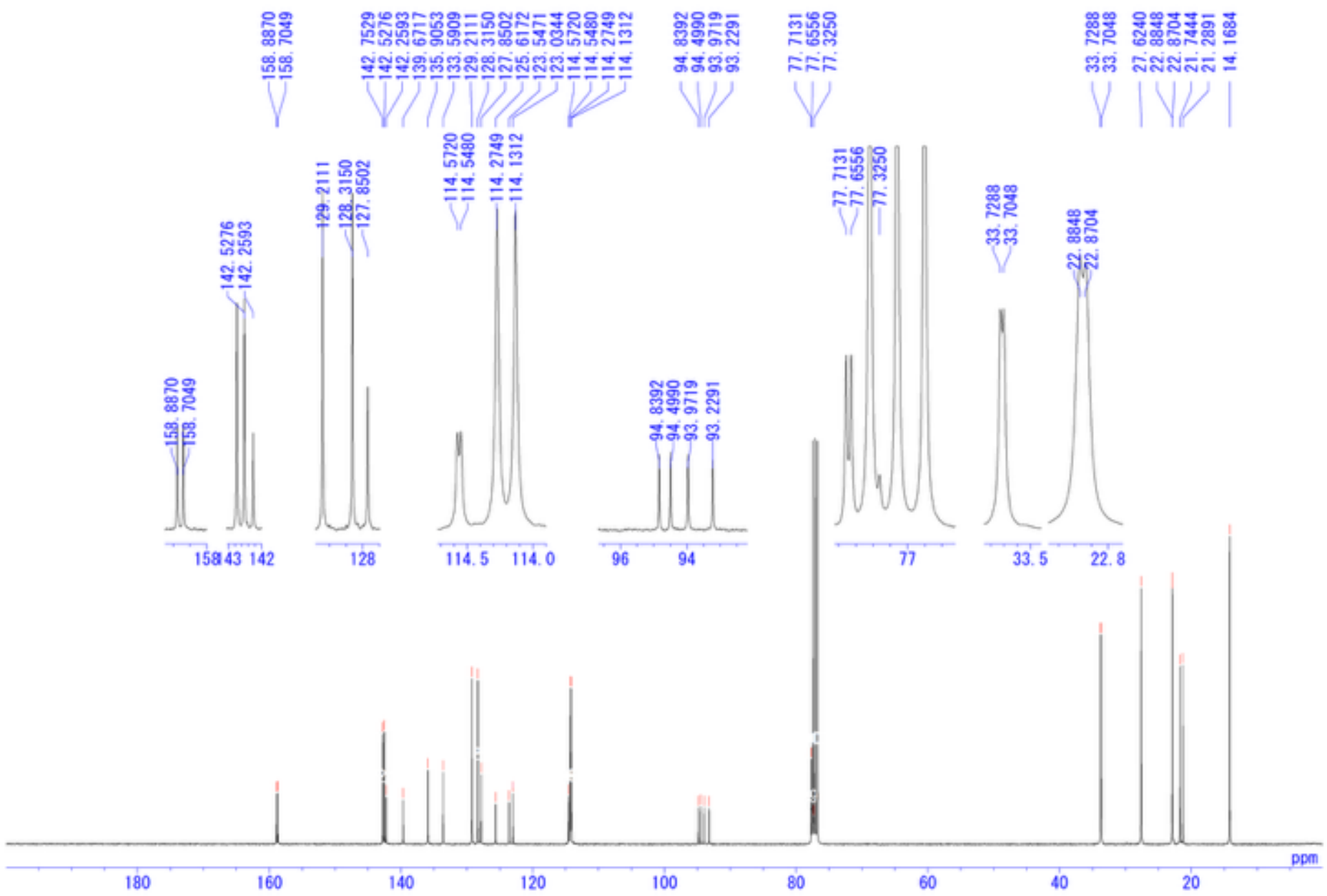

Supplementary Fig. 37 | ^{13}C NMR spectrum of **7** (151 MHz, $CDCl_3$, 25 ºC).

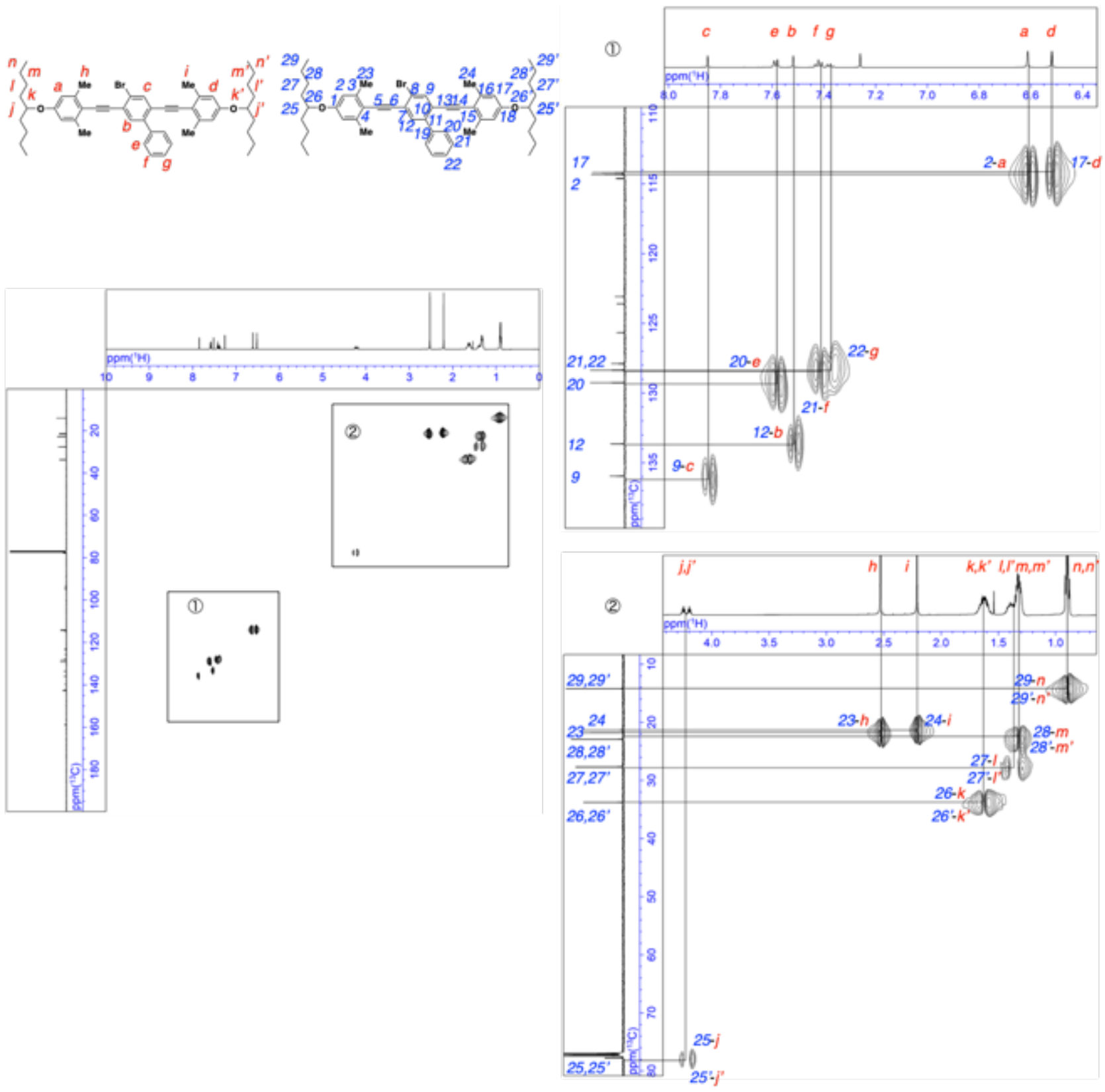

Supplementary Fig. 38 | HSQC correlation of **7** (600 MHz for ^{1}H and 151 MHz for ^{13}C, $CDCl_3$, 25 ºC).

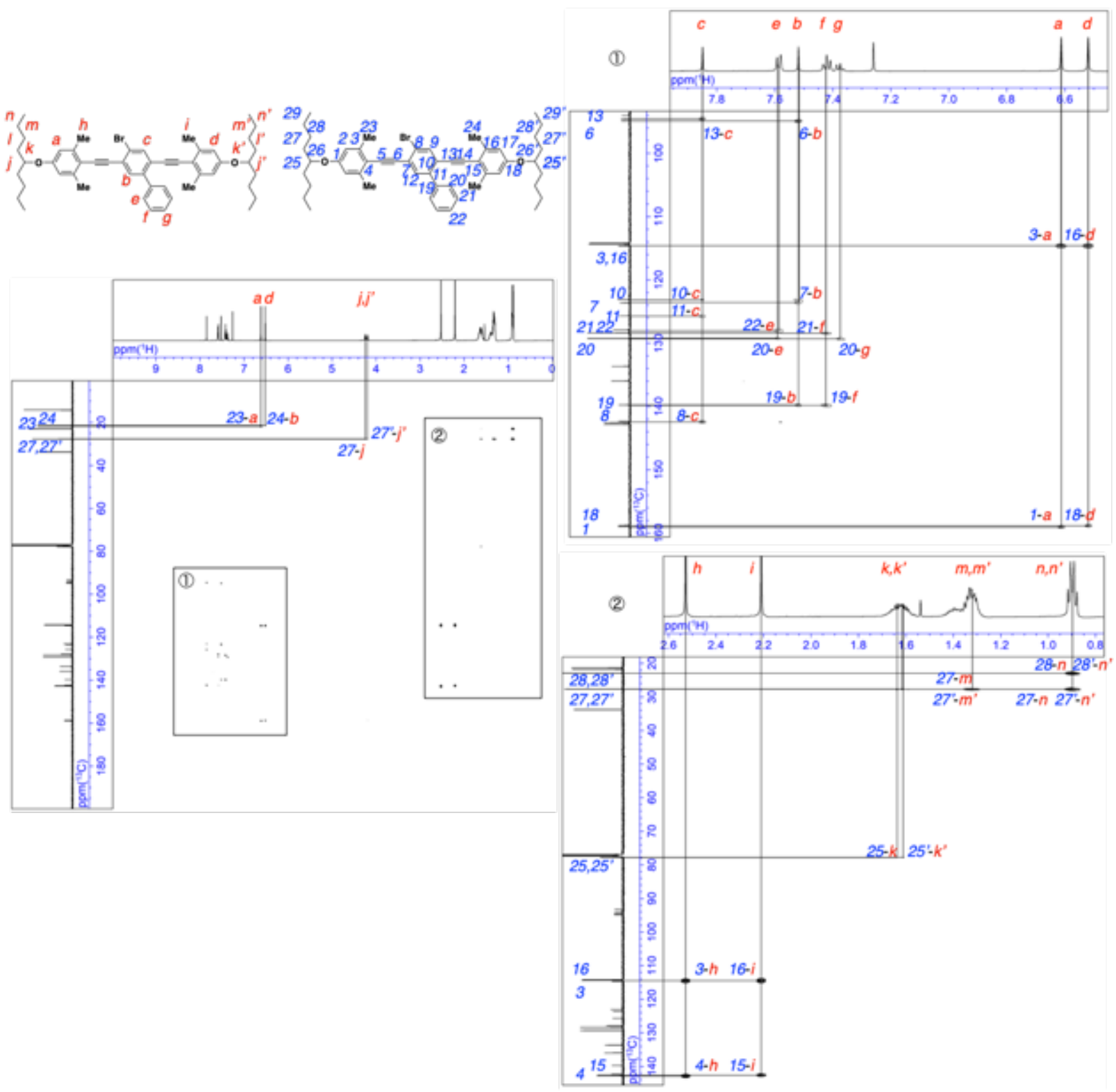

Supplementary Fig. 39 | HMBC correlation of **7** (600 MHz for ^{1}H and 151 MHz for ^{13}C, $CDCl_3$, 25 ºC).

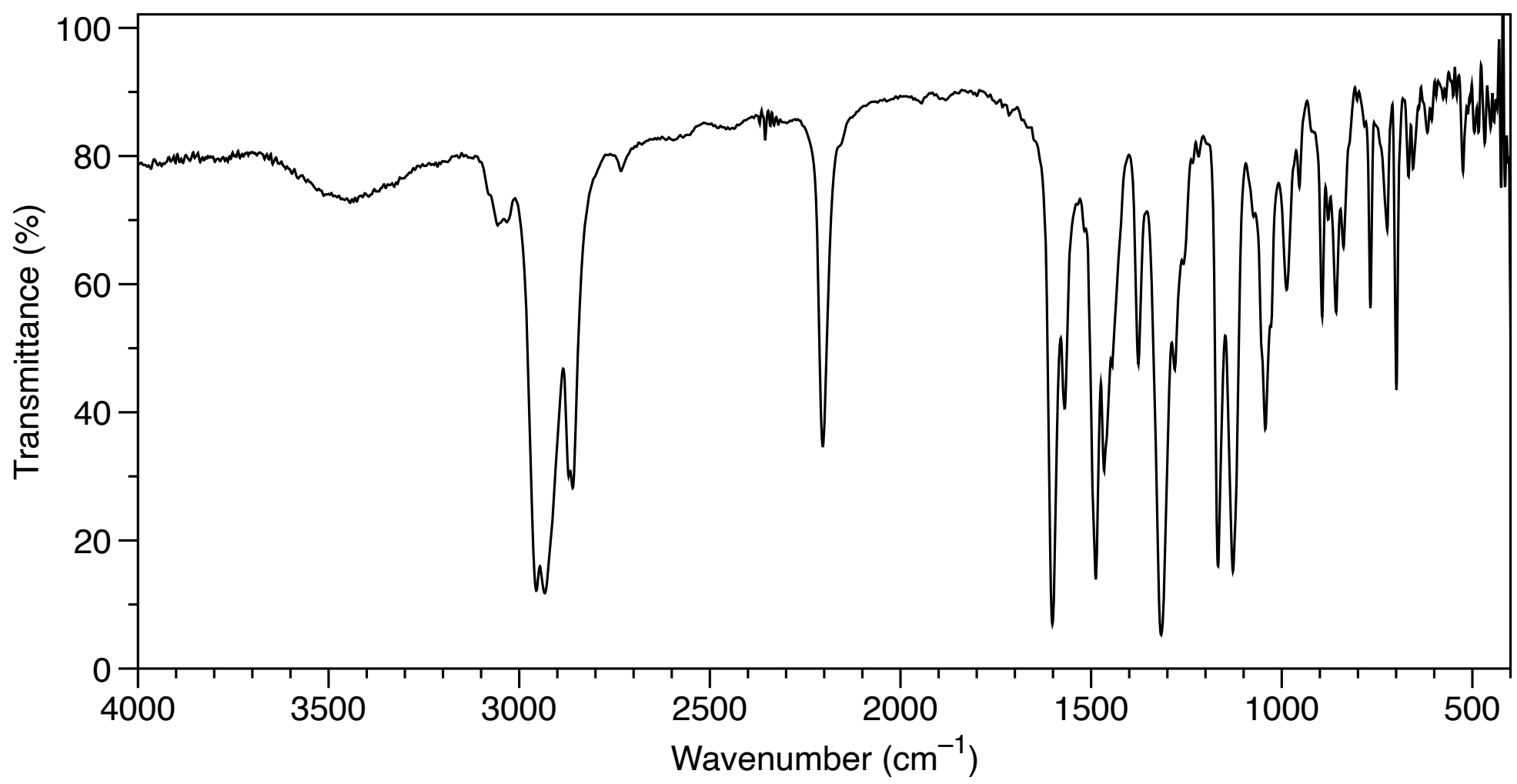

Supplementary Fig. 40 | FT-IR spectrum of **7** (KBr, 25 °C).

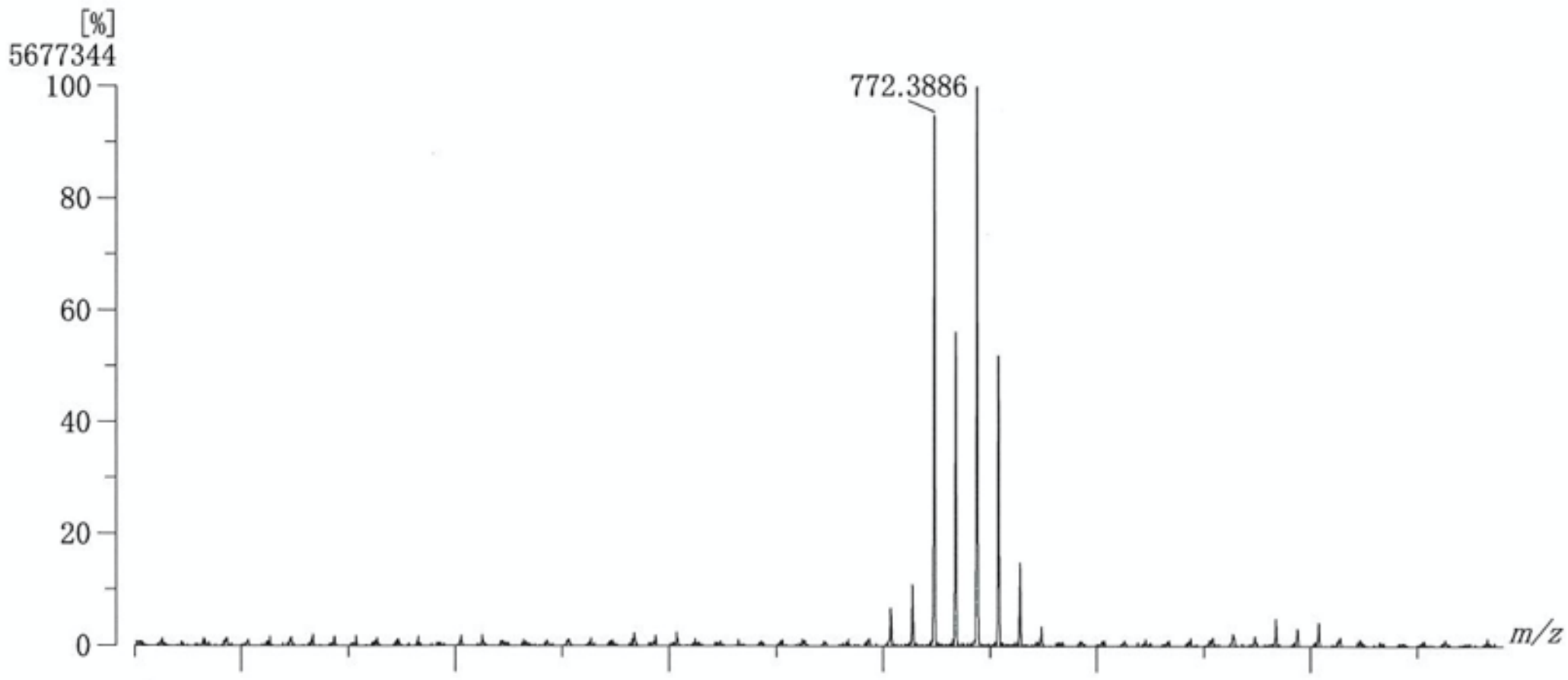

Supplementary Fig. 41 | High-resolution FAB-MS spectra of **7**.

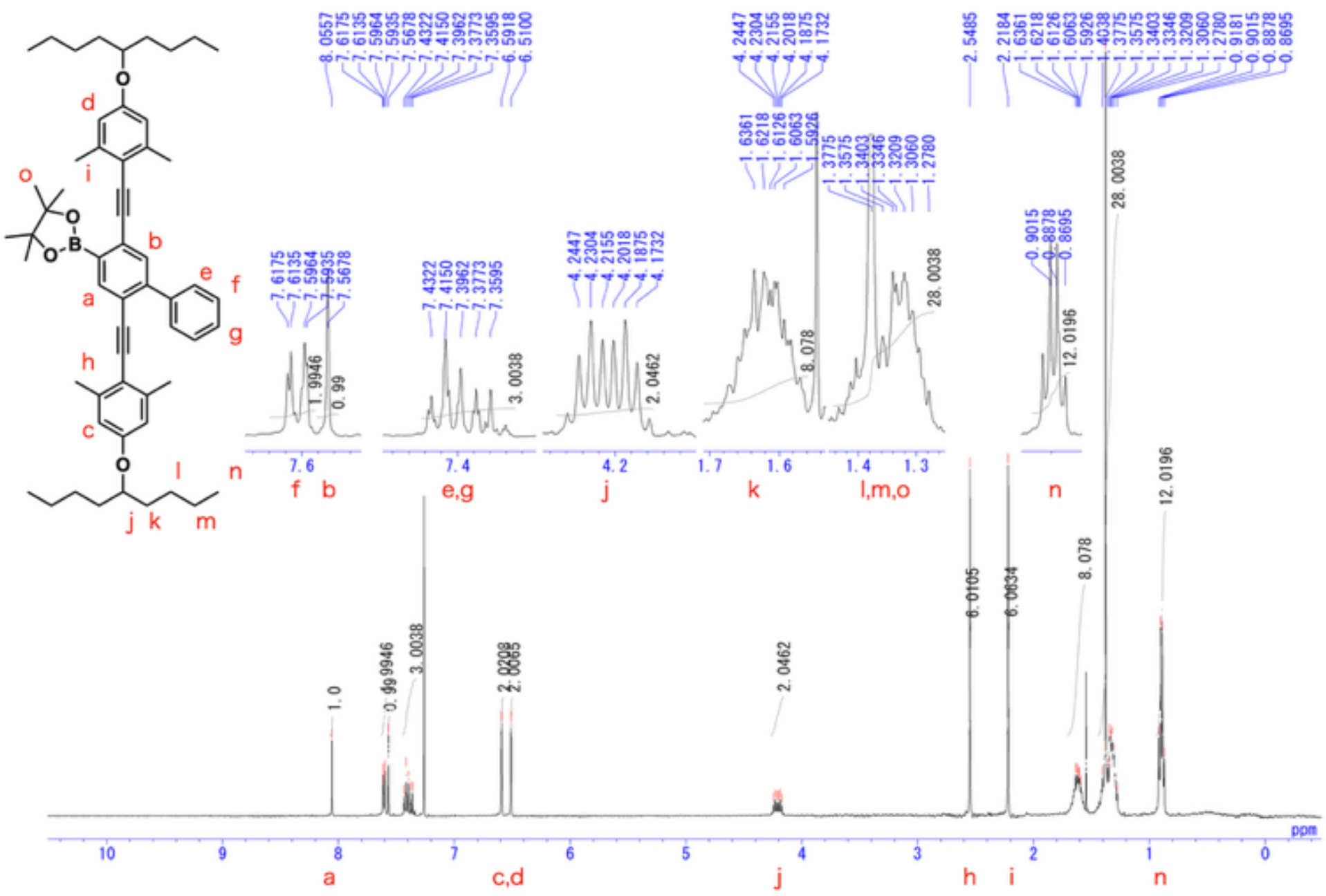

Supplementary Fig. 42 | ^{1}H NMR spectrum of Model-**2** (600 MHz, $CDCl_3$, 25 ºC).

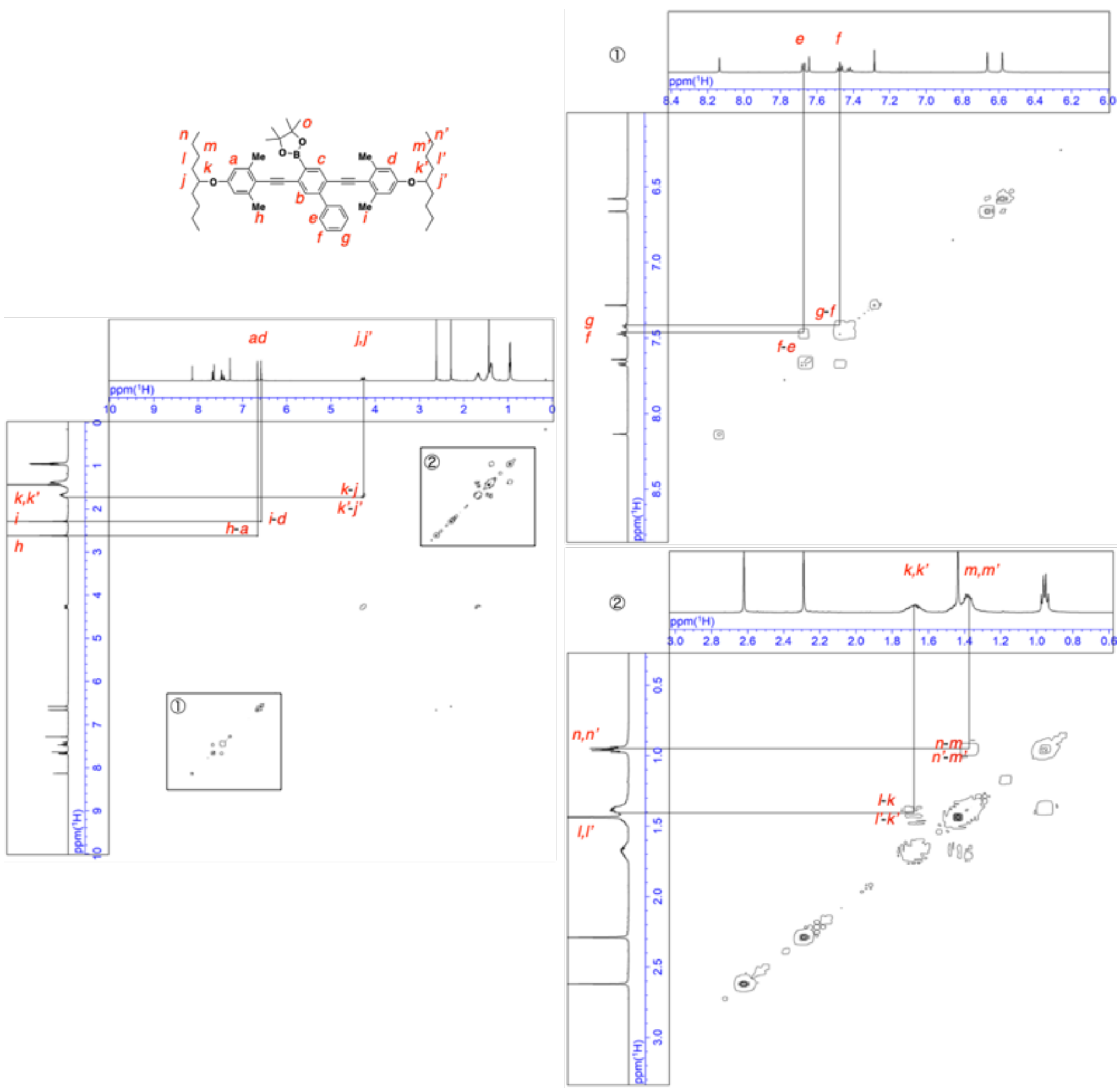

Supplementary Fig. 43 | HH-COSY correlation of Model-**2** (600 MHz, $CDCl_3$, 25 ºC).

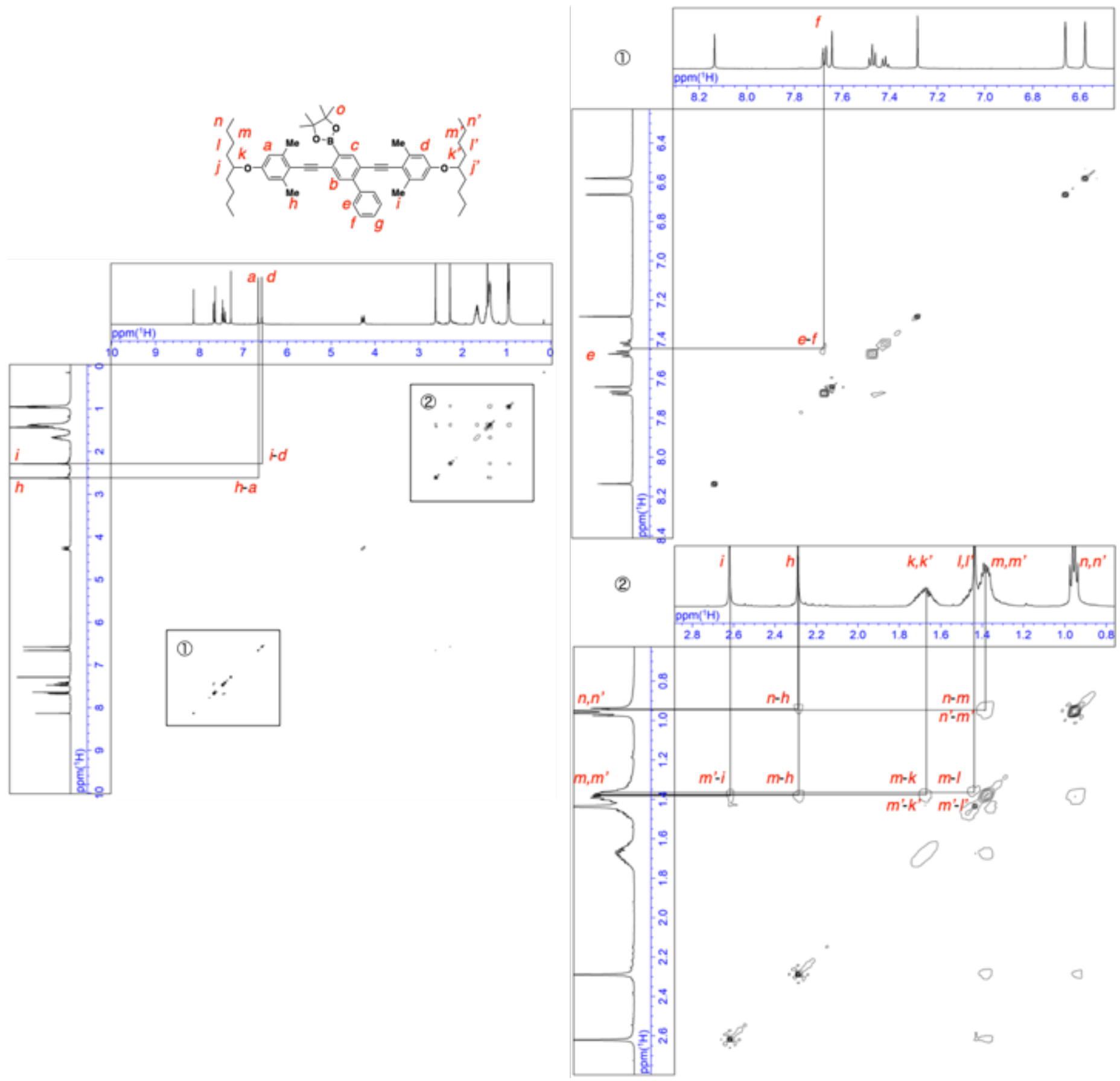

Supplementary Fig. 44 | NOESY correlation of Model-**2** (600 MHz, $CDCl_3$, 25 ºC).

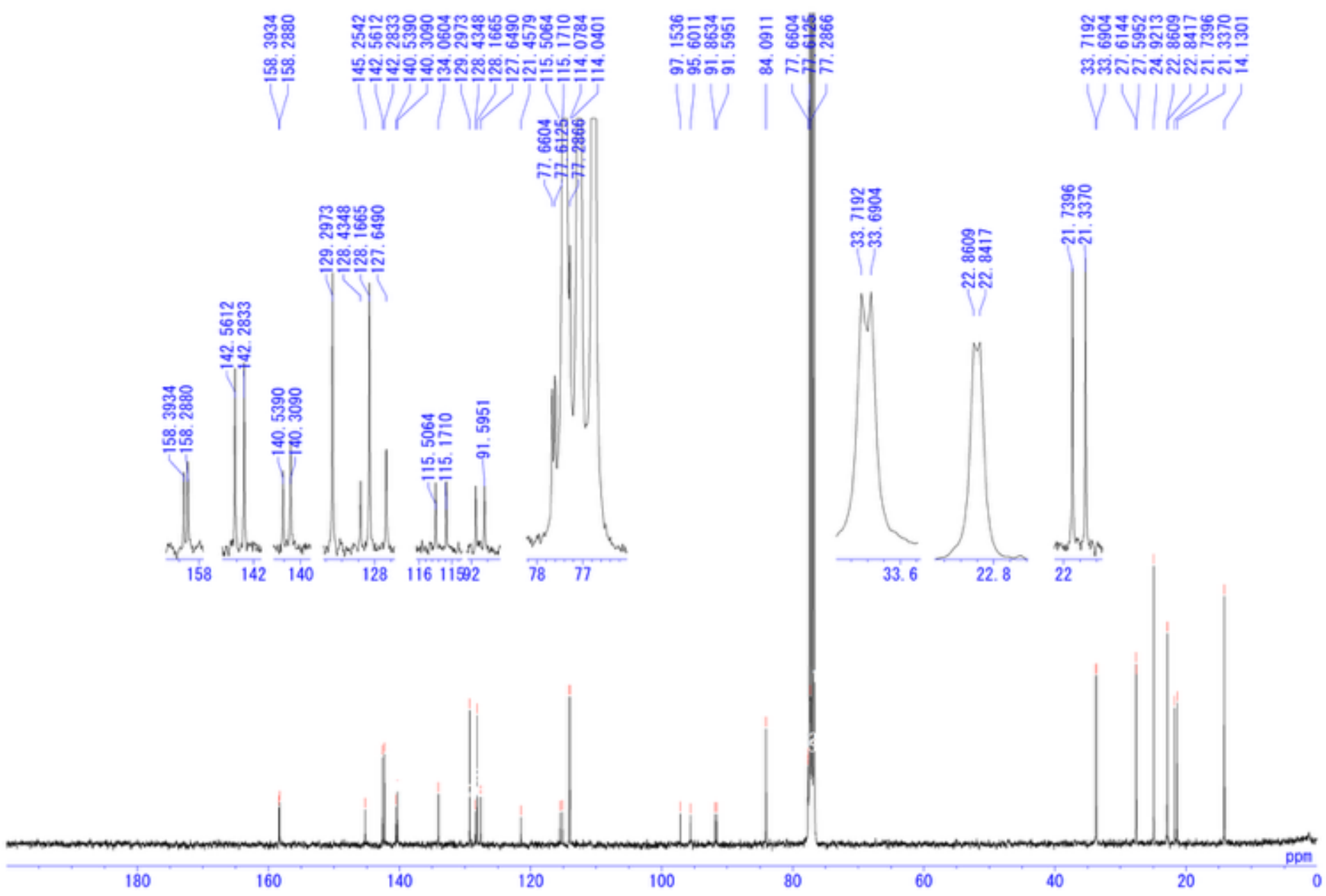

Supplementary Fig. 45 | ^{13}C NMR spectrum of Model-**2** (151 MHz, $CDCl_3$, 25 ºC).

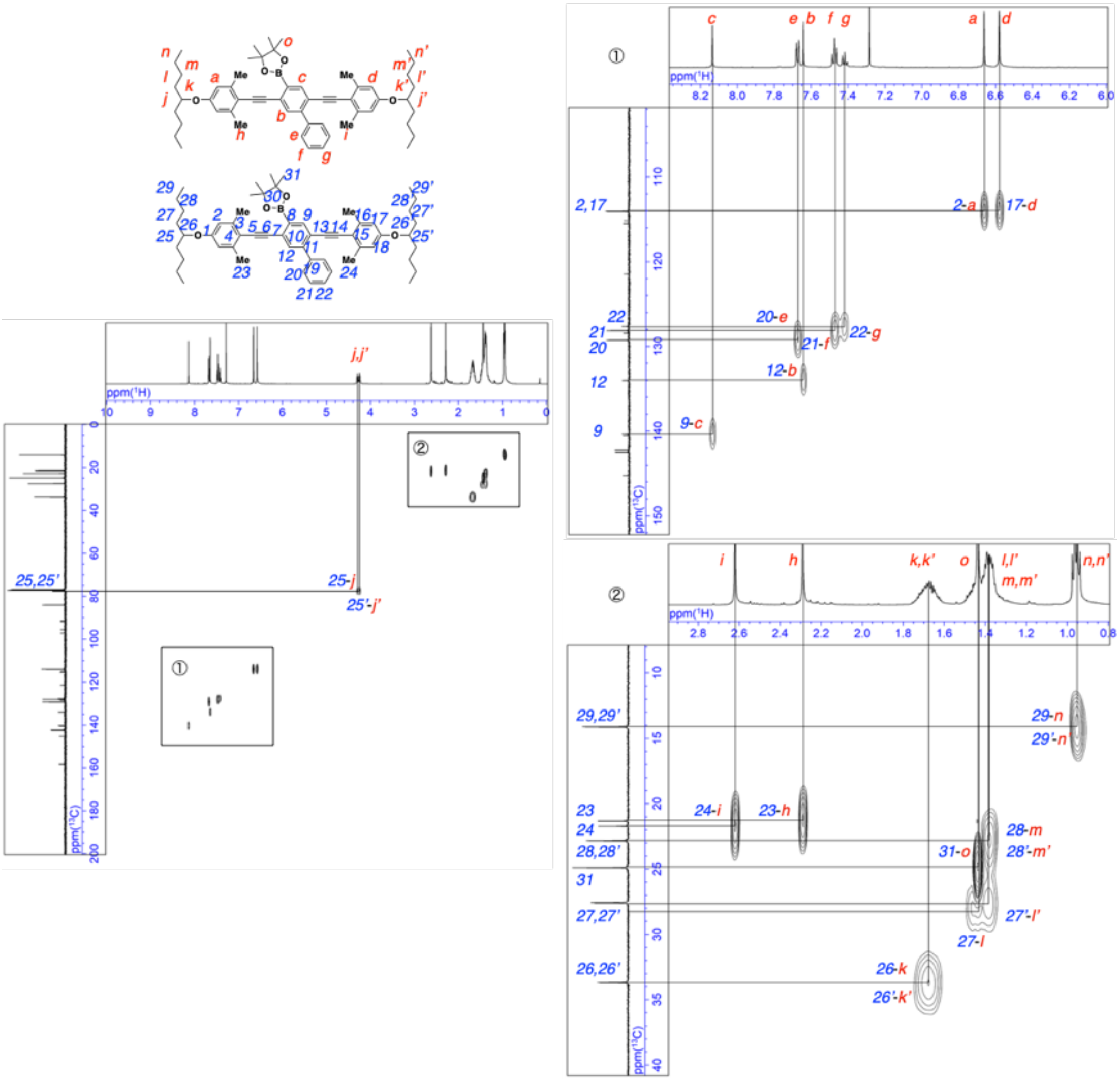

Supplementary Fig. 46 | HSQC correlation of Model-**2** (600 MHz for ^{1}H and 151 MHz for ^{13}C, $CDCl_3$, 25 ºC).

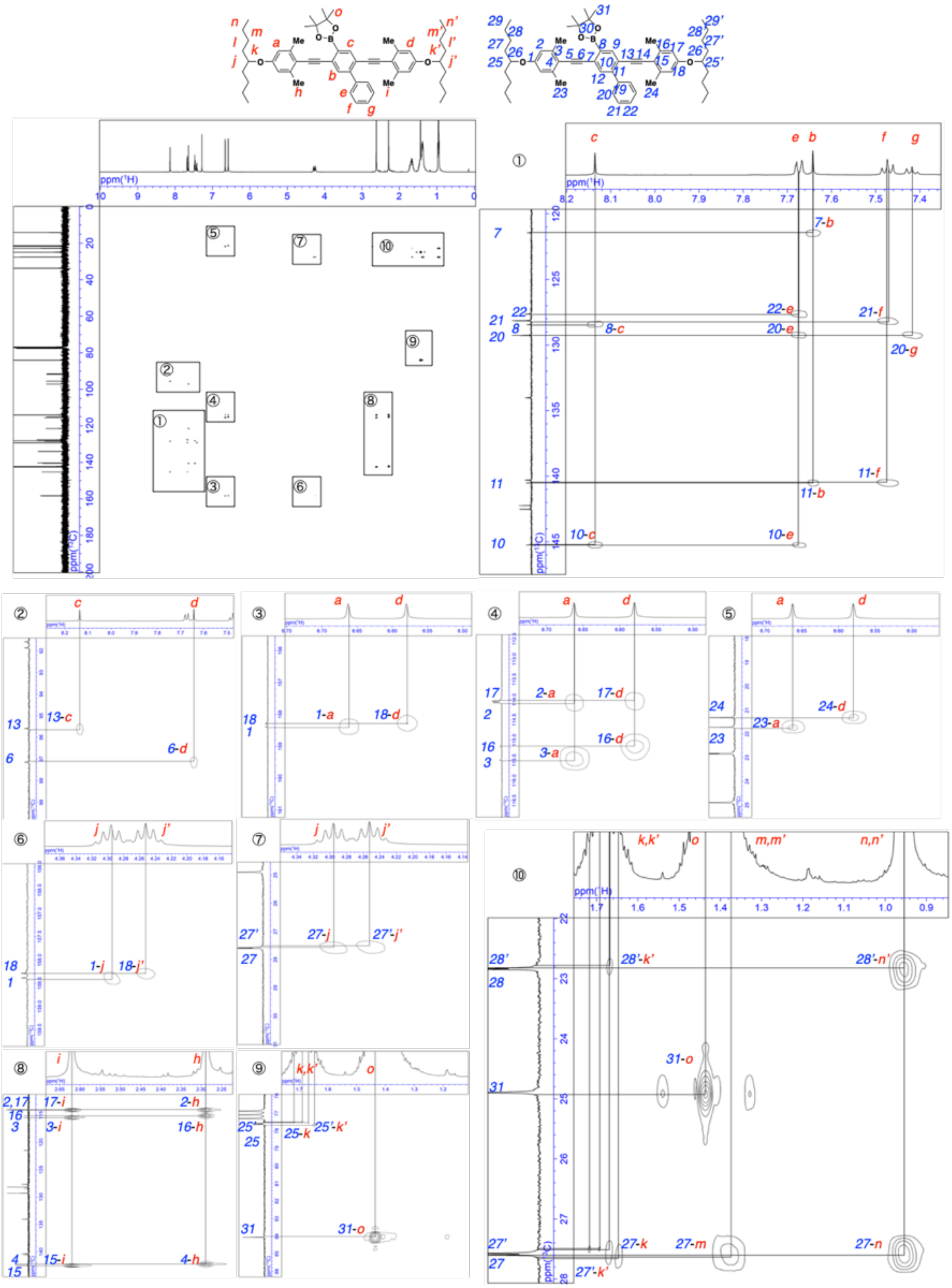

Supplementary Fig. 47 | HMBC correlation of Model-**2** (600 MHz for ^{1}H and 151 MHz for ^{13}C, $CDCl_3$, 25 ºC).

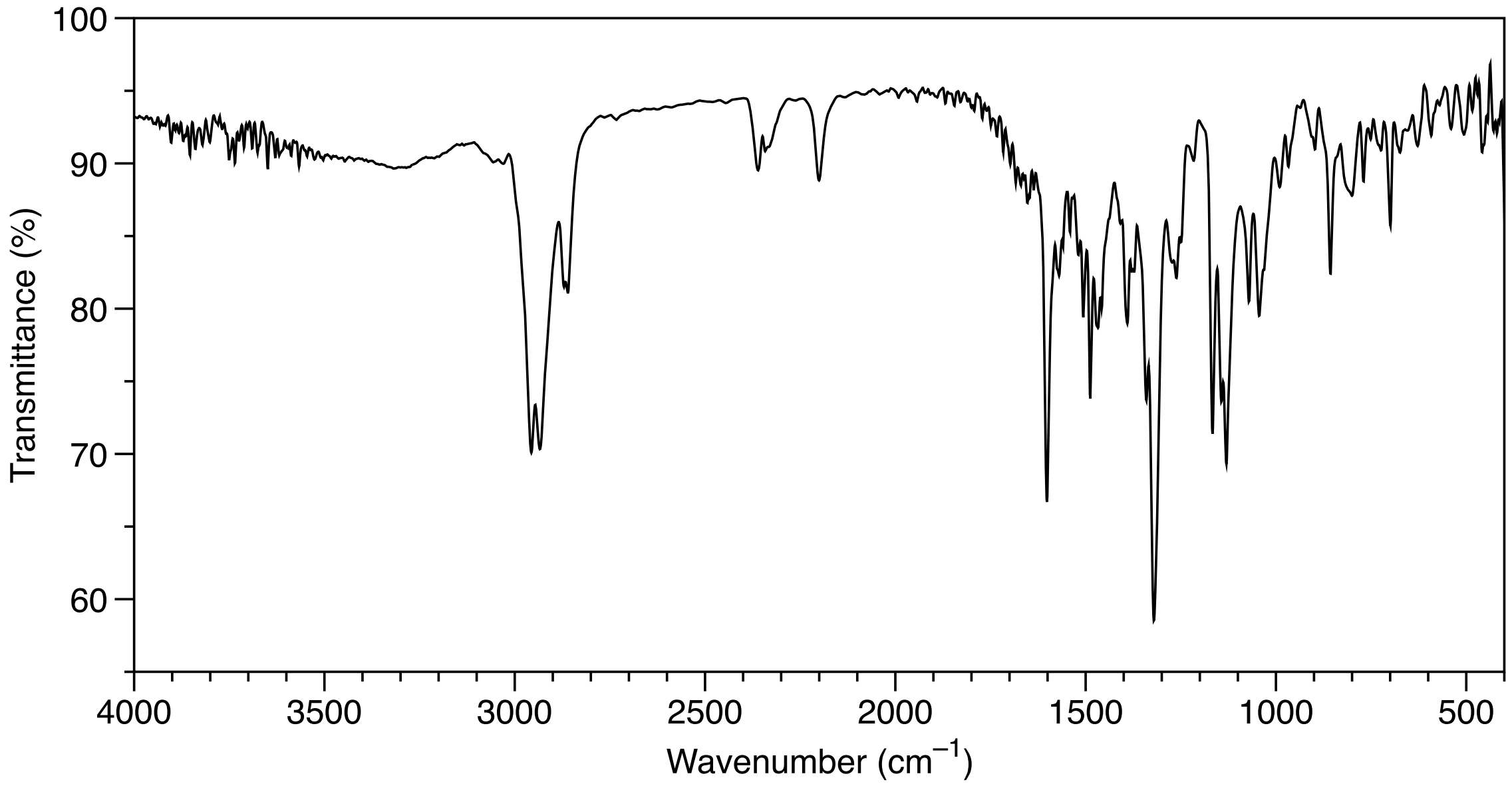

Supplementary Fig. 48 | FT-IR spectrum of Model-**2** (KBr, 25 °C).

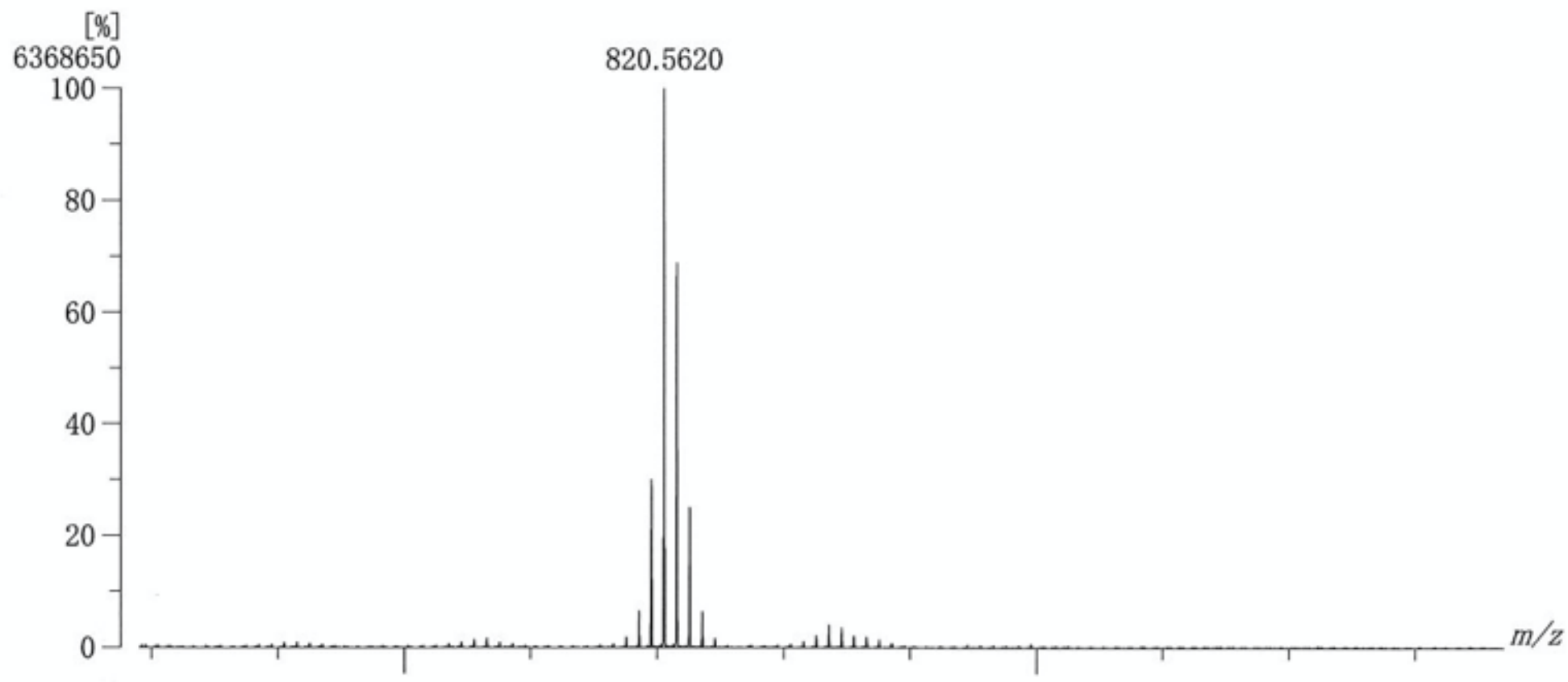

Supplementary Fig. 49 | High-resolution FAB-MS spectra of Model-**2**.

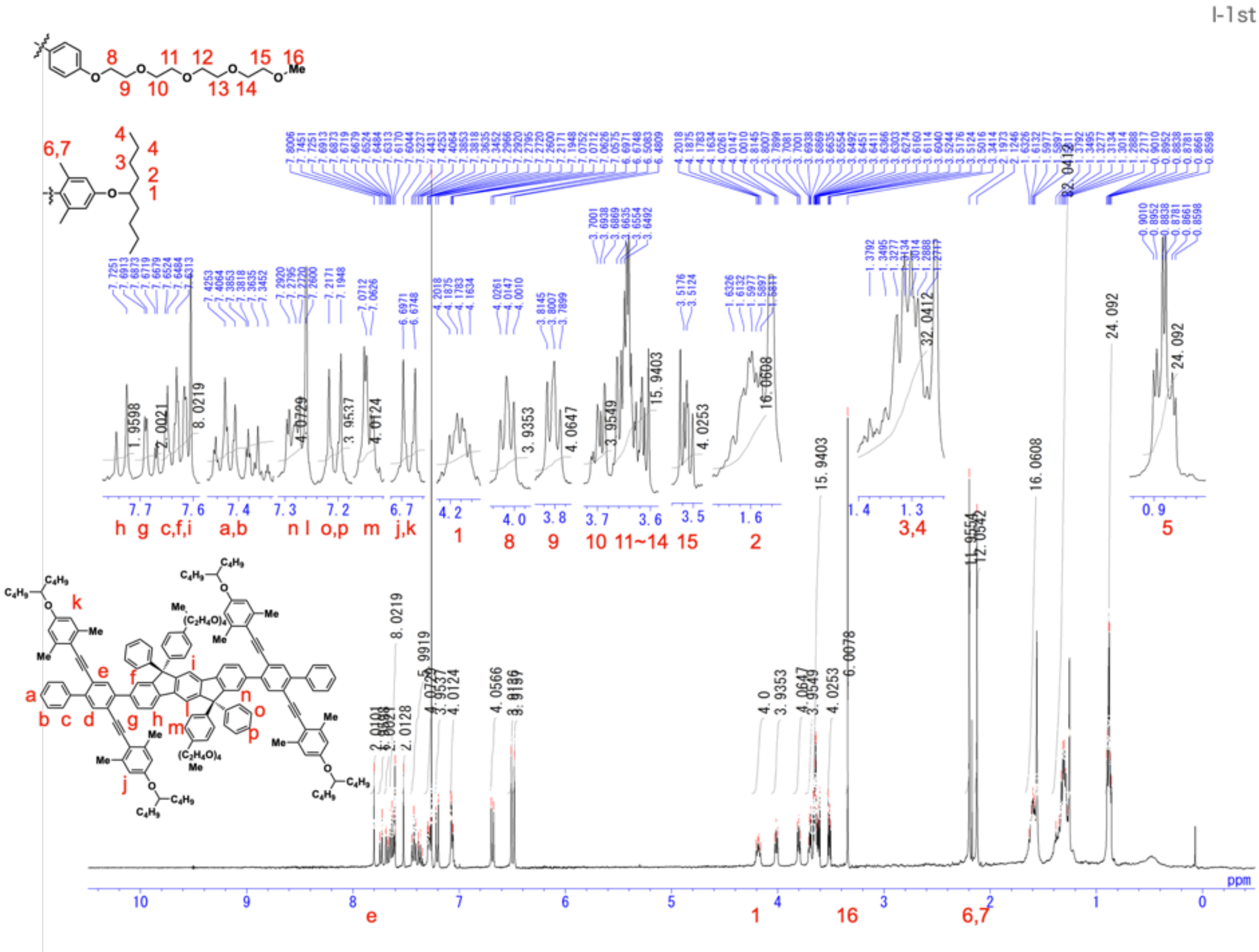

Supplementary Fig. 50 | ^{1}H NMR spectrum of Model-PP-(*S,S*)-**1** (600 MHz, $CDCl_3$, 25 ºC).

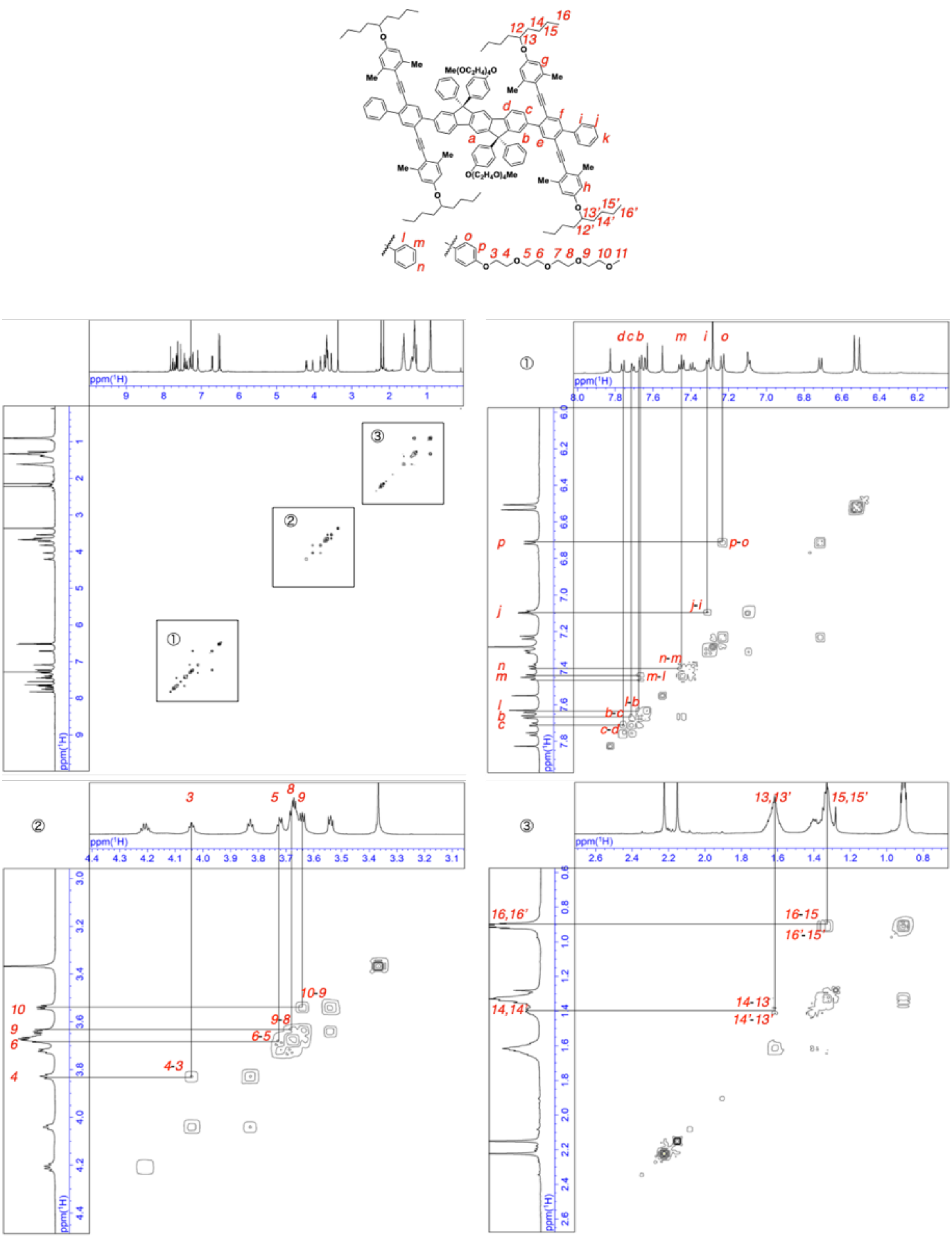

Supplementary Fig. 51 | HH-COSY correlation of Model-PP-(*S,S*)-**1** (600 MHz, $CDCl_3$, 25 ºC).

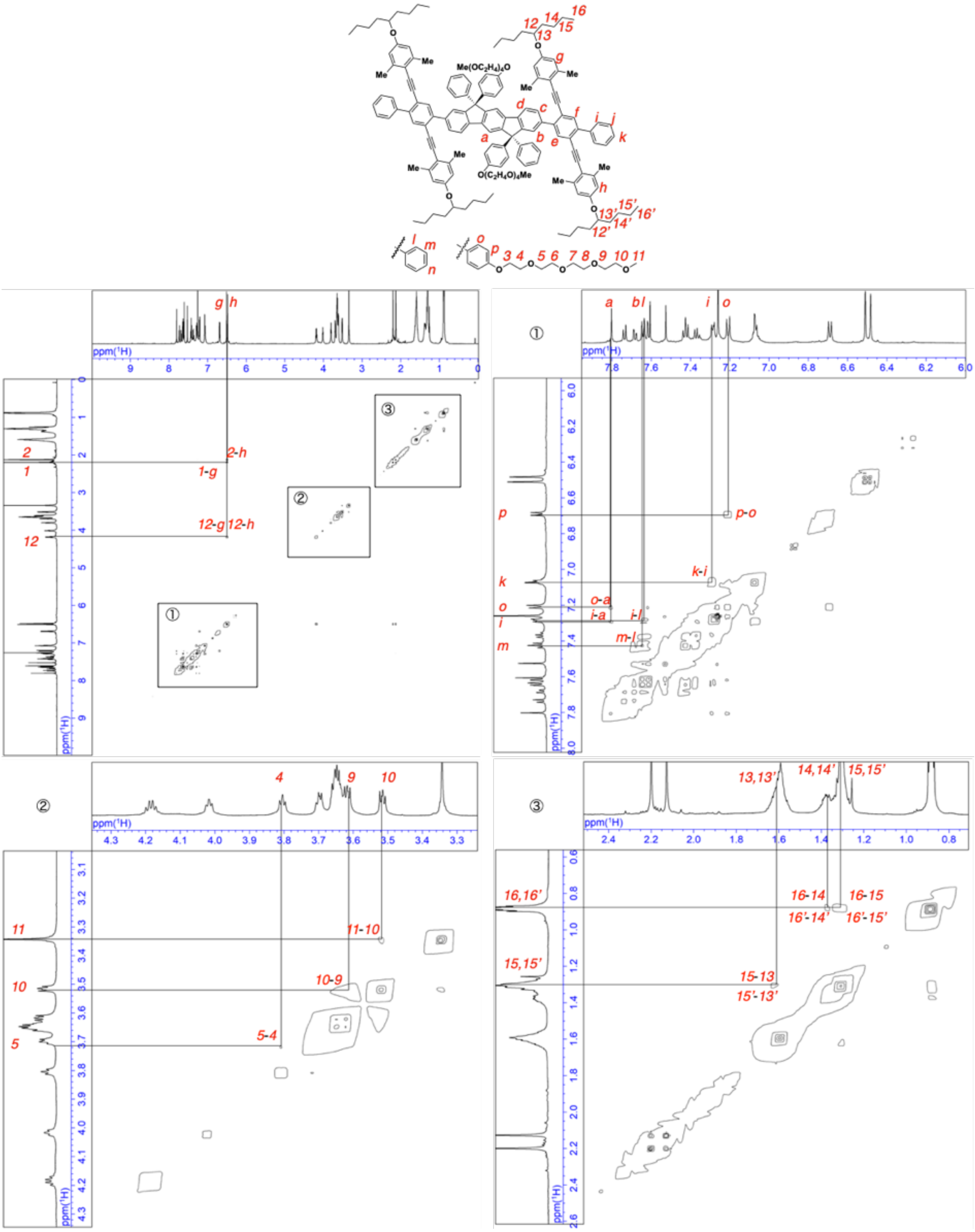

Supplementary Fig. 52 | NOESY correlation of Model-PP-(*S,S*)-**1** (600 MHz, $CDCl_3$, 25 ºC).

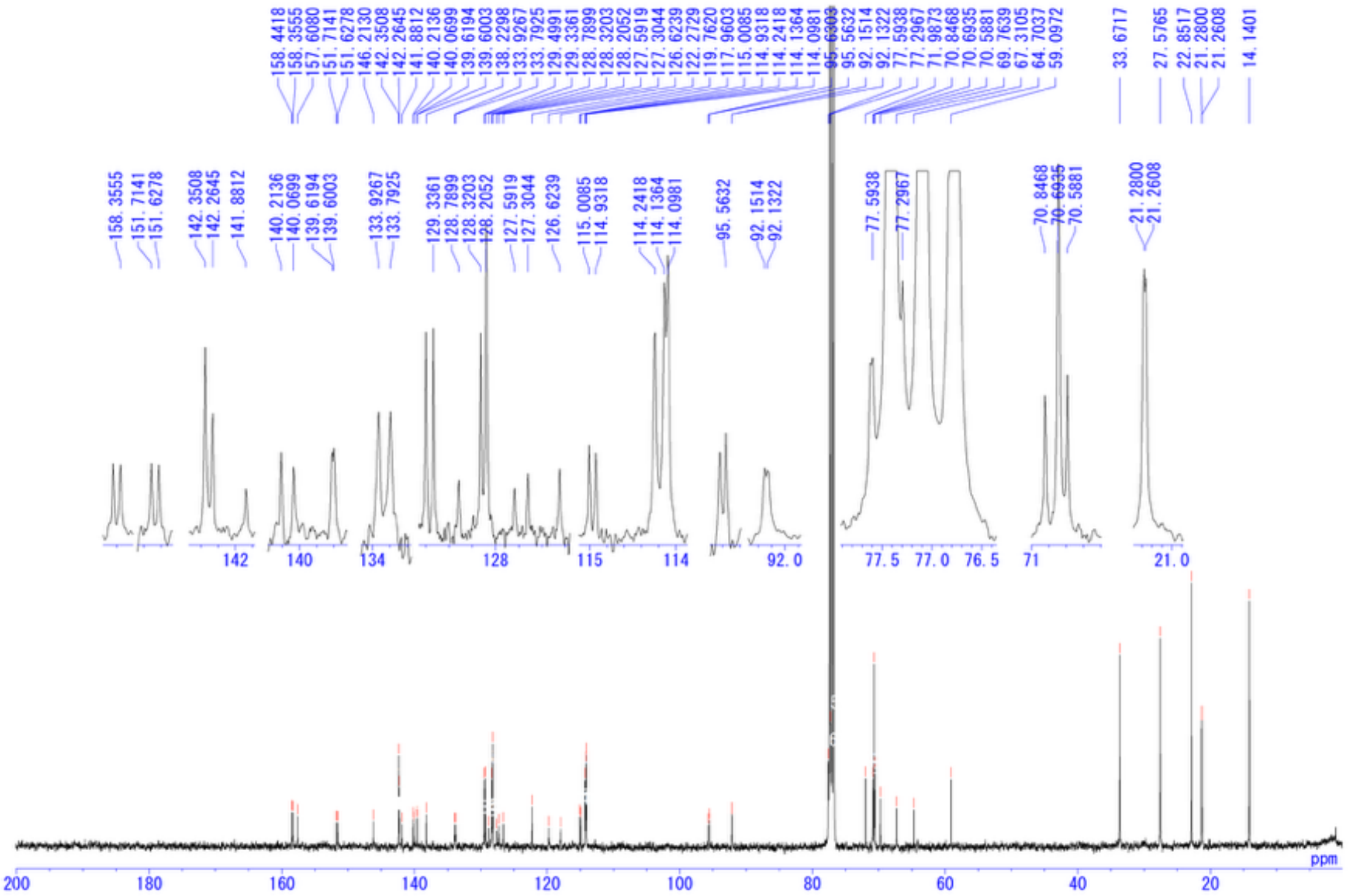

Supplementary Fig. 53 | ^{13}C NMR spectrum of Model-PP-(*S,S*)-**1** (151 MHz, $CDCl_3$, 25 ºC).

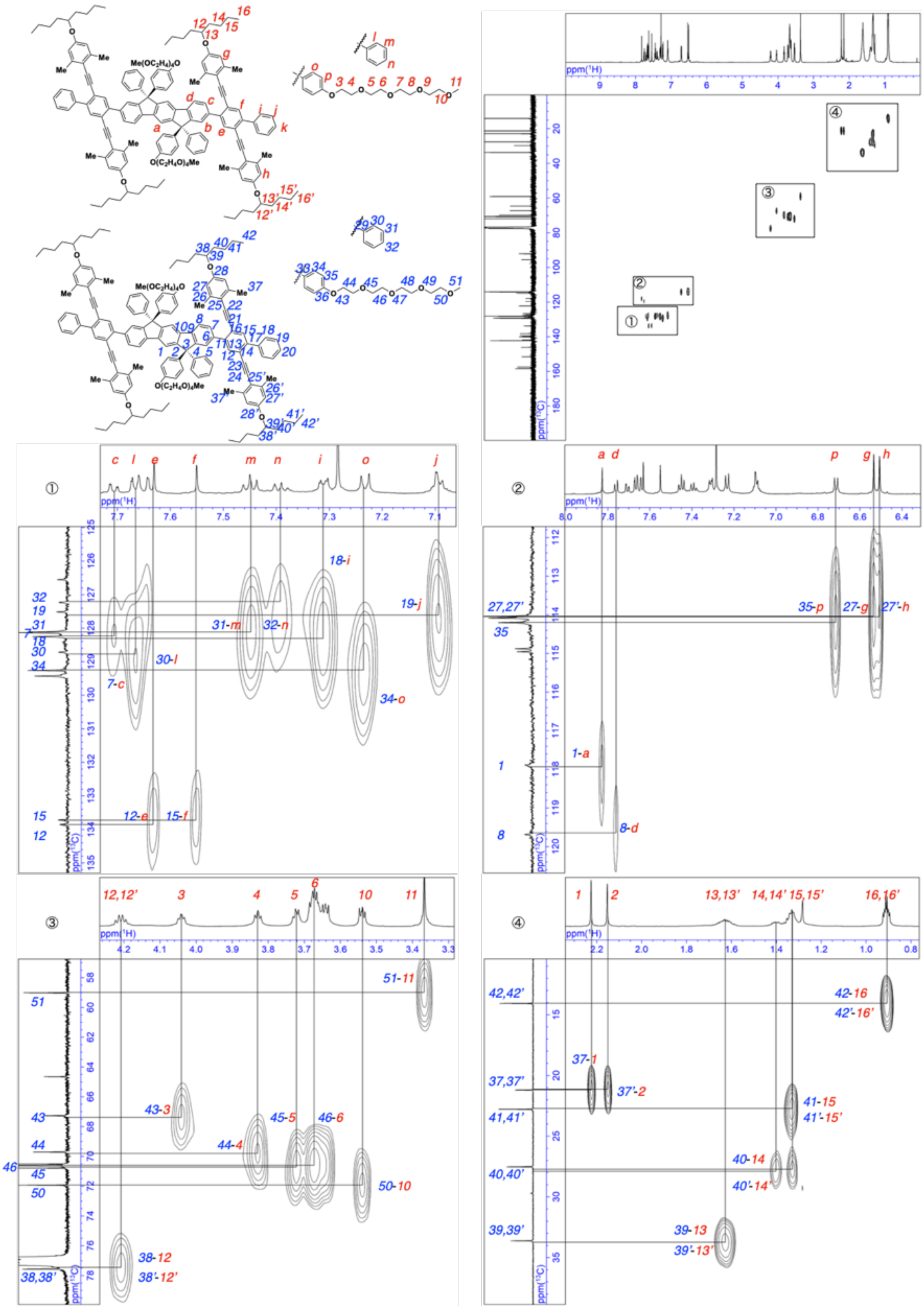

Supplementary Fig. 54 | HSQC correlation of Model-PP-(*S,S*)-**1** (600 MHz for ^{1}H and 151 MHz for ^{13}C, $CDCl_3$, 25 ºC).

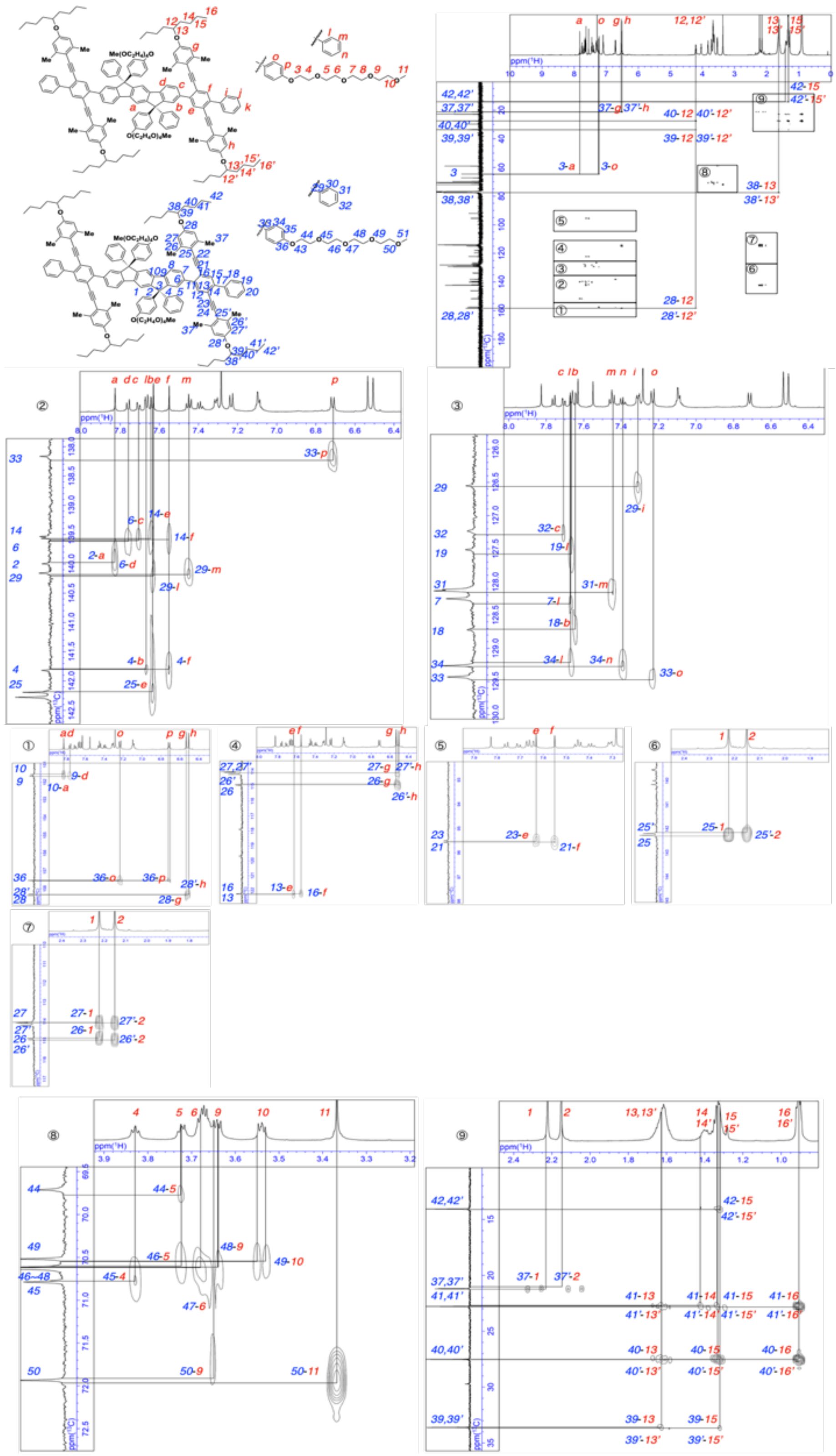

Supplementary Fig. 55 | HMBC correlation of Model-PP-(*S,S*)-**1** (600 MHz for ^{1}H and 151 MHz for ^{13}C, $CDCl_3$, 25 ºC).

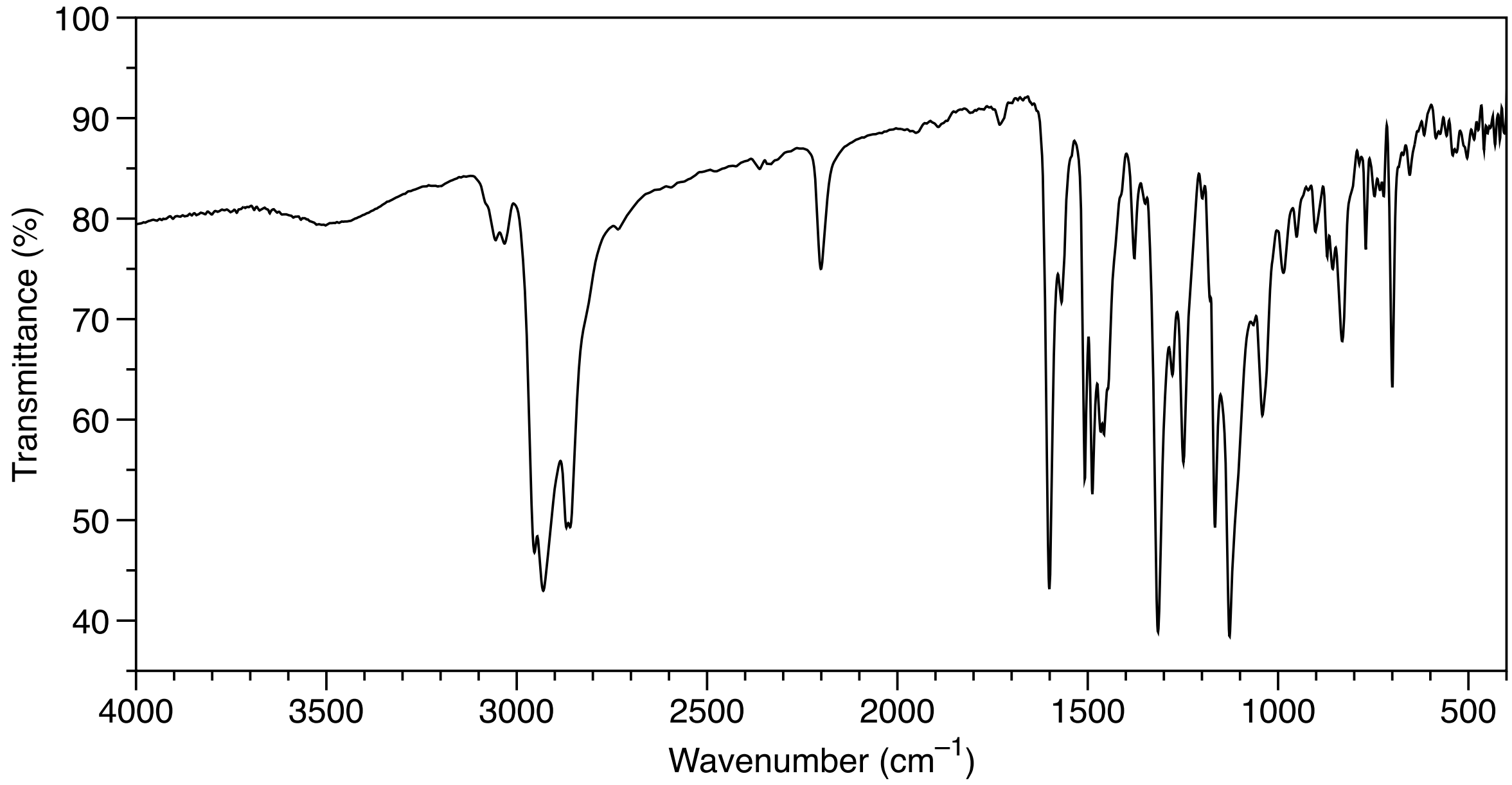

Supplementary Fig. 56 | FT-IR spectrum of Model-PP-(*S,S*)-**1** (KBr, 25 °C).

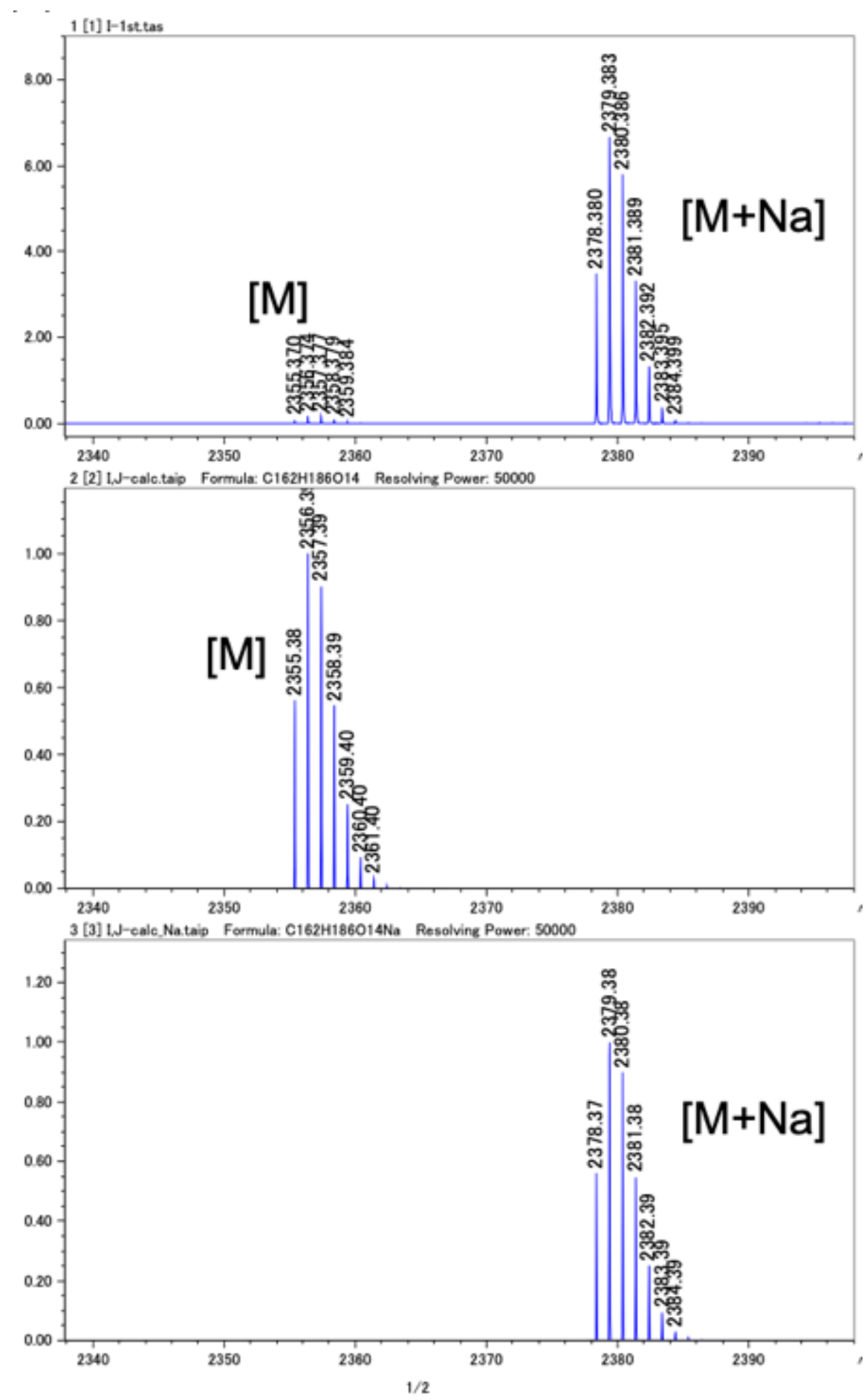

Supplementary Fig. 57 | Observed (upper) and simulated High-resolution MALDI-Spiral-TOF-MS spectrum of Model-PP-(*S,S*)-**1** for $[M]^+$ (middle) and $[M+Na]^+$ (lower).

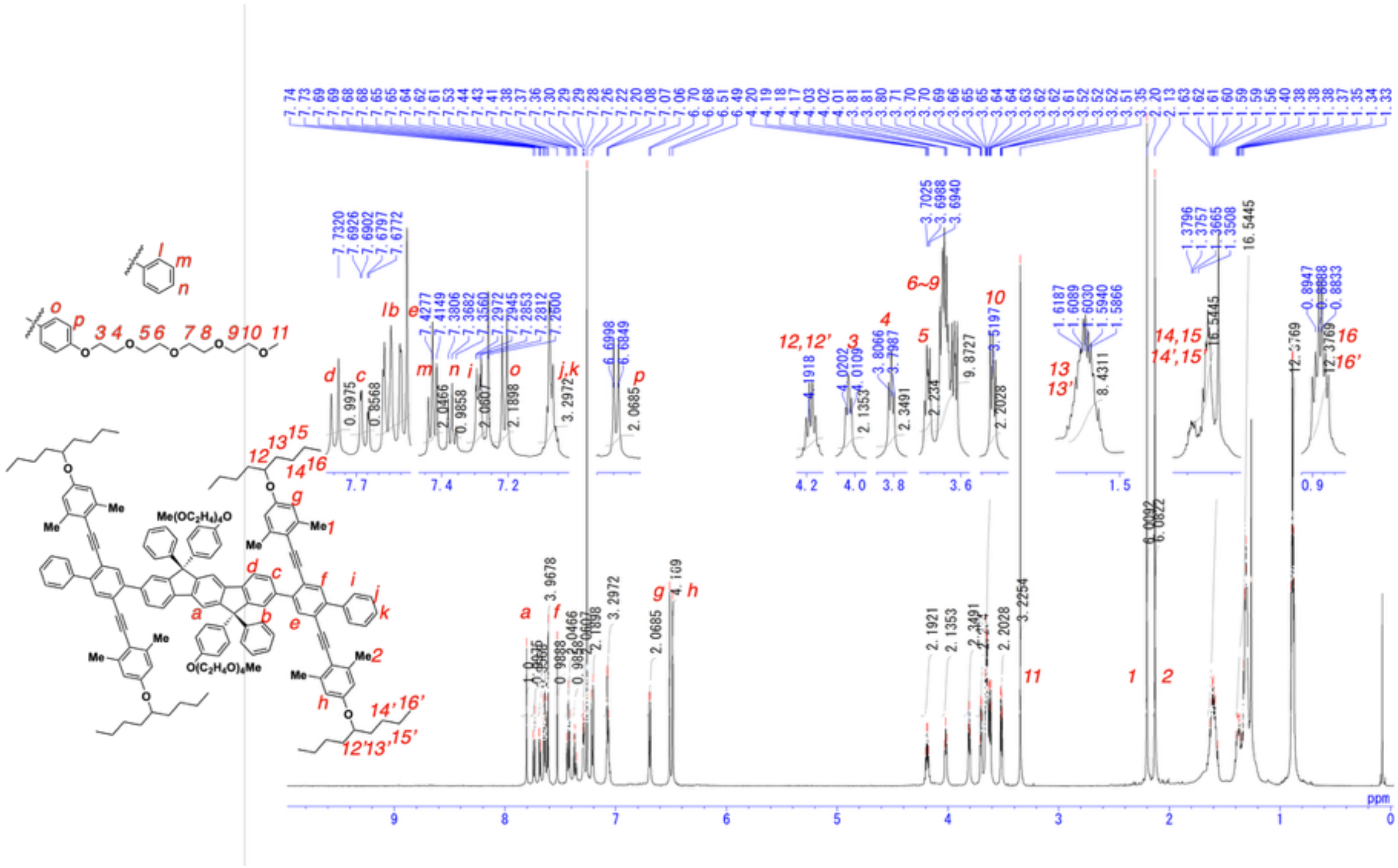

Supplementary Fig. 58 | ^{1}H NMR spectrum of Model-PP-(*R,R*)-**1** (600 MHz, $CDCl_3$, 25 ºC).

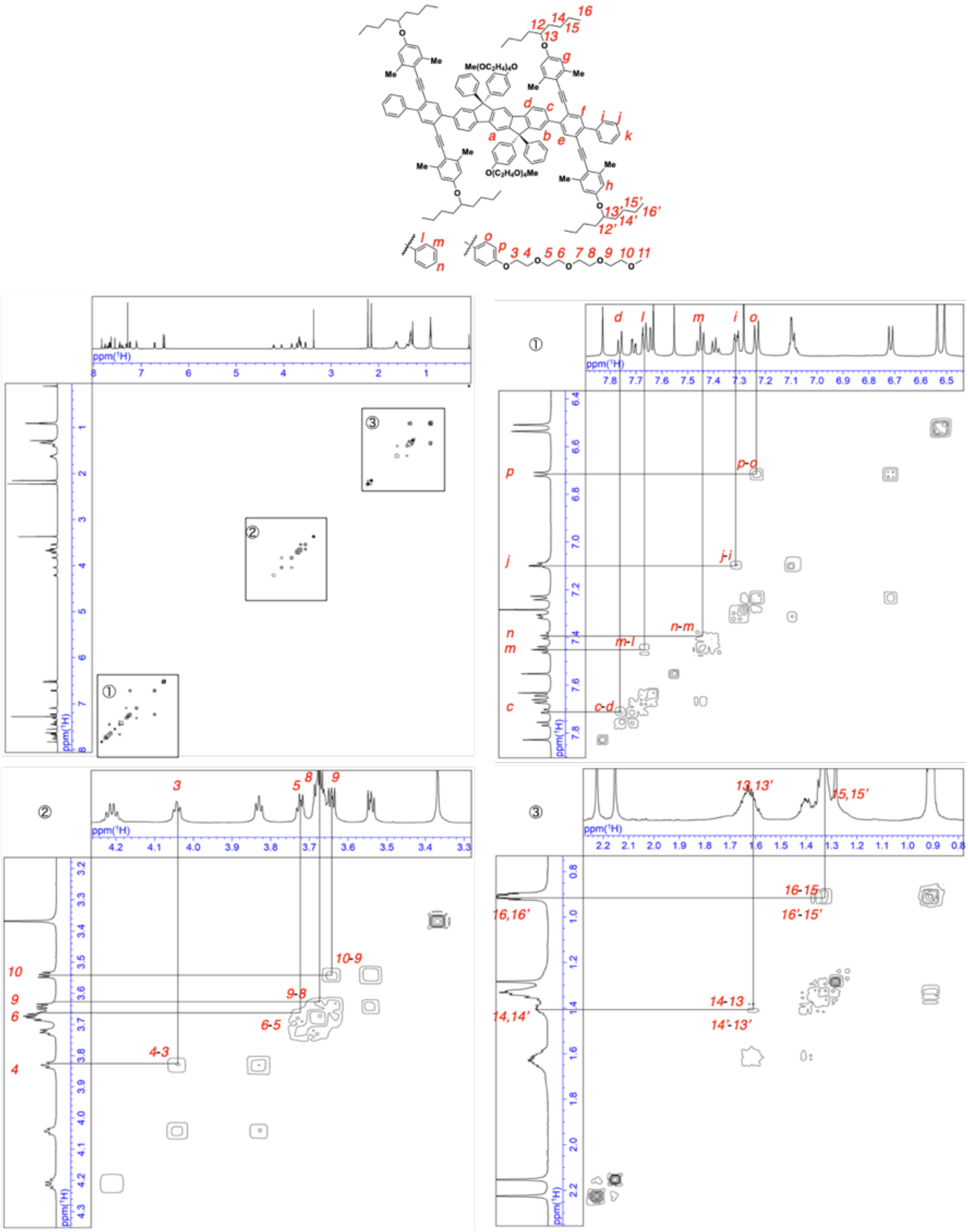

Supplementary Fig. 59 | HH-COSY correlation of Model-PP-(*R,R*)-**1** (600 MHz, $CDCl_3$, 25 ºC).

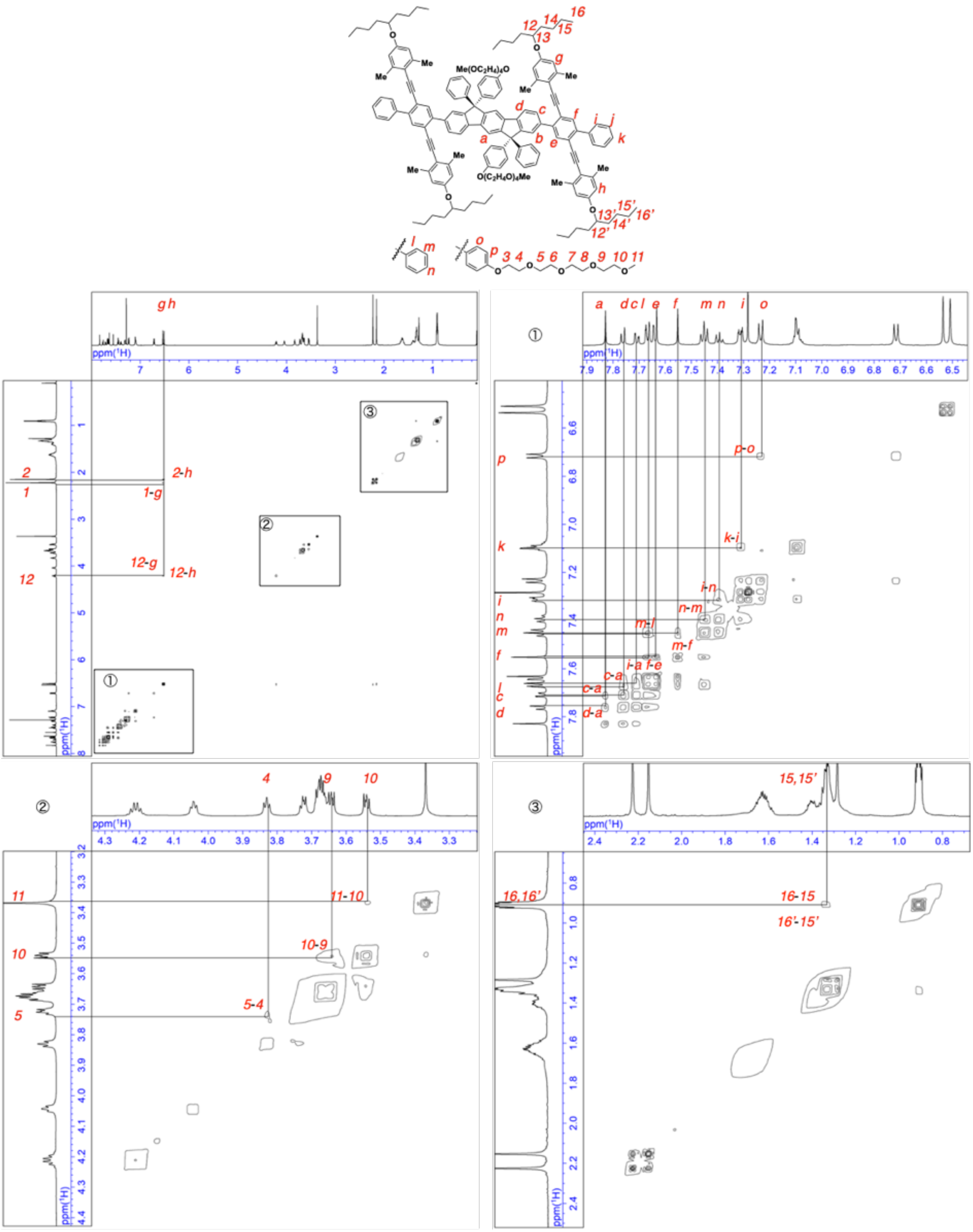

Supplementary Fig. 60 | NOESY correlation of Model-PP-(*R*,*R*)-**1** (600 MHz, $CDCl_3$, 25 ºC).

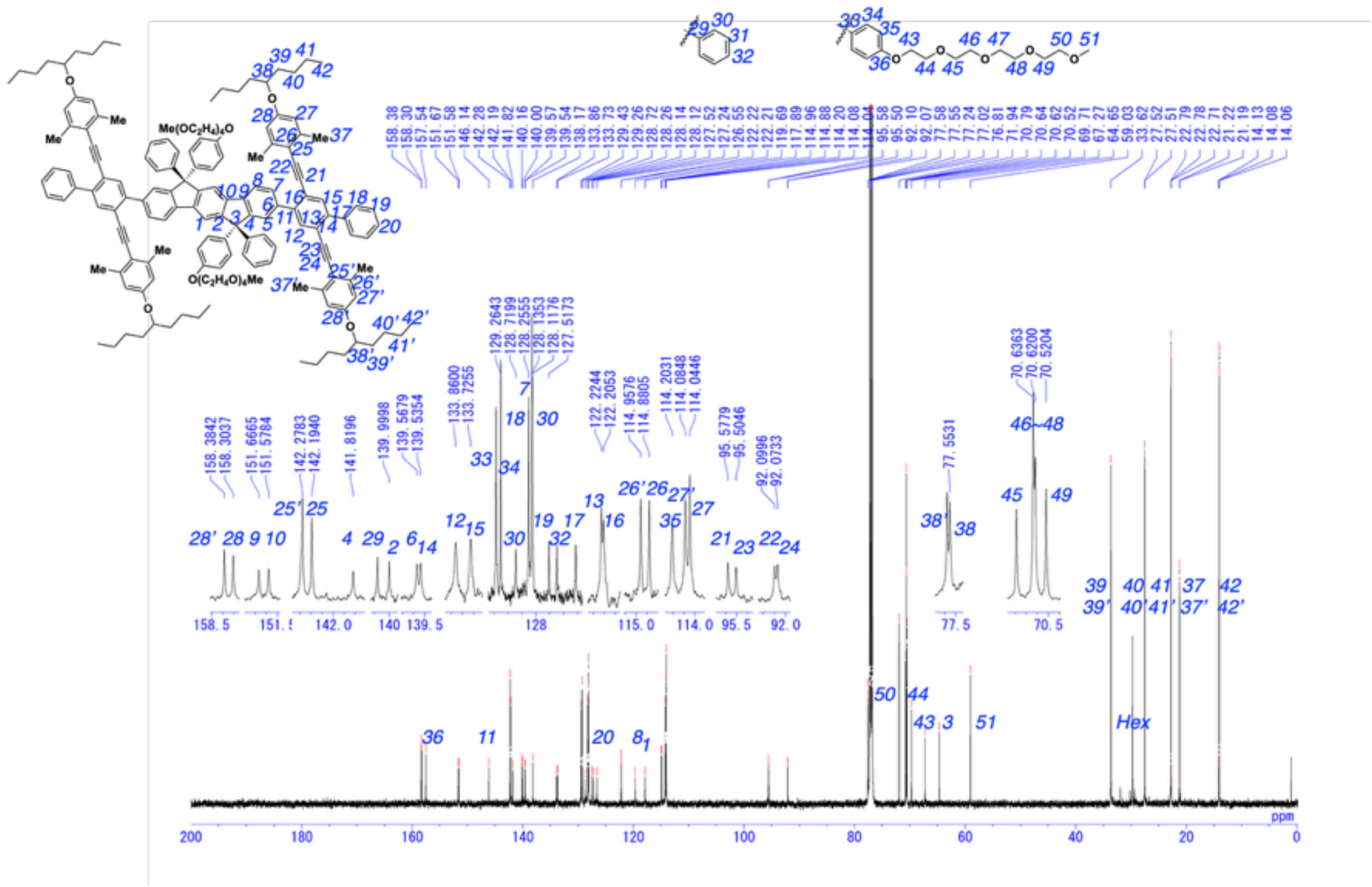

Supplementary Fig. 61 | ^{13}C NMR spectrum of Model-PP-(*R*,*R*)-**1** (151 MHz, $CDCl_3$, 25 ºC).

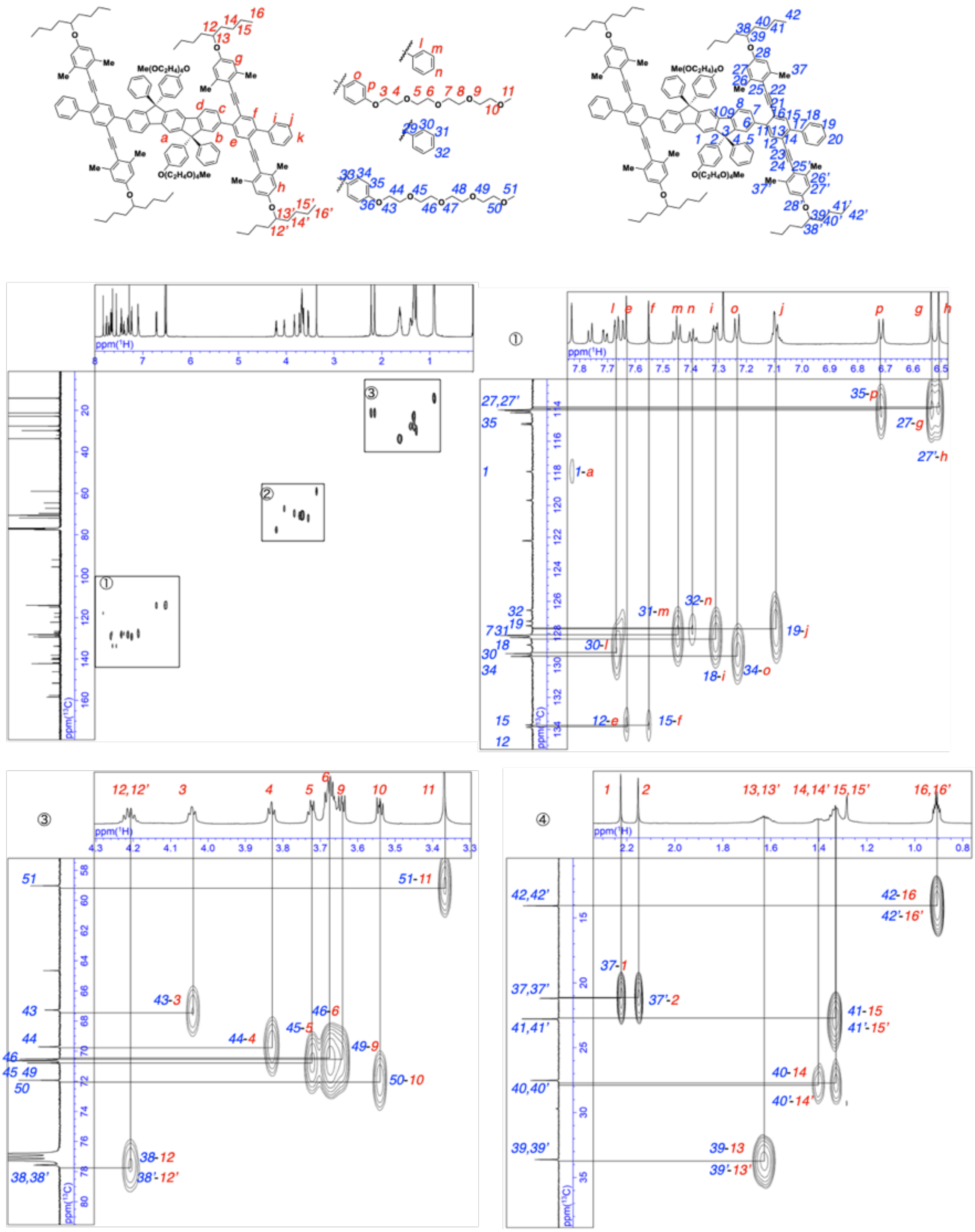

Supplementary Fig. 62 | HSQC correlation of Model-PP-(*R,R*)-**1** (600 MHz for ^{1}H and 151 MHz for ^{13}C, $CDCl_3$, 25 ºC).

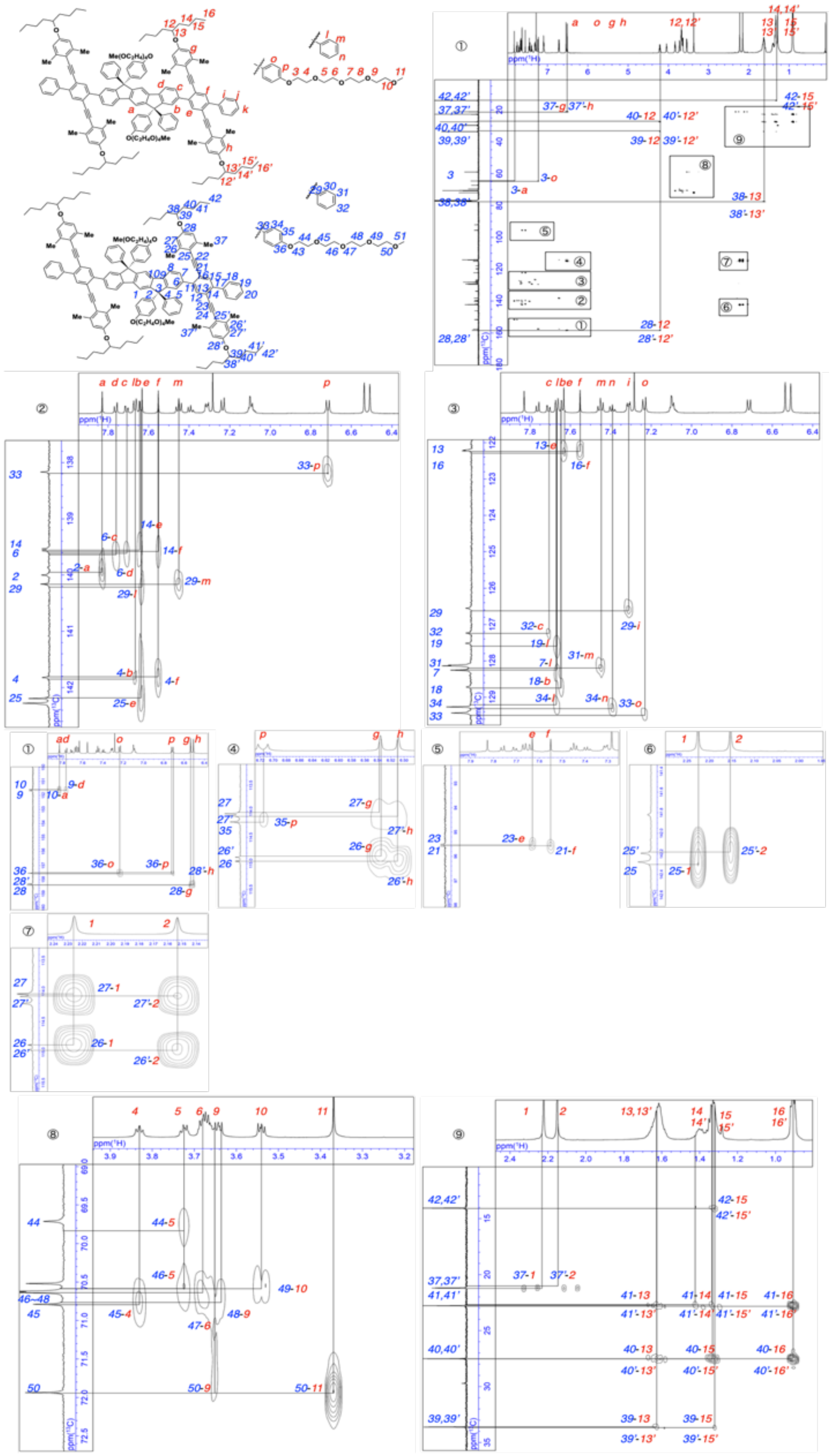

Supplementary Fig. 63 | HMBC correlation of Model-PP-(*R*,*R*)-**1** (600 MHz for ^{1}H and 151 MHz for ^{13}C, $CDCl_3$, 25 ºC).

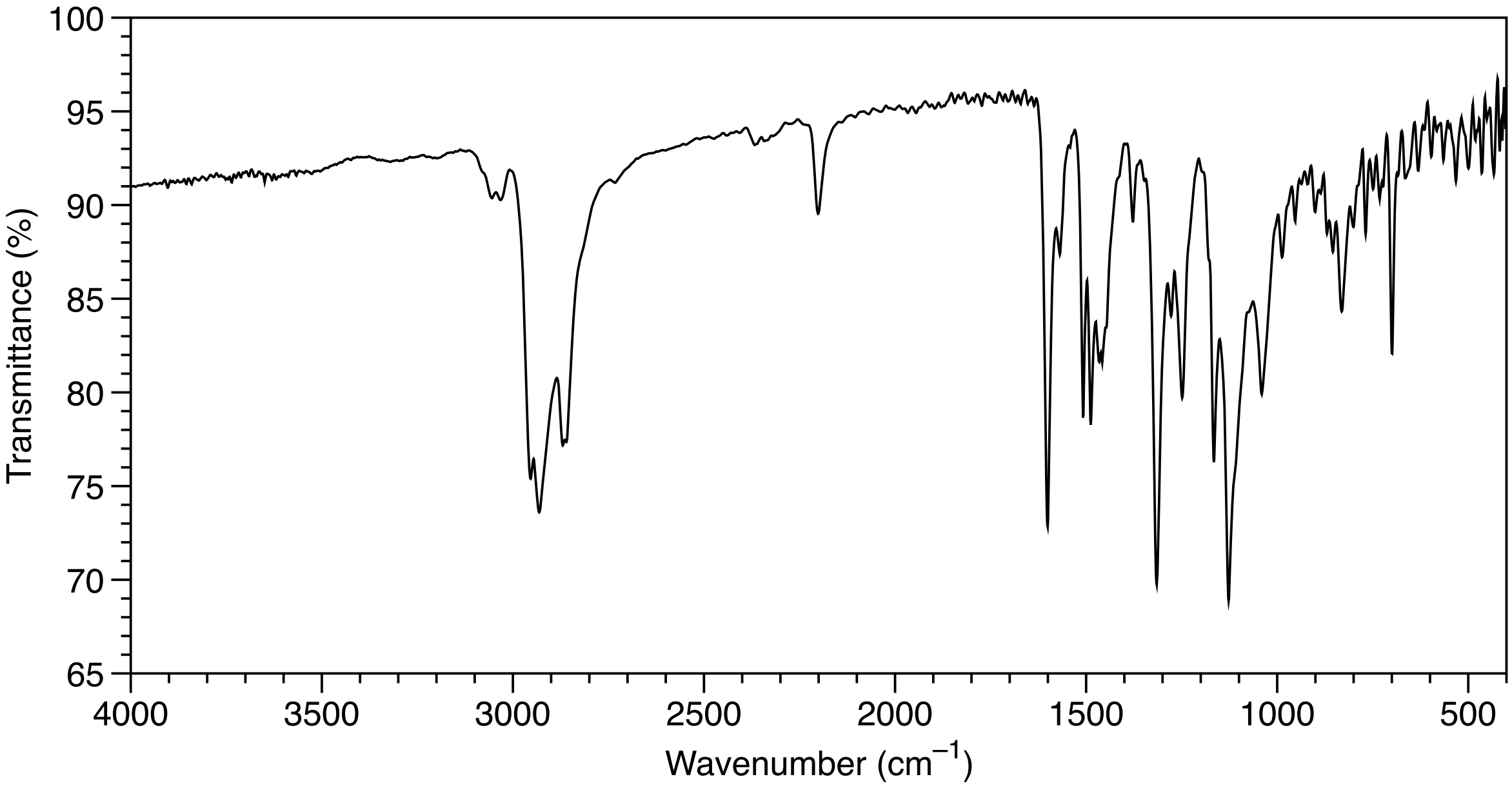

Supplementary Fig. 64 | FT-IR spectrum of Model-PP-(*R,R*)-**1** (KBr, 25 °C).

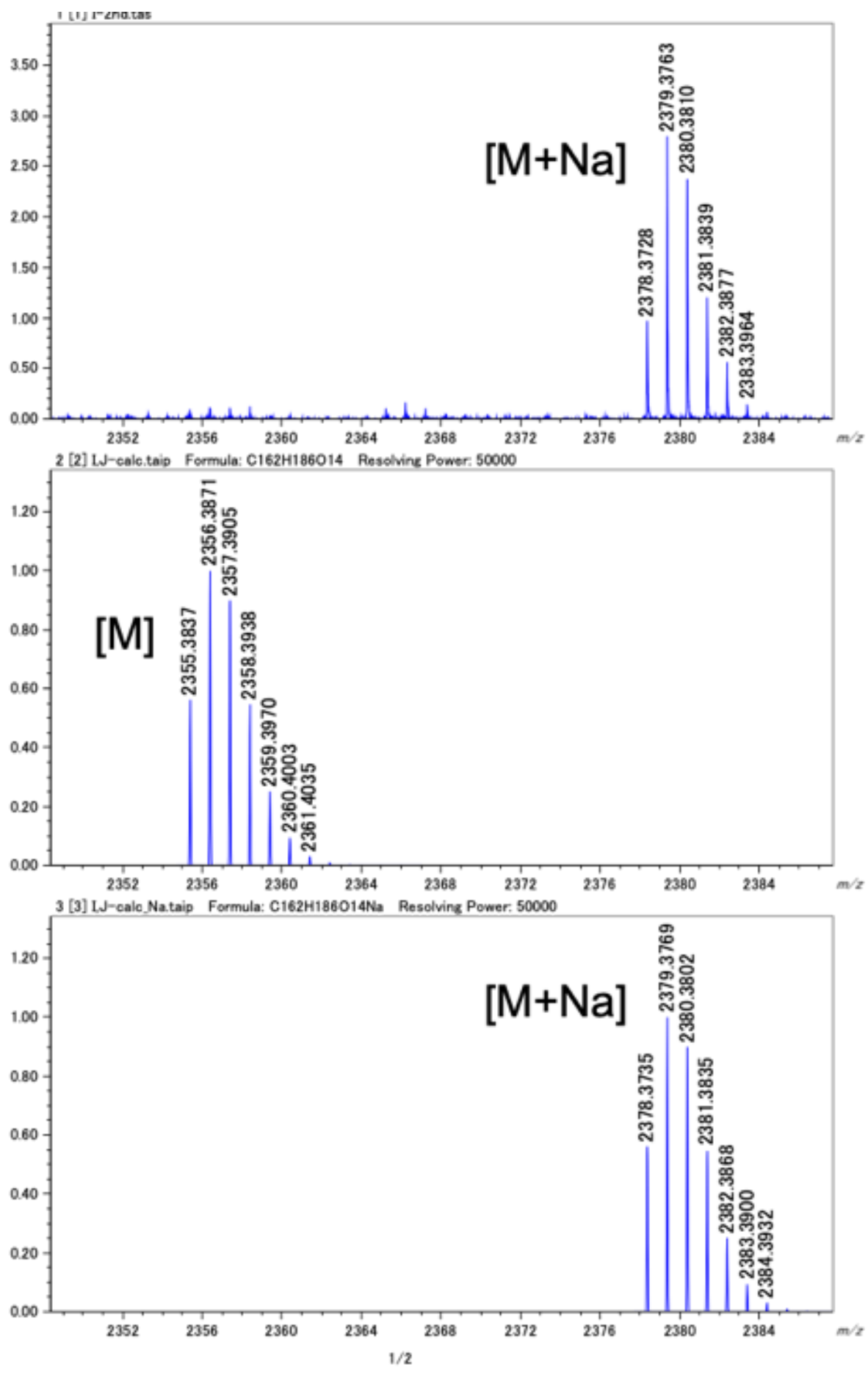

Supplementary Fig. 65 | Observed (upper) and simulated high-resolution MALDI-Spiral-TOF-MS spectra of Model-PP-(*R,R*)-**1** for $[M]^+$ (middle) and $[M+Na]^+$ (lower).

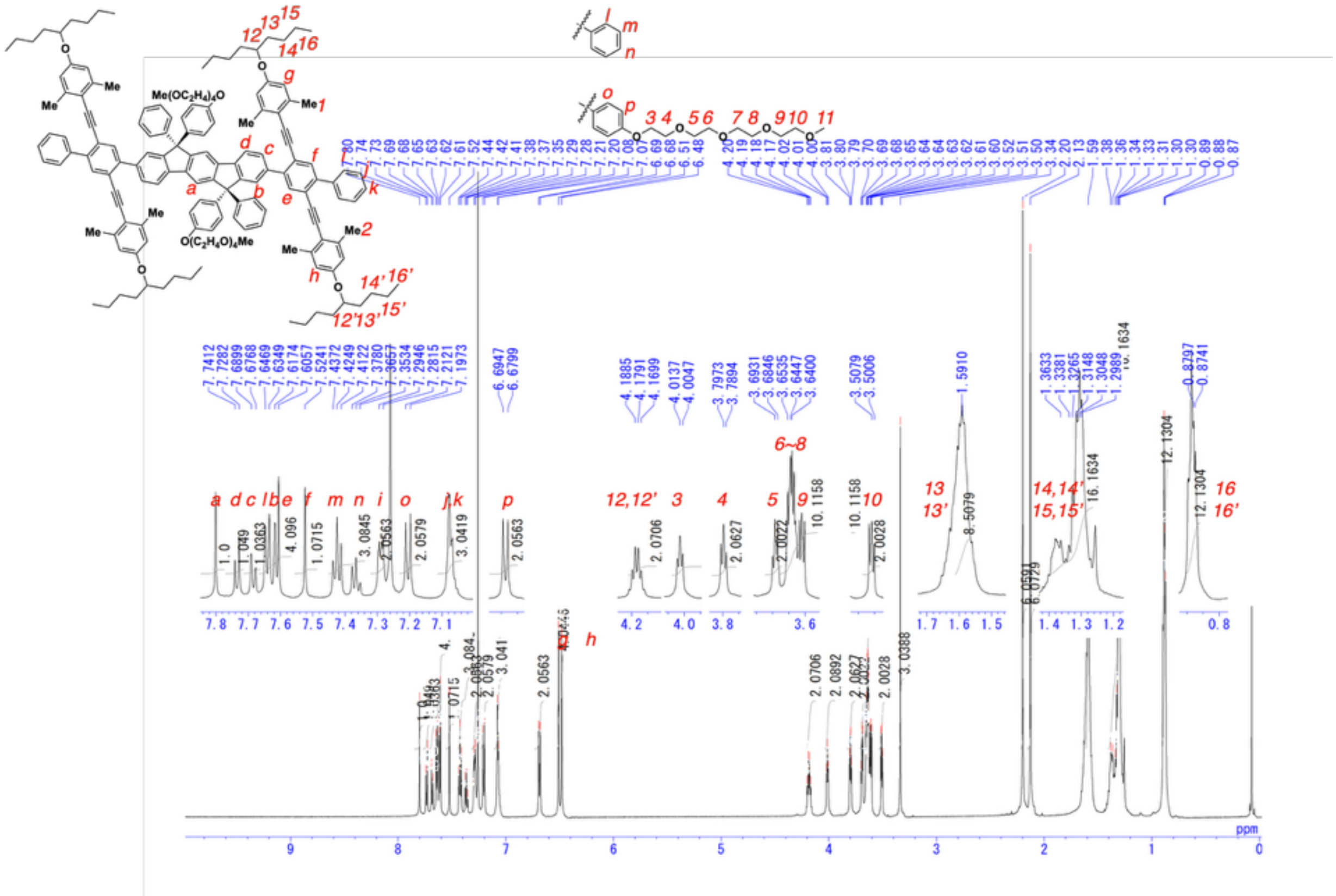

Supplementary Fig. 66 | ^{1}H NMR spectrum of Model-PP-*meso*-**1** (600 MHz, $CDCl_3$, 25 ºC).

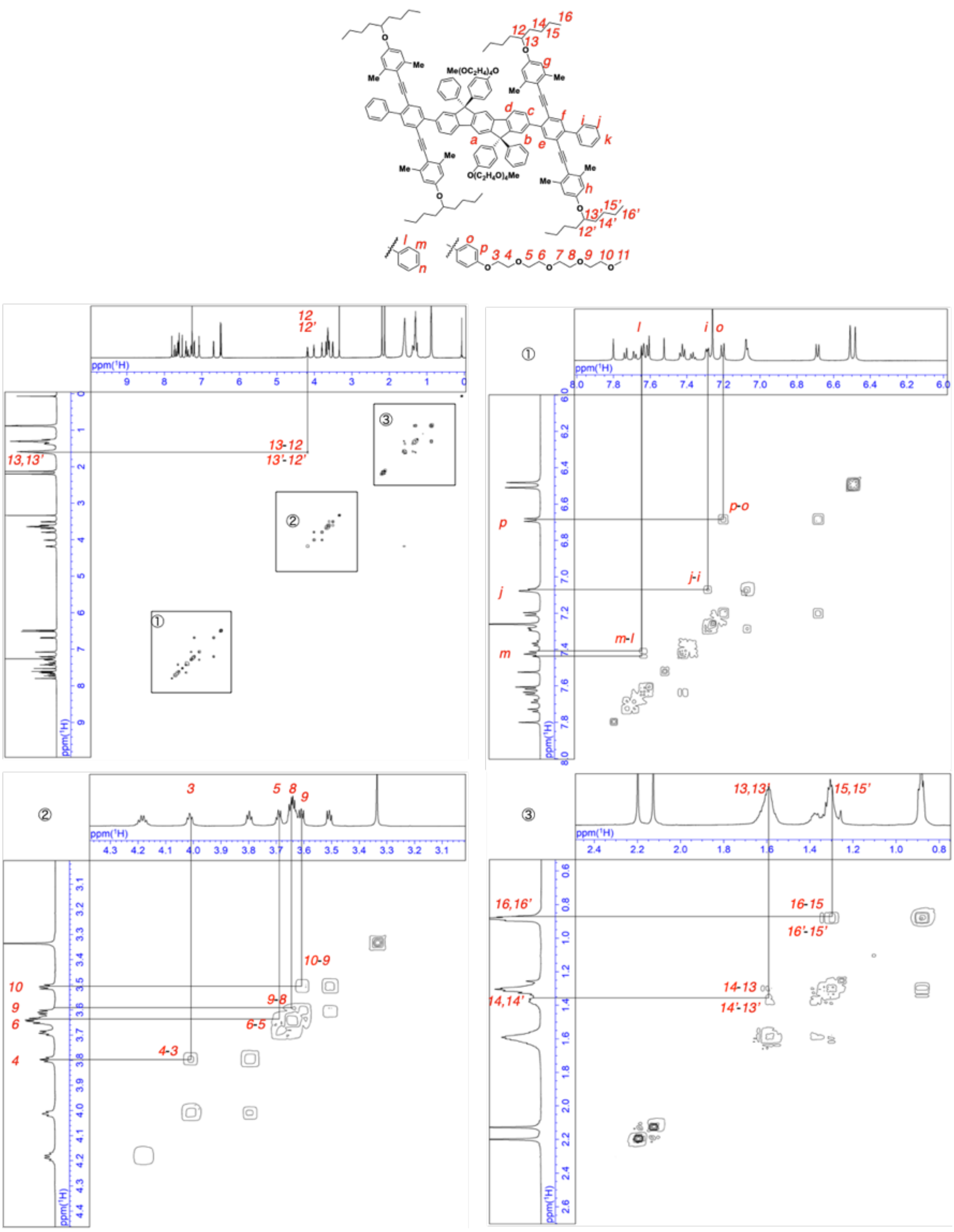

Supplementary Fig. 67 | HH-COSY correlation of Model-PP-*meso*-**1** (600 MHz, $CDCl_3$, 25 ºC).

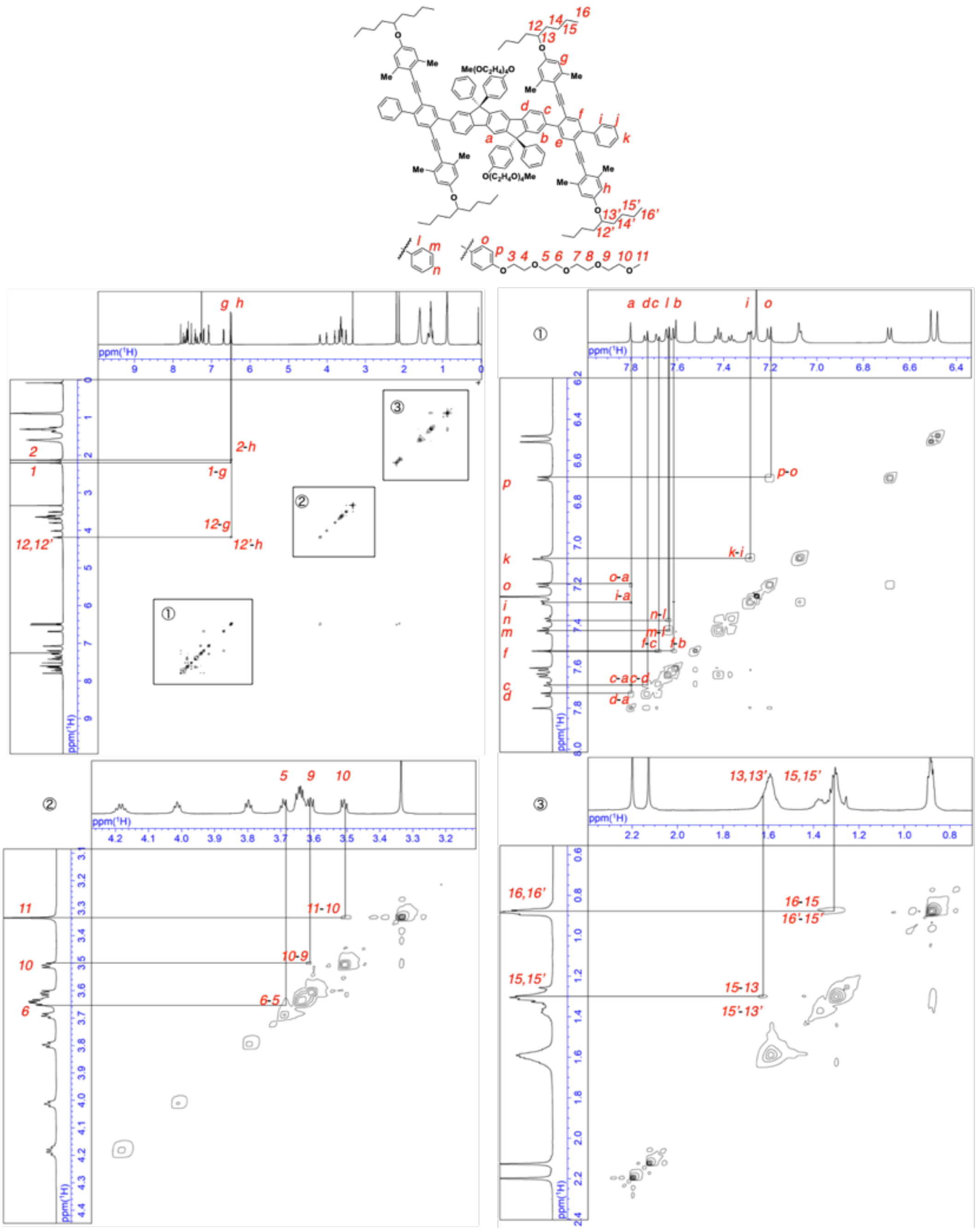

Supplementary Fig. 68 | NOESY correlation of Model-PP-*meso*-**1** (600 MHz, $CDCl_3$, 25 ºC).

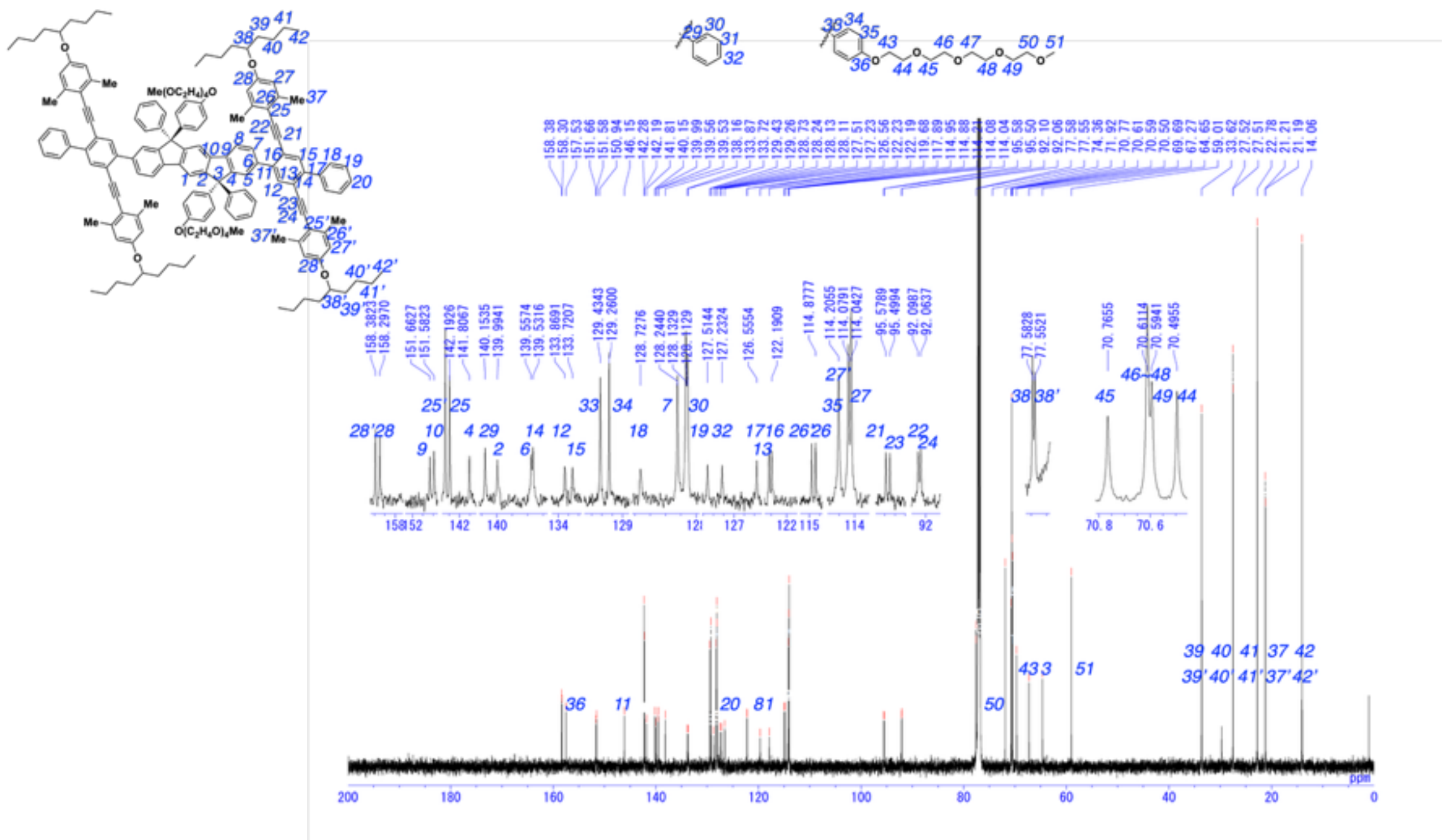

Supplementary Fig. 69 | ^{13}C NMR spectrum of Model-PP-*meso*-**1** (151 MHz, $CDCl_3$, 25 ºC).

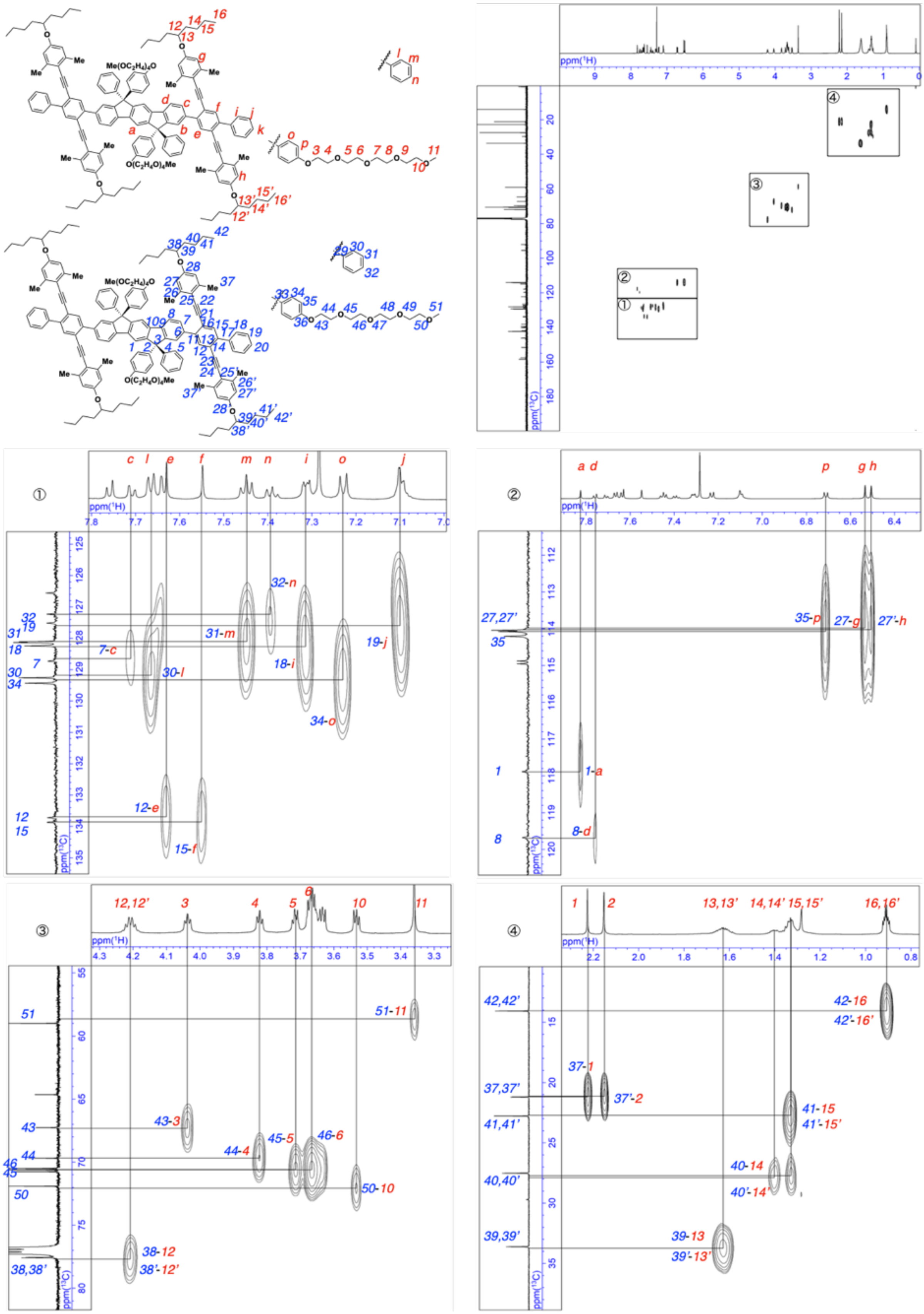

Supplementary Fig. 70 | HSQC correlation of Model-PP-*meso*-**1** (600 MHz for ^{1}H and 151 MHz for ^{13}C, $CDCl_3$, 25 ºC).

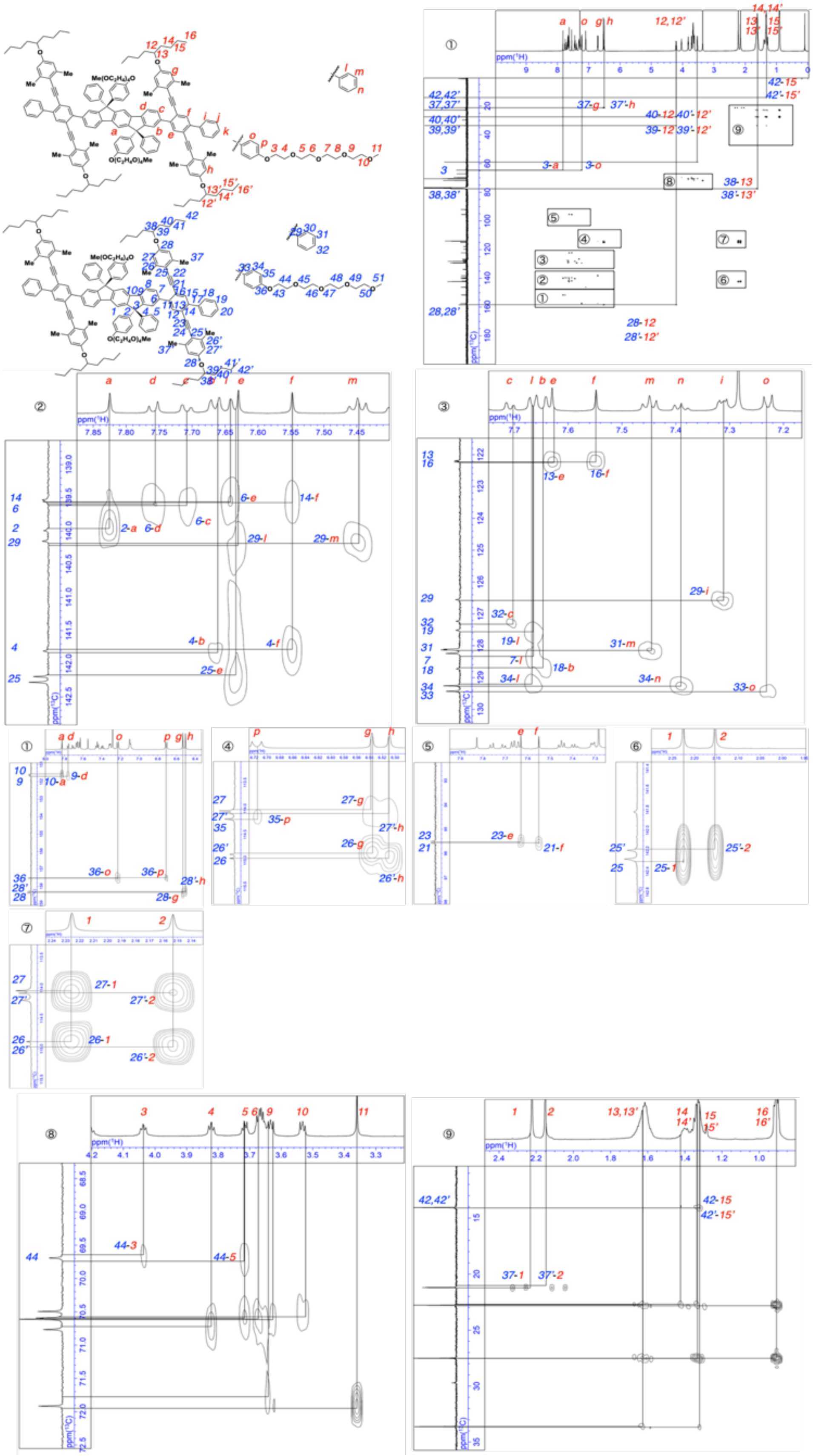

Supplementary Fig. 71 | HMBC correlation of Model-PP-*meso*-**1** (600 MHz for ^{1}H and 151 MHz for ^{13}C, $CDCl_3$, 25 ºC).

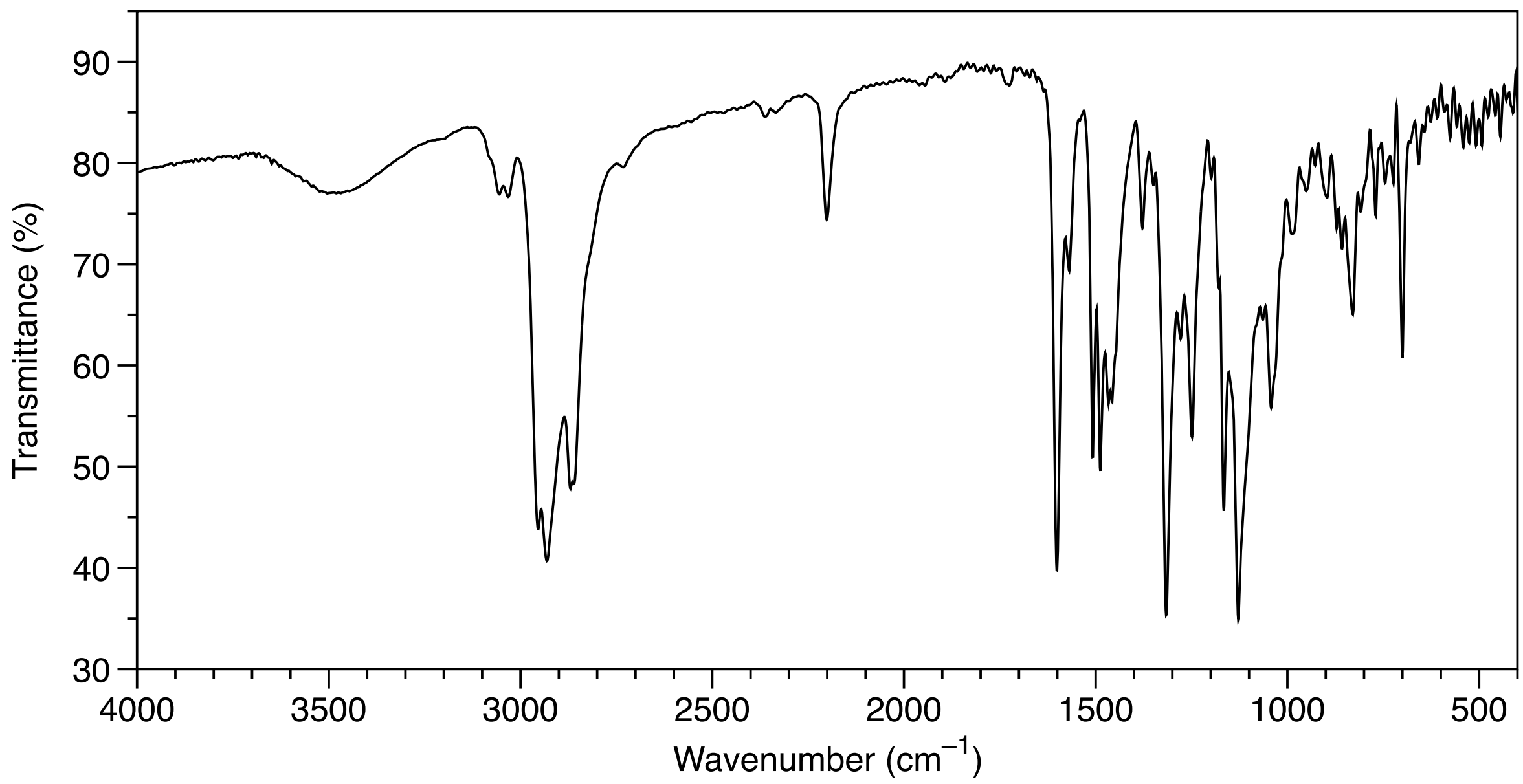

Supplementary Fig. 72 | FT-IR spectrum of Model-PP-*meso*-**1** (KBr, 25 °C).

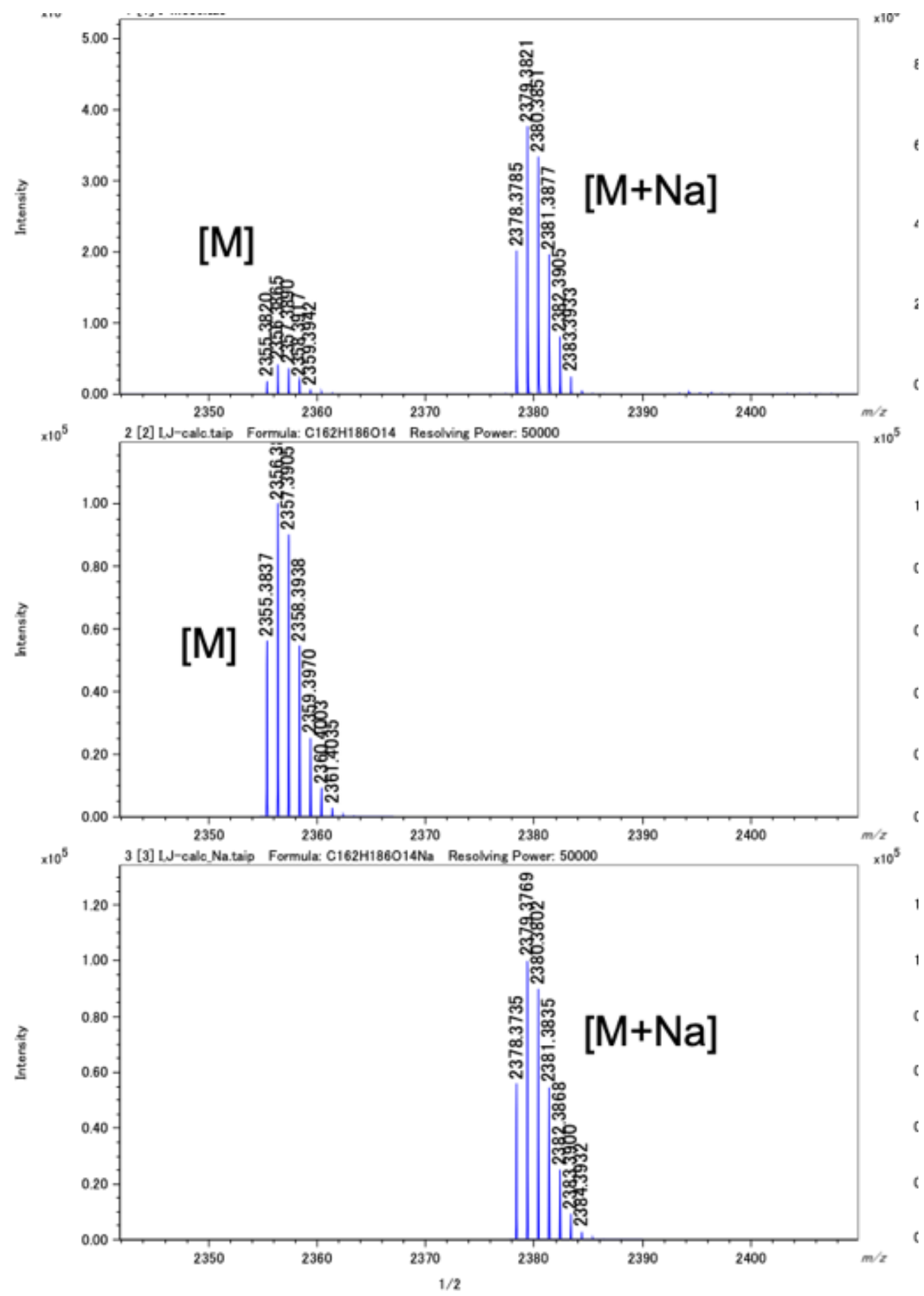

Supplementary Fig. 73 | Observed (upper) and simulated high-resolution MALDI-Spiral-TOF-MS spectra of Model-PP-*meso*-**1** for $[M]^+$ (middle) and $[M+Na]^+$ (lower).

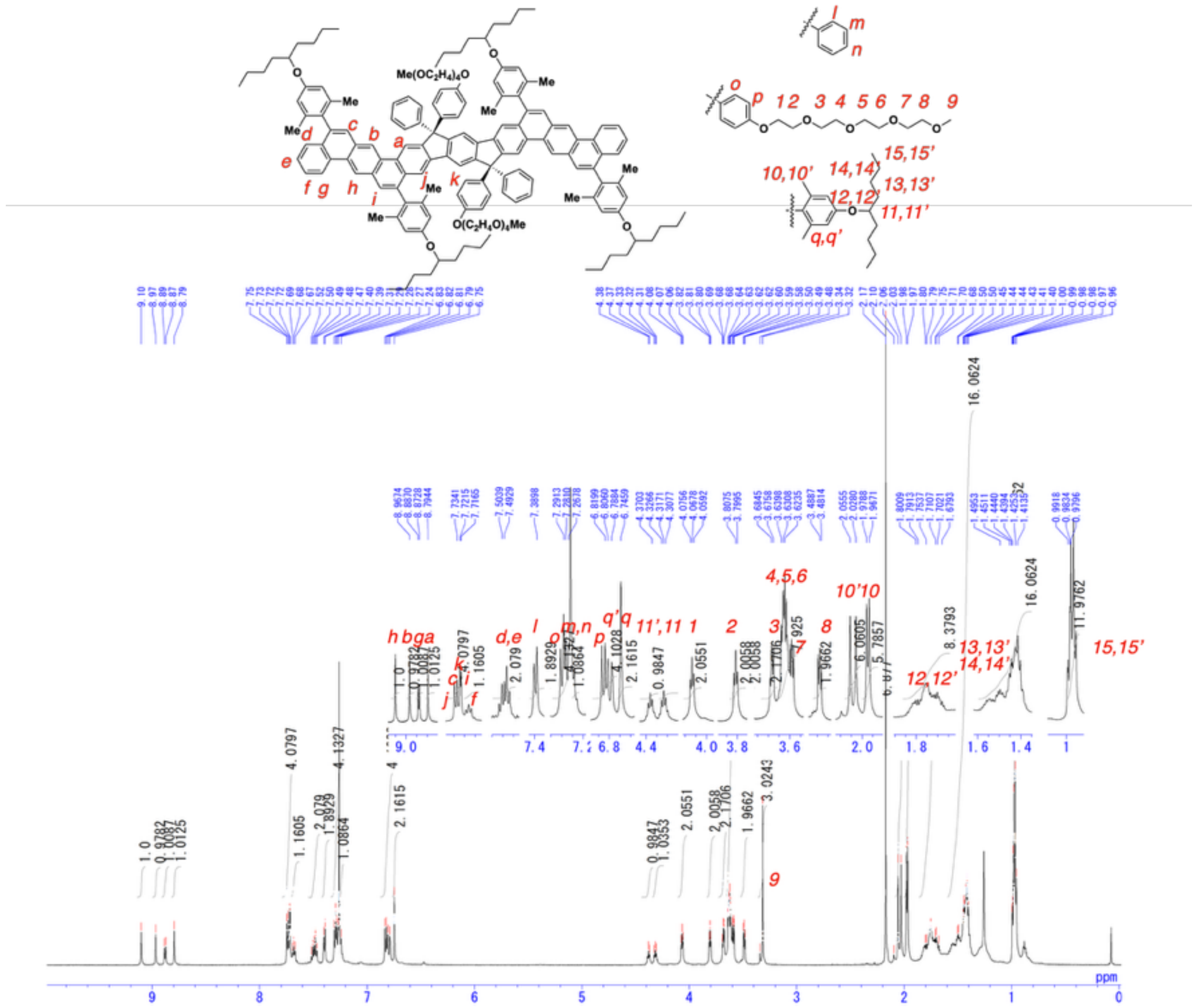

Supplementary Fig. 74 | ^{1}H NMR spectrum of Model-LP-(*S,S*)-**1** (600 MHz, $CDCl_3$, 25 ºC).

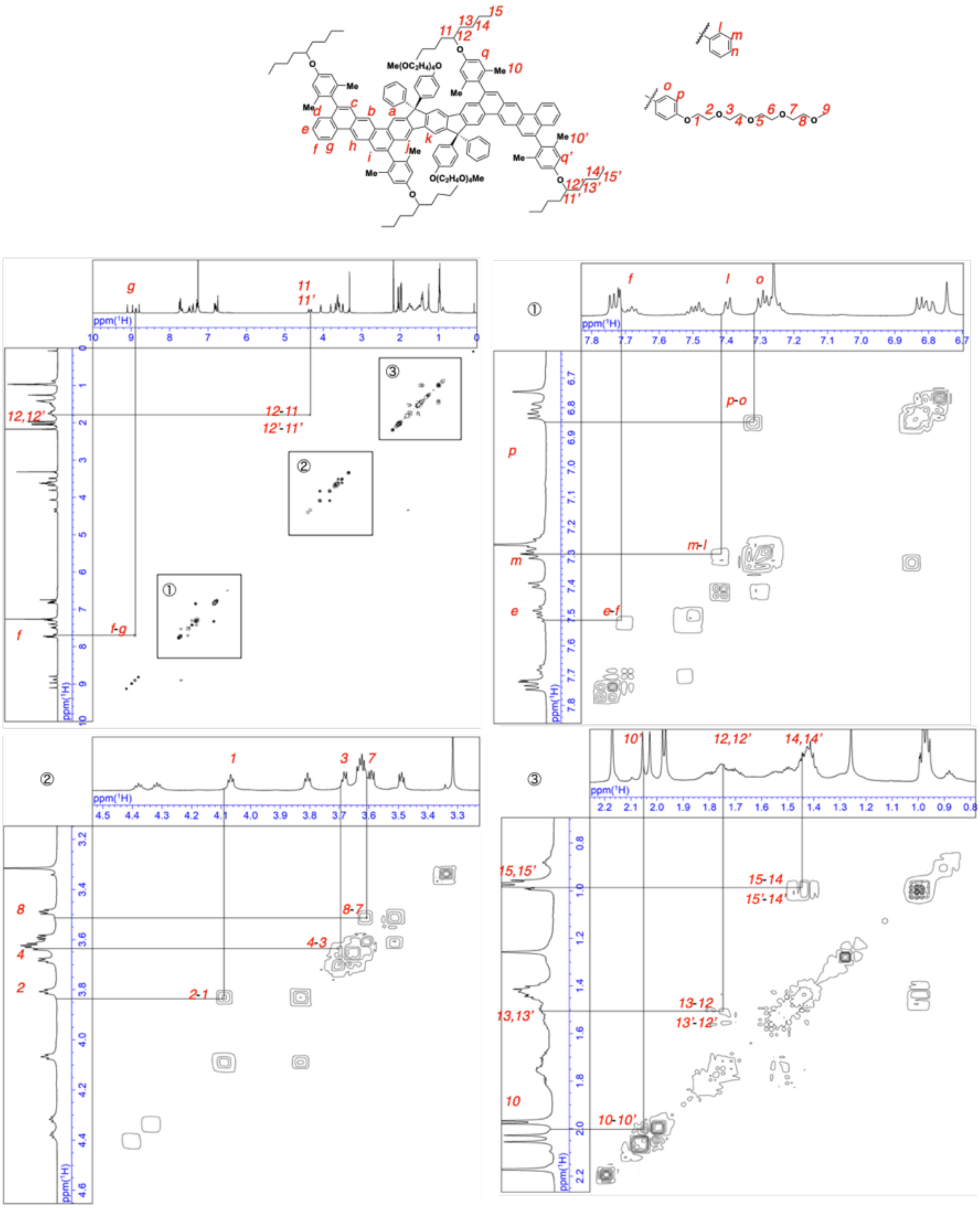

Supplementary Fig. 75 | HH-COSY correlation of Model-LP-(*S,S*)-**1** (600 MHz, $CDCl_3$, 25 ºC).

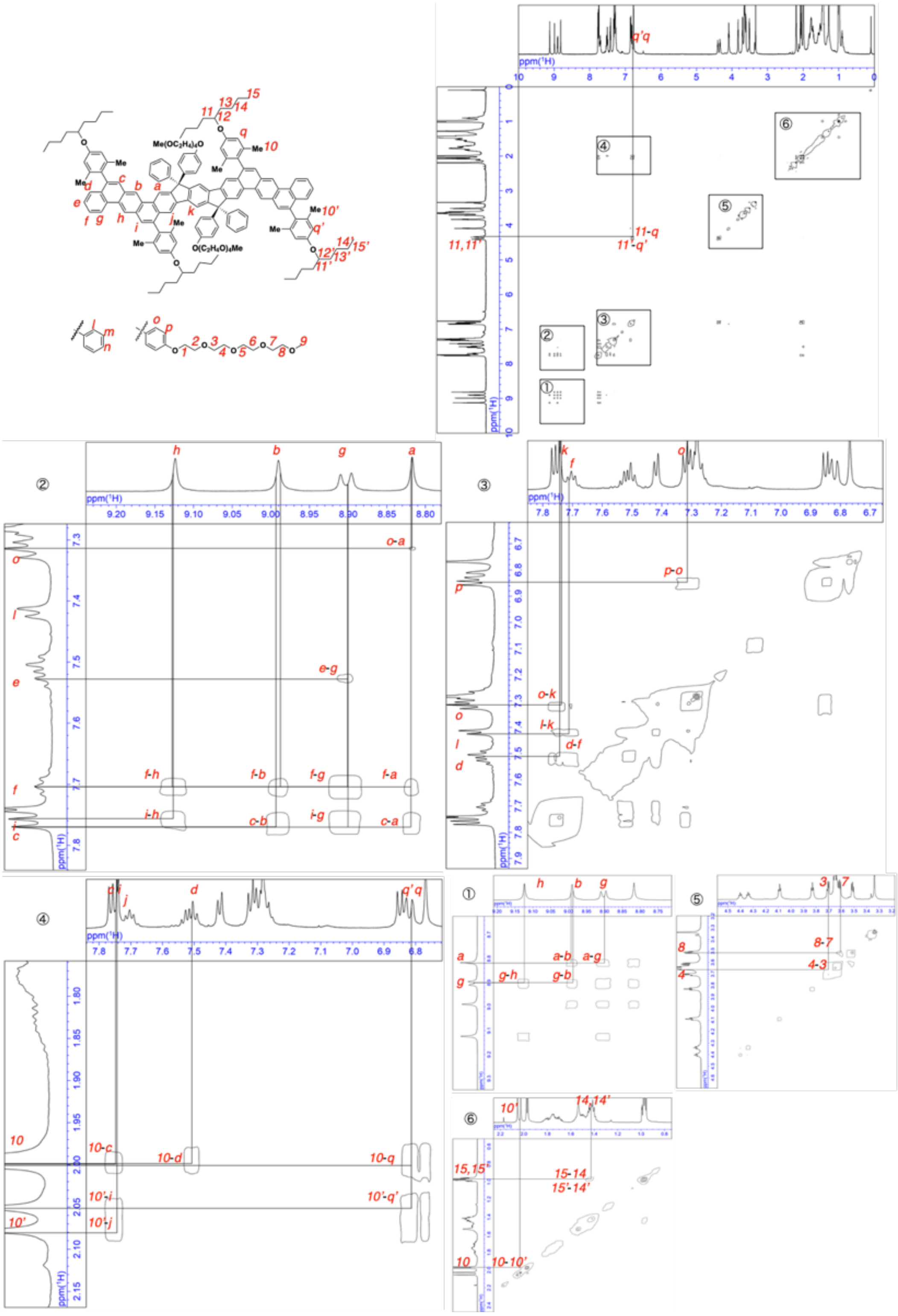

Supplementary Fig. 76 | NOESY correlation of Model-LP-(*S,S*)-**1** (600 MHz, $CDCl_3$, 25 ºC).

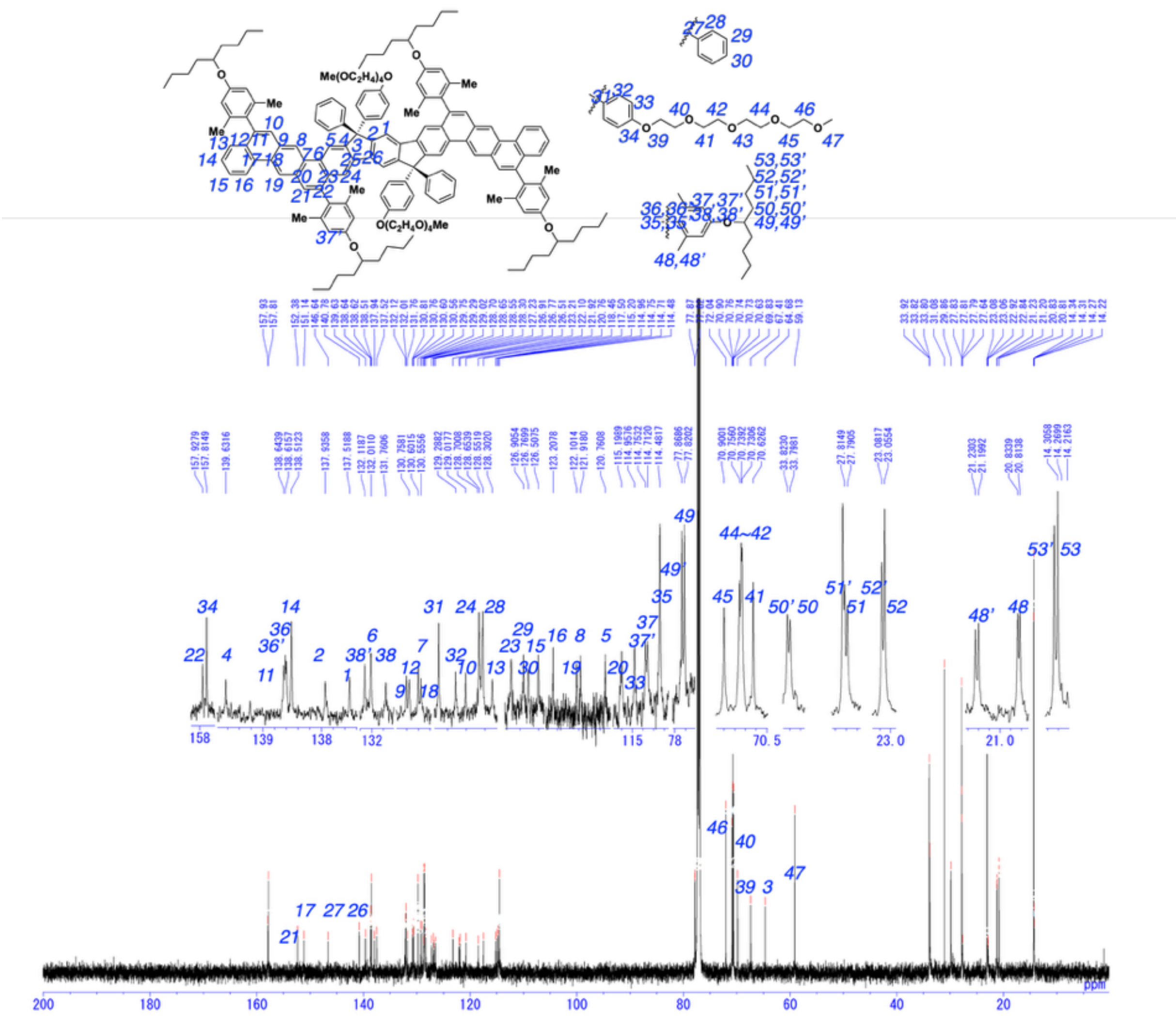

Supplementary Fig. 77 | ^{13}C NMR spectrum of Model-LP-(*S,S*)-**1** (151 MHz, $CDCl_3$, 25 ºC).

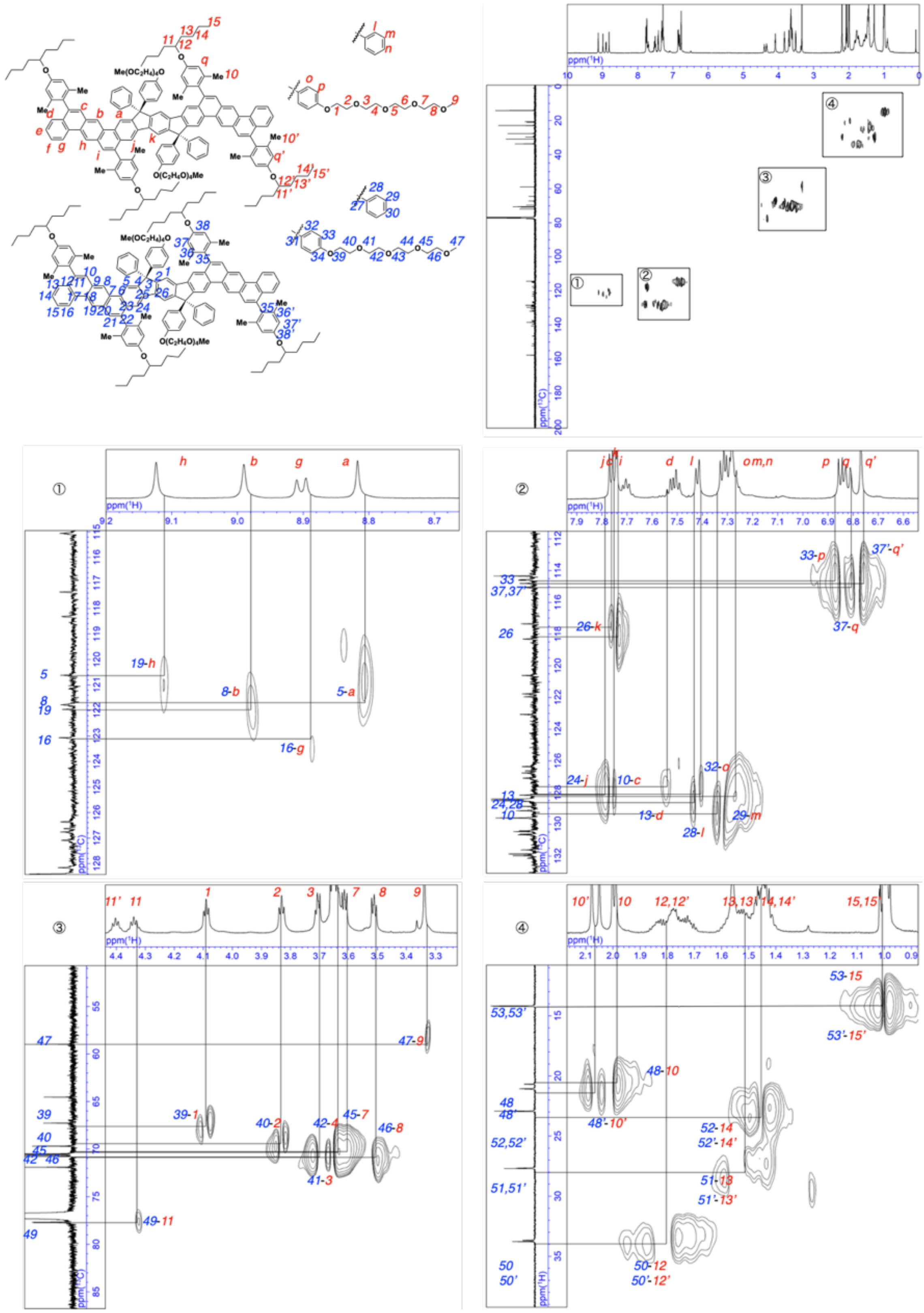

Supplementary Fig. 78 | HSQC correlation of Model-LP-(*S,S*)-**1** (600 MHz for ^{1}H and 151 MHz for ^{13}C, $CDCl_3$, 25 ºC).

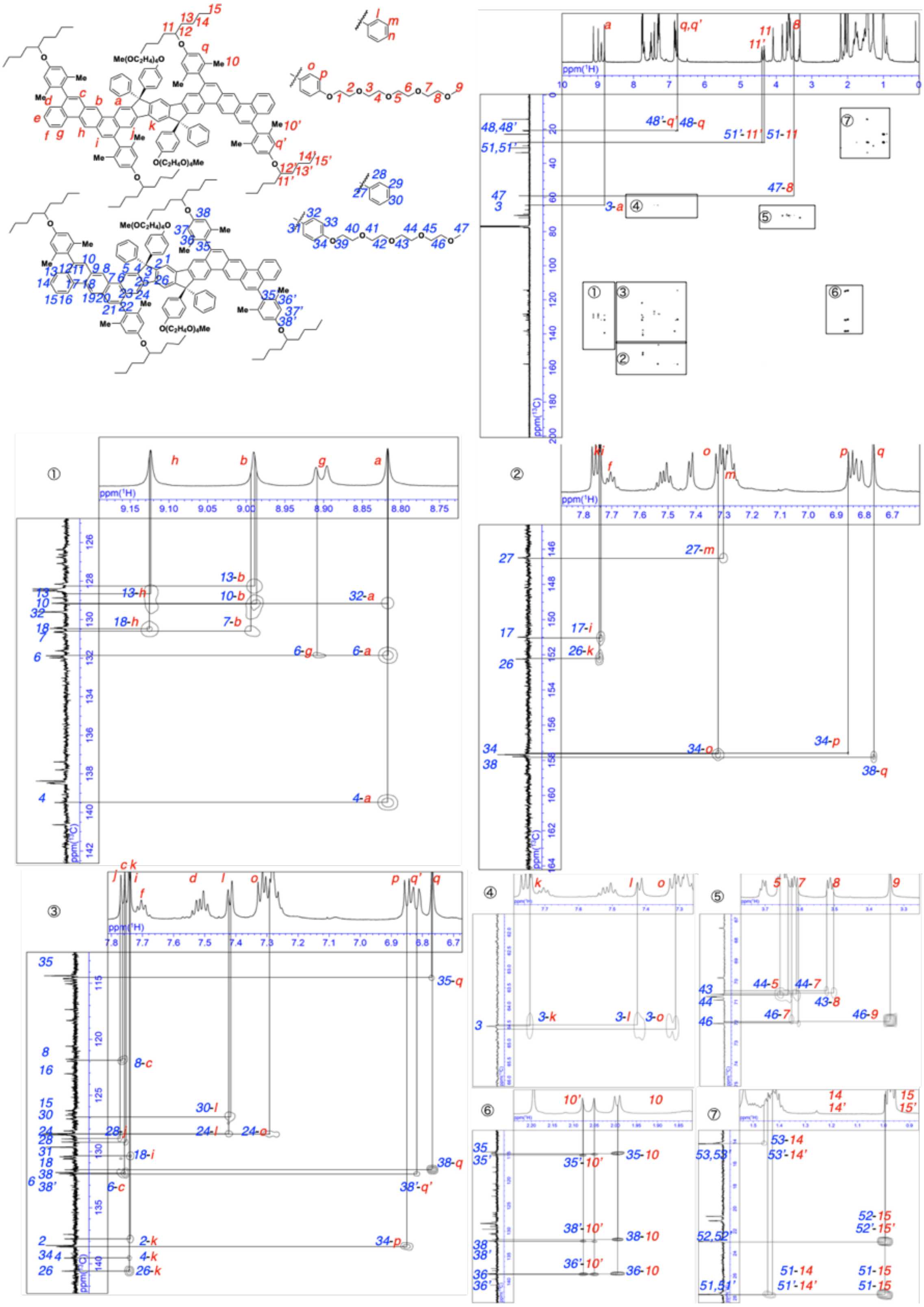

Supplementary Fig. 79 | HMBC correlation of Model-LP-(*S,S*)-**1** (600 MHz for ^{1}H and 151 MHz for ^{13}C, $CDCl_3$, 25 ºC).

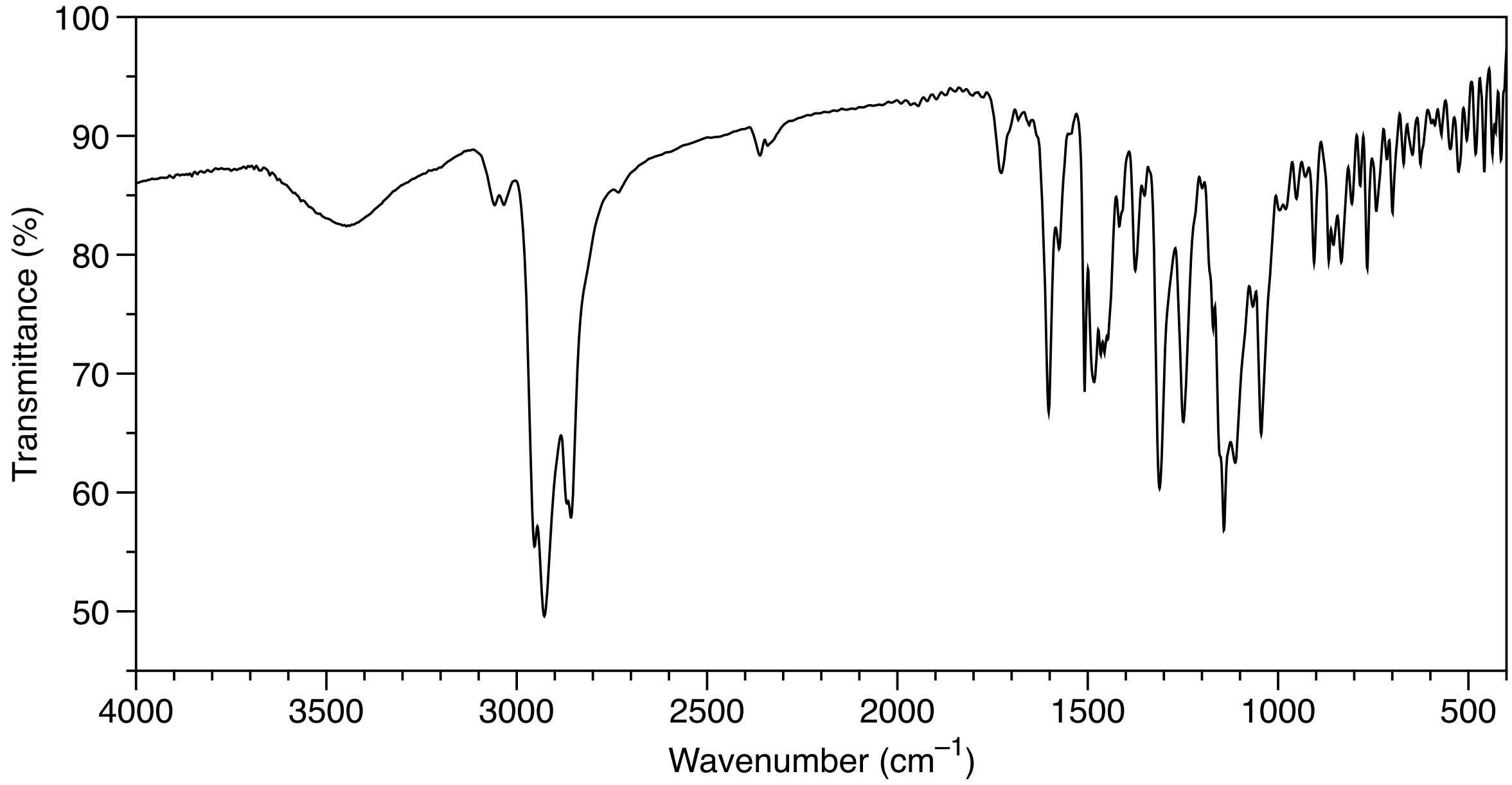

Supplementary Fig. 80 | FT-IR spectrum of Model-LP-(*S,S*)-**1** (KBr, 25 °C).

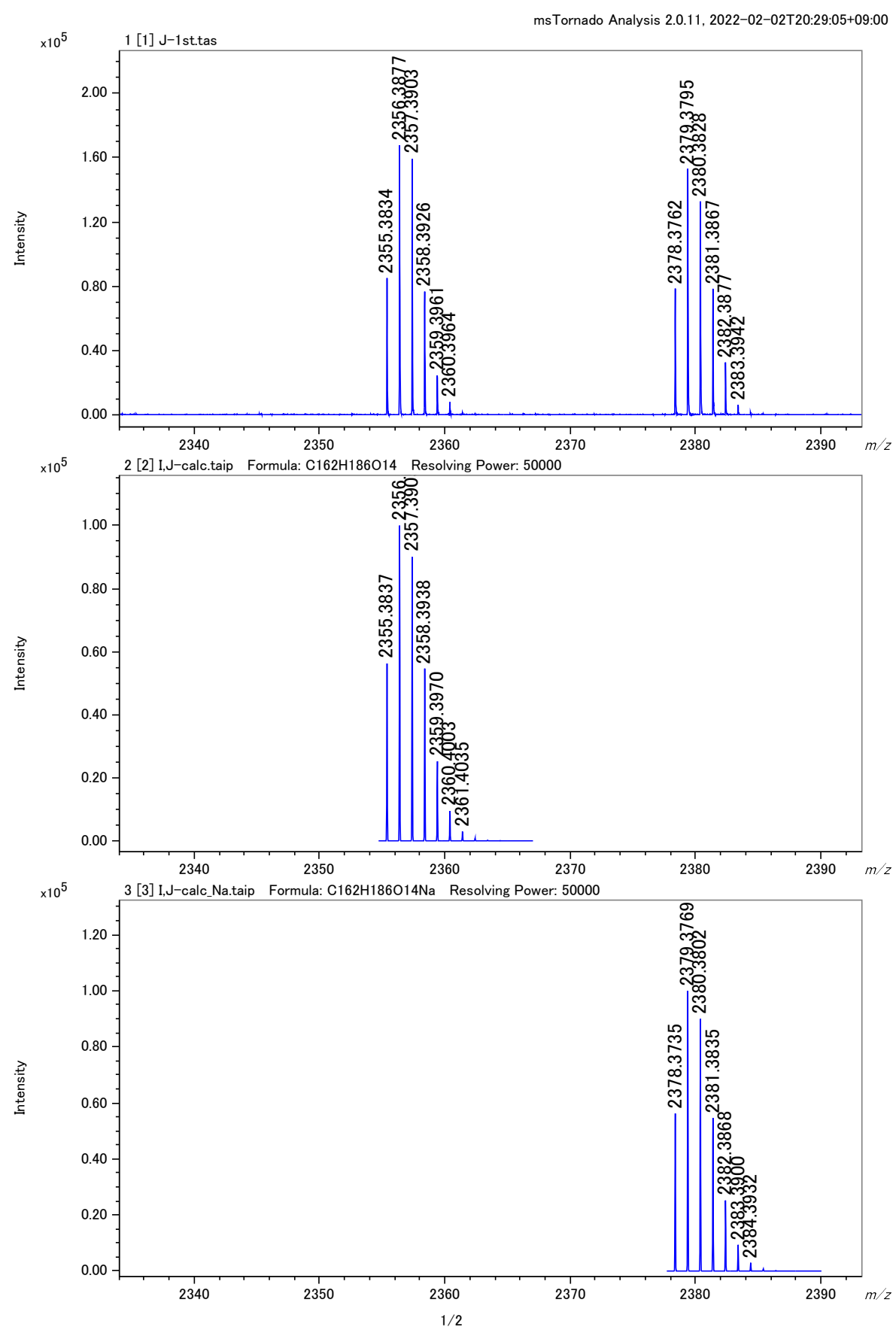

Supplementary Fig. 81 | Observed (a) and simulated high-resolution MALDI-Spiral-TOF-MS spectrum of Model-LP-(*S*,*S*)-**1** for [M]$^+$ (middle) and [M+Na]$^+$ (lower).

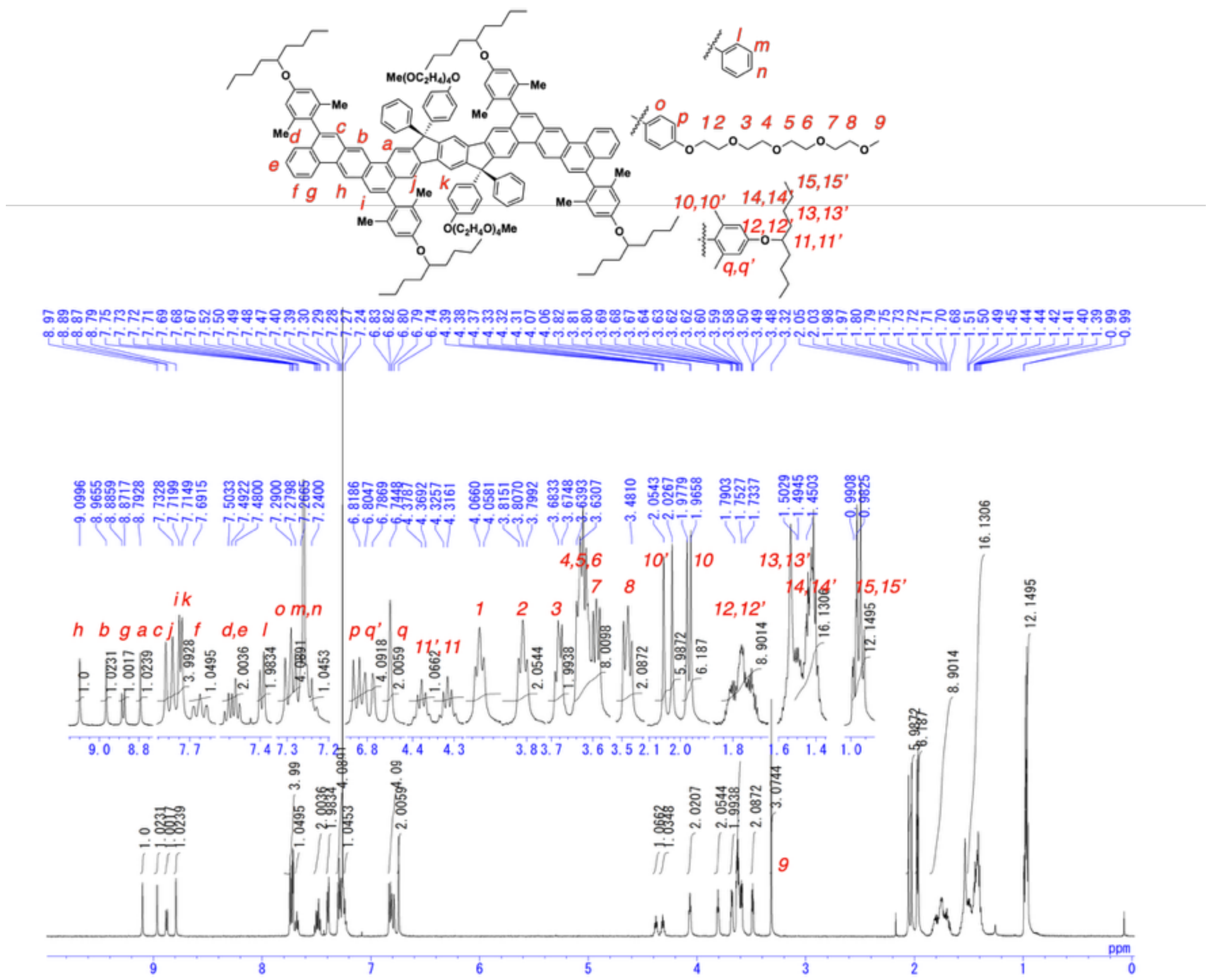

Supplementary Fig. 82 | ^{1}H NMR spectrum of Model-LP-(*R,R*)-**1** (600 MHz, $CDCl_3$, 25 ºC).

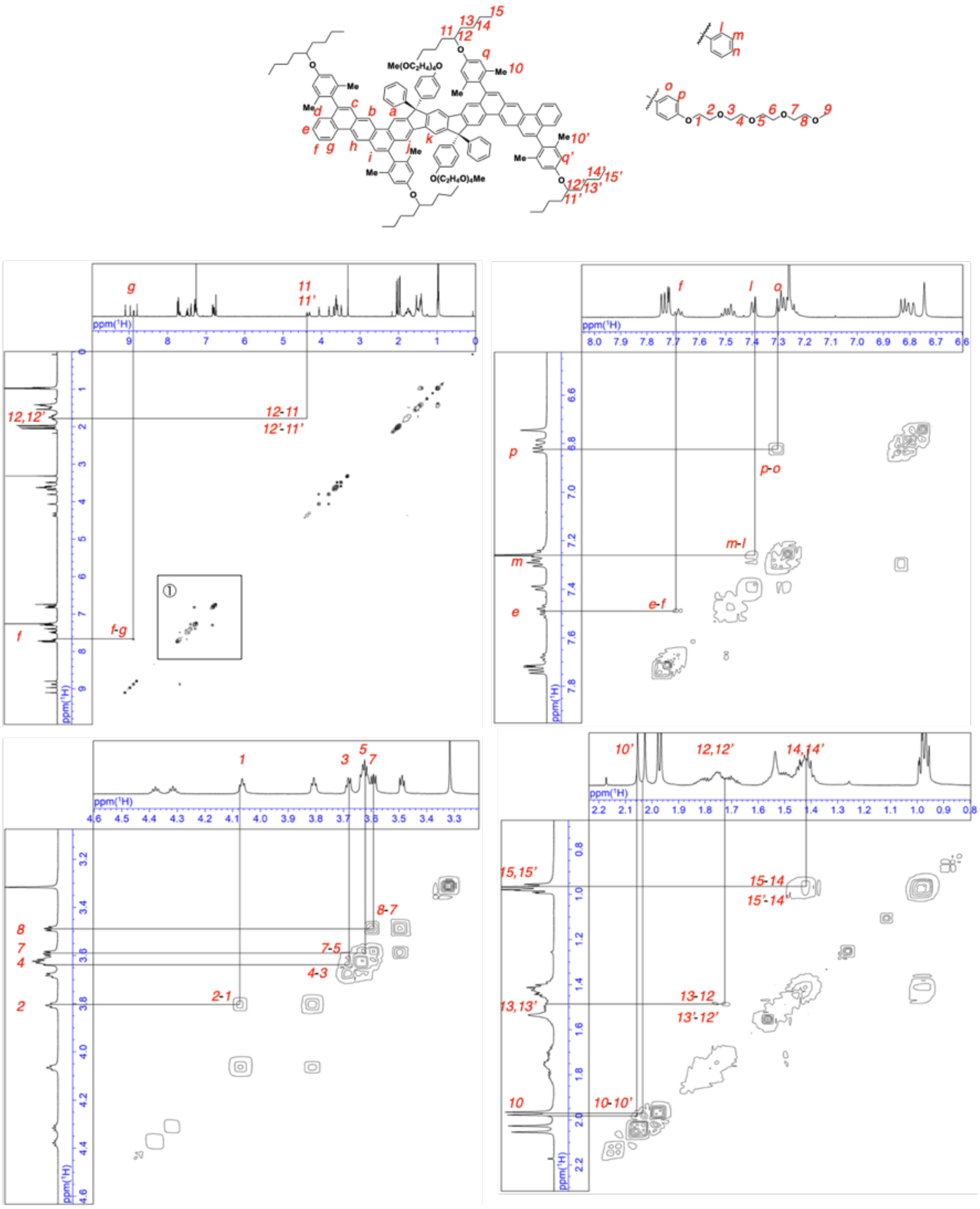

Supplementary Fig. 83 | HH-COSY correlation of Model-LP-(*R,R*)-**1** (600 MHz, $CDCl_3$, 25 ºC).

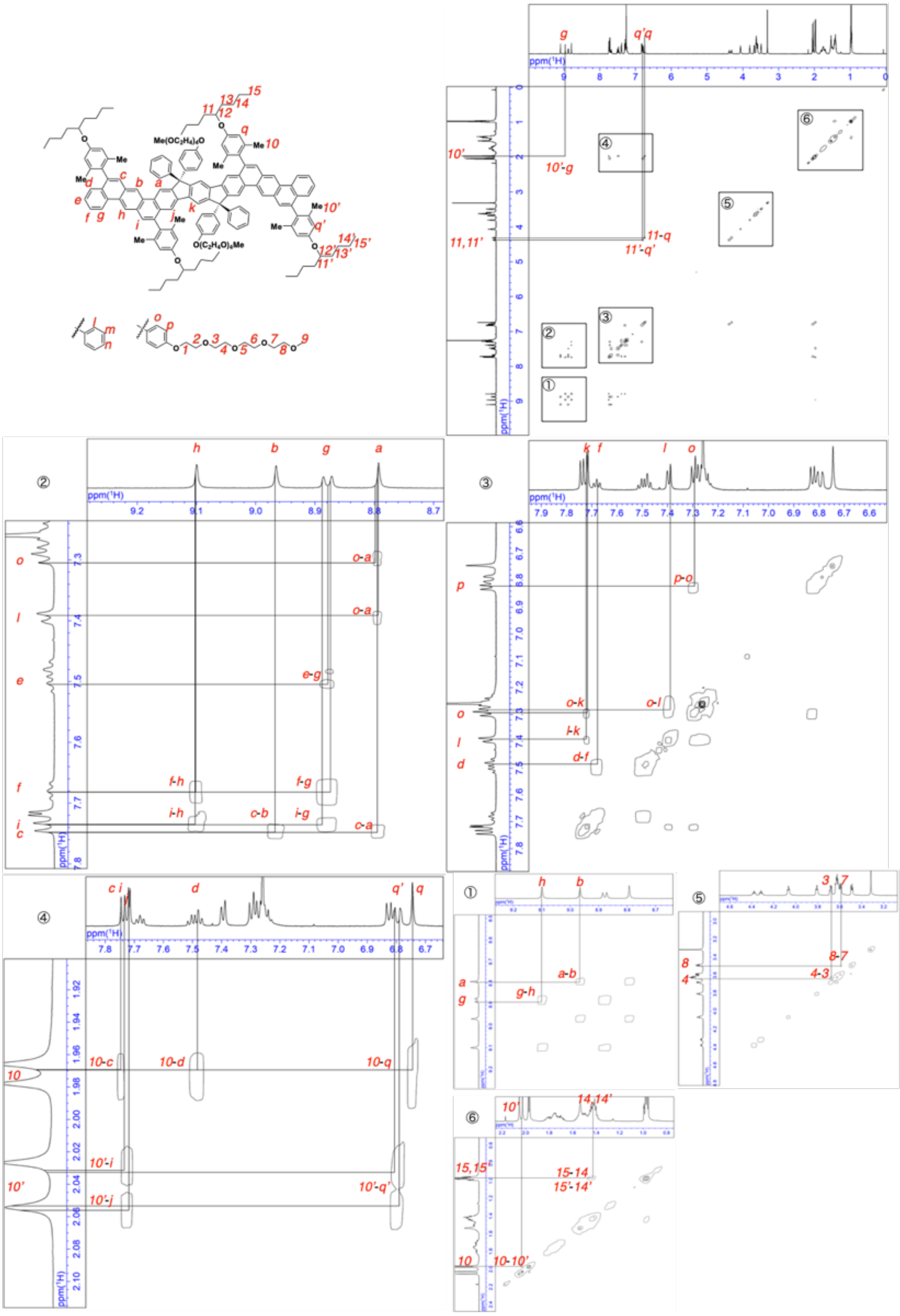

Supplementary Fig. 84 | NOESY correlation of Model-LP-(*R*,*R*)-**1** (600 MHz, $CDCl_3$, 25 ºC).

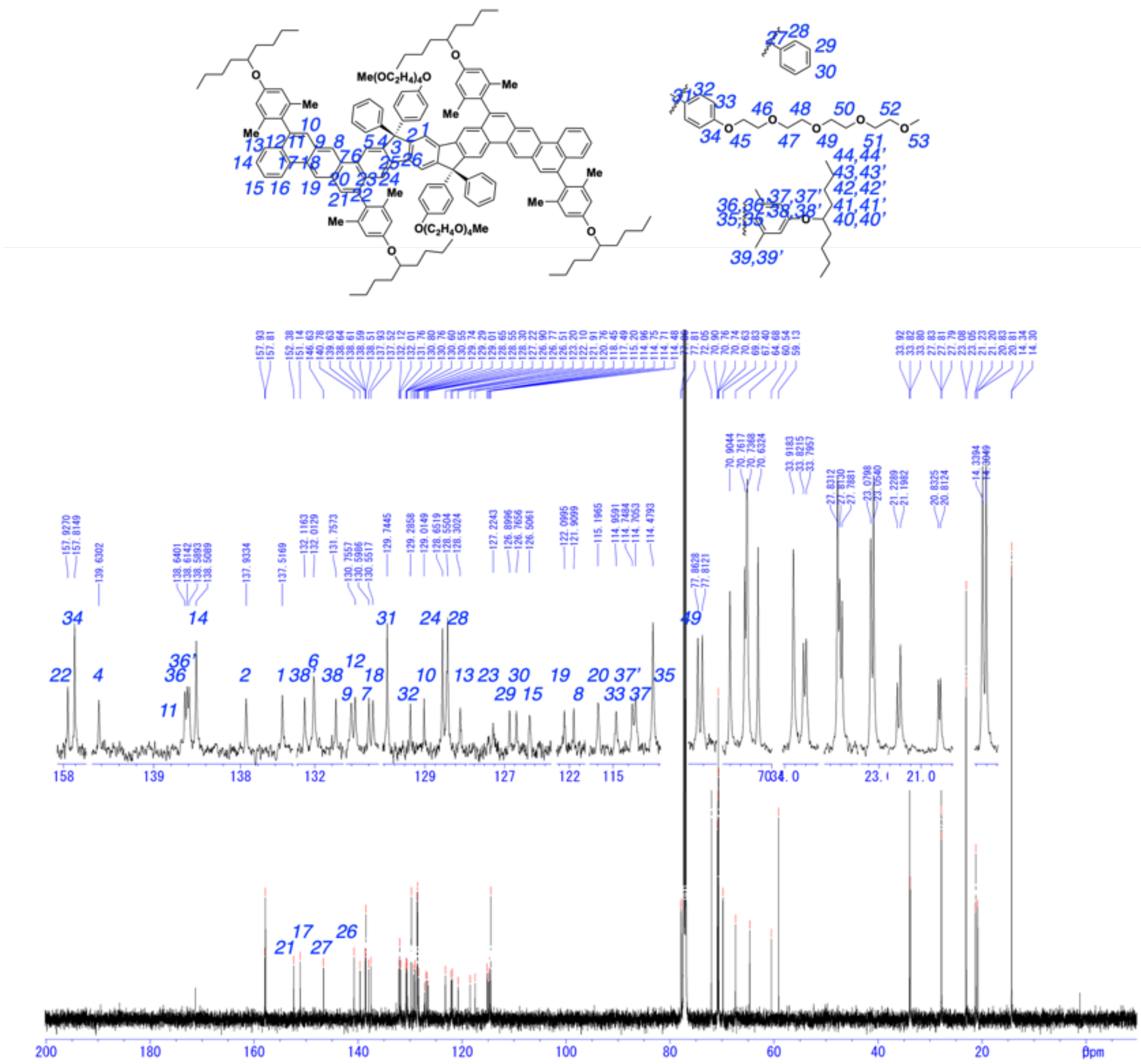

Supplementary Fig. 85 | ^{13}C NMR spectrum of Model-LP-(*R,R*)-**1** (151 MHz, $CDCl_3$, 25 ºC).

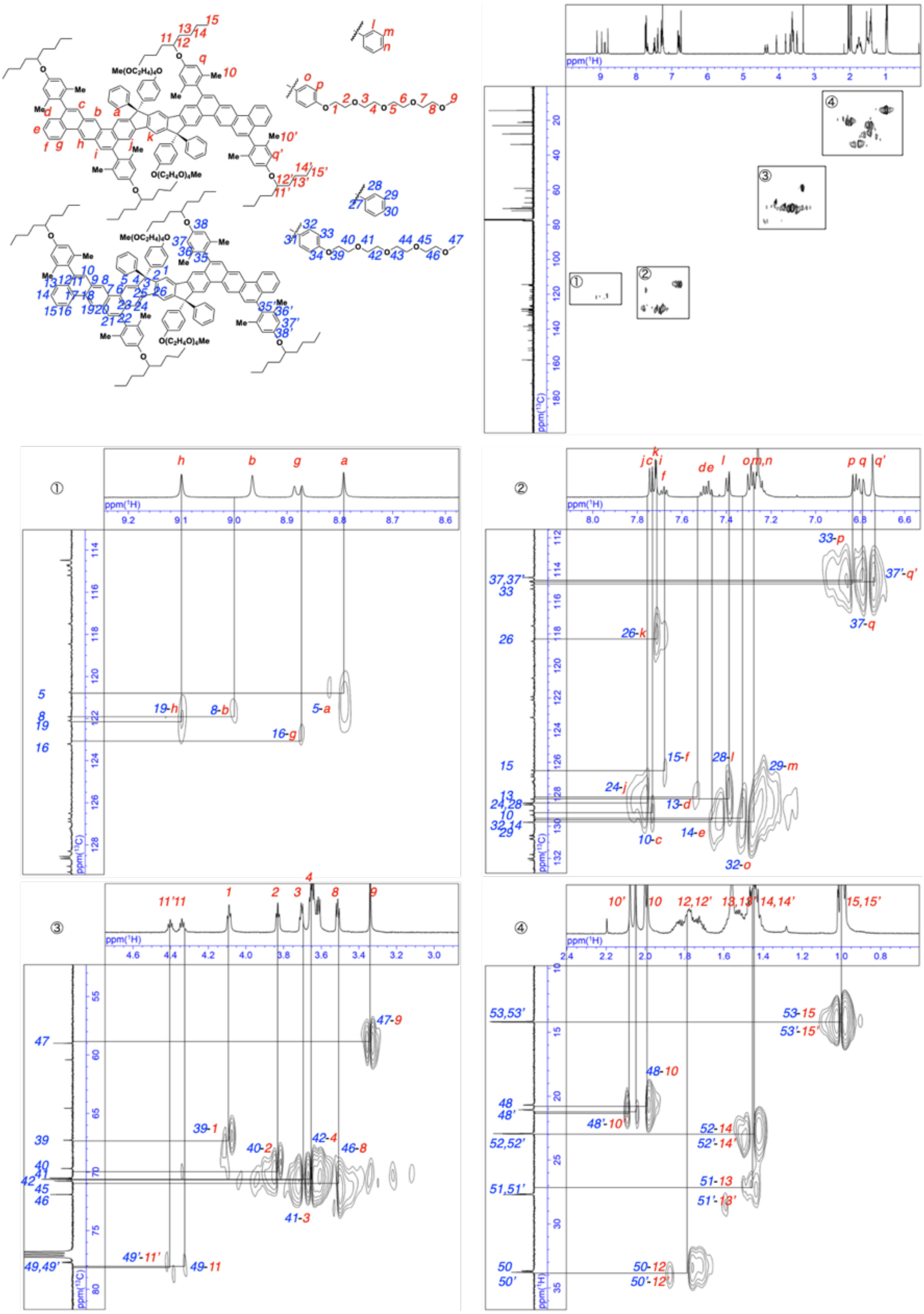

Supplementary Fig. 86 | HSQC correlation of Model-LP-(*R*,*R*)-**1** (600 MHz for ^{1}H and 151 MHz for ^{13}C, $CDCl_3$, 25 ºC).

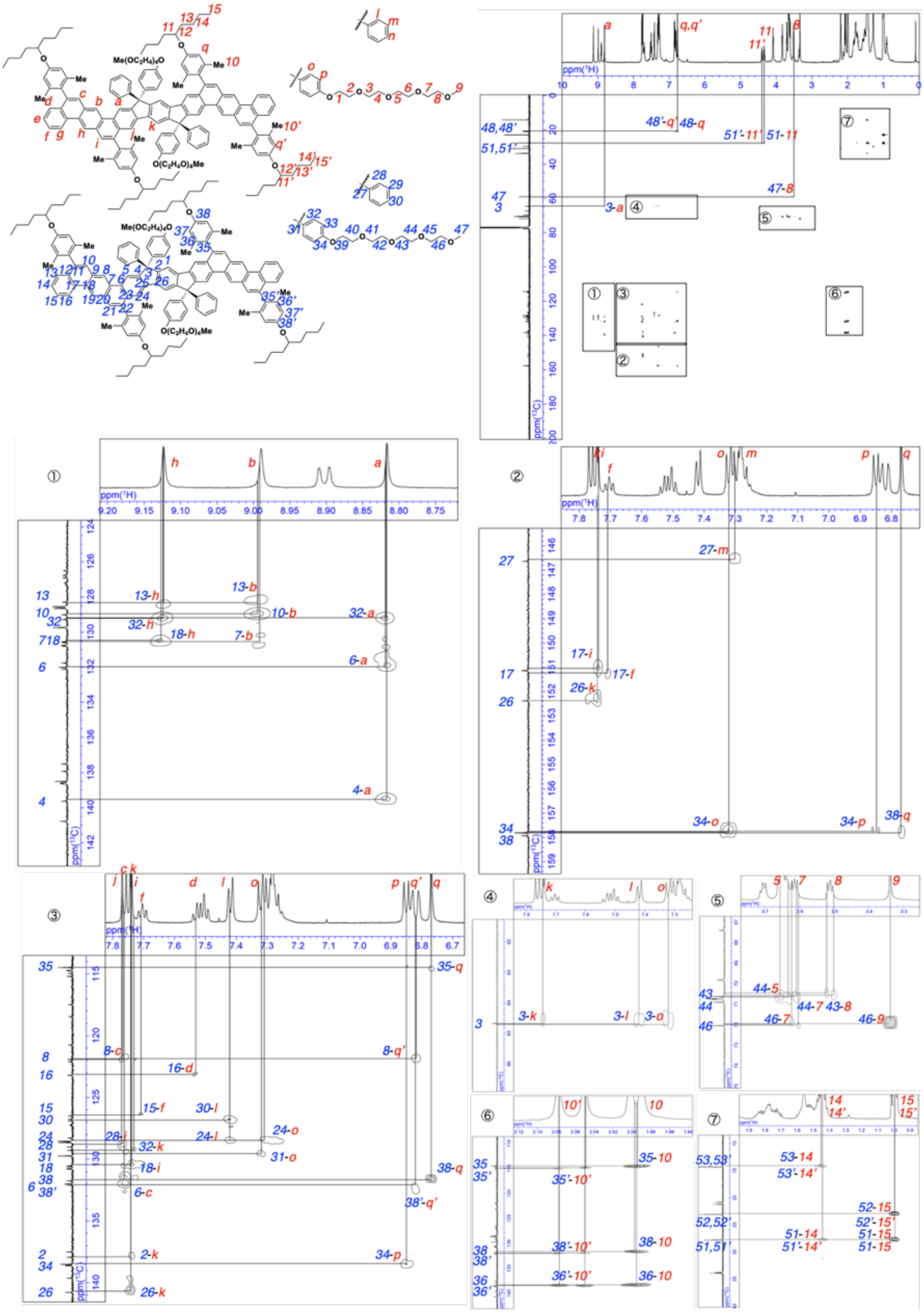

Supplementary Fig. 87 | HMBC correlation of Model-LP-(R,R)-**1** (600 MHz for ^{1}H and 151 MHz for ^{13}C, $CDCl_3$, 25 ºC).

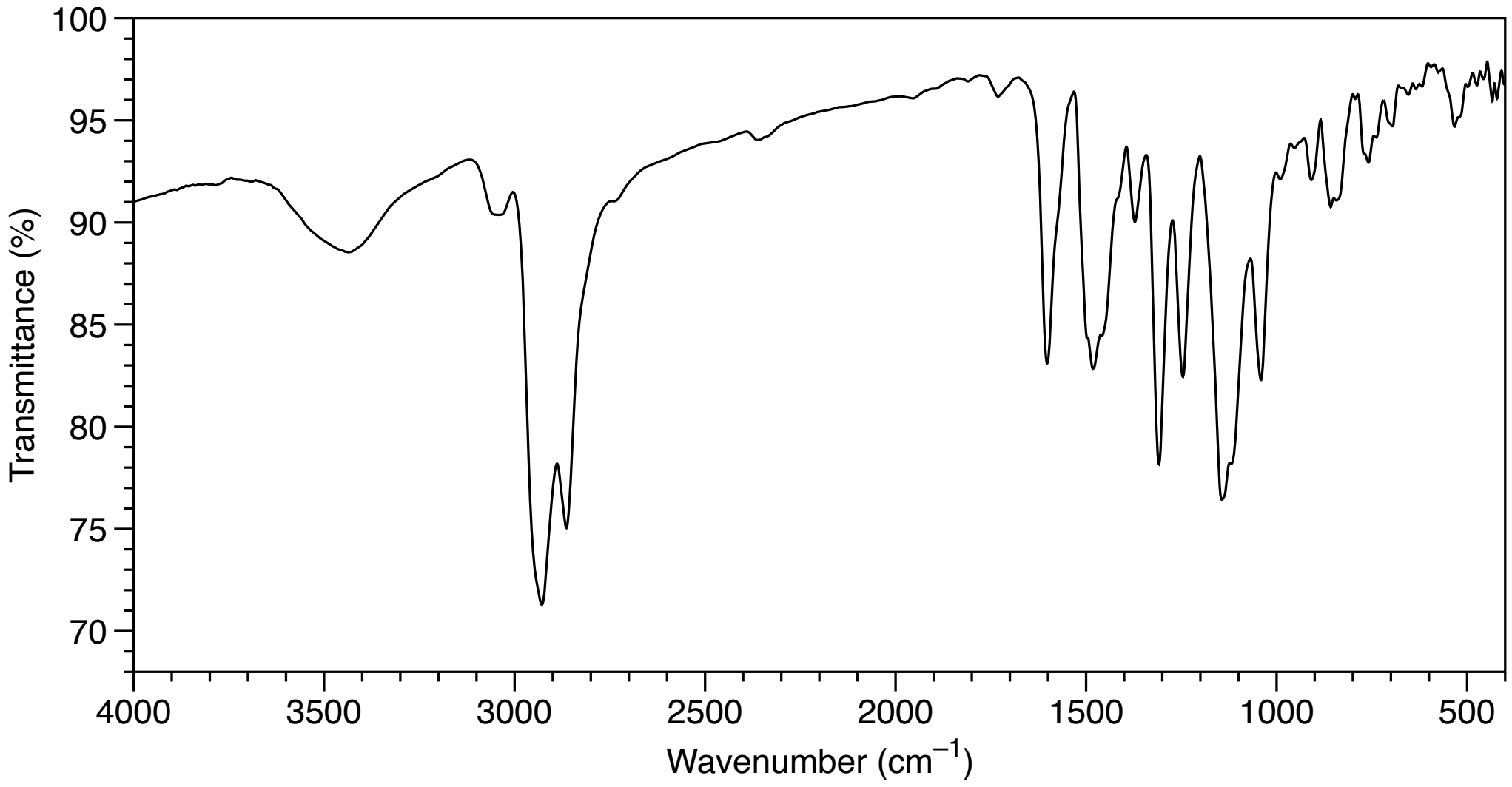

Supplementary Fig. 88 | FT-IR spectrum of Model-LP-(*R,R*)-**1** (KBr, 25 °C).

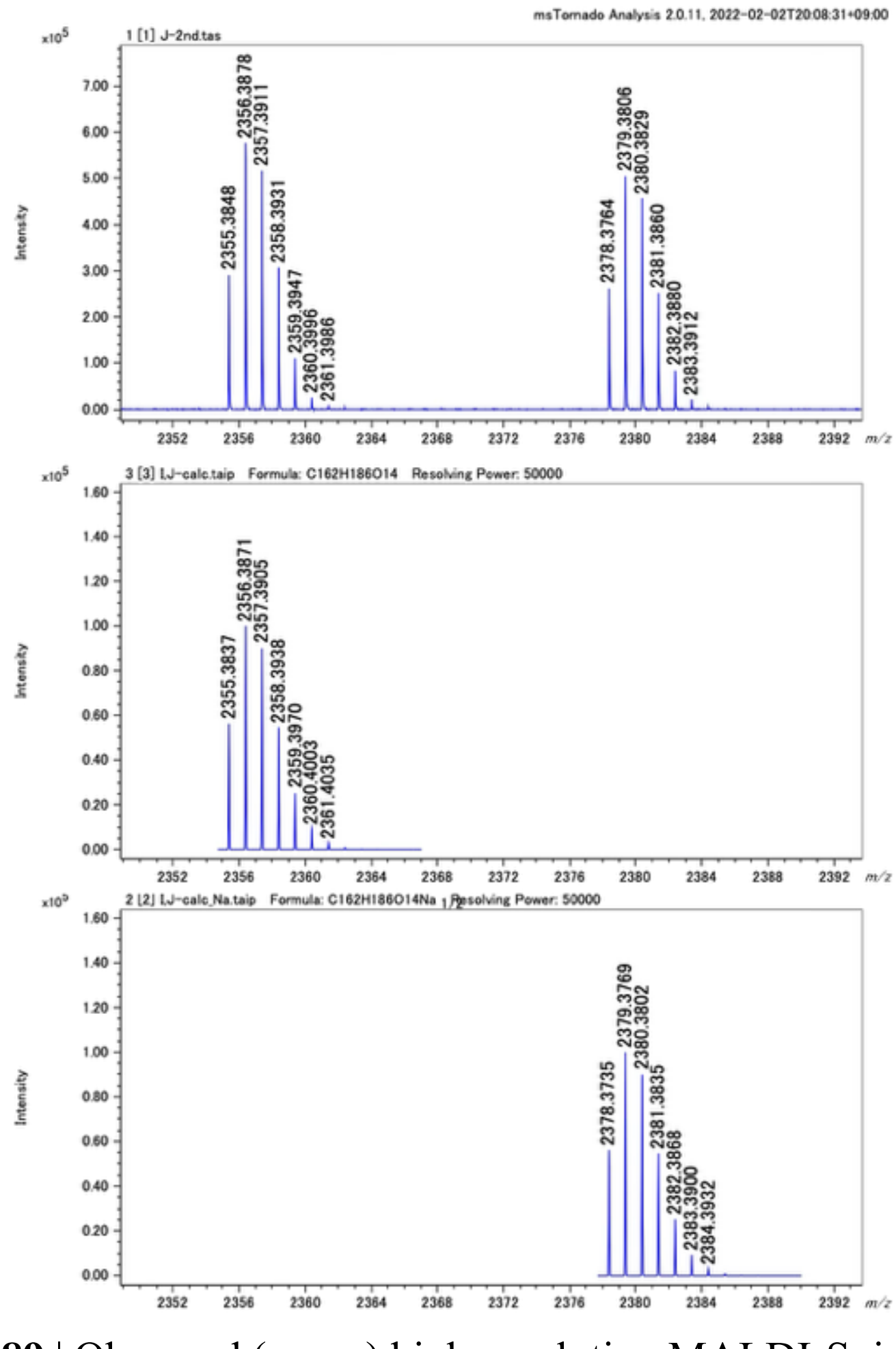

Supplementary Fig. 89 | Observed (upper) high-resolution MALDI-Spiral-TOF-MS spectrum of Model-LP-(*R*,*R*)-**1** for $[M]^+$ (middle) and $[M+Na]^+$ (lower).

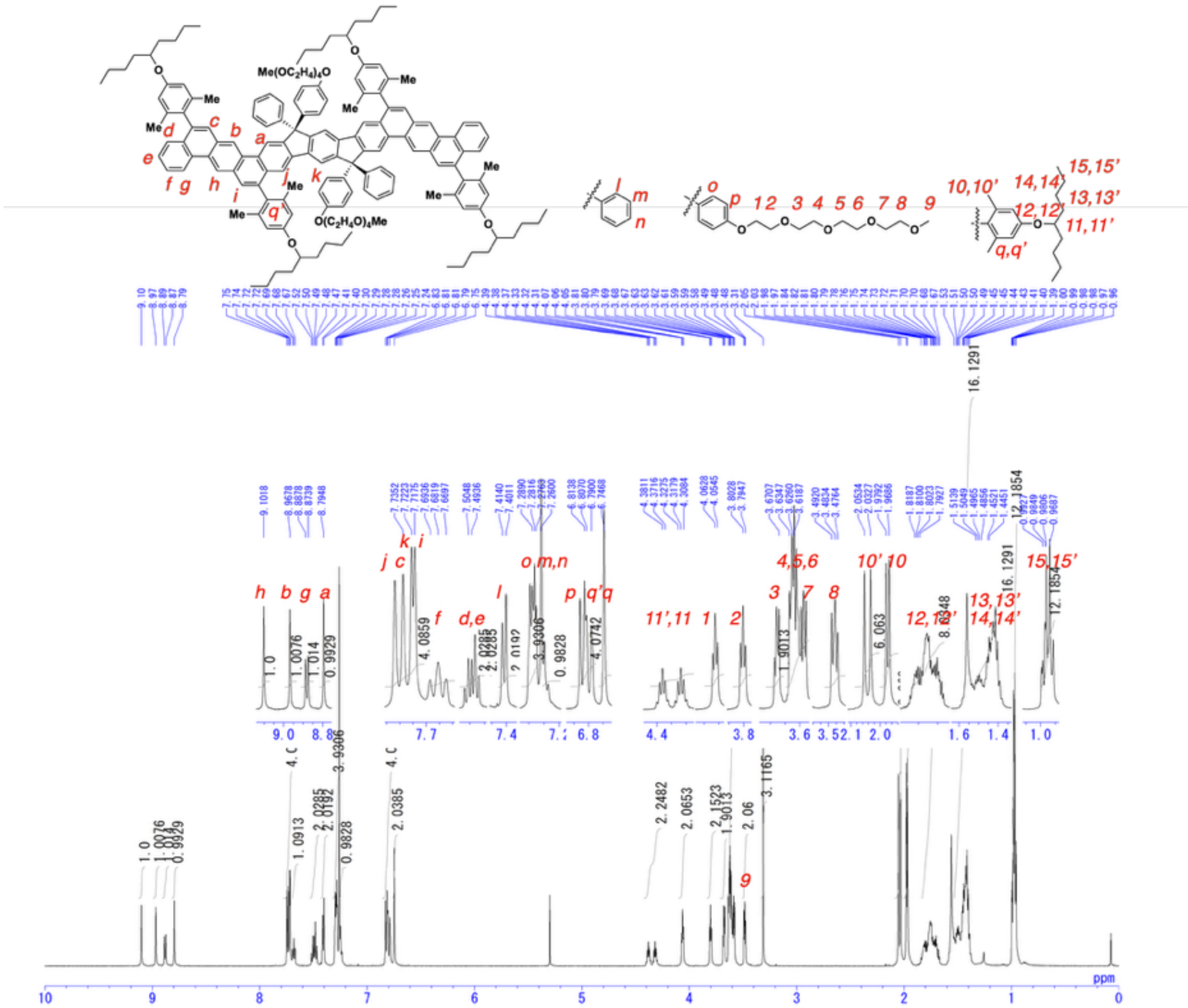

Supplementary Fig. 90 | ^{1}H NMR spectrum of Model-LP-*meso*-**1** (600 MHz, $CDCl_3$, 25 ºC).

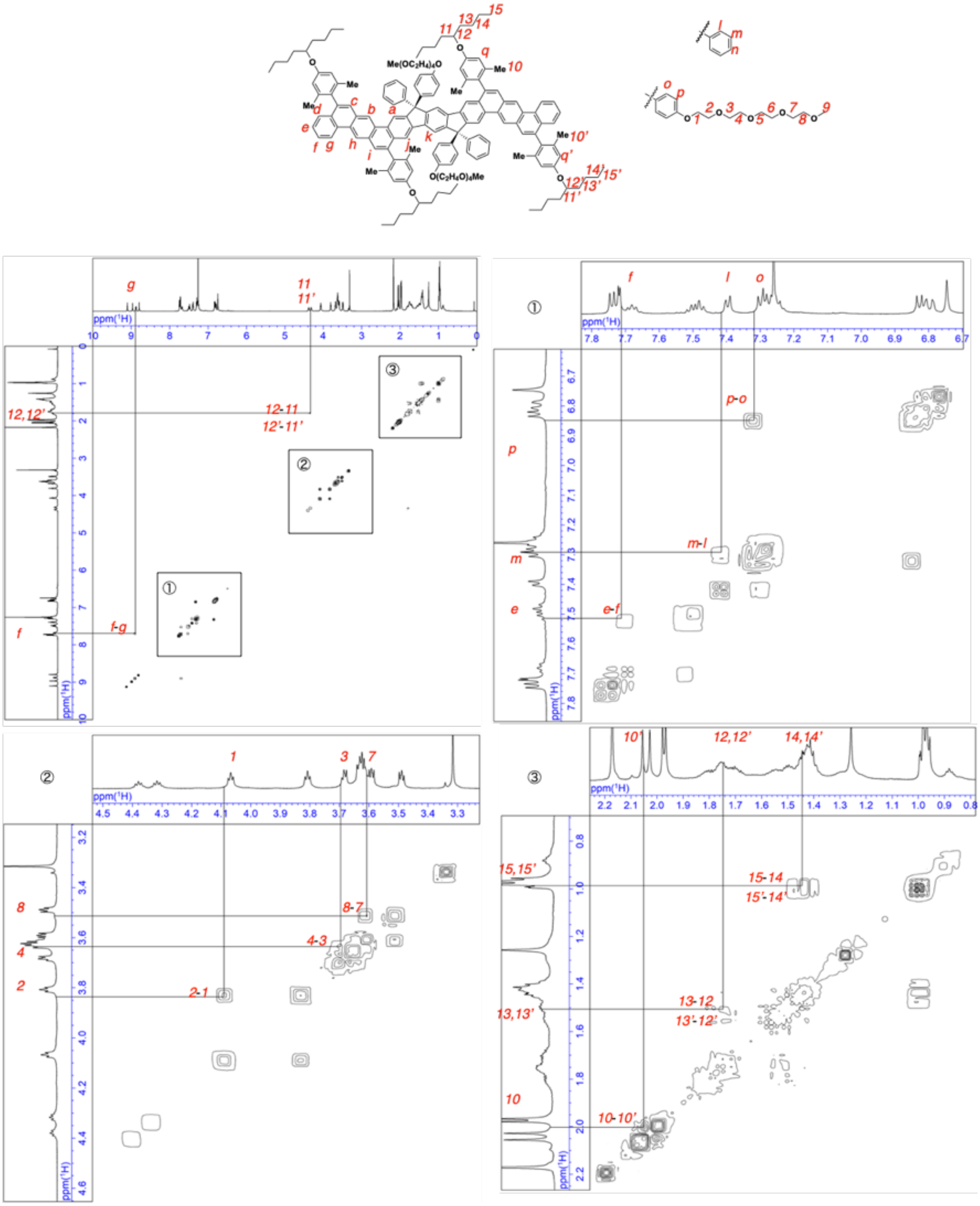

Supplementary Fig. 91 | HH-COSY correlation of Model-LP-*meso*-**1** (600 MHz, $CDCl_3$, 25 ºC).

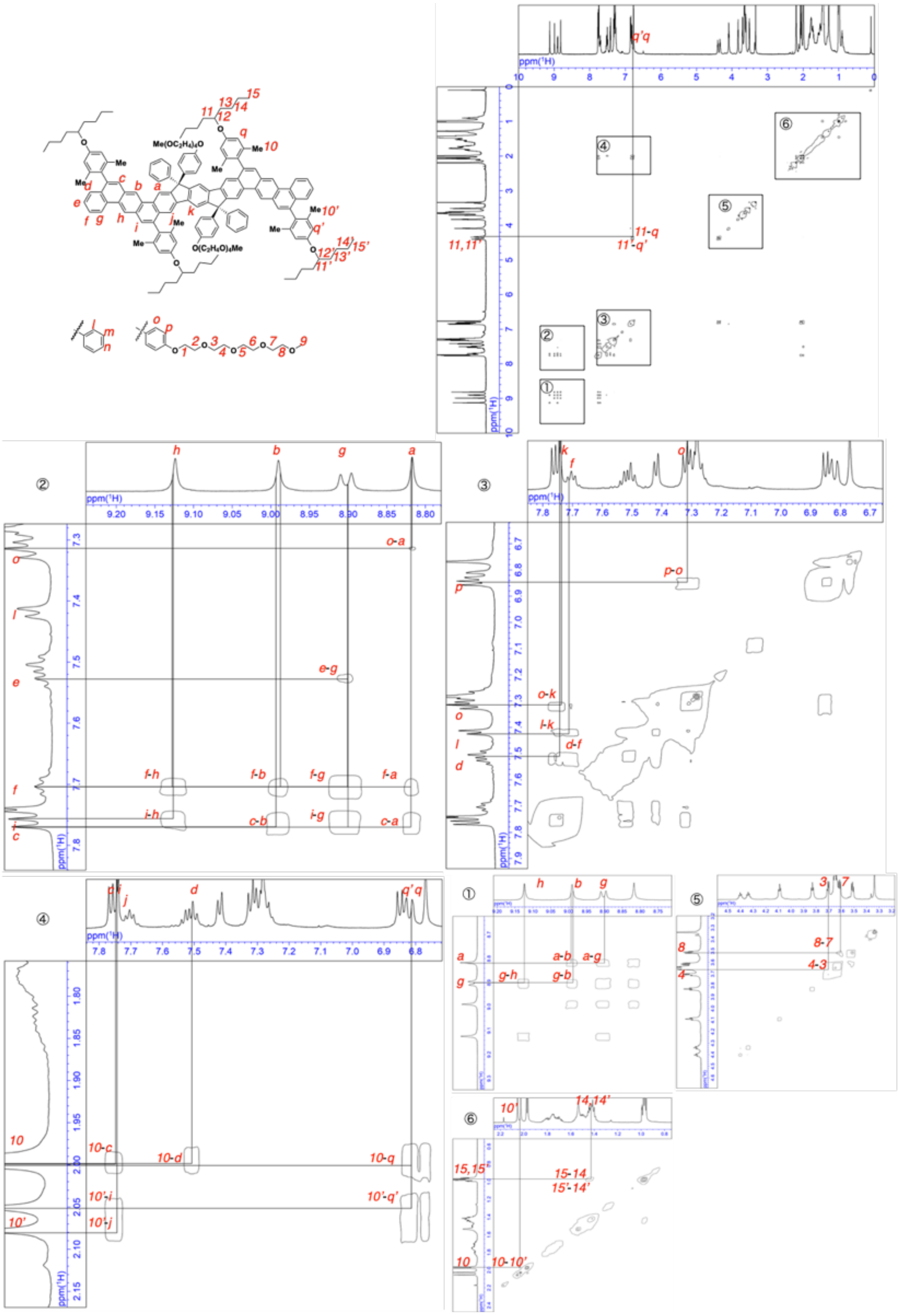

Supplementary Fig. 92 | NOESY correlation of Model-LP-*meso*-**1** (600 MHz, $CDCl_3$, 25 ºC).

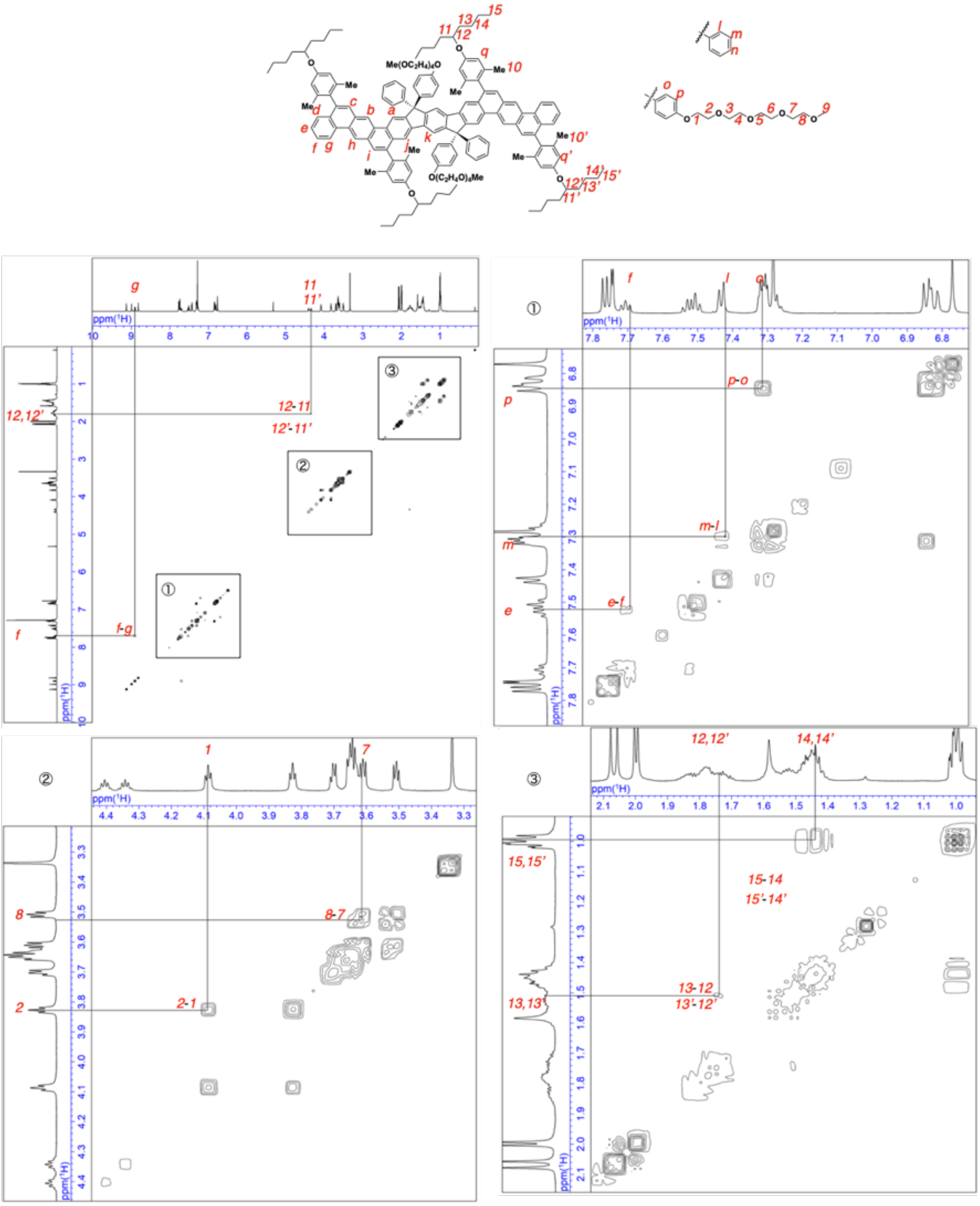

Supplementary Fig. 93 | HH-COSY correlation of Model-LP-*meso*-**1** (600 MHz, $CDCl_3$, 25 ºC).

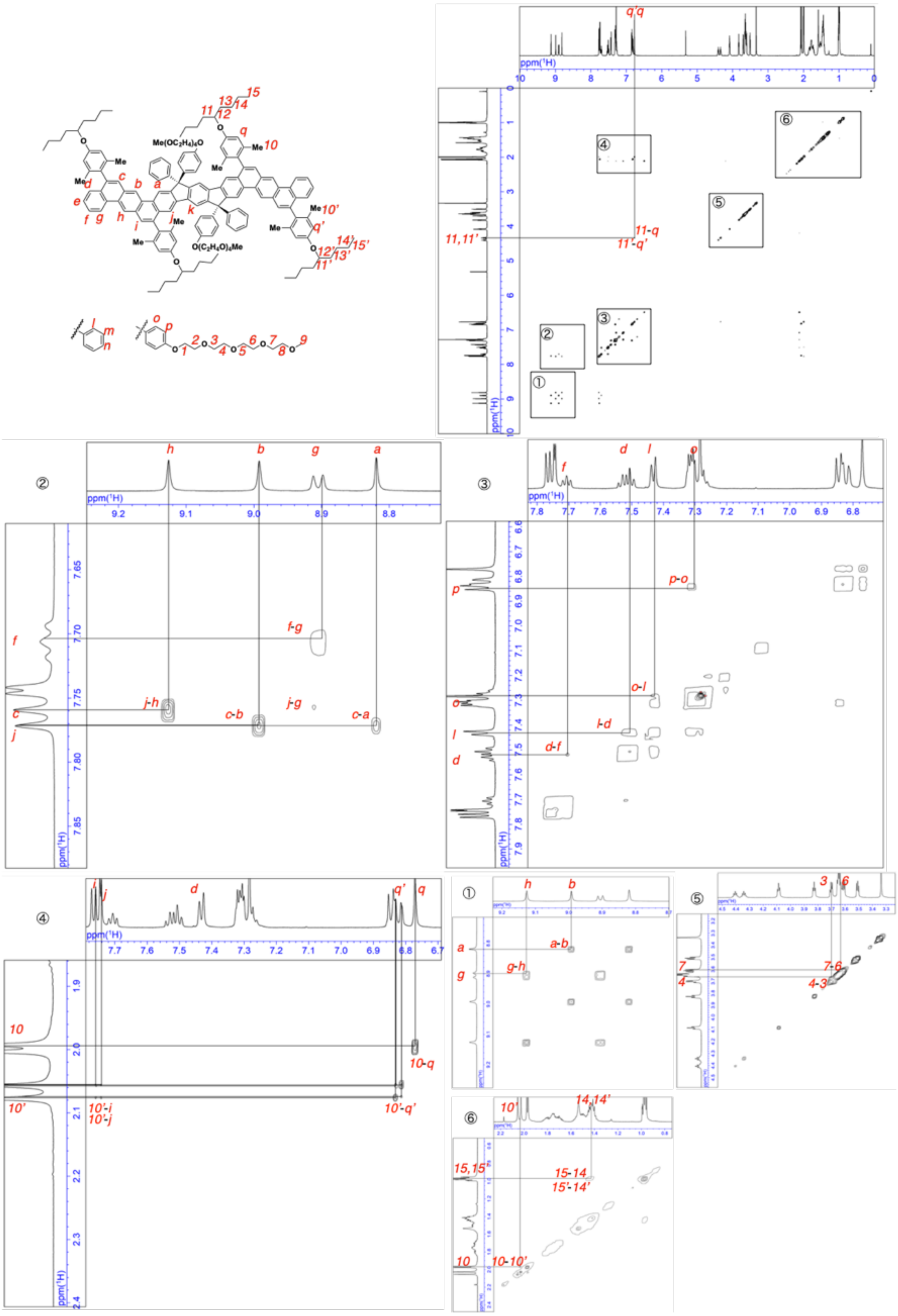

Supplementary Fig. 94 | NOESY correlation of Model-LP-*meso*-**1** (600 MHz, $CDCl_3$, 25 ºC).

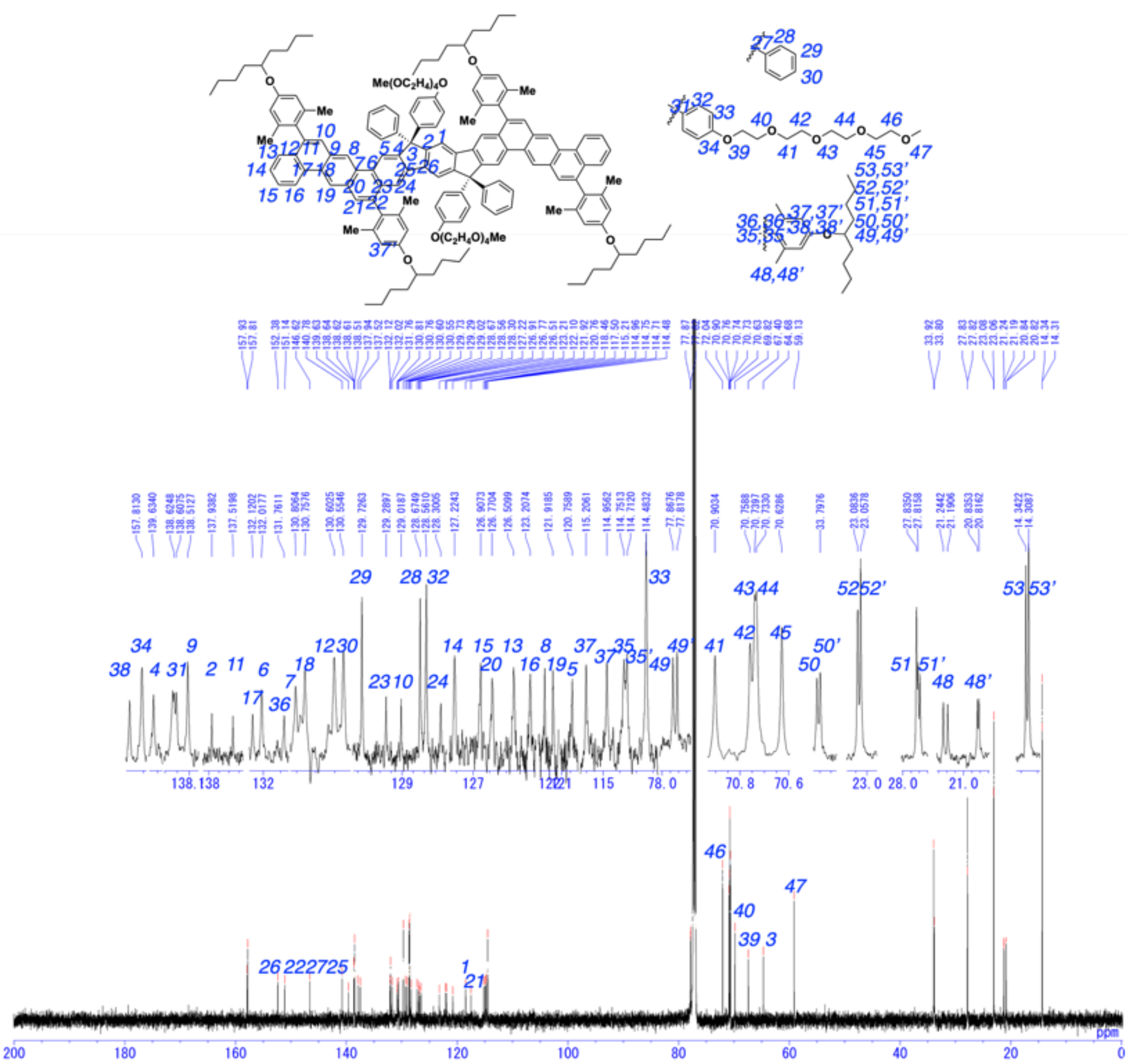

Supplementary Fig. 95 | ^{13}C NMR spectrum of Model-LP-*meso*-**1** (151 MHz, $CDCl_3$, 25 ºC).

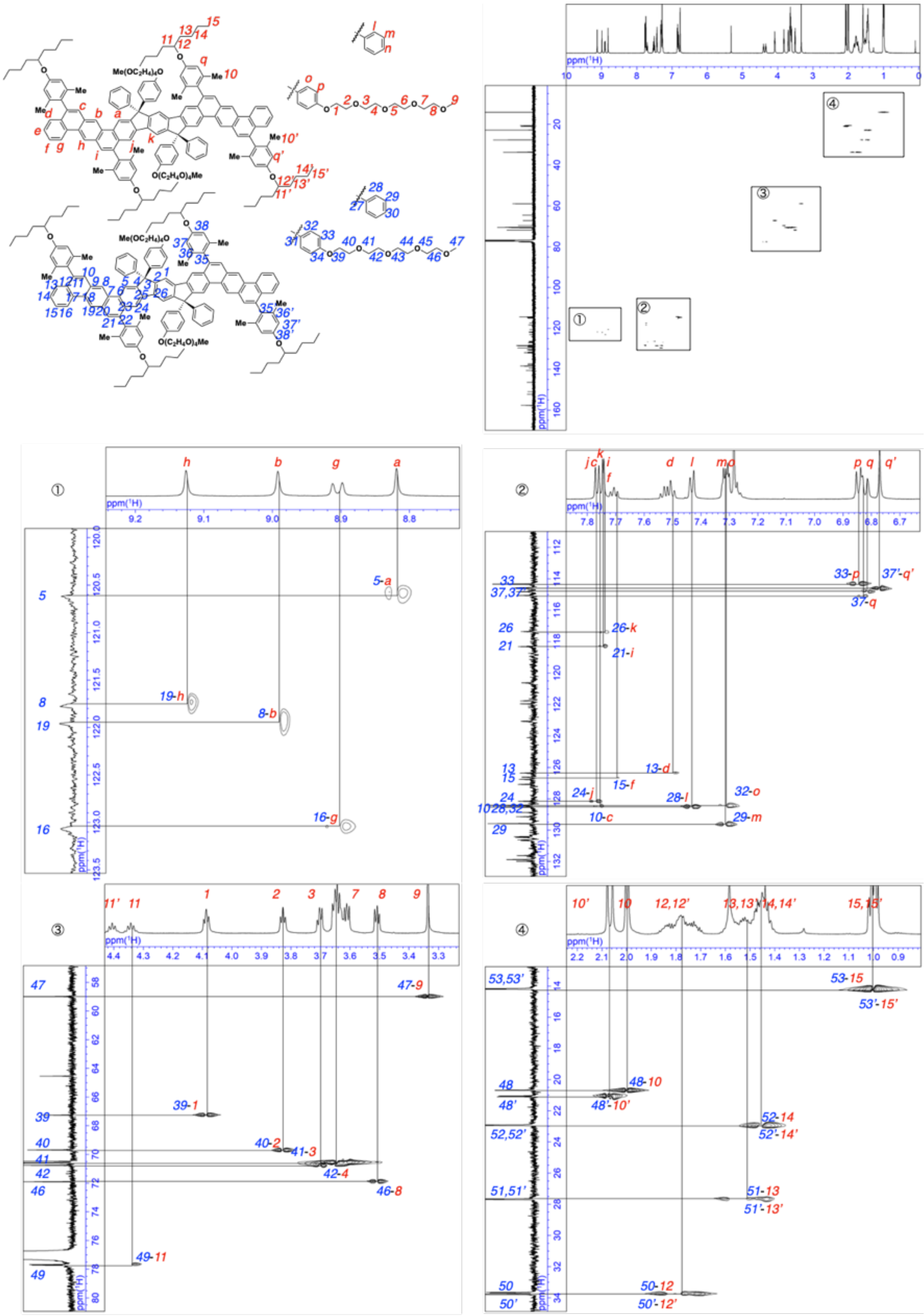

Supplementary Fig. 96 | HSQC correlation of Model-LP-*meso*-1 (600 MHz for ^{1}H and 151 MHz for ^{13}C, $CDCl_3$, 25 ºC)

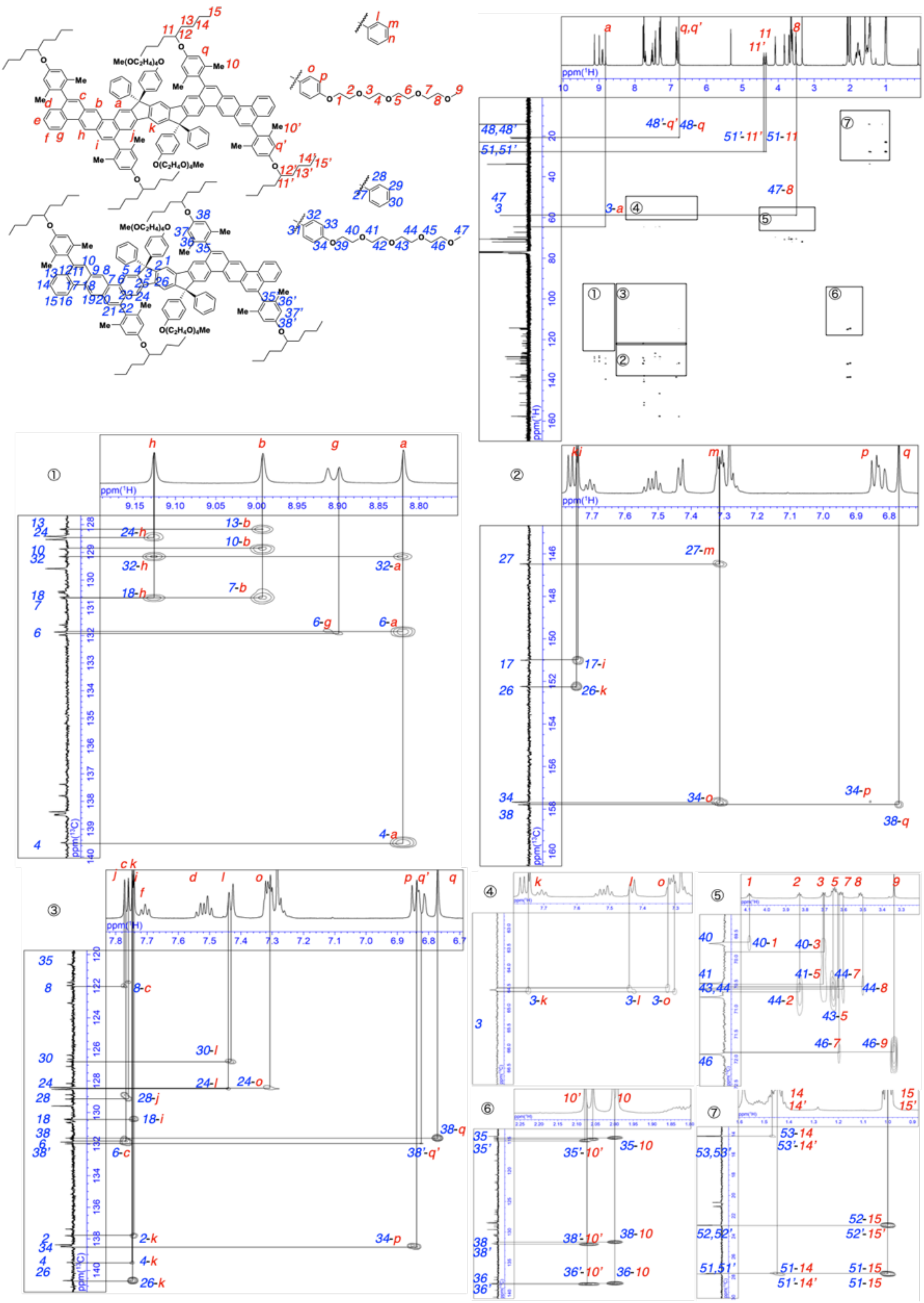

Supplementary Fig. 97 | HMBC correlation of Model-LP-*meso*-1 (600 MHz for ^{1}H and 151 MHz for ^{13}C, $CDCl_3$, 25 ºC).

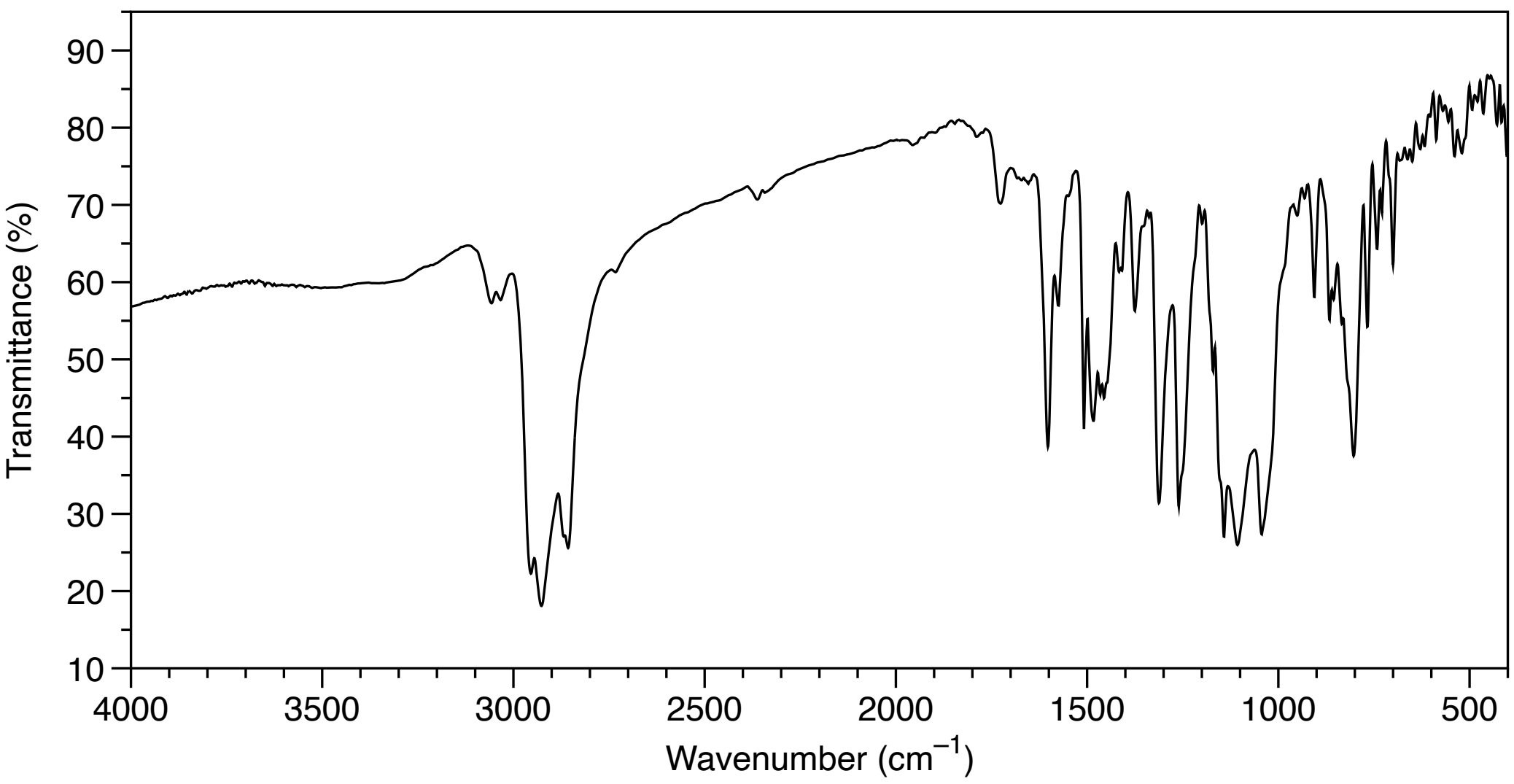

Supplementary Fig. 98 | FT-IR spectrum of Model-LP-*meso*-**1** (KBr, 25 °C).

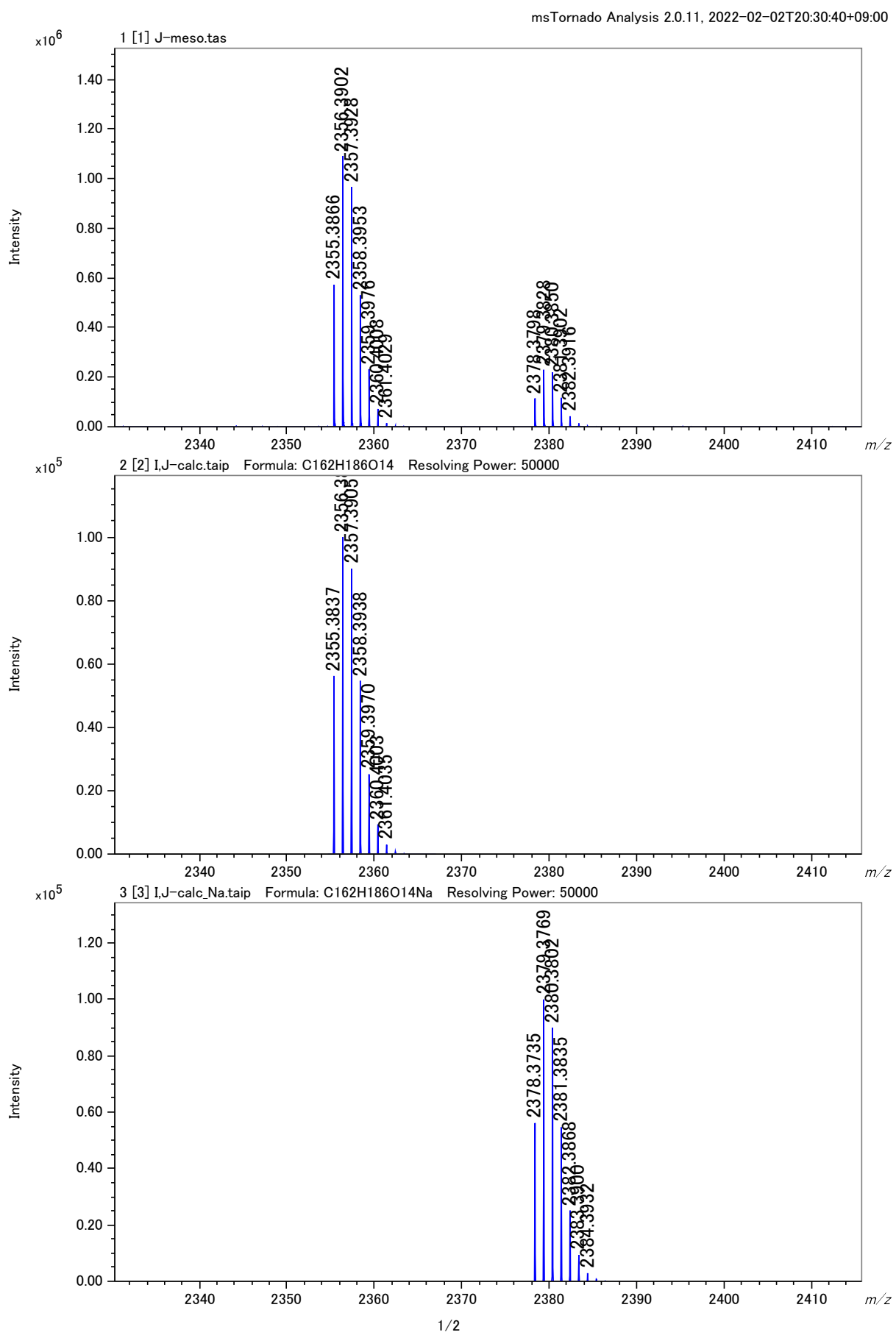

Supplementary Fig. 99 | Observed (upper) and simulated high-resolution MALDI-Spiral-TOF-MS spectrum of Model-LP-*meso*-**1** for $[M]^+$ (middle) and $[M+Na]^+$ (lower).

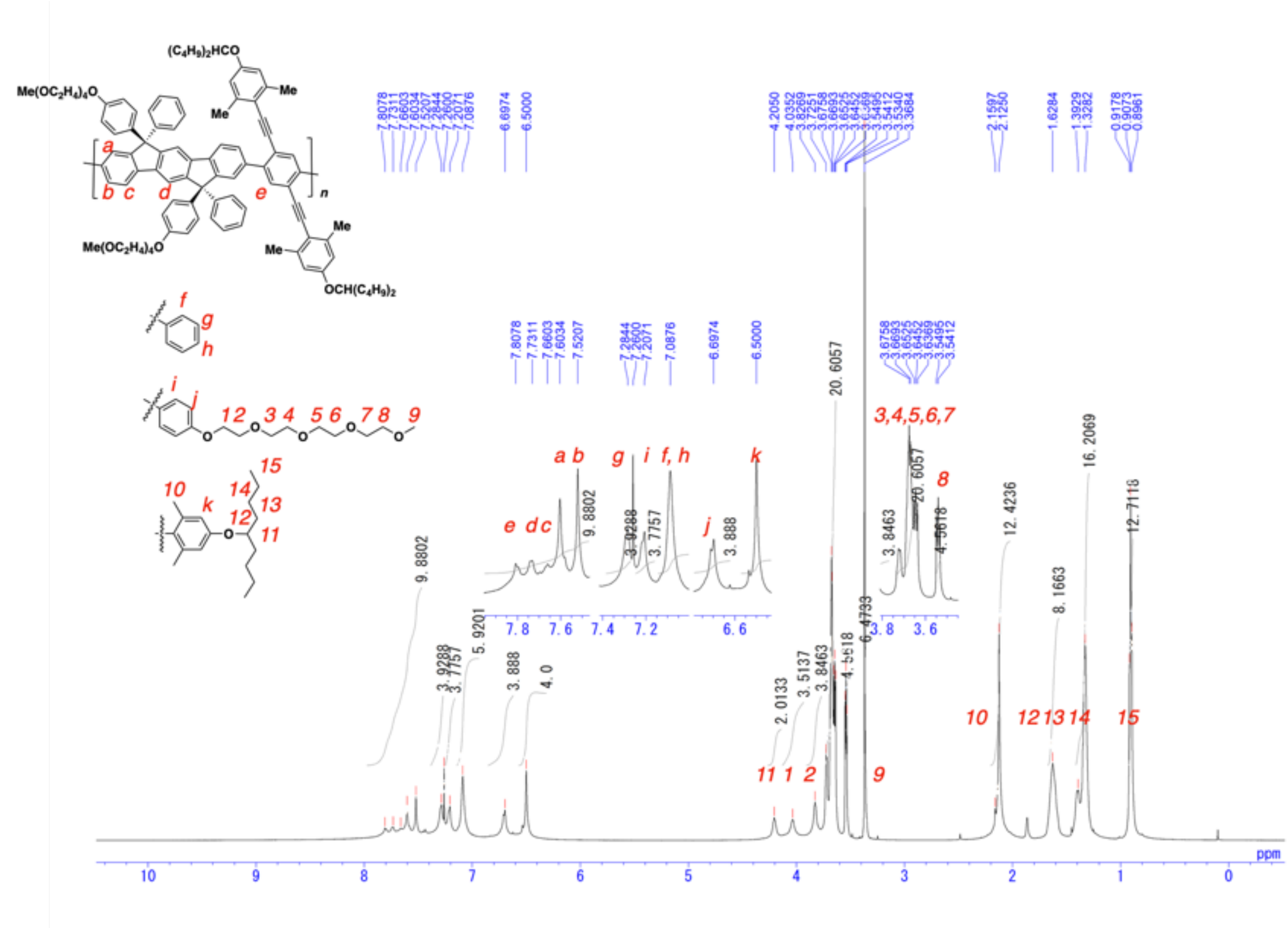

Supplementary Fig. 100 | ^{1}H NMR spectrum of PP-(*S,S*)-**1** (600 MHz, $CDCl_3$, 25 ºC).

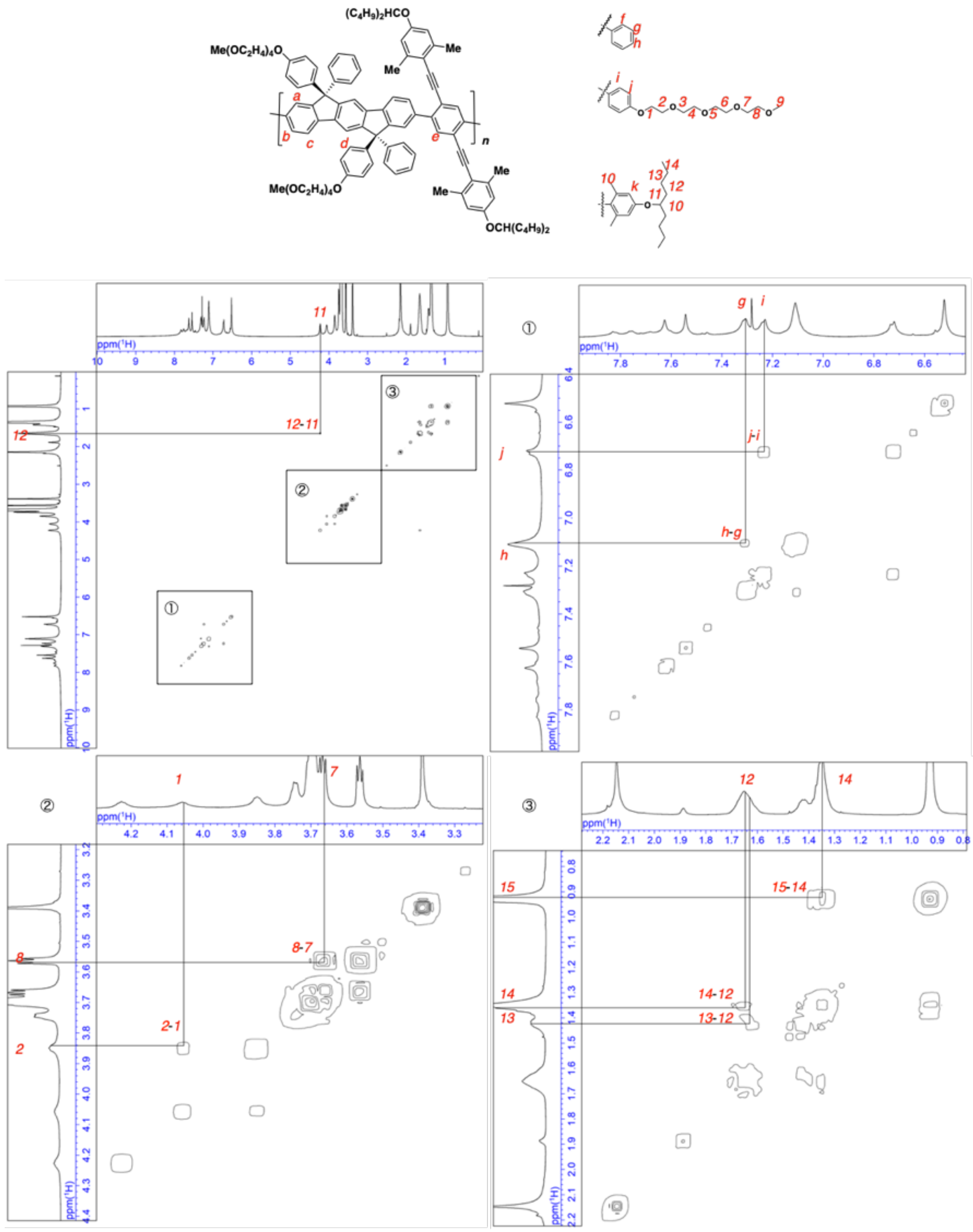

Supplementary Fig. 101 | HH-COSY correlation of PP-(*S,S*)-**1** (600 MHz, $CDCl_3$, 25 ºC).

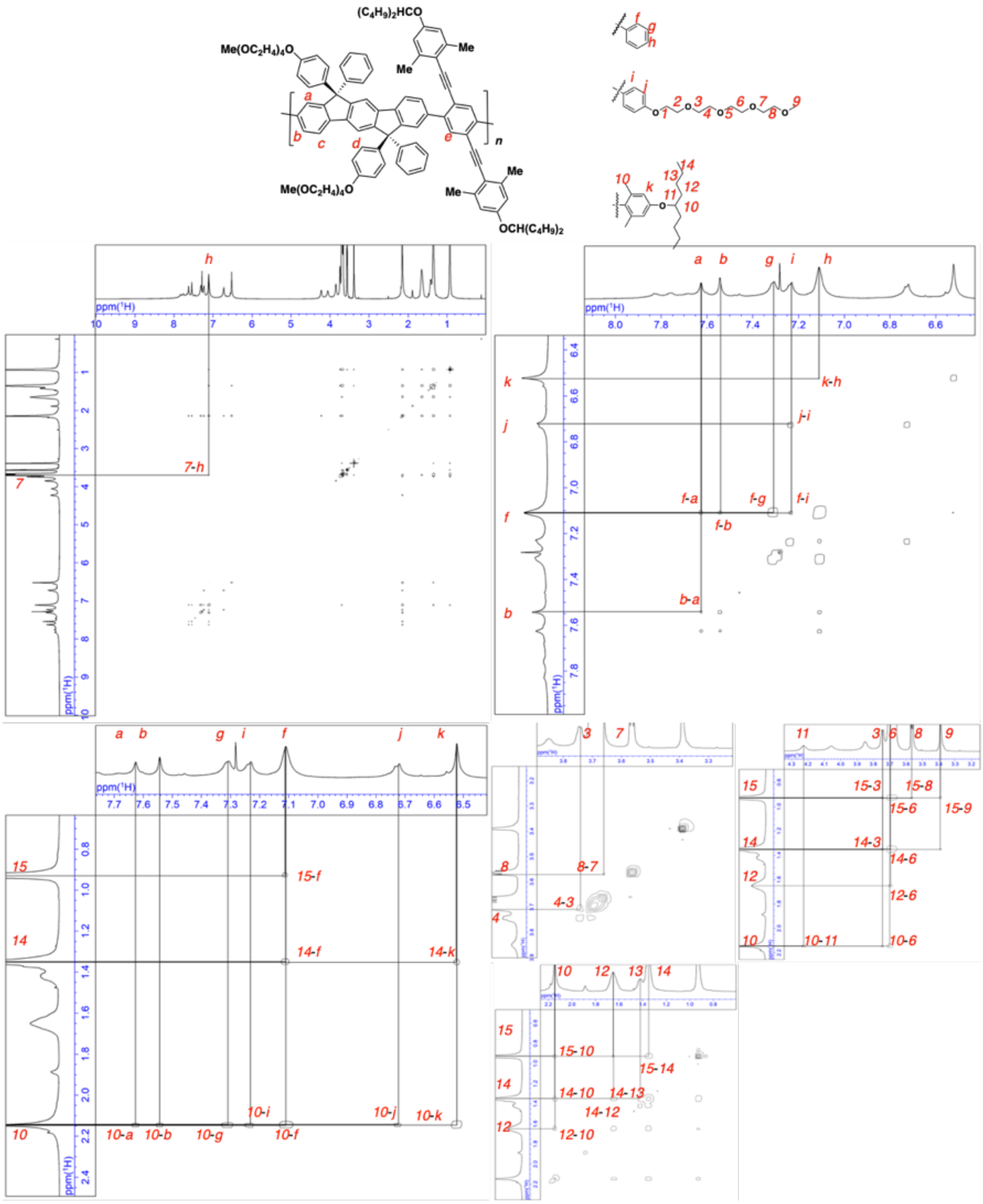

Supplementary Fig. 102 | NOESY correlation of PP-(*S,S*)-**1** (600 MHz, $CDCl_3$, 25 ºC).

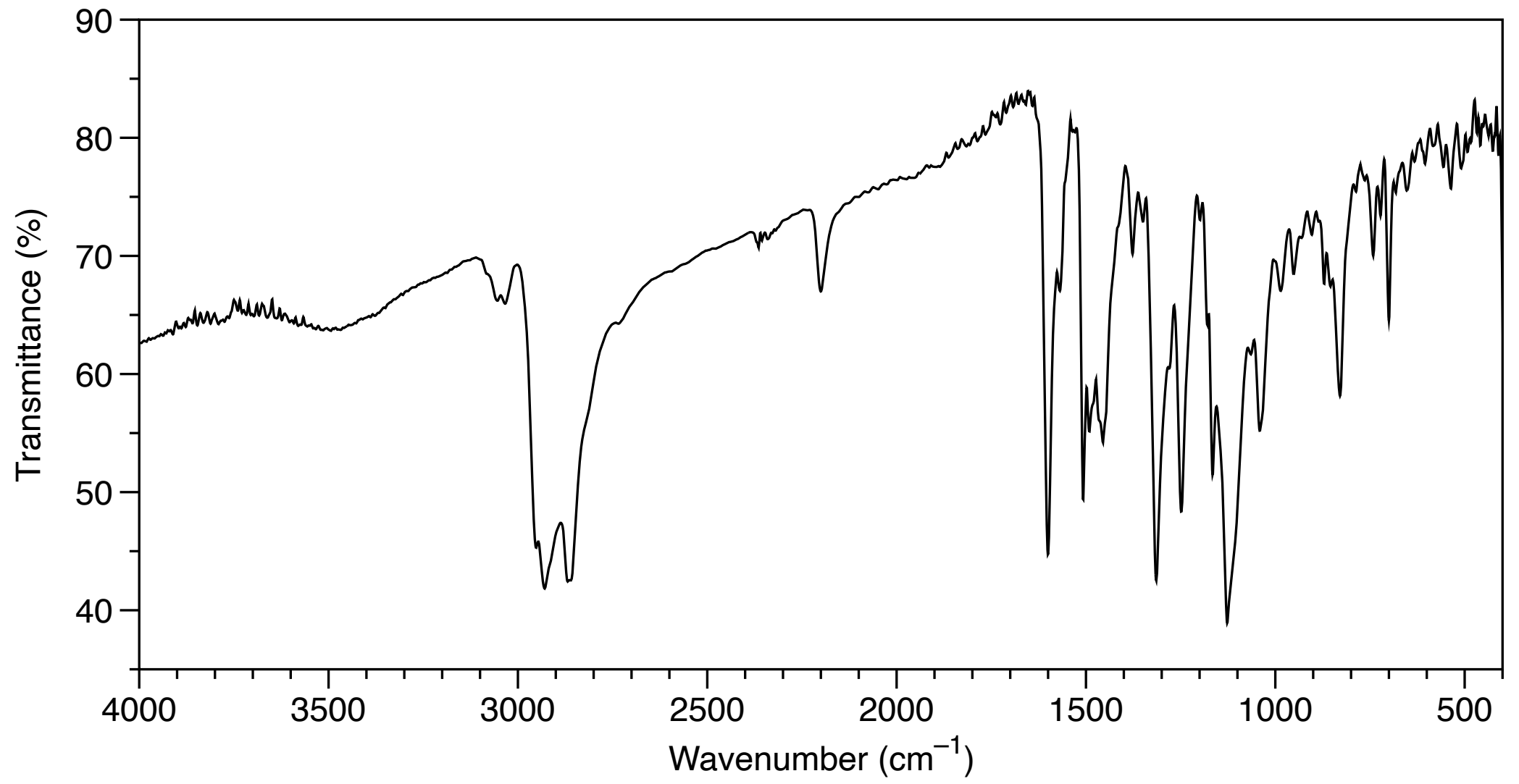

Supplementary Fig. 103 | FT-IR spectrum of PP-(*S,S*)-**1** (KBr, 25 °C).

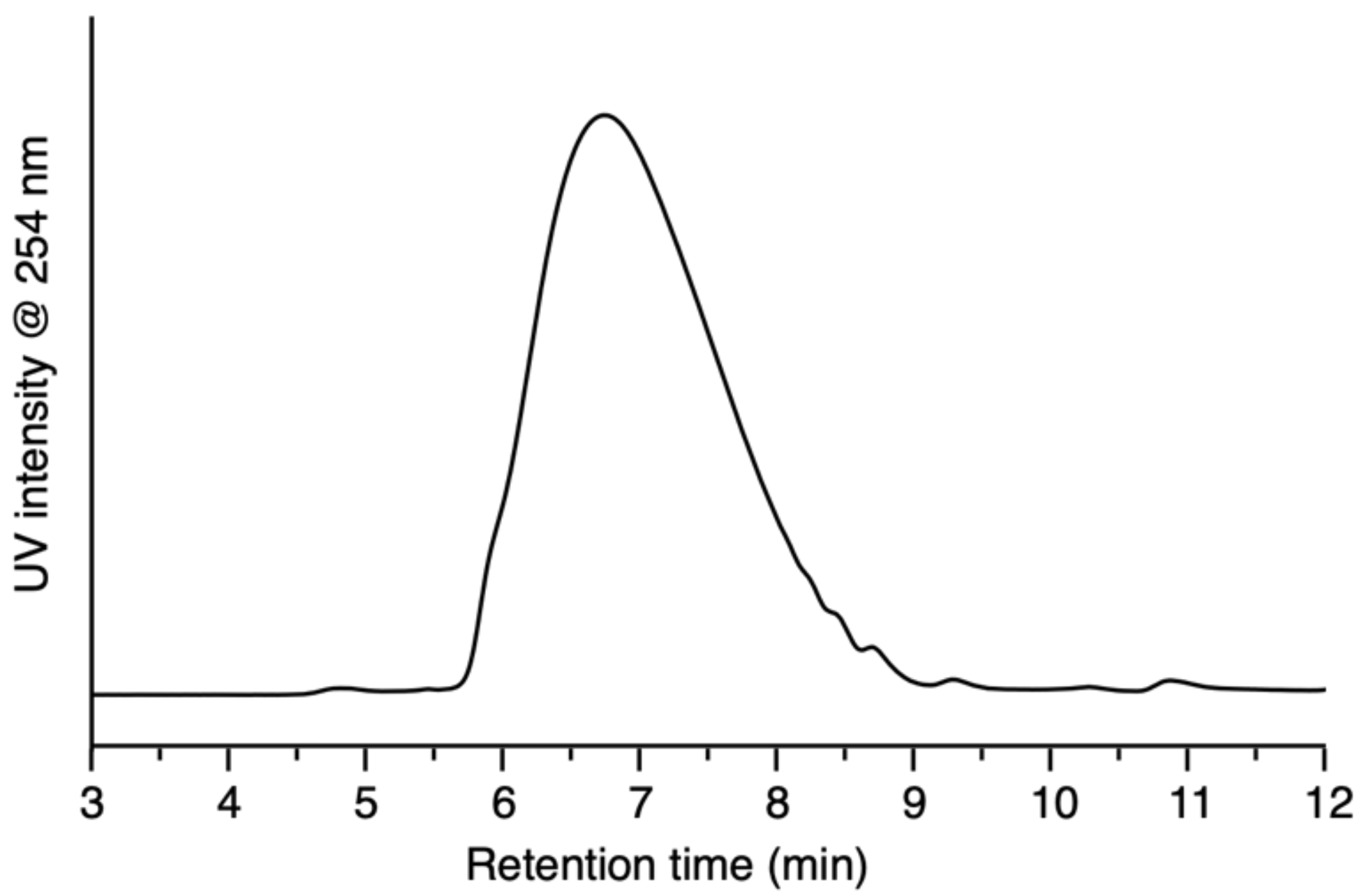

Supplementary Fig. 104 | SEC profile of PP-(*S,S*)-**1** ($CHCl_3$).

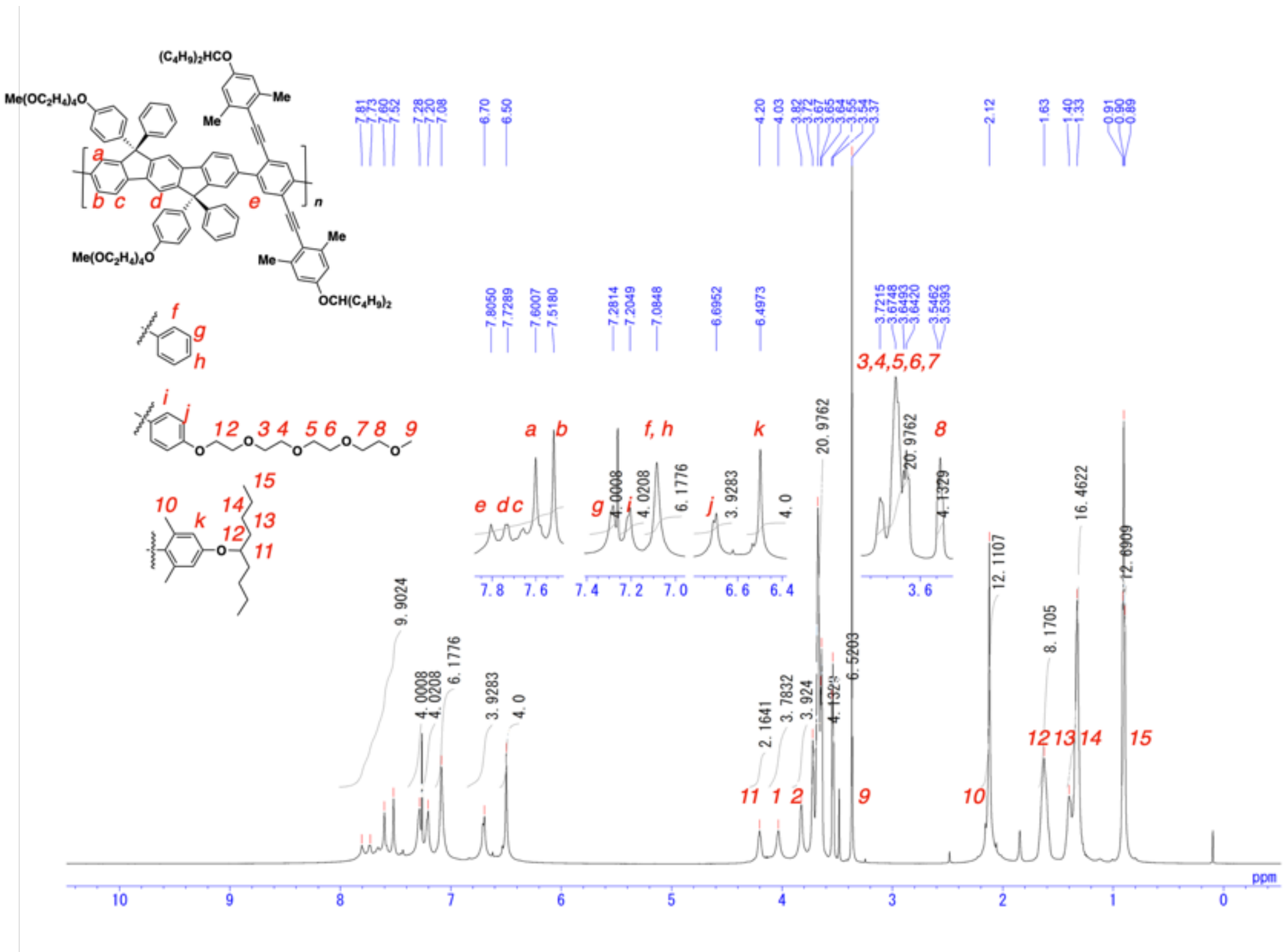

Supplementary Fig. 105 | ^{1}H NMR spectrum of PP-(*R,R*)-**1** (600 MHz, $CDCl_3$, 25 ºC).

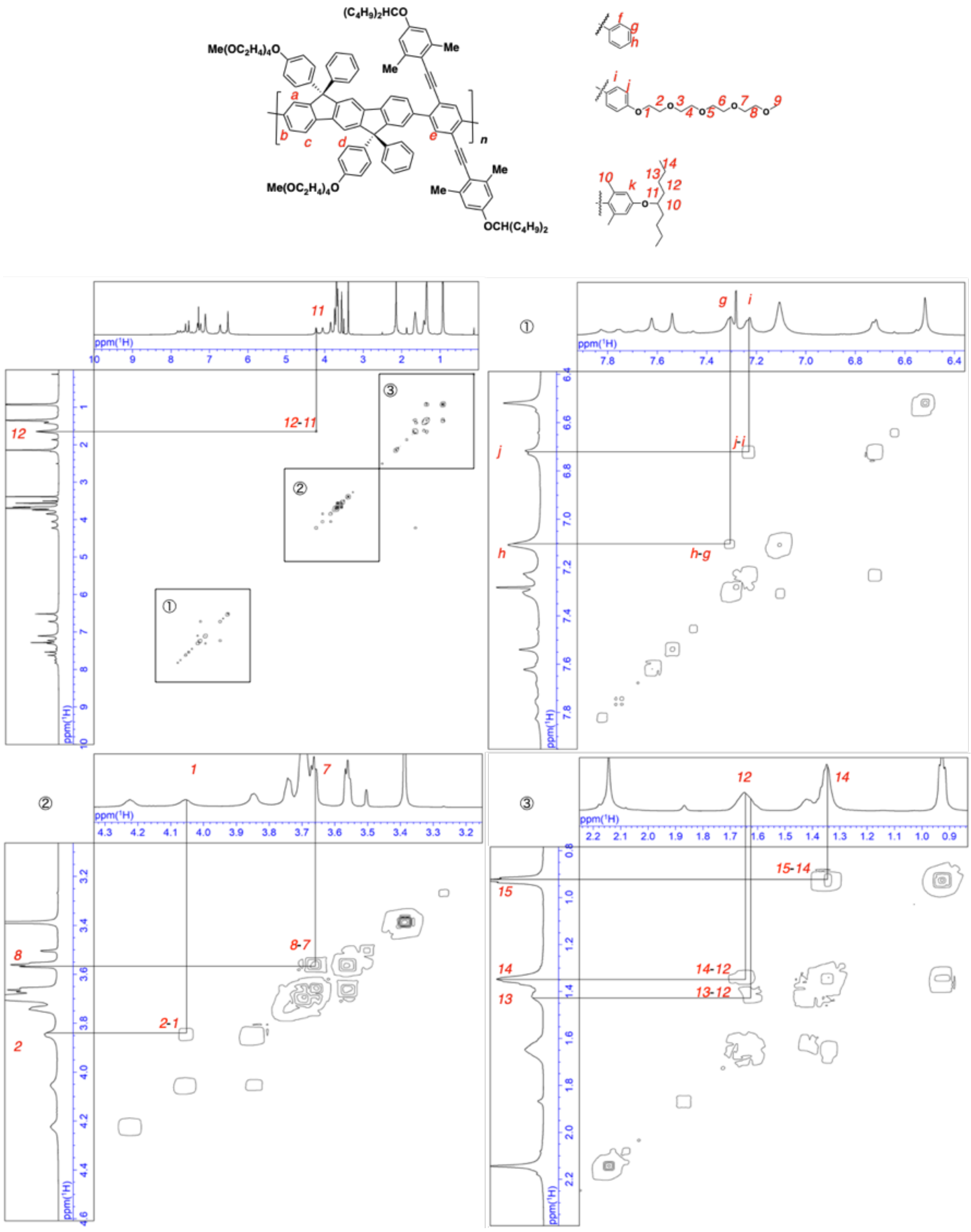

Supplementary Fig. 106 | HH-COSY correlation of PP-(*R,R*)-**1** (600 MHz, $CDCl_3$, 25 ºC).

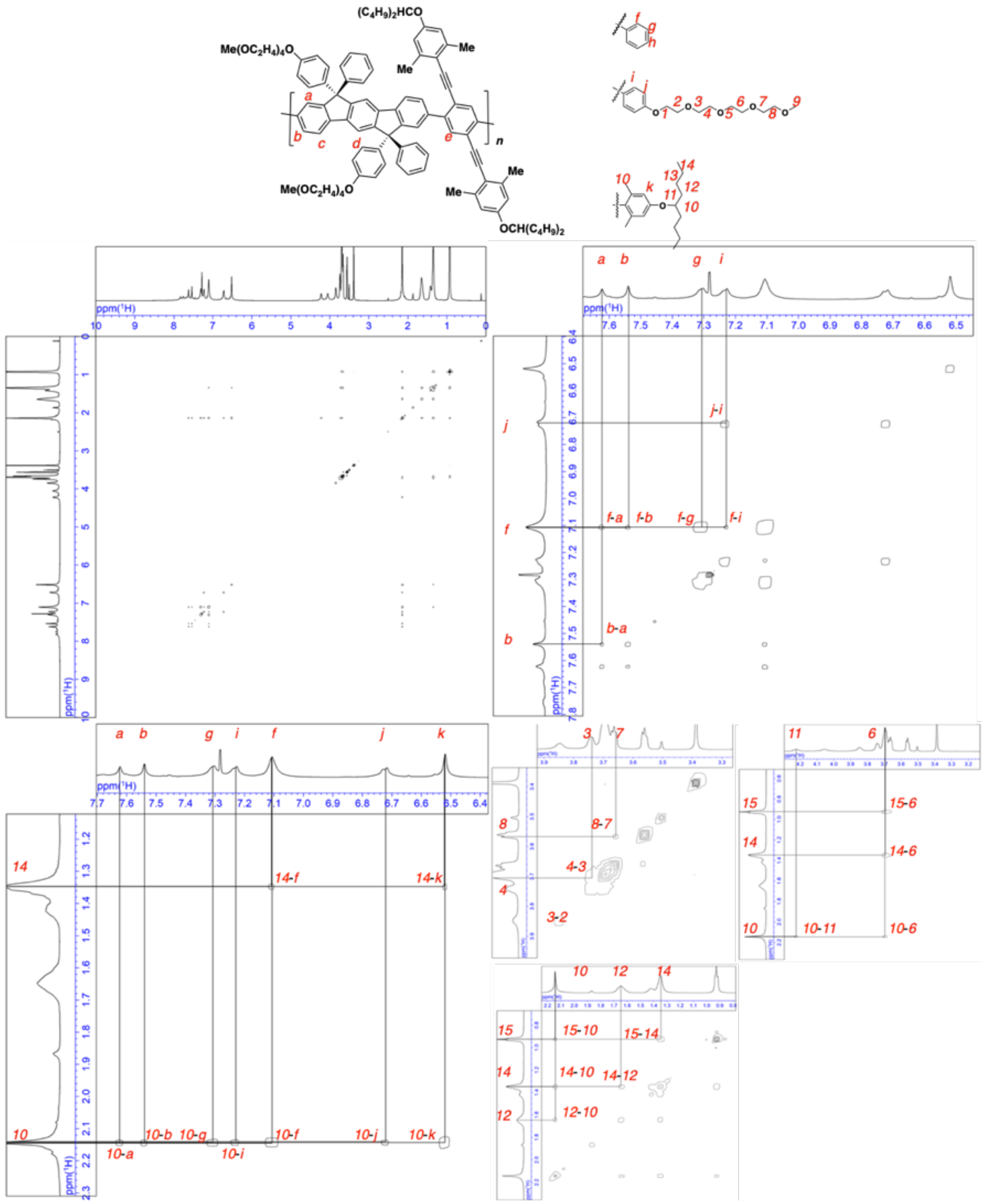

Supplementary Fig. 107 | NOESY correlation of PP-(*R,R*)-**1** (600 MHz, $CDCl_3$, 25 ºC).

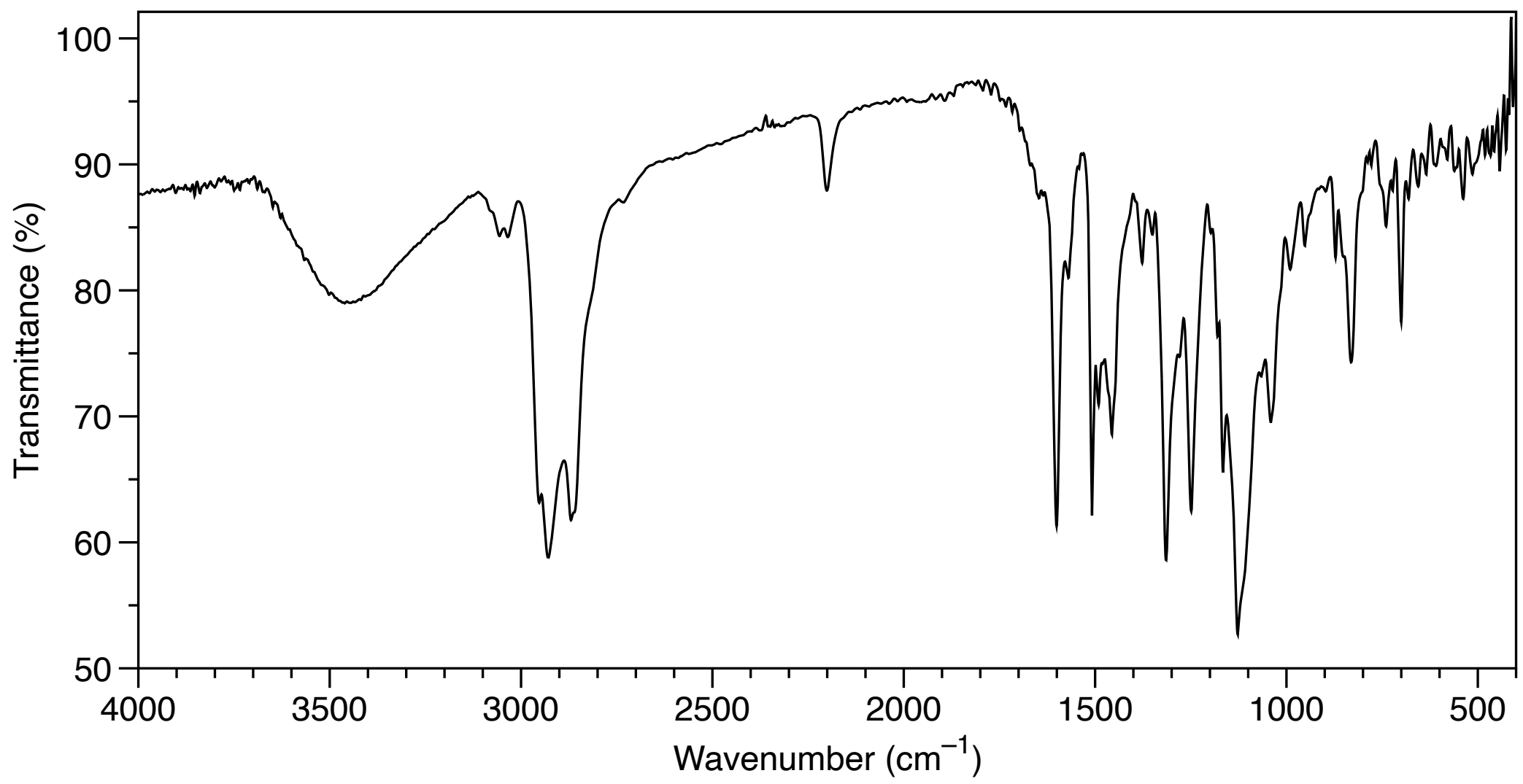

Supplementary Fig. 108 | FT-IR spectrum of PP-(*R,R*)-**1** (KBr, 25 °C).

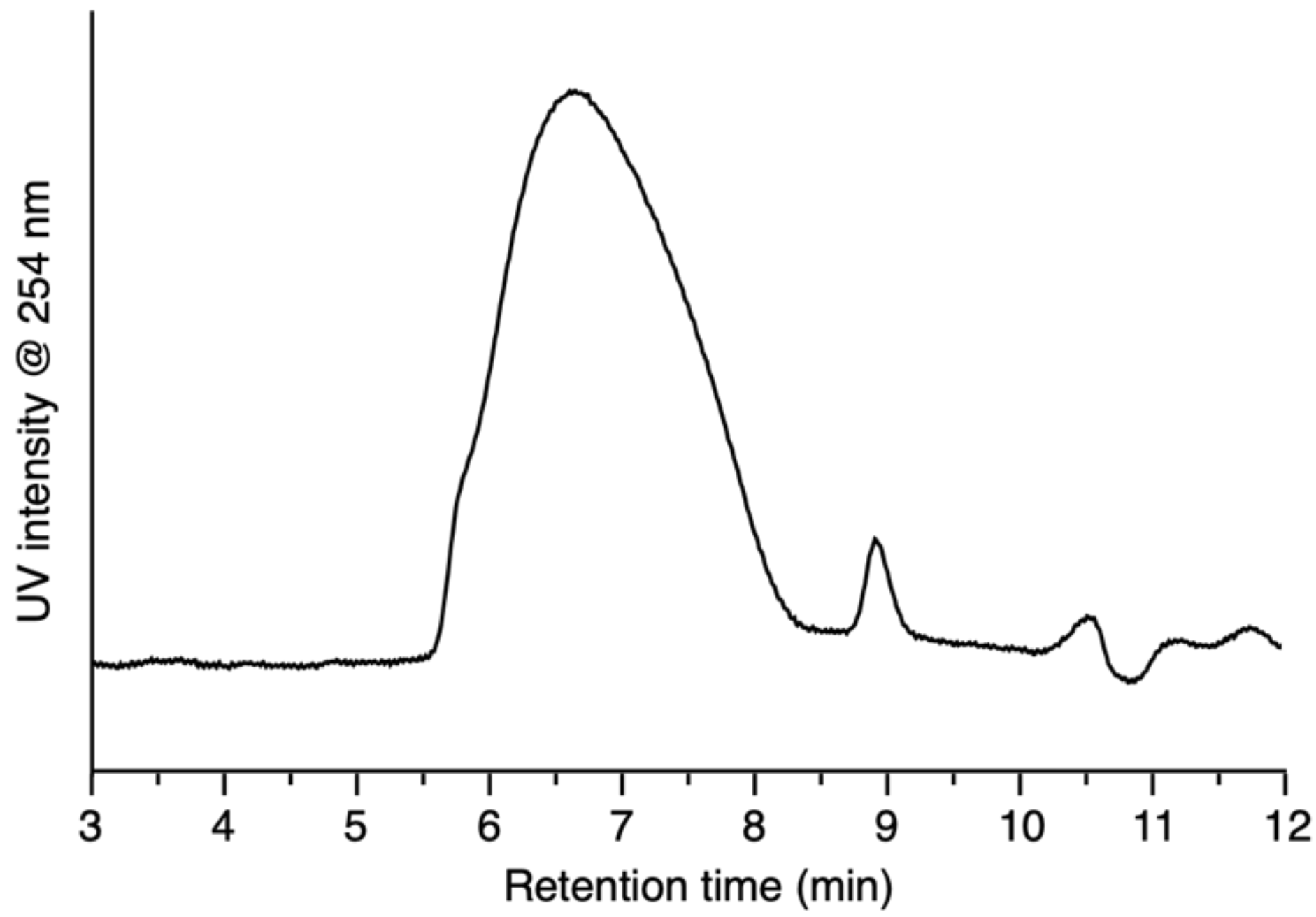

Supplementary Fig. 109 | SEC profile of PP-(*R,R*)-**1** ($CHCl_3$).

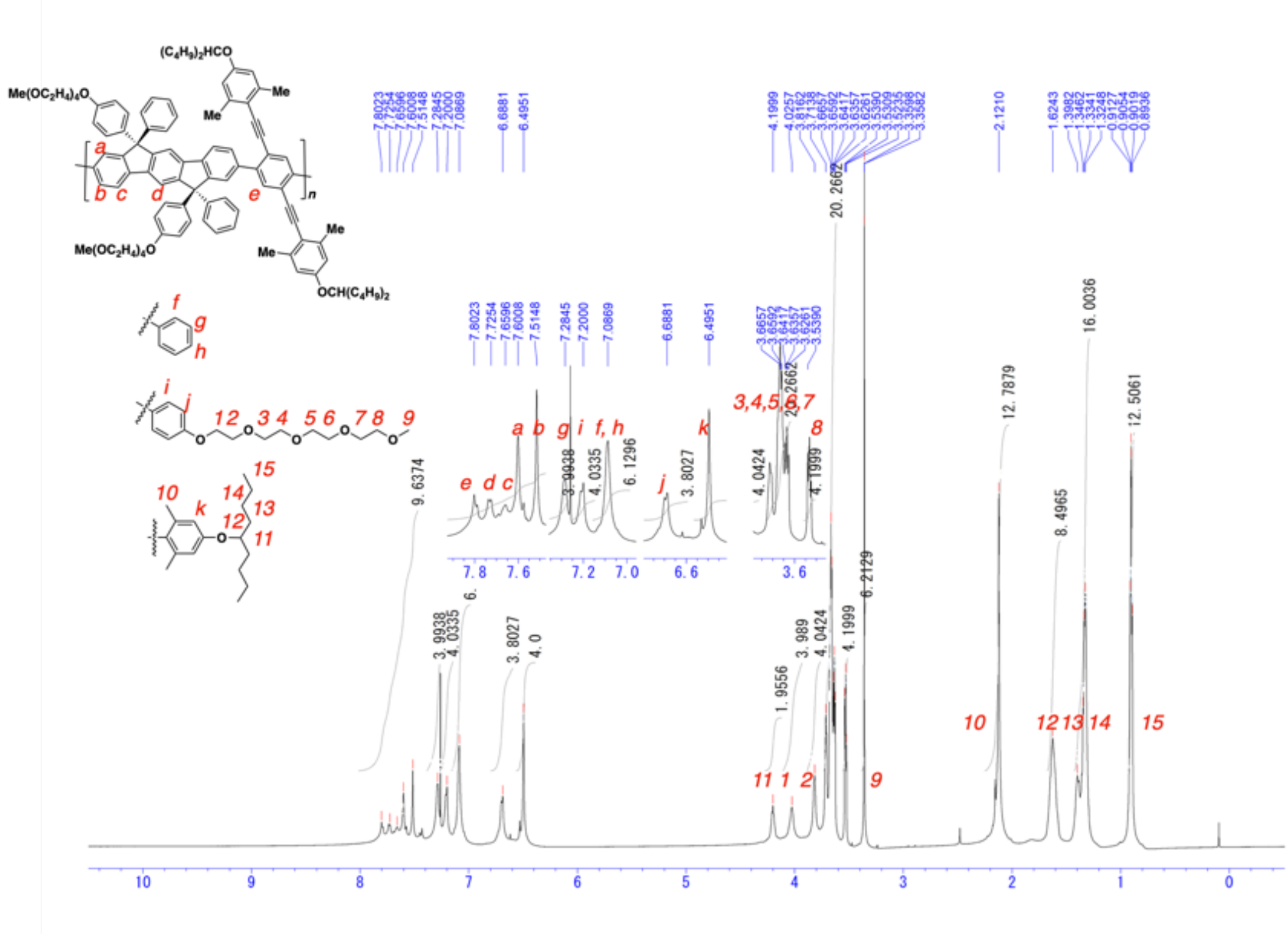

Supplementary Fig. 110 | ^{1}H NMR spectrum of PP-*meso*-**1** (600 MHz, $CDCl_3$, 25 ºC).

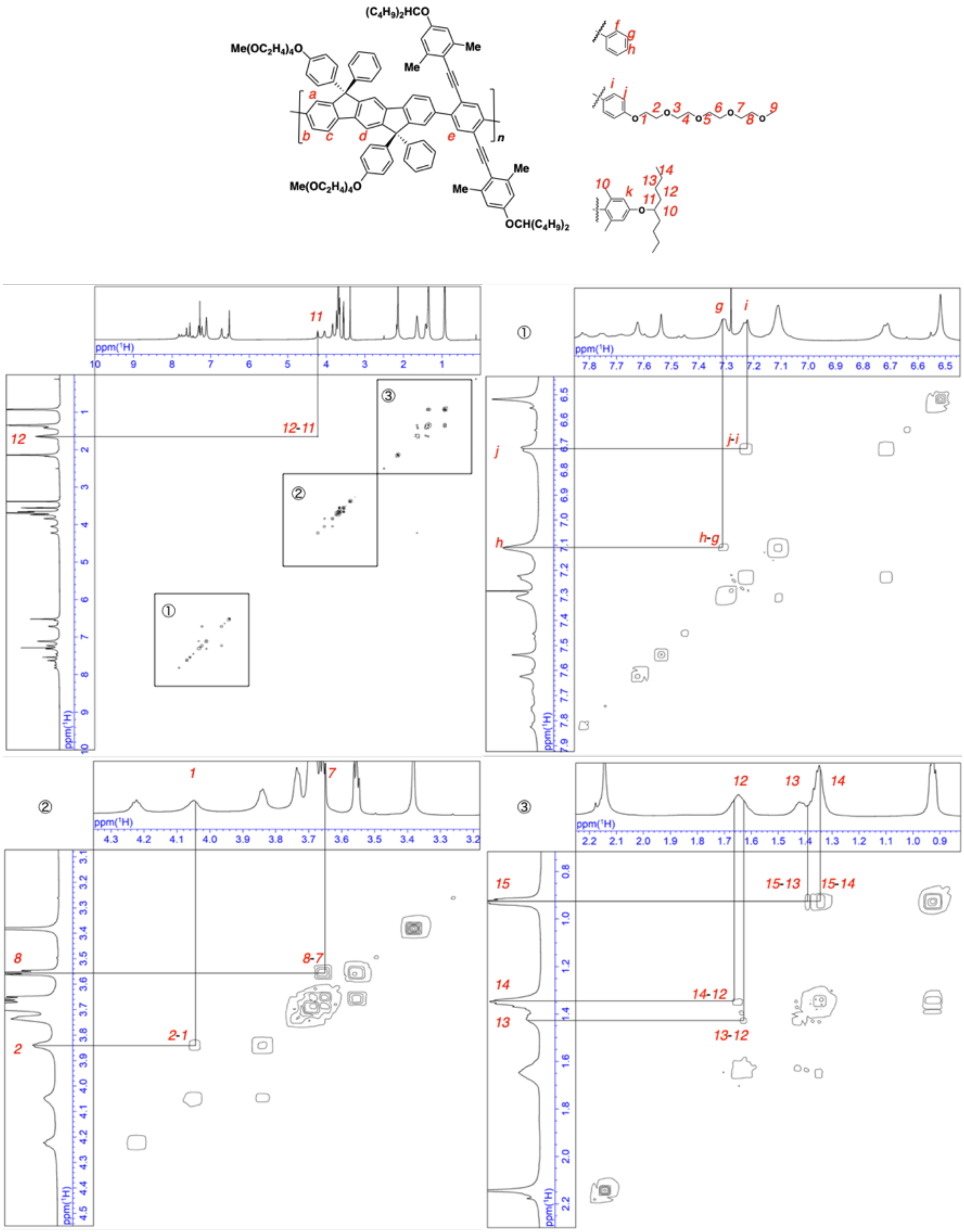

Supplementary Fig. 111 | HH-COSY correlation of PP-*meso*-**1** (600 MHz, $CDCl_3$, 25 ºC).

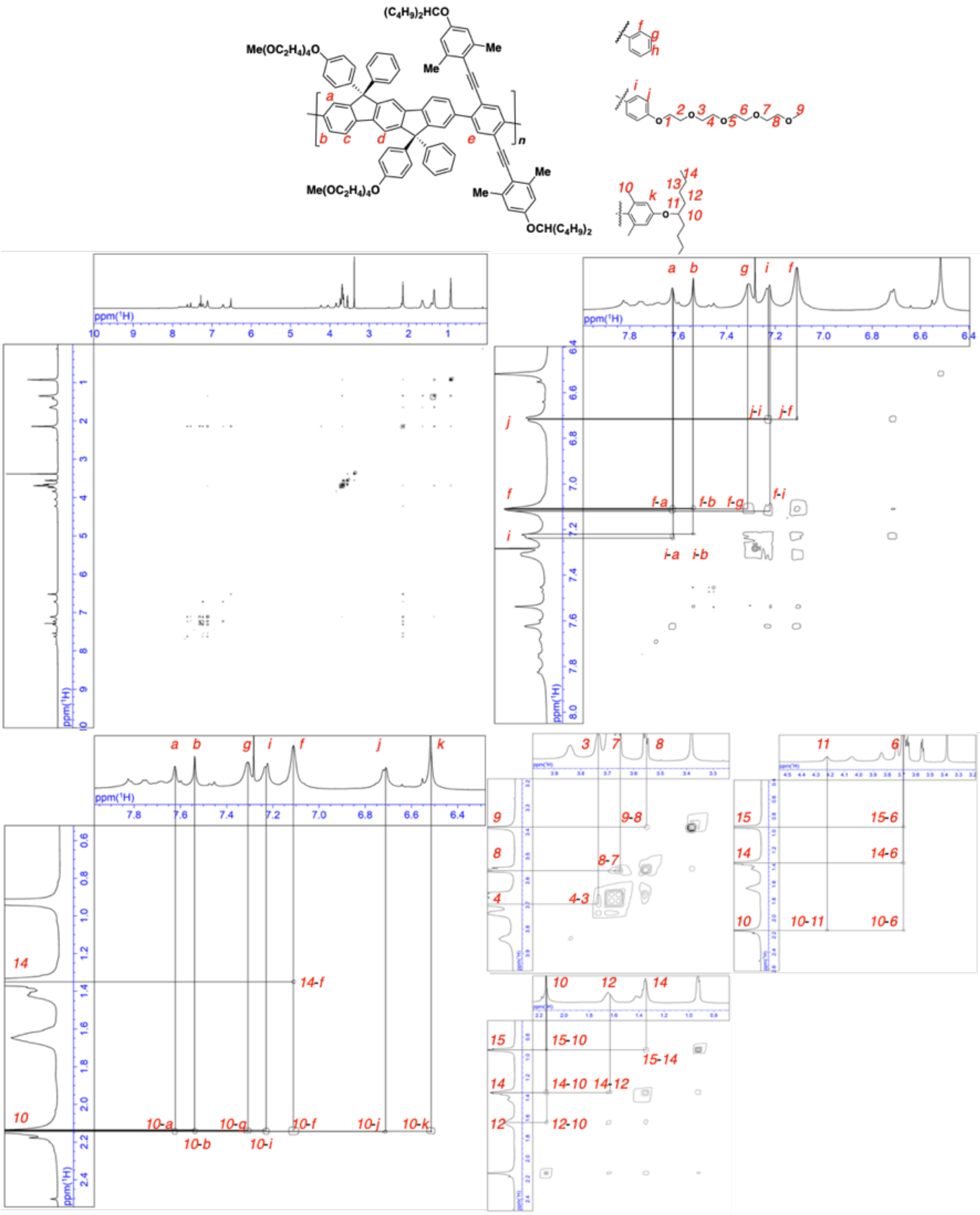

Supplementary Fig. 112 | NOSY correlation of PP-*meso*-**1** (600 MHz, $CDCl_3$, 25 ºC).

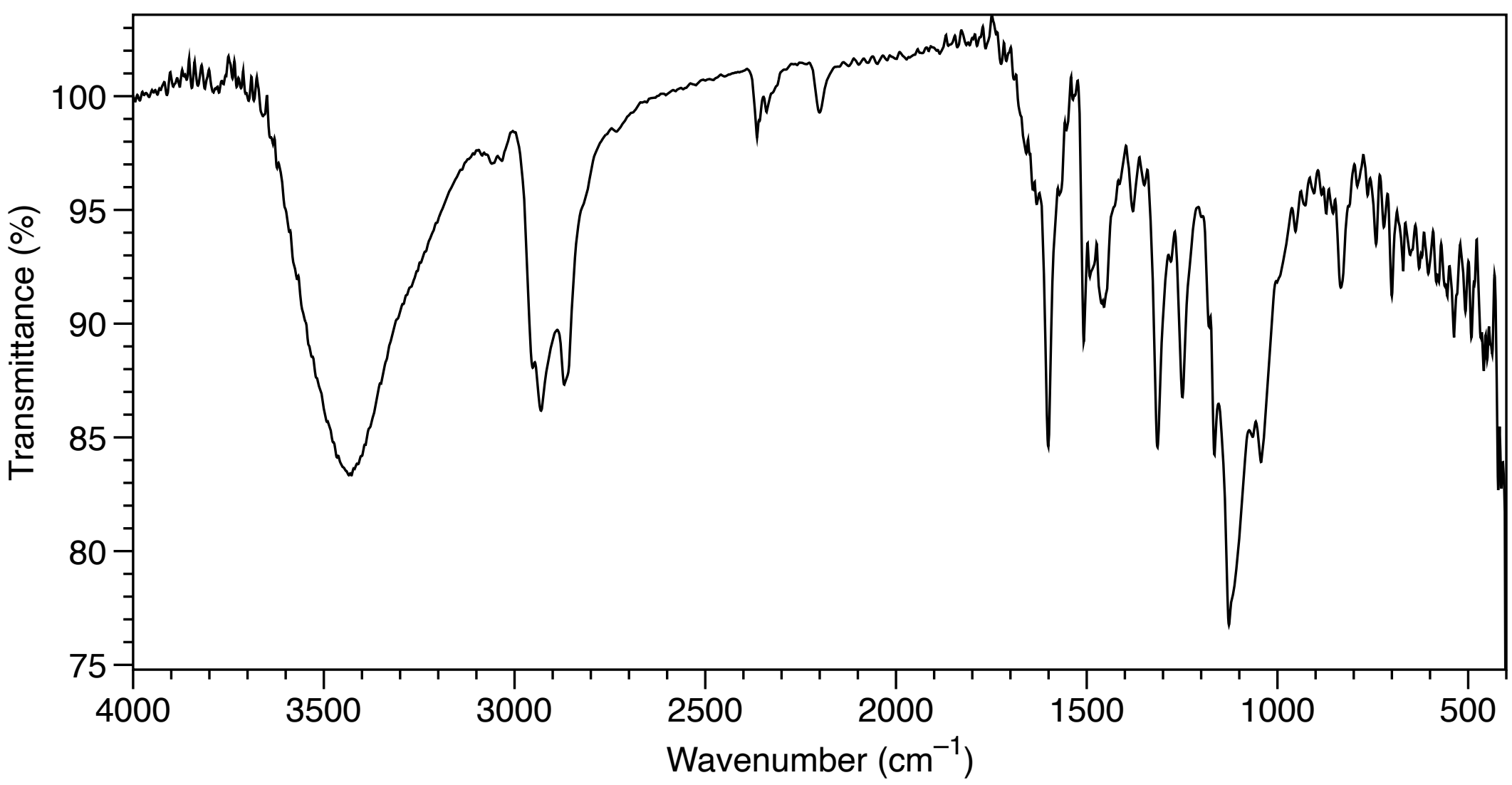

Supplementary Fig. 113 | FT-IR spectrum of PP-*meso*-**1** (KBr, 25 °C).

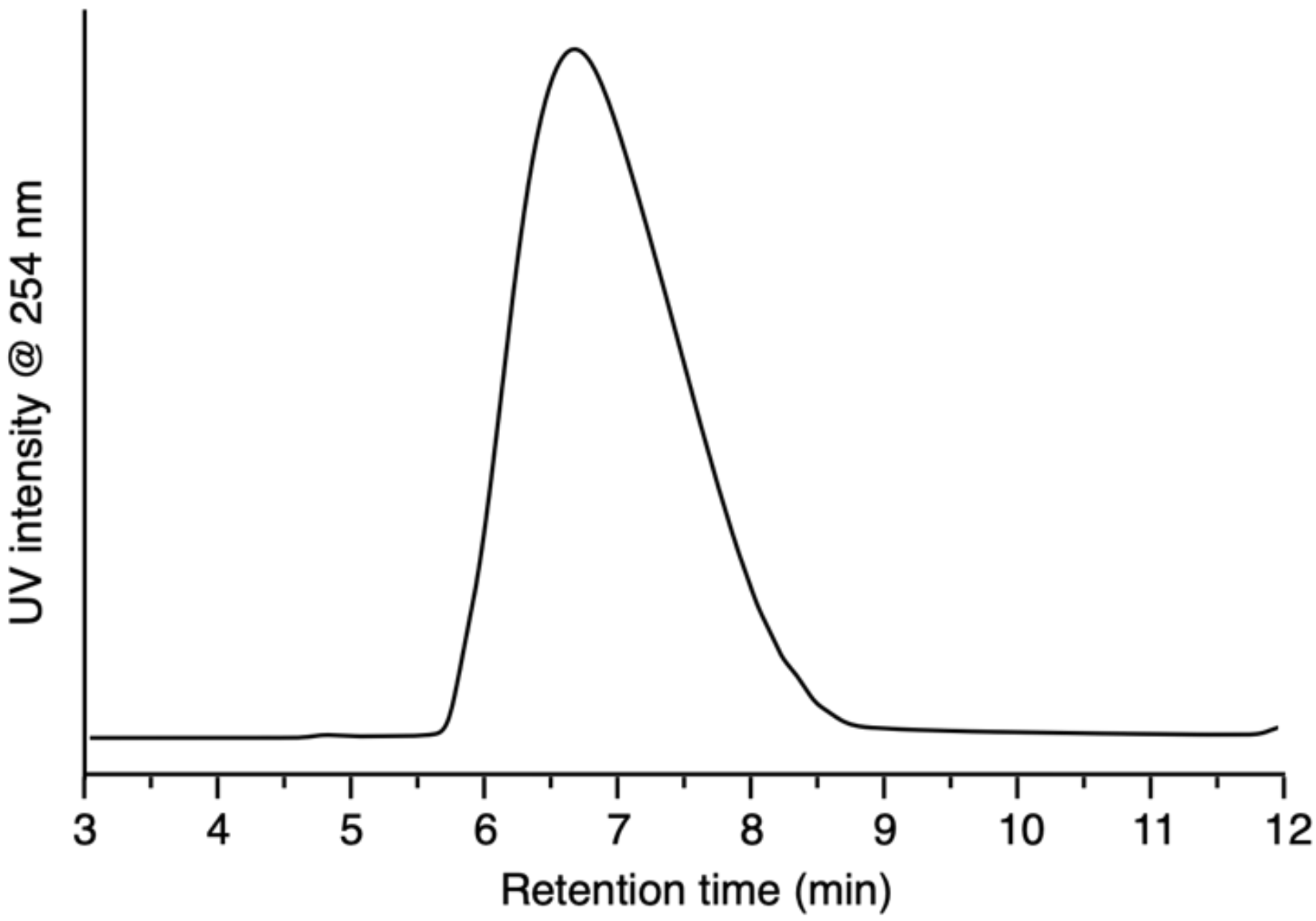

Supplementary Fig. 114 | SEC profile of PP-*meso*-**1** ($CHCl_3$).

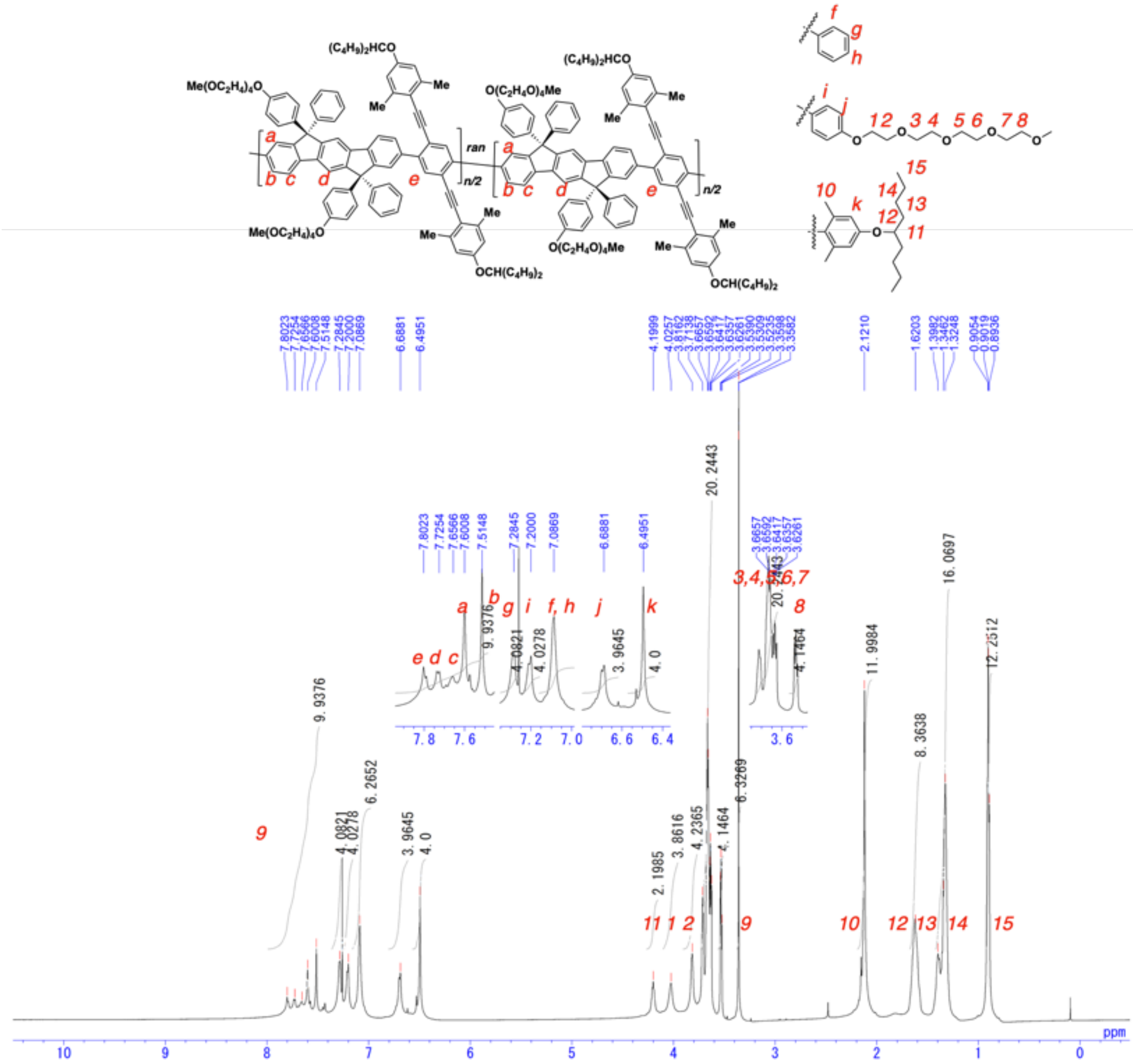

Supplementary Fig. 115 | ^{1}H NMR spectrum of PP-*rac*-**1** (600 MHz, $CDCl_3$, 25 ºC).

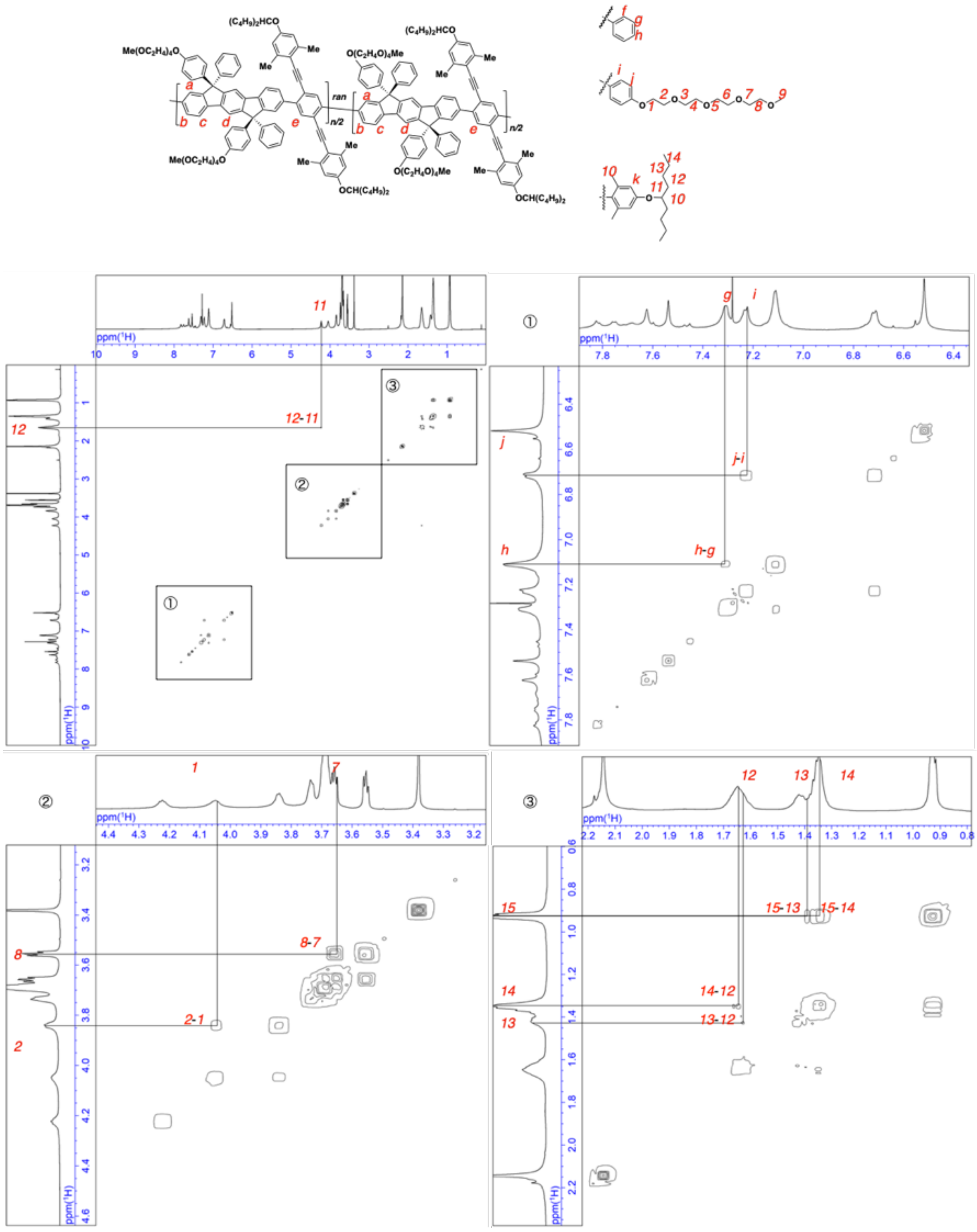

Supplementary Fig. 116 | HH-COSY correlation of PP-*rac*-**1** (600 MHz, $CDCl_3$, 25 ºC).

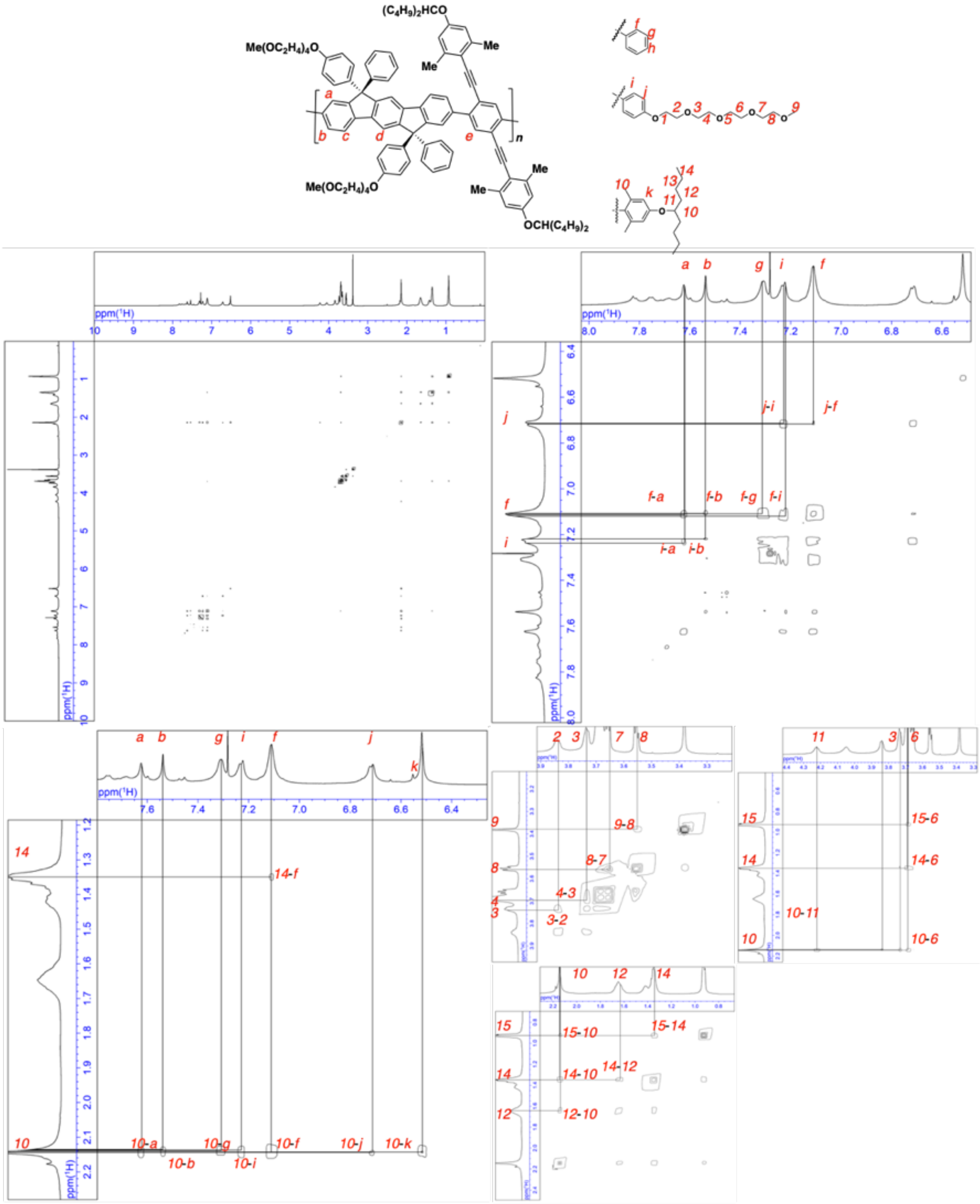

Supplementary Fig. 117 | NOESY correlation of PP-*rac*-**1** (600 MHz, $CDCl_3$, 25 ºC).

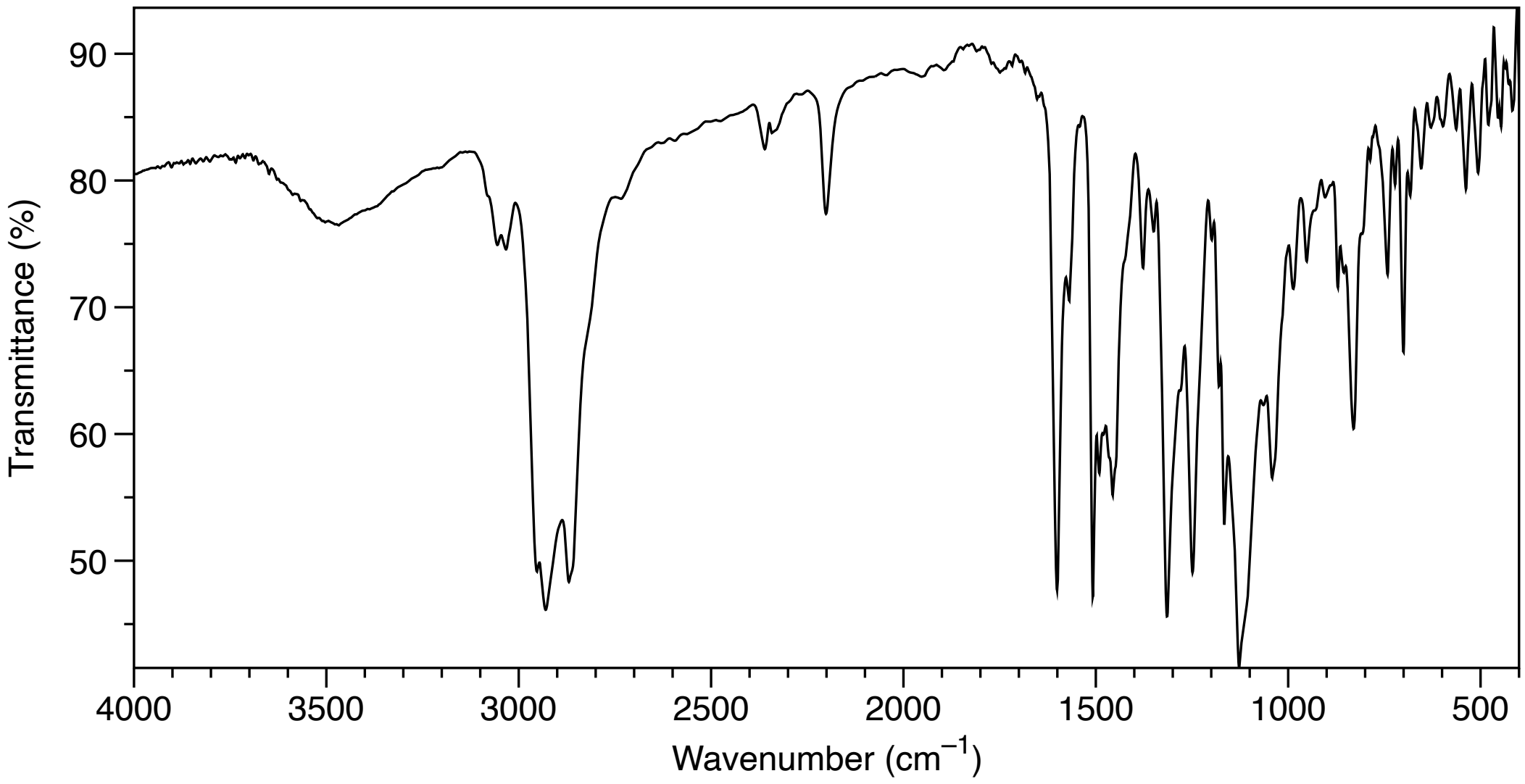

Supplementary Fig. 118 | FT-IR spectrum of PP-*rac*-**1** (KBr, 25 °C).

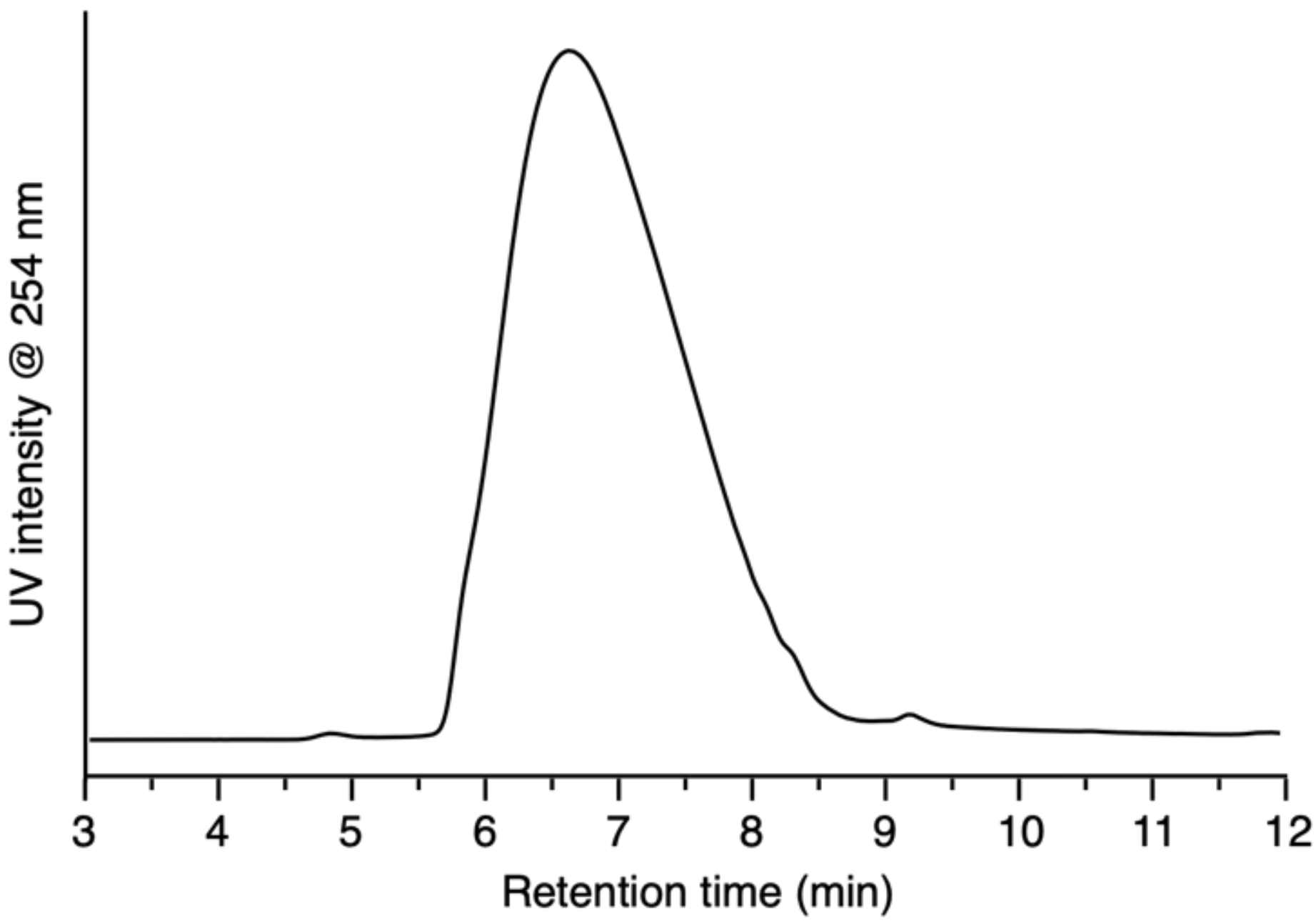

Supplementary Fig. 119 | SEC profile of PP-*rac*-**1** ($CHCl_3$).

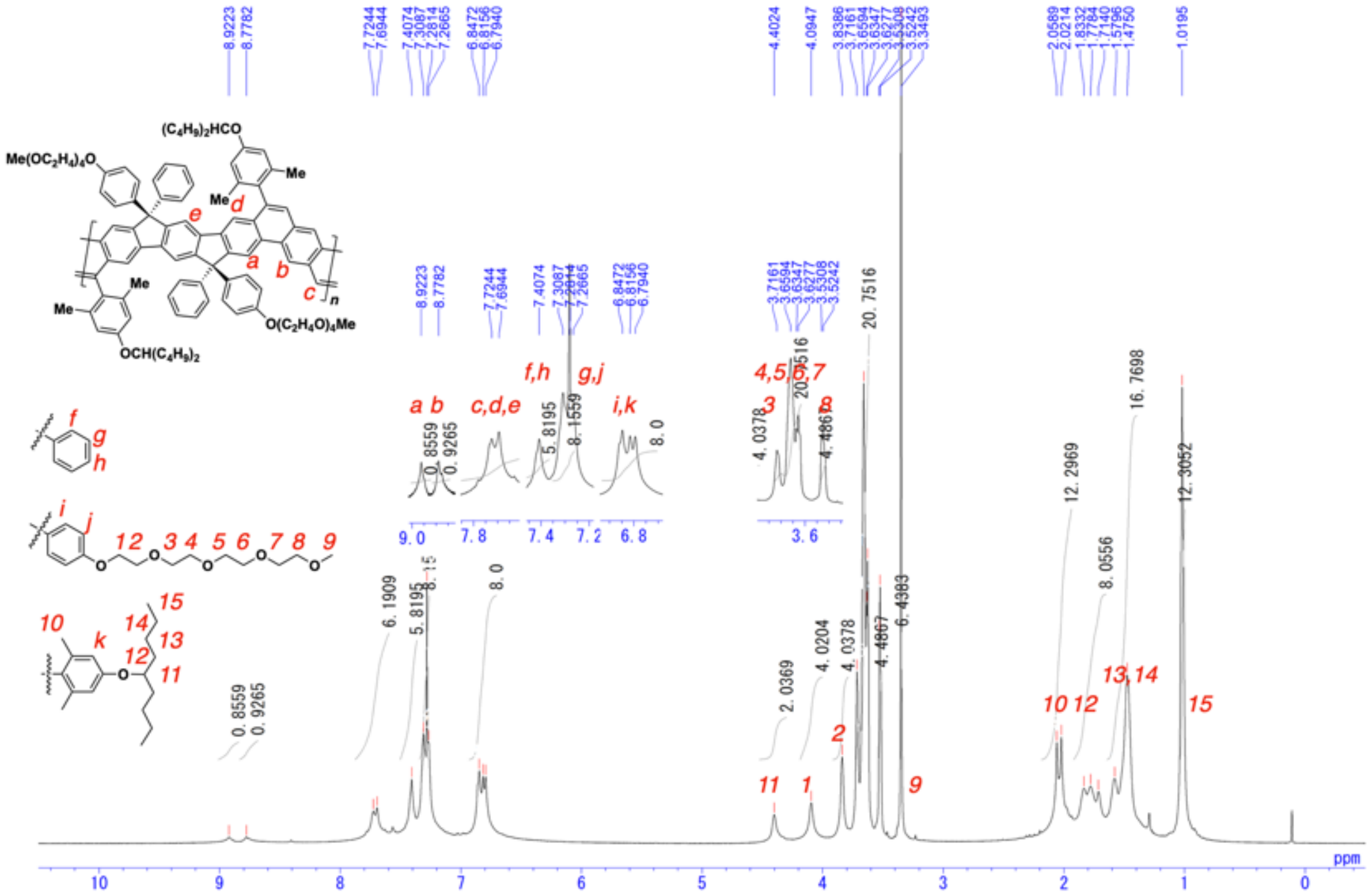

Supplementary Fig. 120 | ^{1}H NMR spectrum of LP-(*S,S*)-**1** (600 MHz, $CDCl_3$, 25 ºC).

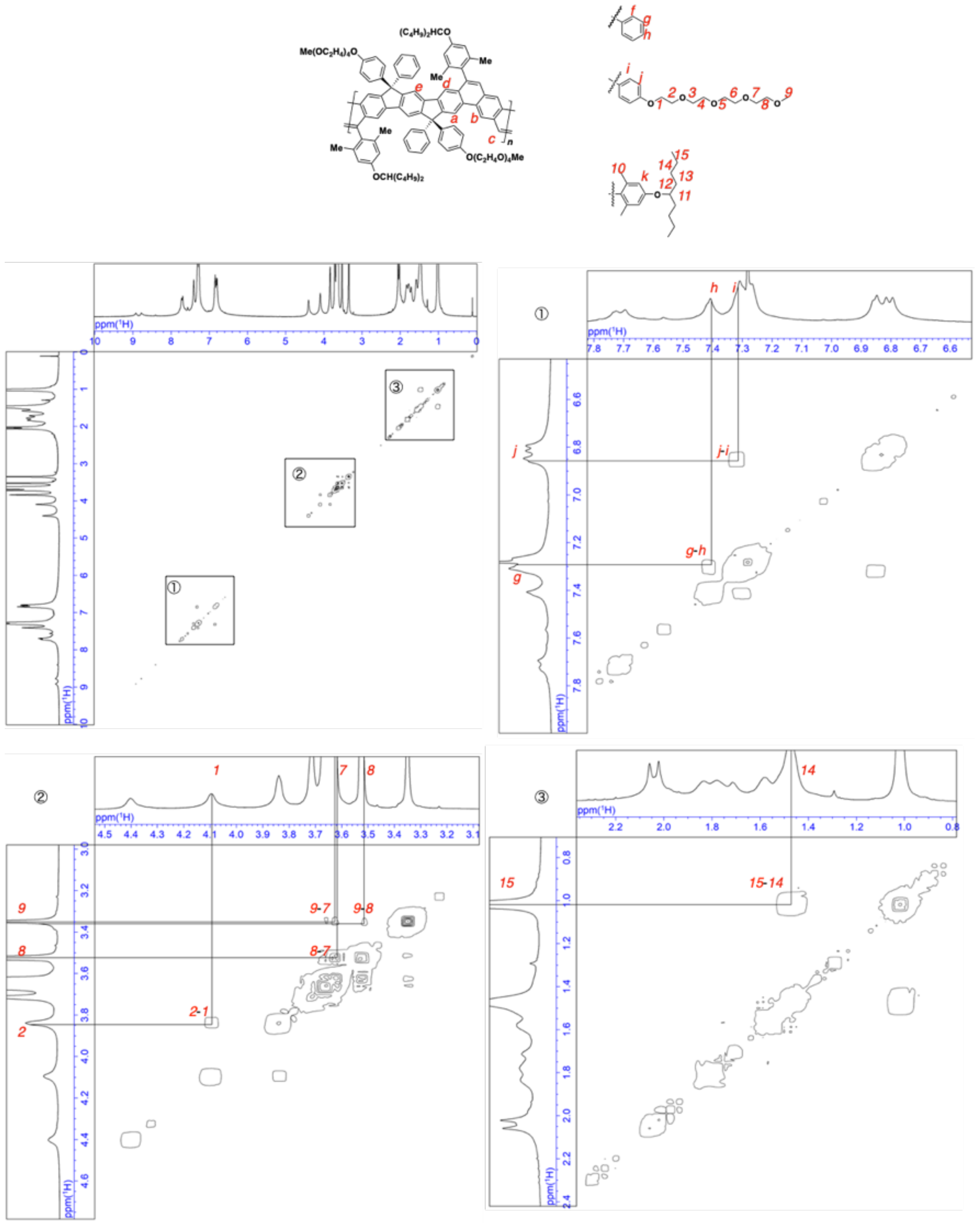

Supplementary Fig. 121 | HH-COSY correlation of LP-(*S,S*)-**1** (600 MHz, $CDCl_3$, 25 ºC).

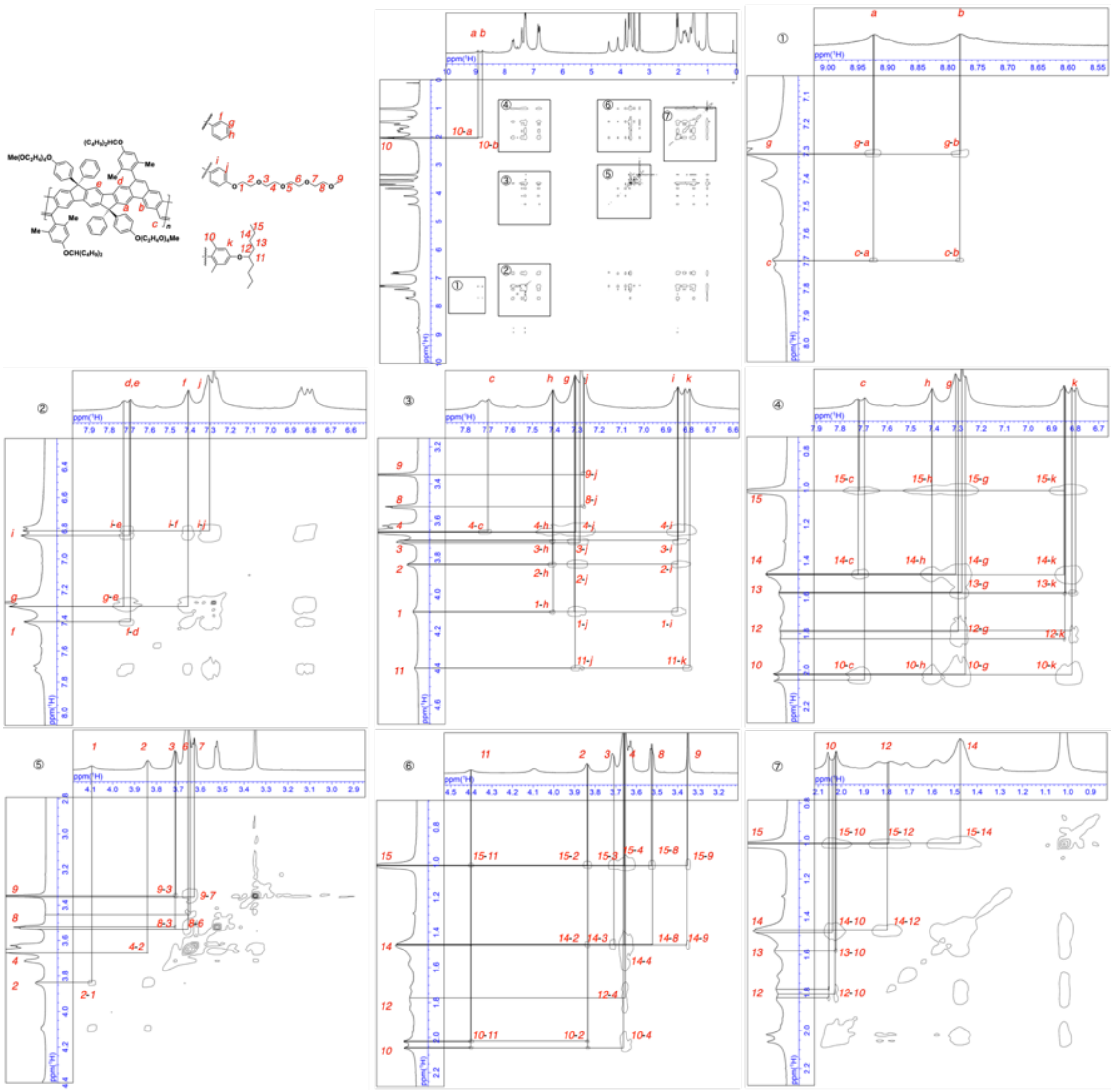

Supplementary Fig. 122 | NOESY correlation of LP-(*S,S*)-**1** (600 MHz, $CDCl_3$, 25 ºC).

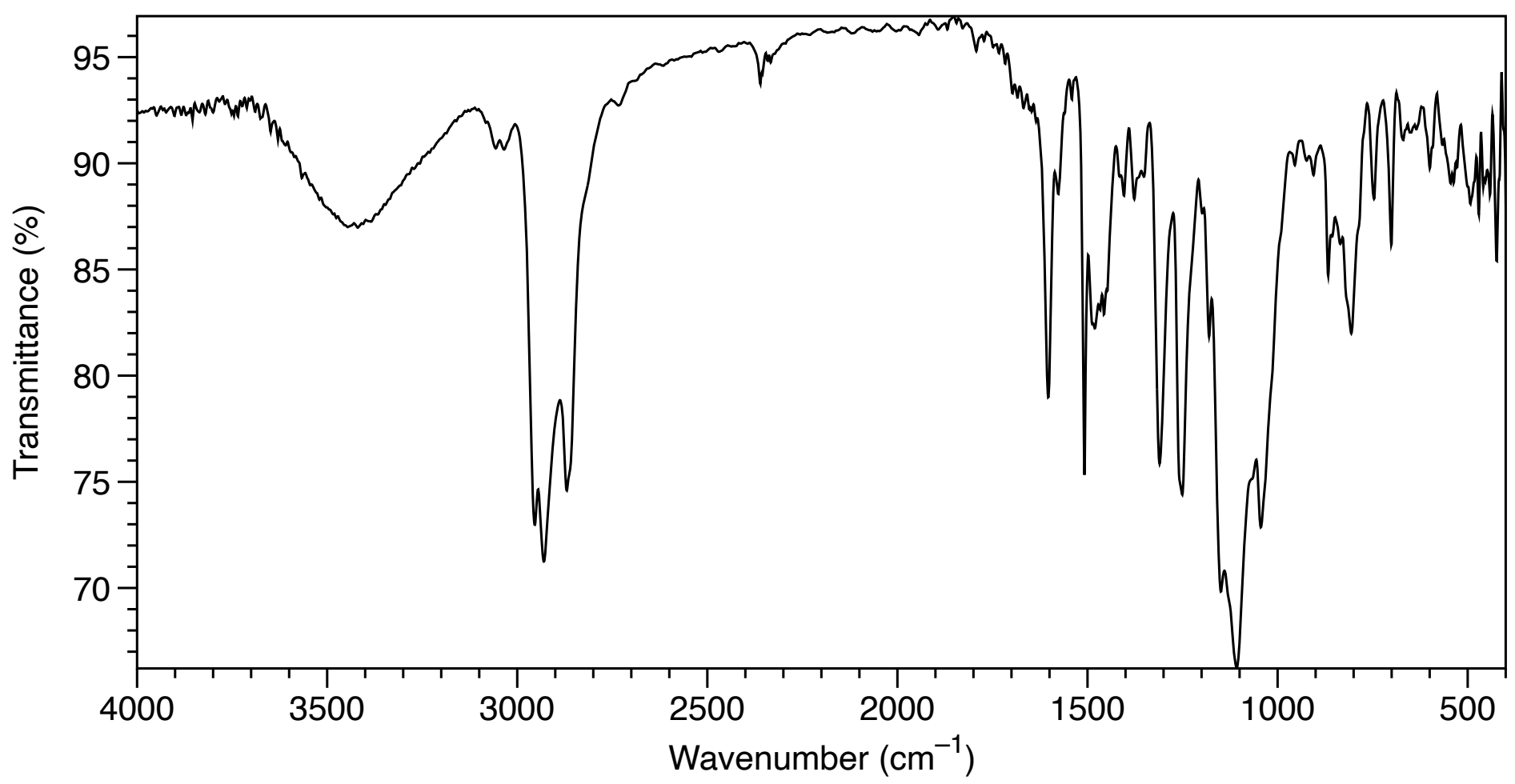

Supplementary Fig. 123 | FT-IR spectrum of LP-(*S,S*)-**1** (KBr, 25 °C).

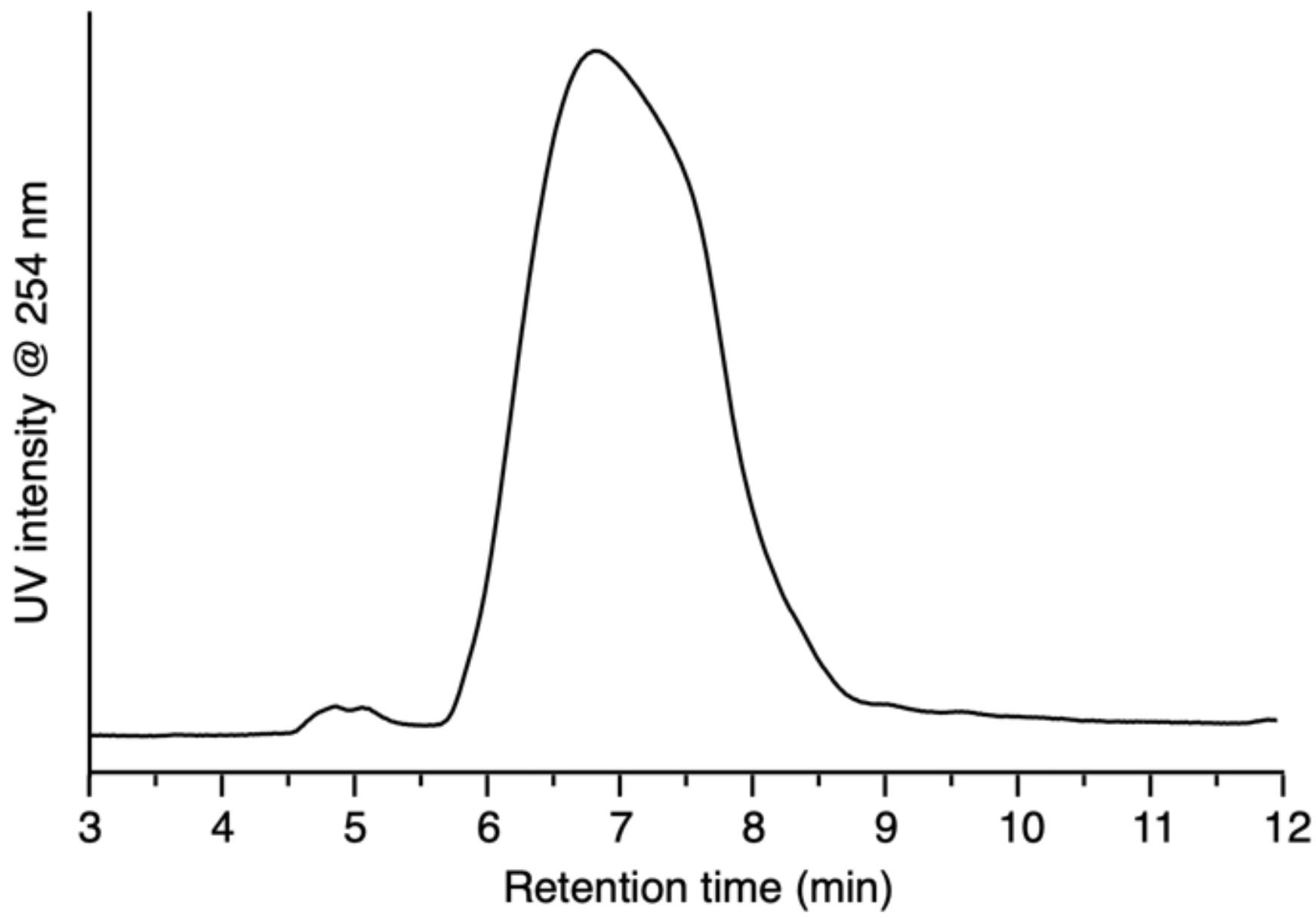

Supplementary Fig. 124 | SEC profiles of LP-(*S*,*S*)-**1** ($CHCl_3$).

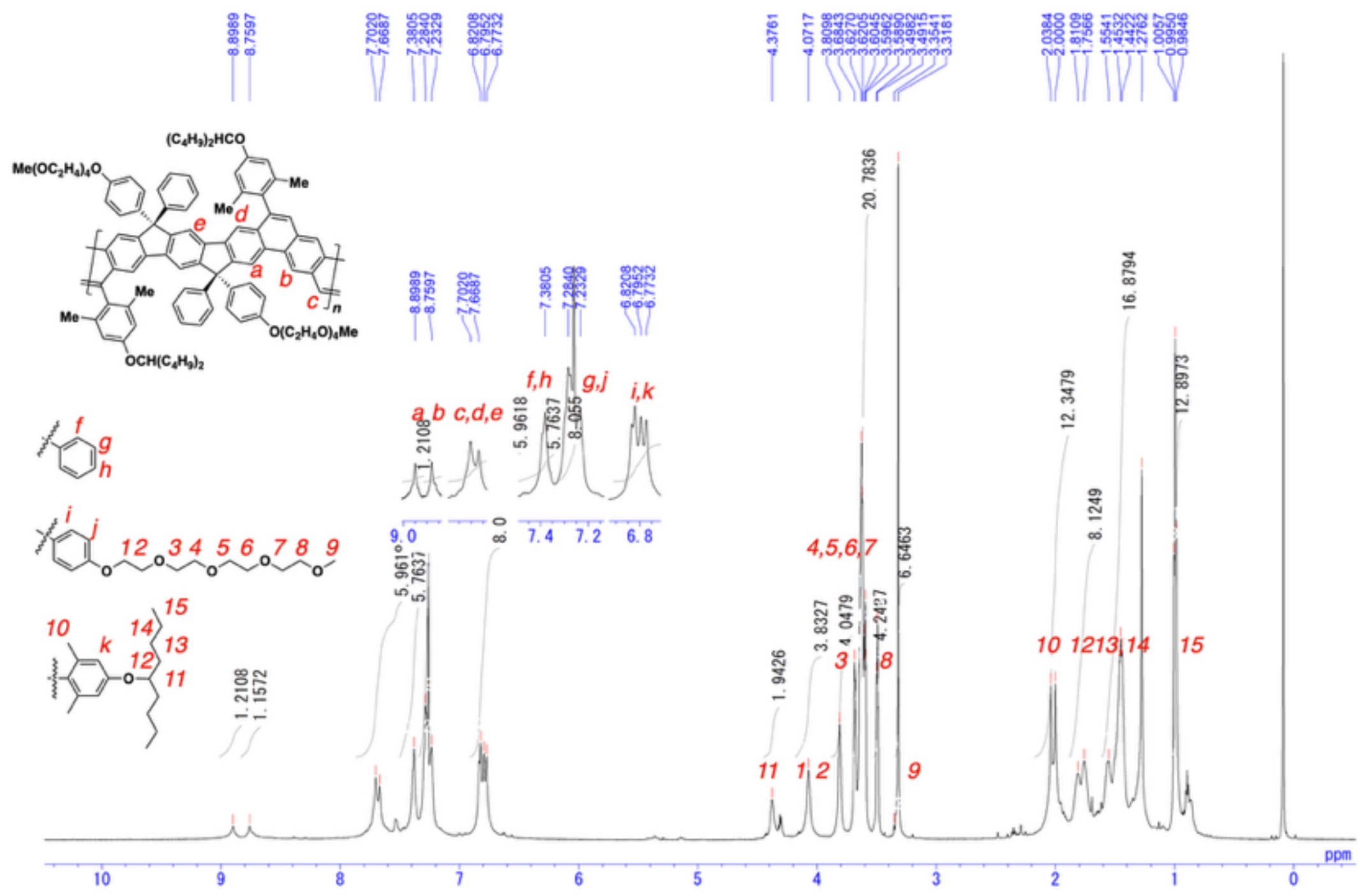

Supplementary Fig. 125 | ^{1}H NMR spectrum of LP-(*R,R*)-**1** (600 MHz, $CDCl_3$, 25 ºC).

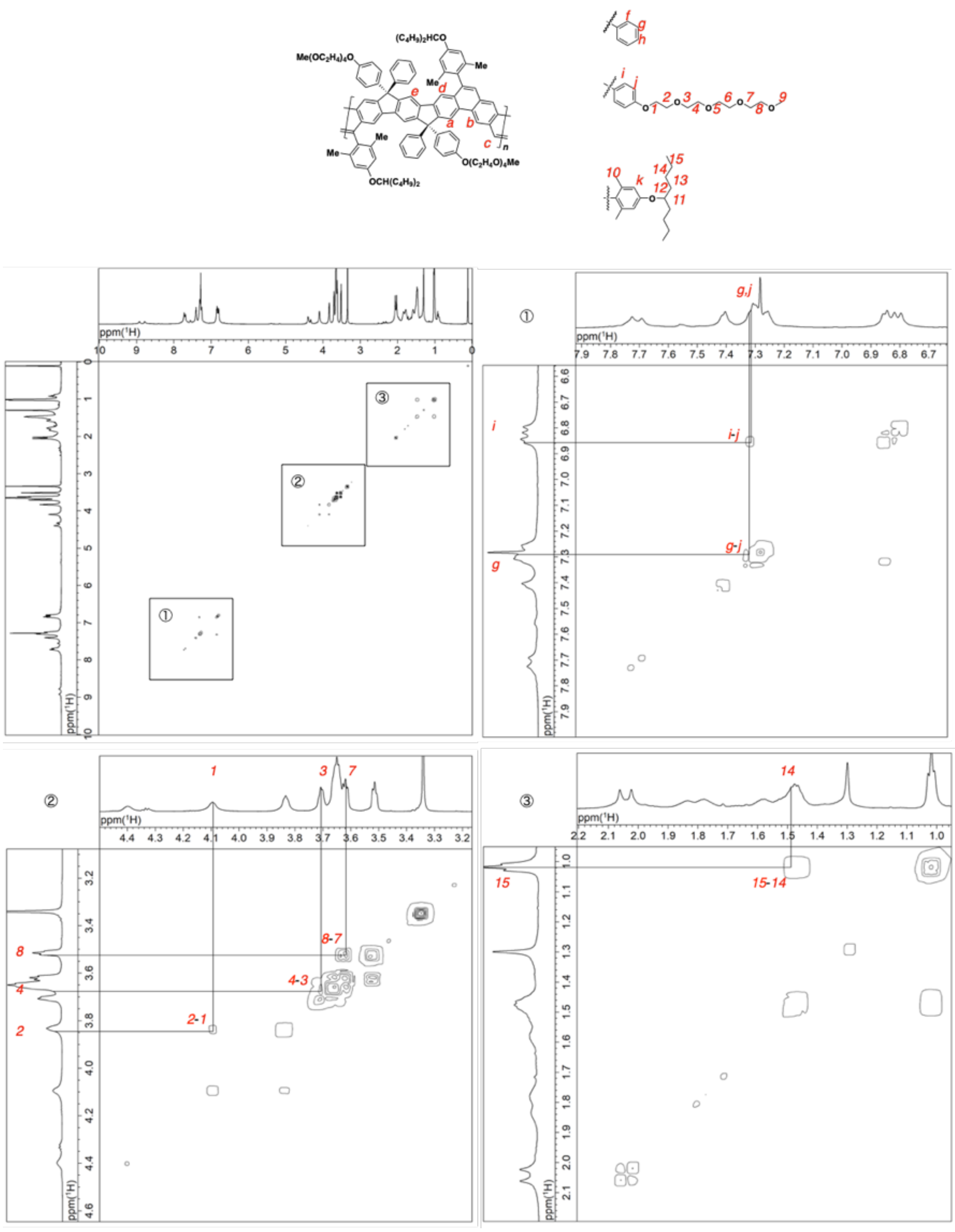

Supplementary Fig. 126 | HH-COSY correlation of LP-(*R,R*)-**1** (600 MHz, $CDCl_3$, 25 ºC).

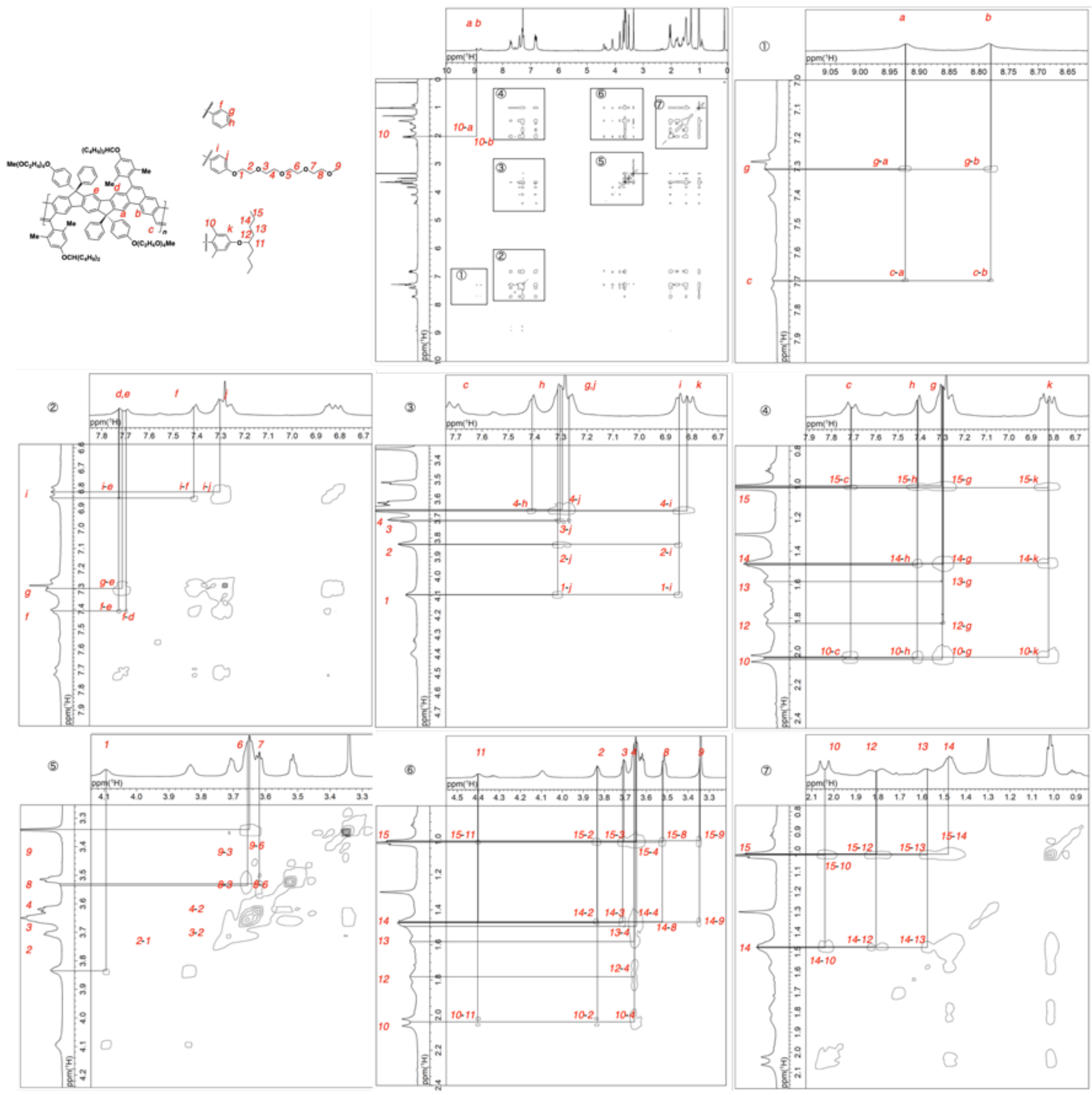

Supplementary Fig. 127 | NOESY correlation of LP-(*R*,*R*)-**1** (600 MHz, $CDCl_3$, 25 ºC).

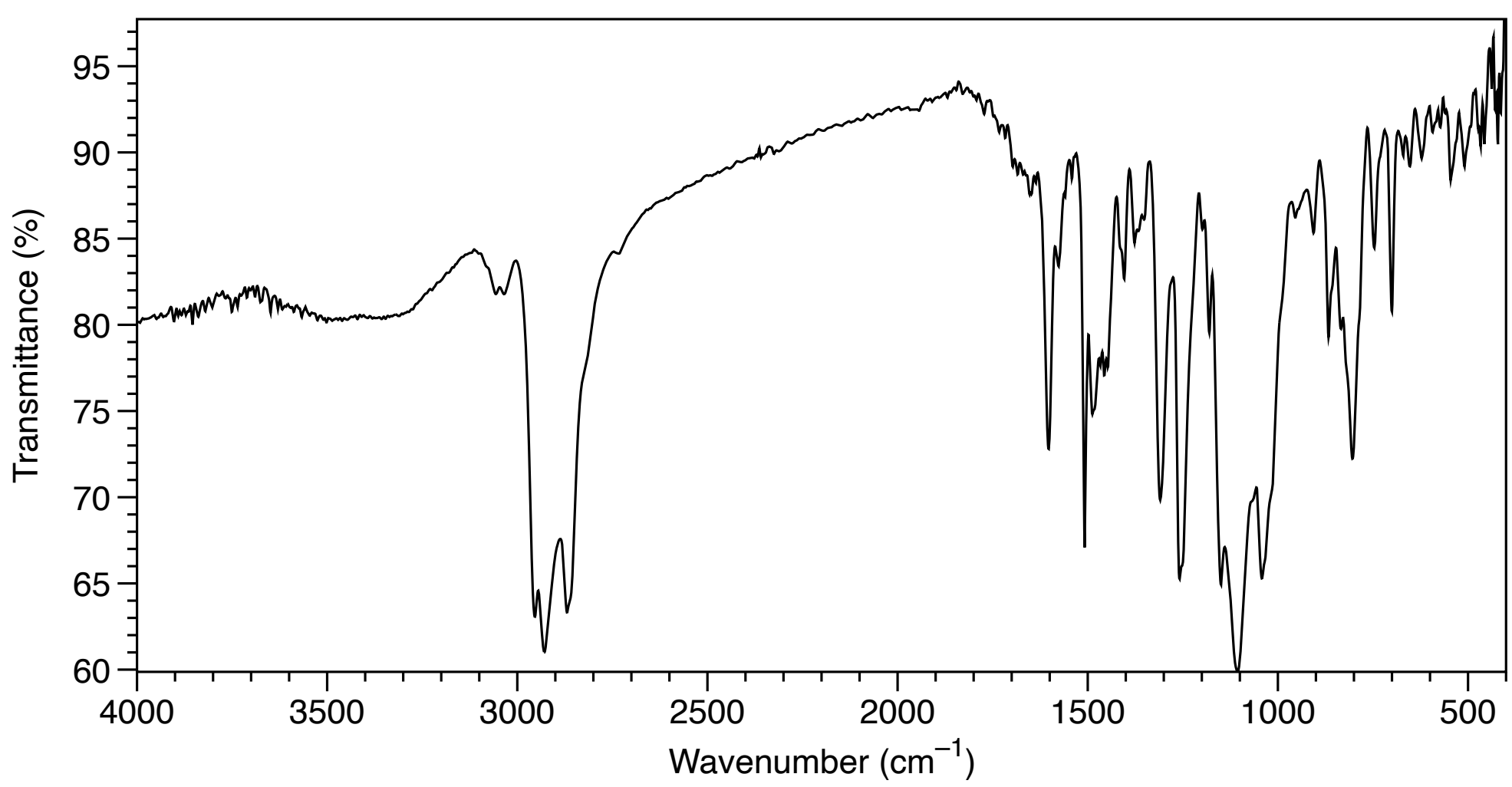

Supplementary Fig. 128 | FT-IR spectrum of LP-(*R*,*R*)-**1** (KBr, 25 °C).

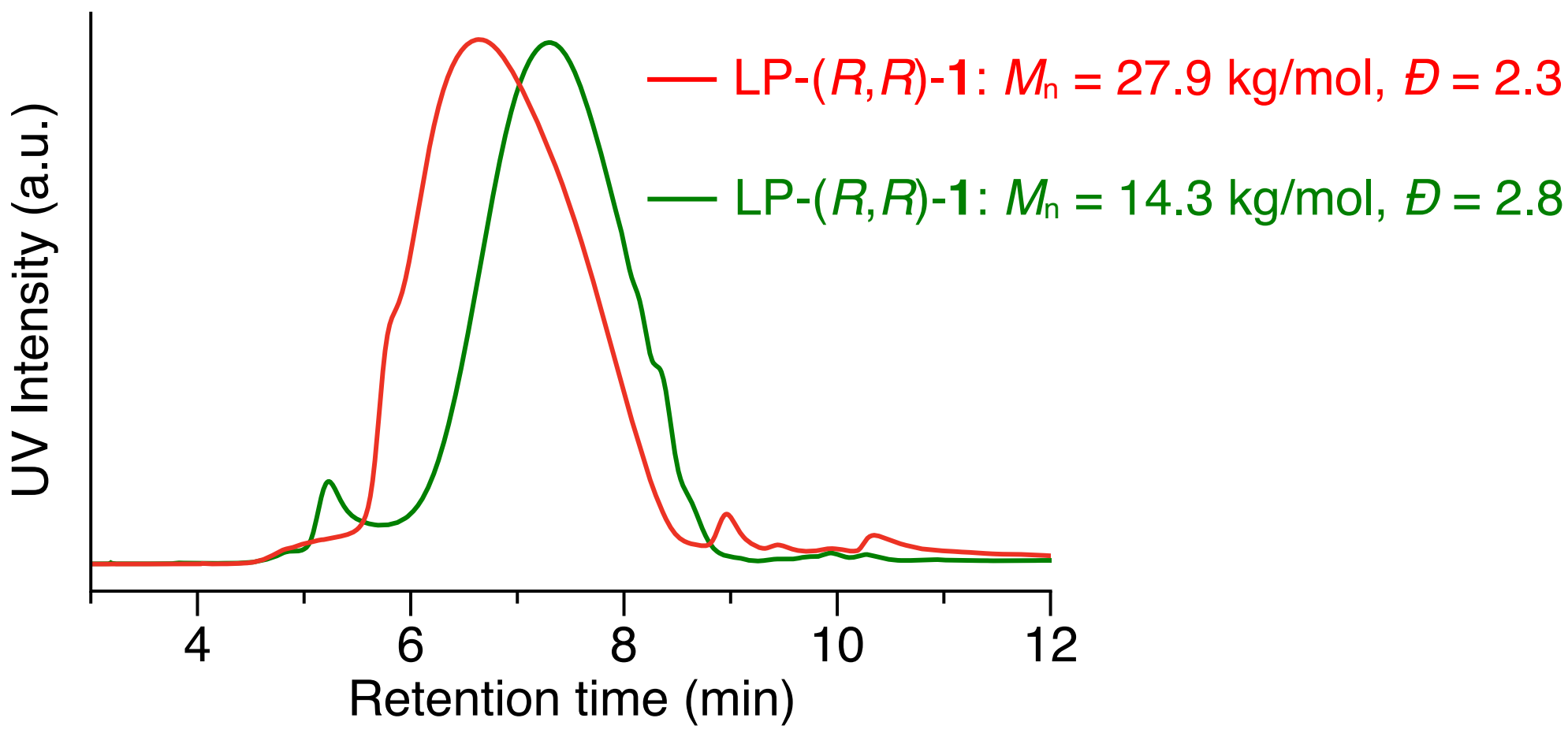

Supplementary Fig. 129 | SEC profiles ($CHCl_3$) of LP-(*R*,*R*)-**1**s with M_n = 27.9 kg/mol, $Đ$ = 2.3 (red) and M_n = 14.3 kg/mol, $Đ$ = 2.8 (green).

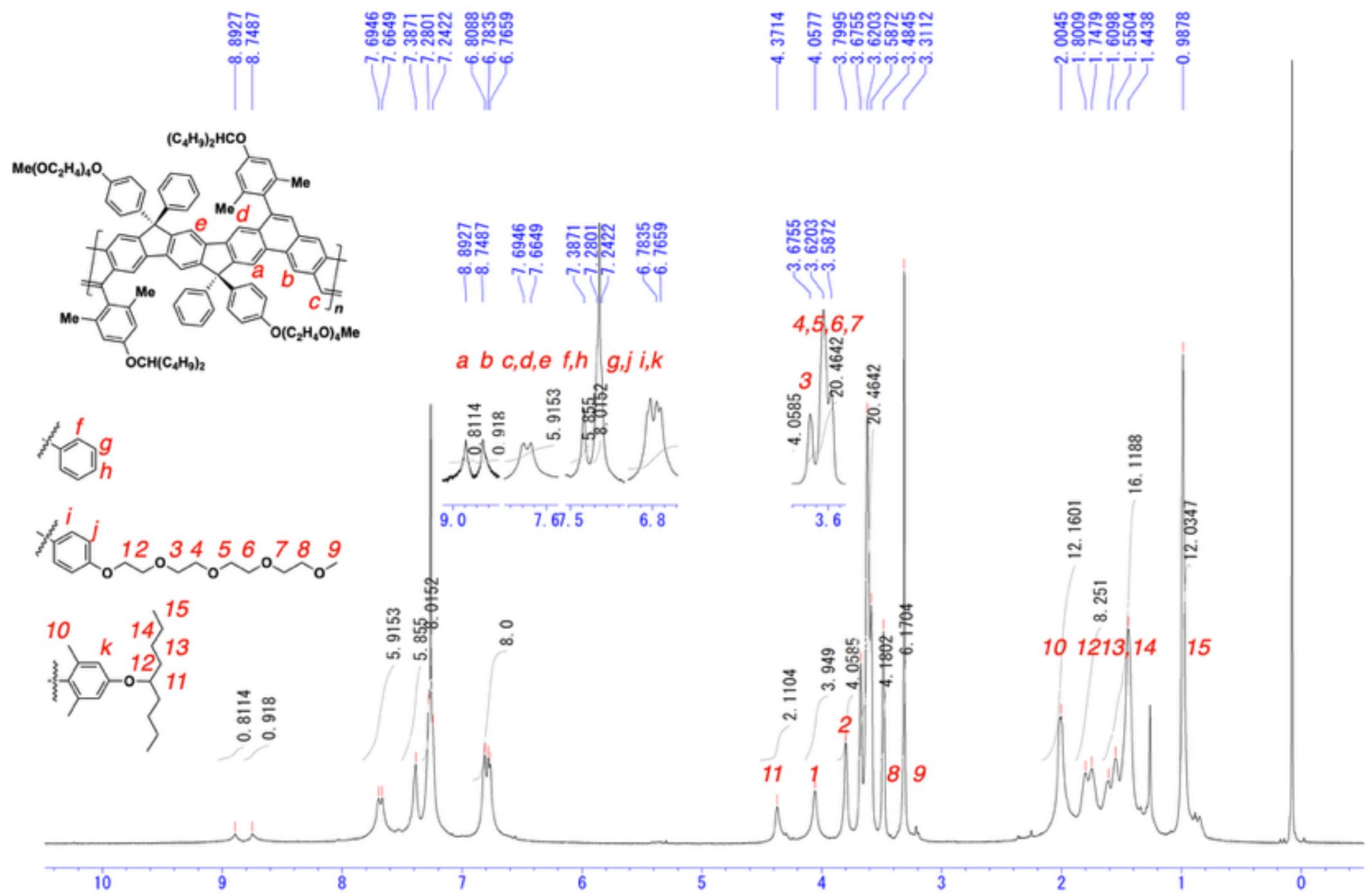

Supplementary Fig. 130 | ^{1}H NMR spectrum of LP-*meso*-**1** (600 MHz, $CDCl_3$, 25 °C).

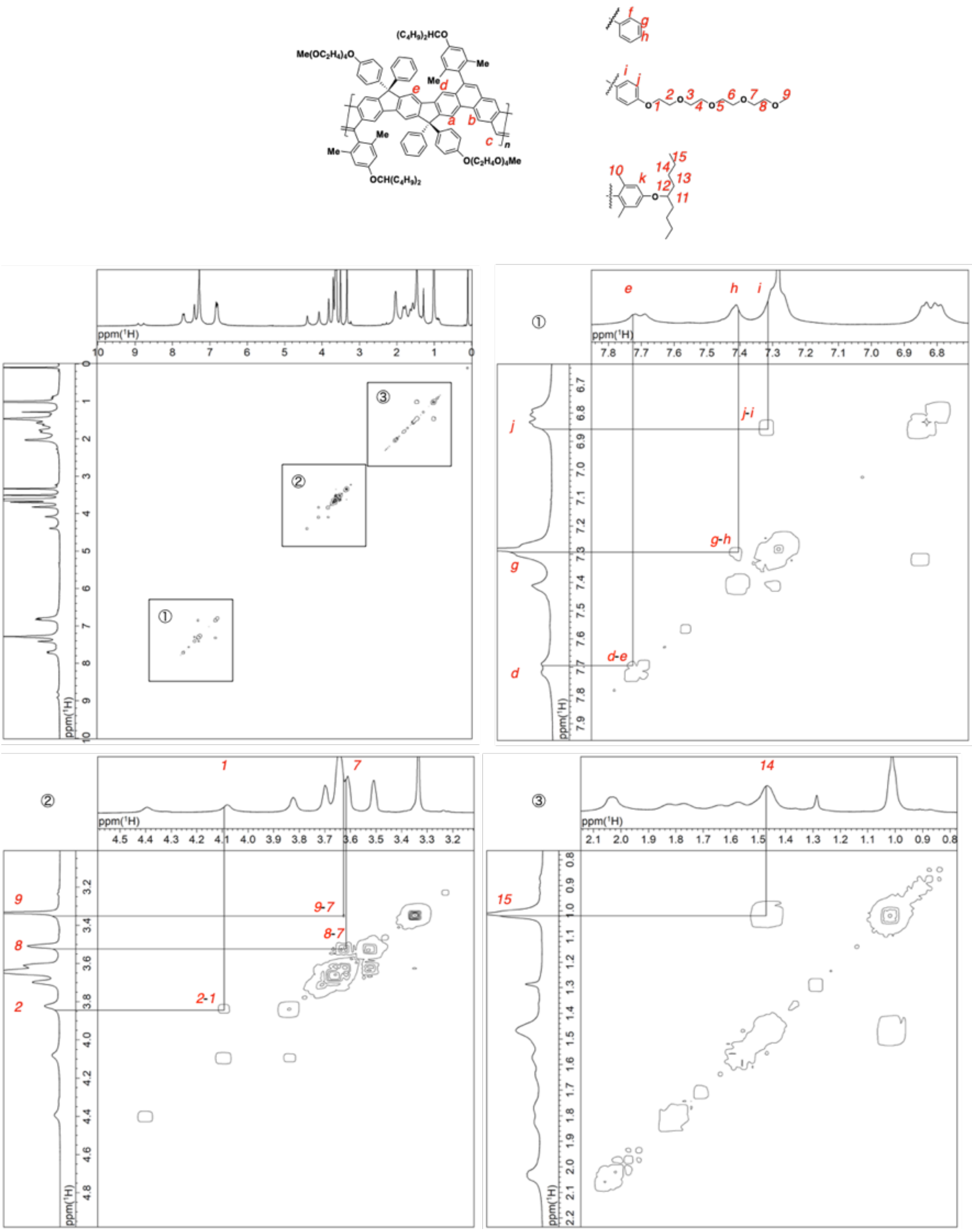

Supplementary Fig. 131 | HH-COSY correlation of LP-*meso*-**1** (600 MHz, $CDCl_3$, 25 ºC).

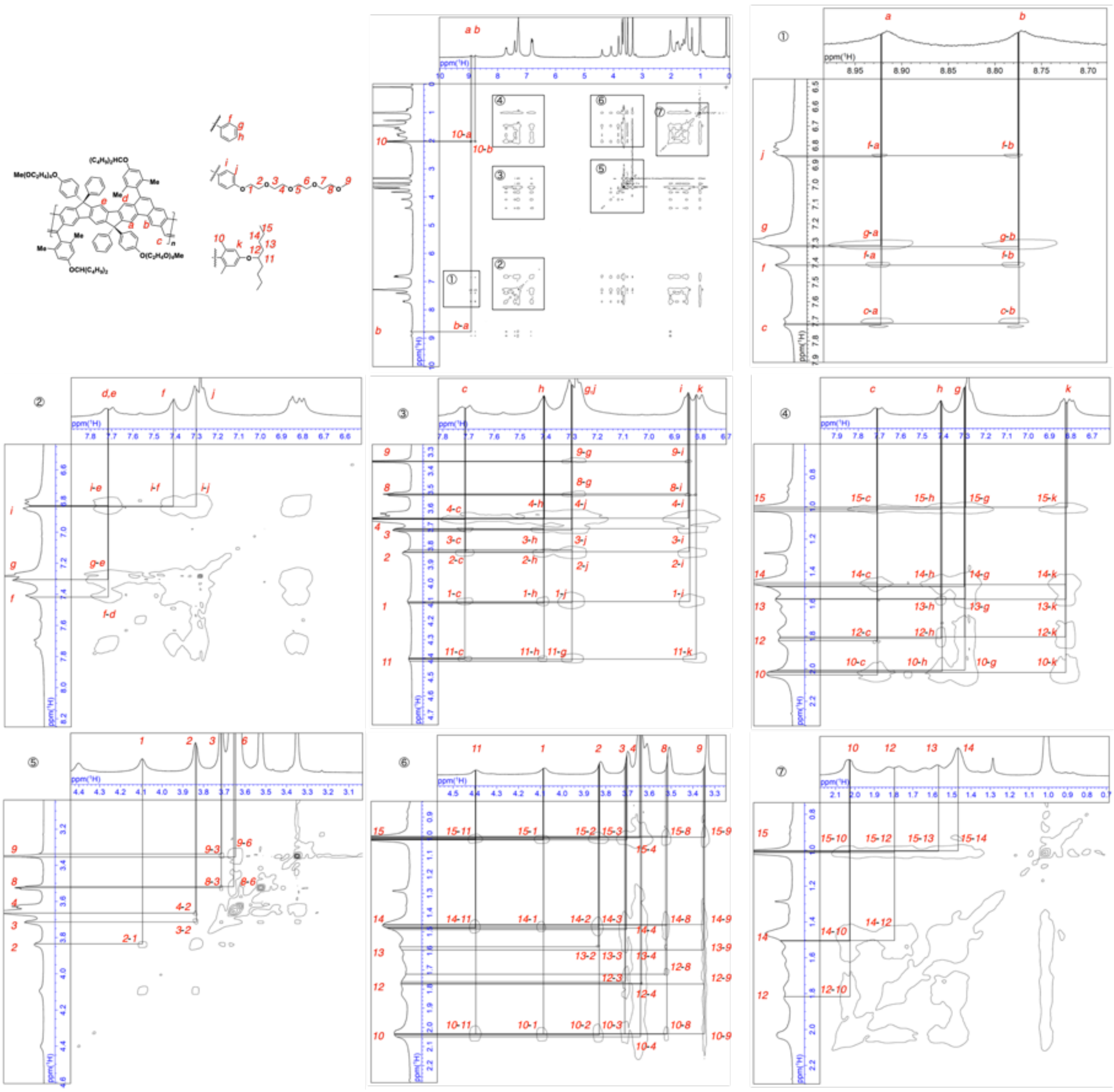

Supplementary Fig. 132 | NOESY correlation of LP-*meso*-**1** (600 MHz, $CDCl_3$, 25 ºC).

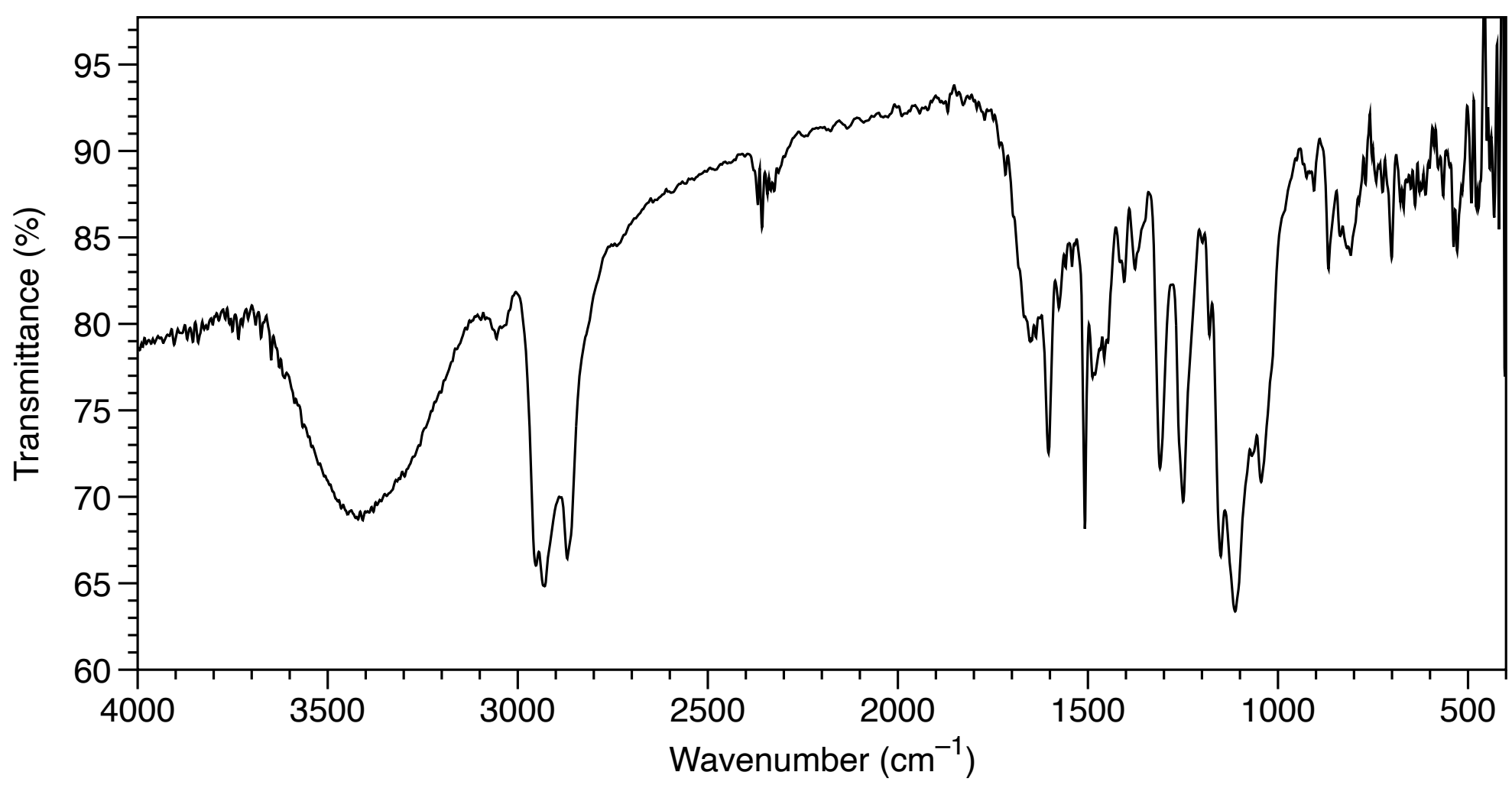

Supplementary Fig. 133 | FT-IR spectrum of LP-*meso*-**1** (KBr, 25 °C).

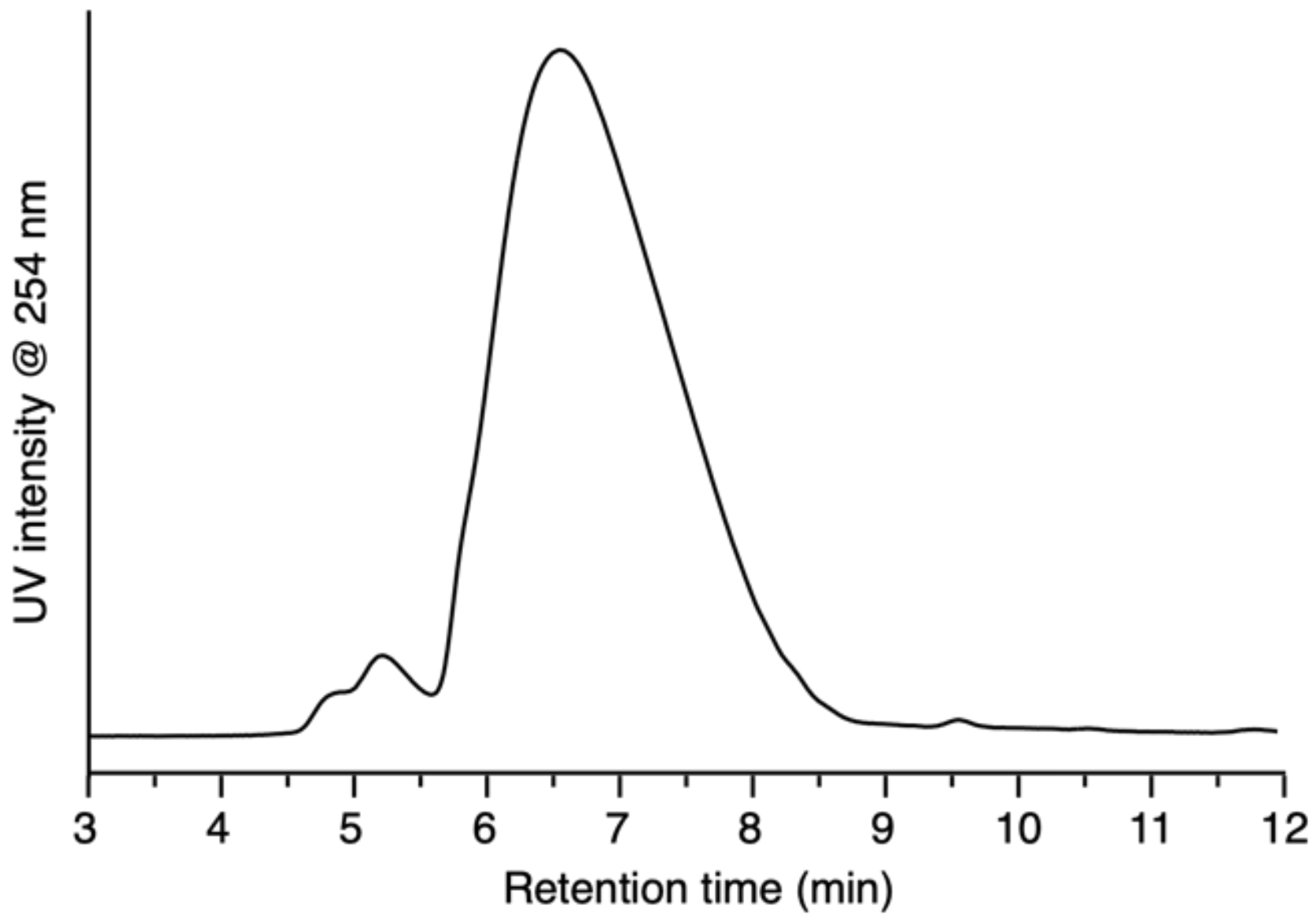

Supplementary Fig. 134 | SEC profile of LP-*meso*-**1** ($CHCl_3$).

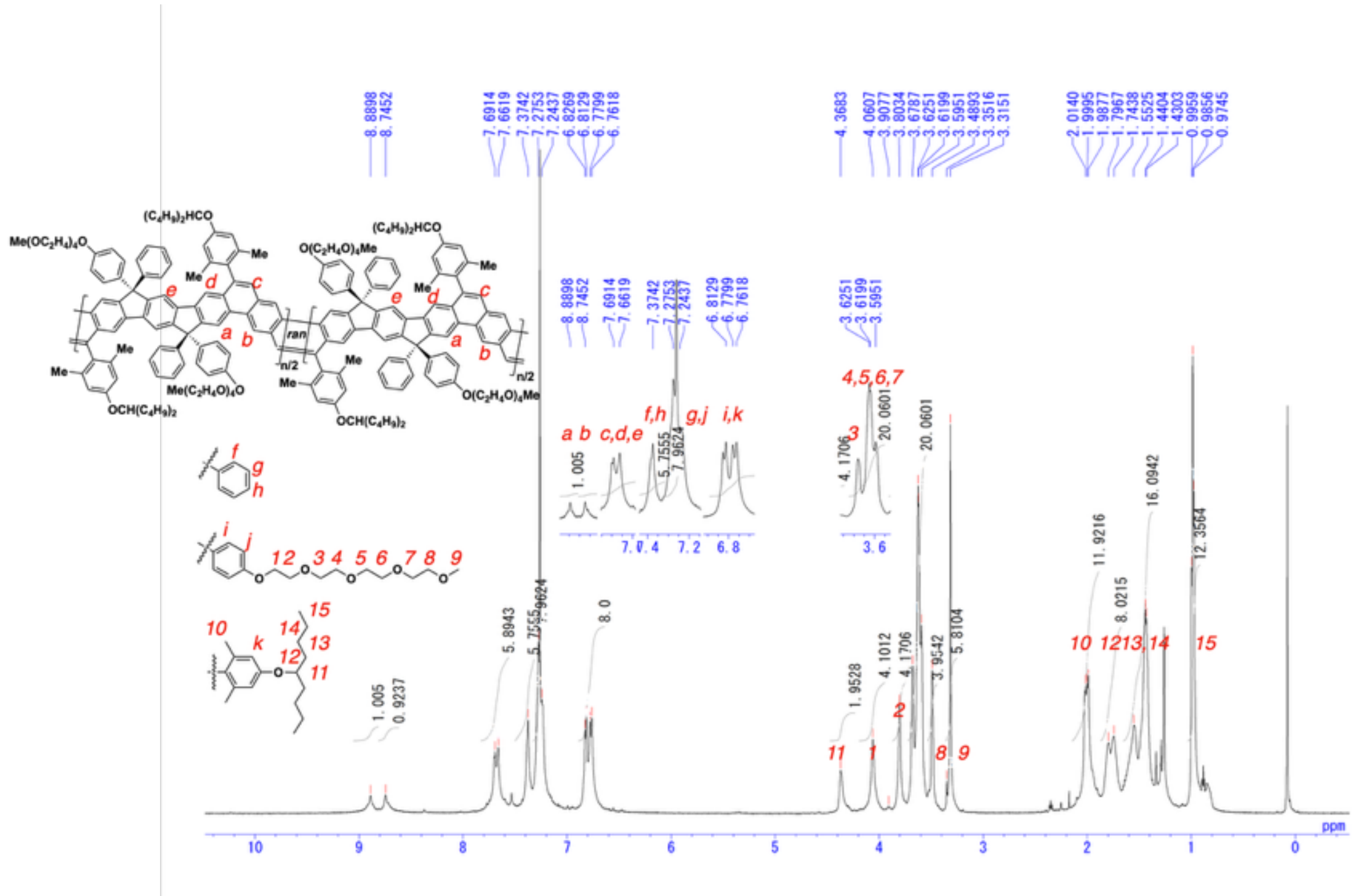

Supplementary Fig. 135 | ^{1}H NMR spectrum of LP-*rac*-**1** (600 MHz, $CDCl_3$, 25 °C).

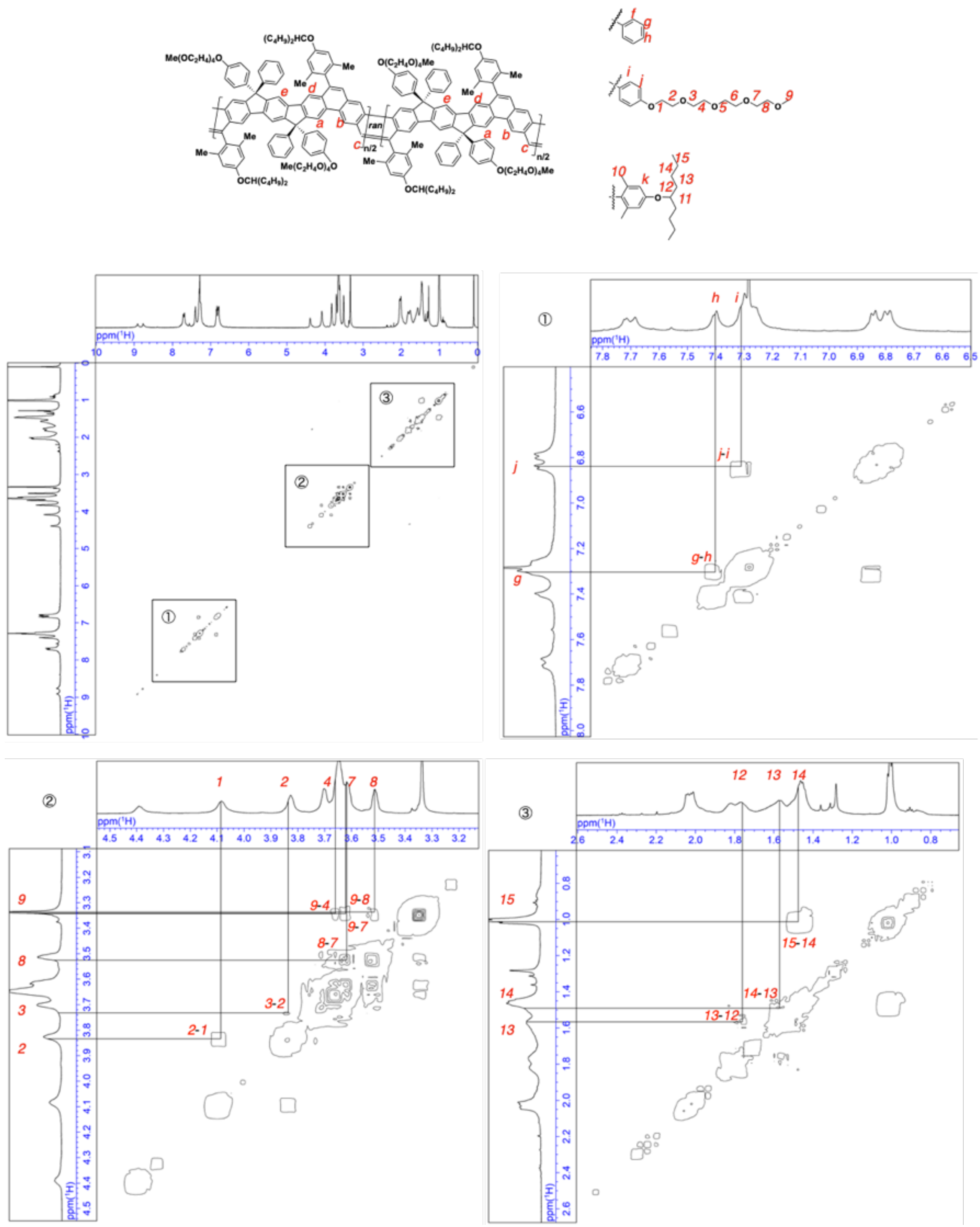

Supplementary Fig. 136 | HH-COSY correlation of LP-*rac*-**1** (600 MHz, $CDCl_3$, 25 ºC).

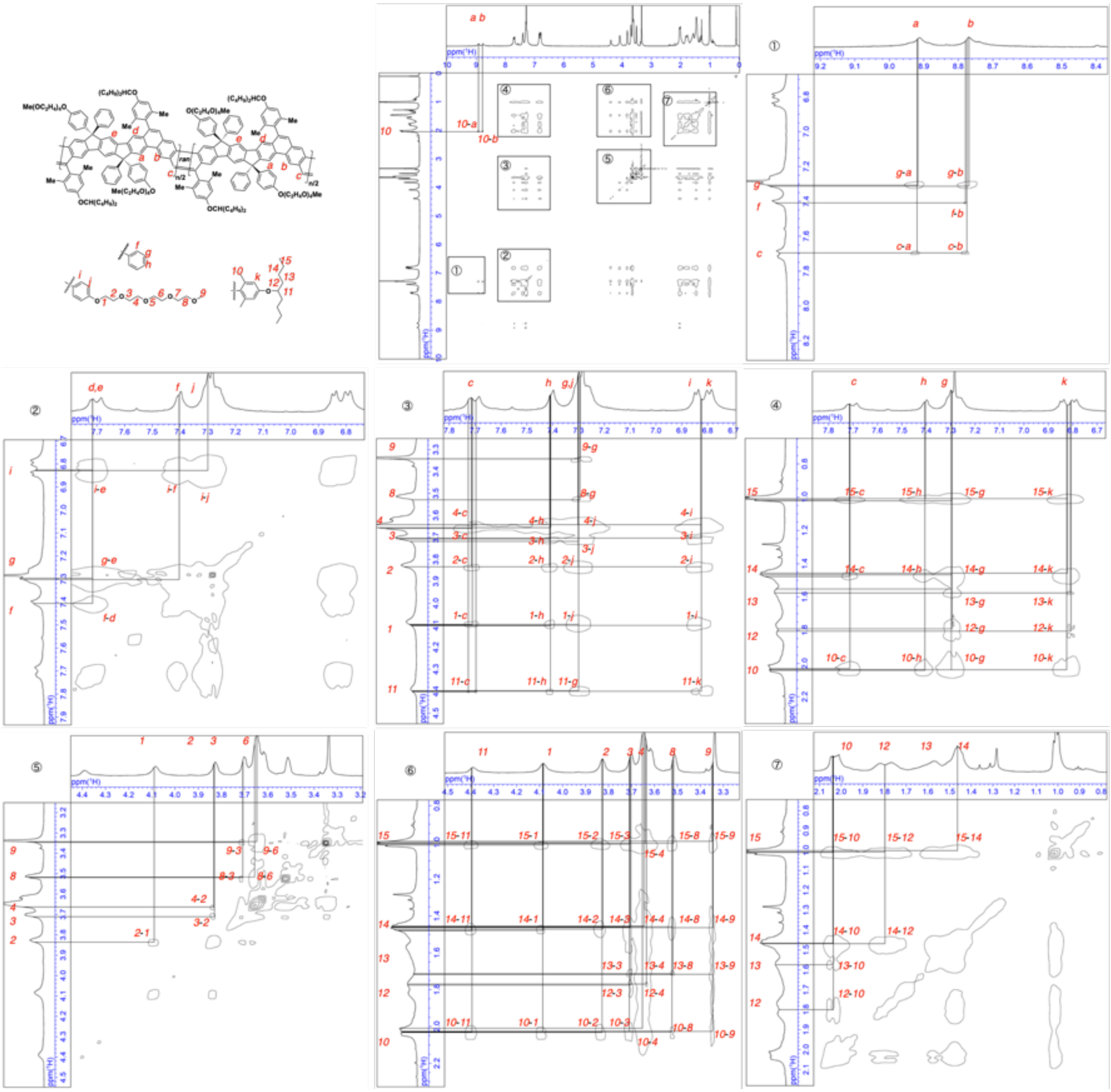

Supplementary Fig. 137 | NOESY correlation of LP-*rac*-**1** (600 MHz, $CDCl_3$, 25 ºC).

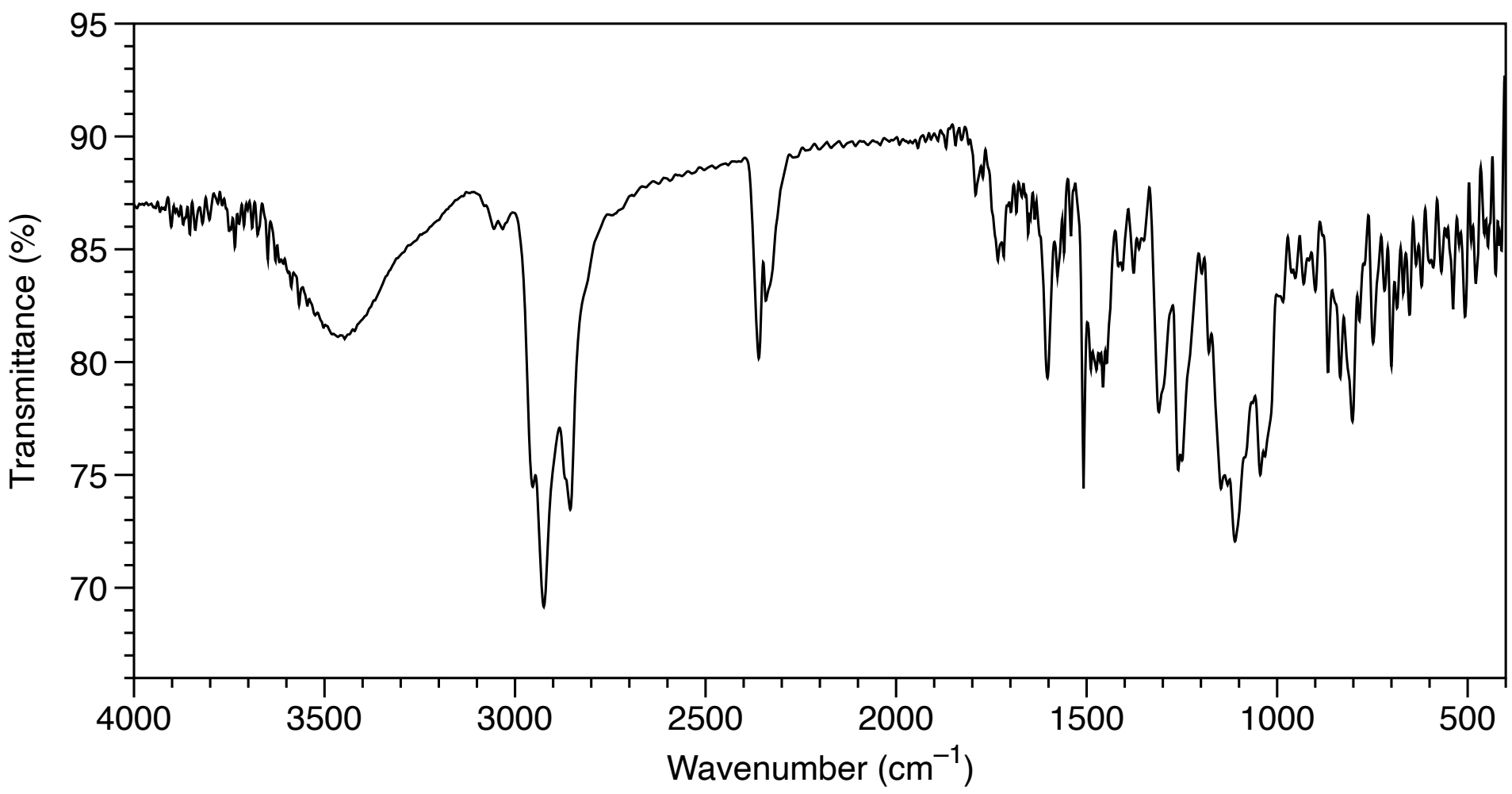

Supplementary Fig. 138 | FT-IR spectrum of LP-*rac*-**1** (KBr, 25 °C).

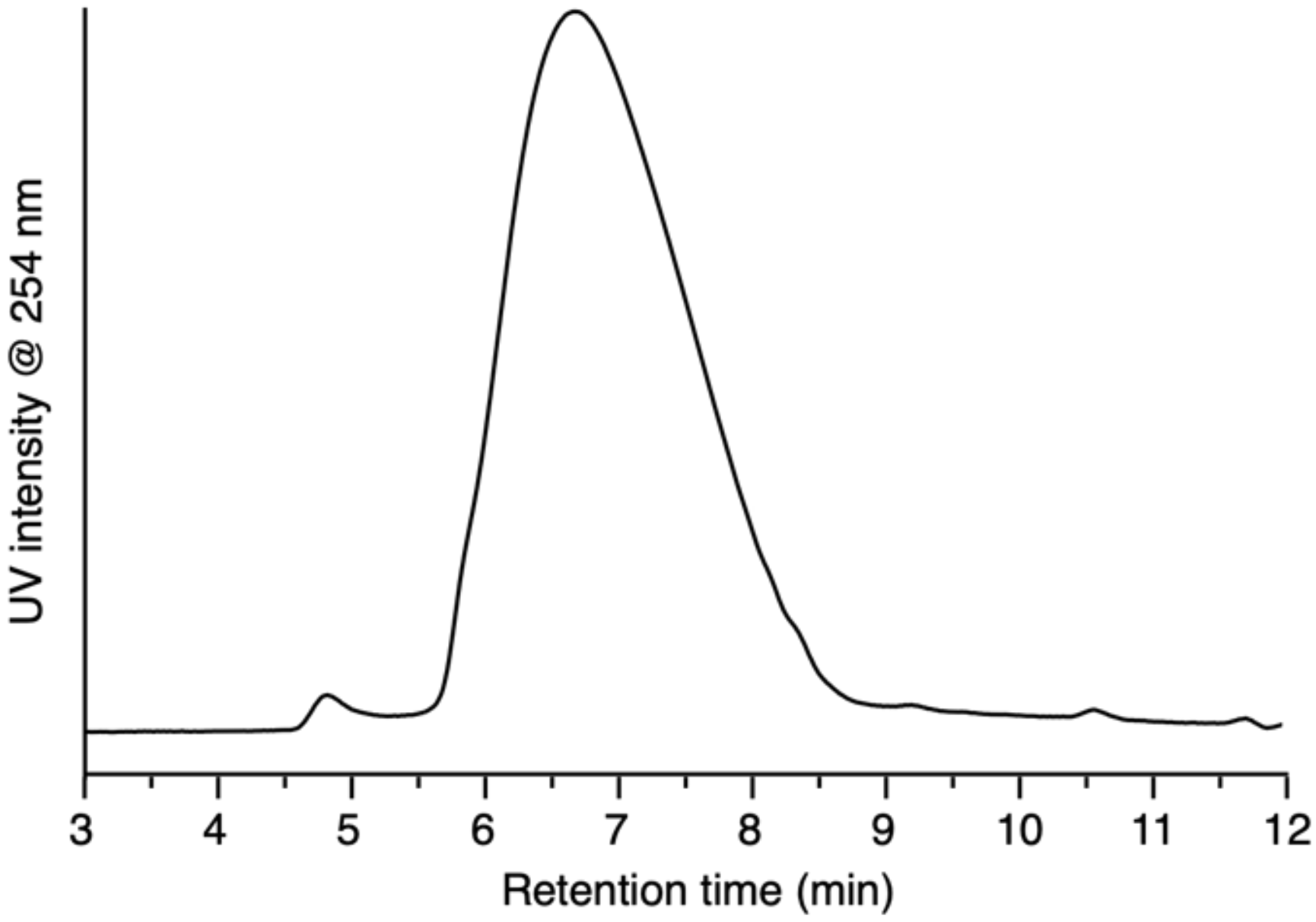

Supplementary Fig. 139 | SEC profile of LP-*rac*-**1** ($CHCl_3$).

7. Supplementary References.

1) Minoi, N., Ishiwari, F., Murotani, K., Nishikubo, R., Fukushima, T., Saeki, A. Surface Passivation of Lead Halide Perovskite Solar Cells by a Bifacial Donor–π–Donor Molecule. *ACS Appl. Mater. Interfaces* **15**, 6708–6715 (2023).
2) Zheng, W., Ikai, T. & Yashima, E. Synthesis of Single-Handed Helical Spiro-Conjugated Ladder Polymers through Quantitative and Chemoselective Cyclizations. *Angew. Chem. Int. Ed*. **60**, 11294–11299 (2021).
3) Frisch, M. J. et al. Gaussian 16, Revision C.01. Gaussian, Inc., Wallingford CT (2016).
4) Kubo, H., Hirose, T., Nakashima, T., Kawai, T., Hasegawa, J.-y., Matsuda & K. Tuning Transition Electric and Magnetic Dipole Moments: [7]Helicenes Showing Intense Circularly Polarized Luminescence. *J. Phys. Chem. Lett*. **12**, 686–695 (2021).
