# Supplementary material for: Bifacial ladder polymers enabled by chirality-assisted synthesis that exhibit self-assembly and chirality-induced spin selectivity
Source: Nat Commun. 2026 Aug 3;17:7602. doi: 10.1038/s41467-026-76059-5 (PMC13433944; doi:10.1038/s41467-026-76059-5)
Supplement: Supplementary file 2 [file 41467_2026_76059_MOESM2_ESM.pdf]

nature portfolio

Peer Review File

Bifacial ladder polymers enabled by chirality-assisted synthesis that exhibit self-assembly and chirality-induced spin selectivity

Corresponding Author: Professor Fumitaka Ishiwari

This file contains all reviewer reports in order by version, followed by all author rebuttals in order by version.

Version 0:

Reviewer comments:

Reviewer #1

(Remarks to the Author)
The manuscript reports a well-designed route to homochiral π-conjugated ladder polymers in which facial orientation is retained along the chain, and it presents amplified solid-state chiroptical responses together with strong CISS signals in thin films. While the synthetic sequence largely follows established polymerization and ladderization approaches, the reported >100-fold CD enhancement and >±90% spin polarization are striking; to justify a Nature Communications-level advance, the manuscript would need either substantially deeper mechanistic insight into the origin of these effects and their dependence on structure/morphology, and/or a clearly articulated, generalizable design rule demonstrated beyond a single system.

Major comments
1. The authors present “bifacial ladder polymers” as a new architectural motif enabled by restricting ladderization to a single geometry through chirality. At present, the conceptual advance is not clearly distinguished from prior work on (i) bifacial/chiral design concepts in related linear π-conjugated polymers, (ii) nor from established ladderization strategies used to access conjugated ladder polymers. The manuscript would benefit from a clearer statement of what is fundamentally new as a design principle and why it should be viewed as a general platform rather than a ladderized extension of an existing concept.

2. The meaning of “bifacial” needs to be defined more explicitly. Is “bifacial” meant to indicate chemically distinct faces (e.g., polar oligoethylene glycol vs hydrophobic phenyls), or does any persistent facial asymmetry—including primarily steric differences such as branched vs linear alkyl substituents (hexyl vs ethylhexyl)—qualify? This distinction directly affects how the mechanistic claims and generality should be interpreted.

3. The manuscript emphasizes preservation of facial differentiation, but it does not clearly articulate what functional advantage is expected to arise specifically from bifaciality beyond structural description. The authors should clarify what bifaciality is intended to enable at the polymer/film level (directional interchain interactions, programmed amphiphilicity, etc.) and why those features should matter for the reported chiroptical and spin-selective responses.

4. The “>100-fold” CD enhancement and “>±90%” spin polarization are striking, but attribution is not sufficiently resolved. It remains unclear whether the improvements are driven mainly by ladderization/rigidification and morphology changes, by ladderization of a homochiral system, or by the specific contribution of preserved bifacial facial differentiation. The claims would be strengthened by controls/comparisons that separate facial differentiation from (i) ladderization-driven ordering, and (ii) by demonstrating generality with a series of related substituent pairs that maintain chirality (hexyl vs ethylhexyl) while varying the type/degree of facial differentiation.

5. Given the defect sensitivity of conjugated ladder polymers, the manuscript should provide more explicit, quantitative evidence for ladderization completeness, structural uniformity, and defect density.

Reviewer #2

(Remarks to the Author)

This manuscript reports the synthesis and spin selectivity of homochiral bifacial ladder polymers. The concept of exploiting the intrinsic rigidity of ladder polymers to construct bifacially chiral macromolecules is highly novel and has the potential to make a significant impact on fundamental macromolecular chemistry. The structural design is particularly intriguing, and the synthesis, characterization, and discussion are carried out in a meticulous and highly professional manner. The manuscript is clearly written and scientifically sound. In its current form, the work is suitable for publication.
That said, the reviewer would like to offer the following suggestions to further enhance the scientific merit and broader impact of the manuscript.
1. This is a purely conceptual suggestion. Could bifacial chirality be regarded as an analogue of tacticity in conventional polymers (e.g., isotactic versus syndiotactic polypropylene)? If so, it may be valuable to discuss whether these concepts could be unified under a broader framework of “polymer tacticity,” which may help situate bifacial chirality within established polymer science terminology.
2. Ladder polymers are well known for their exceptional thermal and chemical stability, as exemplified by classical systems such as BBL and more recent ladder-type polyanilines. Such robustness may translate into enhanced stability of chirality and chirality-related properties in the present system. It is therefore strongly suggested that the authors perform experiments, either in this work or in future studies, to demonstrate this stability. For example, does the chiral bifacial polymer retain its spin selectivity and circular dichroism signals after exposure to elevated temperatures? Is the chirality preserved up to the polymer’s decomposition temperature? How does the material respond to harsh acidic or basic conditions, or to strong light irradiation? Demonstrating such robustness would substantially strengthen the case for ladder polymers as functional chiral materials with practical relevance.

Reviewer #3

(Remarks to the Author)
The paper by Ishiwari deals with the synthesis of bifacial ladder polymers , that is rigid double-stranded backbones bearing two deliberately differentiated molecular faces. The design takes advantage of a chirality-assisted synthetic strategy, an underdeveloped yet very important concept, which the auhtors masterfully develop for polymer synthesis and enantiospecific ladderization. The paper is very well writte and it is, in my view exciting chemistry. The cartoon representation of the chirality processes involved are particularly effective. The efficiency of the ladderization is properly demonstrated with the aid of model compounds. Structure-property-relationship in terms of spin-induced selectivity unravel the added significance of the ladder polymers. Although the authors have presented a similar synthetic approach in other contexts, the paper is very original and respresents a huge step forward in the field of chirality assisted synthesis and ladder polymers. I recommend publication upon addressing the following minor points:
a) the ladderization brings a systematic increase in the polydispersity index. I would have xpected the opposite, with an increas in polymer rigidity. Can the authors comment on this point;
b) the auhtors should specifiy the scale for average batches of enantiomeric separation through chiral HPLC
c) please remove the following from the bastract “ a new acrhetecural motif in polymer science.
d) please cite relevant references: 10.1002/anie.202407495; 10.3390/sym16010116

Version 1:

Reviewer comments:

Reviewer #1

(Remarks to the Author)
The authors have addressed the reviewers’ concerns, and the manuscript is ready for publication.

Reviewer #2

(Remarks to the Author)
I have examined the authors' responses to the previous round of reviewers' comments. They have addressed the comments in a satisfactory manner. I recommend the publication of this work in its current form.

Reviewer #3

(Remarks to the Author)
In my opinion the authors have addressed in a satisfactory manner all comments by the reviewers. Puslish as is. This is a truly fine paper.

Open Access This Peer Review File is licensed under a Creative Commons Attribution 4.0 International License, which permits use, sharing, adaptation, distribution and reproduction in any medium or format, as long as you give appropriate credit to the original author(s) and the source, provide a link to the Creative Commons license, and indicate if changes were made.
In cases where reviewers are anonymous, credit should be given to 'Anonymous Referee' and the source.
The images or other third party material in this Peer Review File are included in the article's Creative Commons license, unless indicated otherwise in a credit line to the material. If material is not included in the article's Creative Commons license and your intended use is not permitted by statutory regulation or exceeds the permitted use, you will need to obtain permission directly from the copyright holder.
To view a copy of this license, visit https://creativecommons.org/licenses/by/4.0/

Point-to-Point Answers to Reviewers' Comments and Revisions:

For all the reviewers:

We would like to express our sincere thanks to all the reviewers for spending his/her valuable time on this manuscript and providing useful comments, which helped to improve the paper. We carefully revised the manuscript by taking the reviewers' comments into account.

For Reviewer #1:

The manuscript reports a well-designed route to homochiral π-conjugated ladder polymers in which facial orientation is retained along the chain, and it presents amplified solid-state chiroptical responses together with strong CISS signals in thin films. While the synthetic sequence largely follows established polymerization and ladderization approaches, the reported >100-fold CD enhancement and >±90% spin polarization are striking; to justify a Nature Communications-level advance, the manuscript would need either substantially deeper mechanistic insight into the origin of these effects and their dependence on structure/morphology, and/or a clearly articulated, generalizable design rule demonstrated beyond a single system.

=> We would like to express our sincerest gratitude to this reviewer for this encouraging comment.

Major comments

1. The authors present "bifacial ladder polymers" as a new architectural motif enabled by restricting ladderization to a single geometry through chirality. At present, the conceptual advance is not clearly distinguished from prior work on (i) bifacial/chiral design concepts in related linear π-conjugated polymers, (ii) nor from established ladderization strategies used to access conjugated ladder polymers. The manuscript would benefit from a clearer statement of what is fundamentally new as a design principle and why it should be viewed as a general platform rather than a ladderized extension of an existing concept.

=> We thank the reviewer for this insightful comment regarding the conceptual novelty and positioning of this work.

First, with respect to bifacial or chiral π-conjugated polymers, previously reported systems [ref. #21 (originally #42), Ishshiwari, F. et al. *Chem. Commun.* **60**, 10870–10873 (2024), ref. #22, Gavvalapalli N. et al. *Chem. Sci.*, **10**, 5976–5982 (2019)] incorporating asymmetric or bifacial monomer units typically retain rotational freedom along the polymer backbone or lack control over the relative orientation of repeating units. As a result, facial differentiation is not preserved uniformly at the macromolecular level. In contrast, the present ladder polymer architecture, based on conformationally locked double-stranded backbones, enforces a uniform facial orientation along the entire polymer chain, thereby ensuring persistent facial asymmetry even in solution.

Second, regarding ladderization strategies, we emphasize that the ladderization reactions employed in this study are well-established and we do not claim novelty in the ladderization strategy itself. Rather, the conceptual advance lies in the integration of (i) C_2-symmetric bifacial monomer design and (ii) a regioselective ladderization process, which together enforce a single mode of covalent connectivity. While many established ladderization strategies are not inherently regioselective, the present system intentionally employs a geometrically selective ladderization reaction, which enables the formation of a single, well-defined ladder structure with uniform facial orientation. Therefore, we consider that the fundamental advance of this work is the establishment of a design principle in which molecular chirality is used to control covalent connectivity and macromolecular geometry, enabling structurally defined bifacial ladder polymers.

Importantly, this structural definition provides a platform for the face-selective arrangement of substituents and functional groups along the polymer backbone, effectively serving as supramolecular scaffolds [Fukushima T. et al. *Chem. Sci.*, **9**, 2028–2041 (2018)] for organizing functional molecular units. In this sense, the present system should be viewed not as a simple ladderized extension of existing concepts, but as a general framework for translating molecular asymmetry into persistent, functionally addressable macromolecular architecture.

Following the reviewer's suggestion, we have added an explanatory sentence to clarify these points in the Introduction of the revised manuscript, along with the relevant references.

[Page 2, lines 61–67] "Unlike π-conjugated polymers incorporating bifacially asymmetric monomer units[21,22], which typically retain rotational freedom along the backbone or lack control over the orientation of repeat units, the present ladder polymer architecture enforces a uniform facial orientation along the polymer chain, thereby ensuring persistent facial asymmetry even in solution and providing a unique platform for the face-selective arrangement of substituents and functional groups[23]."

Ref #22) Chaudhuri, S., Mohanan, M., Willems, A. V., Bertke J. A. & Gavvalapalli N. β-Strand inspired bifacial p-conjugated polymers *Chem. Sci.*, **10**, 5976–5982 (2019)

Ref #23) Ishiwari, F., Shoji, Y. & Fukushima T. Supramolecular scaffolds enabling the controlled assembly of functional molecular units. *Chem. Sci.*, **9**, 2028–2041 (2018).

2. The meaning of "bifacial" needs to be defined more explicitly. Is "bifacial" meant to indicate chemically distinct faces (e.g., polar oligoethylene glycol vs hydrophobic phenyls), or does any persistent facial asymmetry—including primarily steric differences such as branched vs linear alkyl substituents (hexyl vs ethylhexyl)—qualify? This distinction directly affects how the mechanistic claims and generality should be interpreted.

=> We thank the reviewer for raising this point regarding the definition of "bifacial". In the present work, the term "bifacial" refers to a ladder polymer architecture in which two different substituents are persistently oriented on opposite faces of the rigid ladder backbone, thereby

generating permanent facial asymmetry along the polymer chain. Thus, the key requirement is the presence of persistent facial asymmetry arising from differentiation between the substituents on the two faces.

This definition also includes cases where substituents differ in chemical structure, even when they are constitutional isomers (*e.g.*, *n*-octyl vs 2-ethylhexyl), as long as they give rise to persistent facial differentiation. This definition does not require the two faces to differ strongly in polarity (*e.g.*, hydrophilic vs hydrophobic), but rather focuses on the presence of persistent structural differentiation.

In the present system, the two faces are composed of chemically distinct substituents that differ in both structure and polarity. This further emphasizes the bifacial nature of the ladder polymer. We have clarified this definition in the revised manuscript as follows.

[Page 2–3, line 72–75] In this work, the term “bifacial” refers to a ladder polymer architecture in which two different substituents are persistently oriented on opposite faces of the rigid ladder backbone, thereby generating permanent facial asymmetry along the polymer chain.

3. The manuscript emphasizes preservation of facial differentiation, but it does not clearly articulate what functional advantage is expected to arise specifically from bifaciality beyond structural description. The authors should clarify what bifaciality is intended to enable at the polymer/film level (directional interchain interactions, programmed amphiphilicity, etc.) and why those features should matter for the reported chiroptical and spin-selective responses.

=> We thank the reviewer for this important comment regarding the functional role of bifaciality.

Bifacial architectures are expected to enable face-selective intermolecular interactions along the polymer backbone arising from the differentiation of the two faces. These interactions are expected to influence intermolecular organization and promote the formation of well-defined supramolecular structures in the solid state, including the helical assemblies observed in thin films.

In addition, the rigid ladder structure, fixed by covalent bonds, may facilitate the contribution of otherwise weak interactions, further supporting the formation of higher-order supramolecular organization. In this sense, bifaciality, in combination with ladderization and the polymeric nature of the system, provides a structural basis for controlled organization of the π-conjugated backbones.

These structural features are directly relevant to the observed chiroptical and spin-selective responses. In particular, the formation of well-defined chiral supramolecular organization is considered to enhance intermolecular electronic coupling, consistent with the exciton-coupling mechanism discussed in the manuscript, thereby contributing to the amplification of circular dichroism.

For the spin-selective response, it has been experimentally observed that chiral supramolecular organization can enhance spin polarization, although the detailed mechanism remains an open question. The present results are consistent with this empirical relationship between chiral organization and spin-selective transport.

Taken together, these considerations clarify the functional role of bifaciality in enabling supramolecular organization and its associated chiroptical and spin-selective properties.

Following the reviewer's suggestion, we have revised the manuscript to more clearly articulate this point in the Introduction as follows.

[Page 2, lines 67–71] "Such bifacial ladder polymers are expected to enable face-selective intermolecular interactions arising from the differentiation of the two faces along the polymer backbone. In addition, the rigid ladder structure, fixed by covalent bonds, may facilitate the contribution of otherwise weak interactions, thereby promoting the formation of higher-order supramolecular organization and the emergence of associated functional properties."

4. The ">100-fold" CD enhancement and ">±90%" spin polarization are striking, but attribution is not sufficiently resolved. It remains unclear whether the improvements are driven mainly by ladderization/rigidification and morphology changes, by ladderization of a homochiral system, or by the specific contribution of preserved bifacial facial differentiation. The claims would be strengthened by controls/comparisons that separate facial differentiation from (i) ladderization-driven ordering, and (ii) by demonstrating generality with a series of related substituent pairs that maintain chirality (hexyl vs ethylhexyl) while varying the type/degree of facial differentiation.

=> We thank the reviewer for this insightful and important comment. We agree that the present data do not allow us to unambiguously separate the contribution of preserved facial differentiation from those of ladderization-induced rigidification/ordering and morphology. Therefore, we interpret preserved facial differentiation as one contributing structural factor, rather than the sole origin of the observed responses.

Regarding the CD enhancement, we interpret this behavior as arising from chiral supramolecular organization, which can give rise to exciton-coupling effects. As discussed in response to Comment 3, such chiral supramolecular organization is considered to originate from the structural characteristics of the bifacial ladder polymers, while the rigid ladder backbone and polymeric nature of the system may facilitate the amplification of otherwise weak chiral interactions.

Following the reviewer's suggestion, we have added an explanatory sentence to clarify this relationship as follows.

[Page 11–12, lines 406–408] "This behavior may arise from the rigid ladder backbone and polymeric nature of the system, which together can facilitate the amplification of otherwise weak chiral interactions, as described in the Introduction."

=> Regarding the high spin polarization values, it has been experimentally established that chiral supramolecular organization can enhance spin-selective transport, although the detailed microscopic mechanism remains under active investigation. In particular, the large spin polarization observed in many CISS systems has been suggested to involve collective or dynamic chiral effects in condensed phases, beyond what would be expected from conventional spin–orbit coupling alone. This point has also been highlighted in a recent comprehensive review of CISS phenomena [Kusunose, H., Kishine, J.-i., Yamamoto, H. M., Emergence of chirality from electron spins, physical fields, and material-field composites. *Appl. Phys. Lett.* **124**, 260501 (2024)], where the origin of the large spin polarization remains an open question.

In line with this view, the formation of chiral supramolecular organization in the present system is considered to contribute to the high spin polarization observed in the thin films. At the same time, we acknowledge that multiple structural factors—including preserved facial differentiation, ladderization, backbone rigidity, and polymeric effects—are likely involved, and their individual contributions cannot be unambiguously separated at this stage. We agree that systematic comparisons using related substituent pairs would be valuable to further disentangle these contributions; however, such investigations are beyond the scope of the present study and will be addressed in future work.

Accordingly, we have added an explanatory sentence and a new reference (#26) in the revised manuscript to clarify this point.

[Page 13, lines 462–464] "While it has been experimentally established that chiral organization can enhance spin polarization, the detailed mechanism remains under active investigation[25,26]."

Ref. #26) Kusunose, H., Kishine, J.-i., Yamamoto, H. M., Emergence of chirality from electron spins, physical fields, and material-field composites. *Appl. Phys. Lett.* **124**, 260501 (2024).

5. Given the defect sensitivity of conjugated ladder polymers, the manuscript should provide more explicit, quantitative evidence for ladderization completeness, structural uniformity, and defect density.

=> We thank the reviewer for this helpful comment regarding ladderization completeness, structural uniformity, and possible defect formation in the ladder polymers.

We note that several independent lines of evidence supporting near-complete ladderization and high structural uniformity are already provided in the manuscript. First, the model reaction demonstrates that the Friedel–Crafts-type cyclization proceeds quantitatively and exclusively at the geometrically correct position, producing a single ladderized product without detectable regioisomeric byproducts. Second, the ^{1}H NMR spectra of the ladder polymers do not show additional signals attributable to structural defects or incomplete ladderization within the sensitivity of the measurements. Third, the FT-IR spectra confirm the disappearance of the internal alkyne stretching band (~2200 cm^{-1}), indicating efficient intramolecular cyclization.

Finally, the SEC traces remain essentially unchanged after ladderization, indicating that no detectable chain scission or intermolecular crosslinking occurs during the cyclization process.

Taken together, these results support near-complete ladderization and a high degree of structural uniformity in the bifacial ladder polymers within the sensitivity of the employed characterization methods. Although these data were already provided and discussed in the original manuscript, following the reviewer's suggestion we have revised the text to more clearly summarize these points and guide the reader to the relevant data.

[Page 9, lines 303–306] "Taken together, these spectroscopic and chromatographic analyses indicate that ladderization proceeds to near-completion along the polymer backbone, yielding structurally uniform ladder polymers with no detectable defects within the sensitivity of the employed characterization methods."

For Reviewer #2:

This manuscript reports the synthesis and spin selectivity of homochiral bifacial ladder polymers. The concept of exploiting the intrinsic rigidity of ladder polymers to construct bifacially chiral macromolecules is highly novel and has the potential to make a significant impact on fundamental macromolecular chemistry. The structural design is particularly intriguing, and the synthesis, characterization, and discussion are carried out in a meticulous and highly professional manner. The manuscript is clearly written and scientifically sound. In its current form, the work is suitable for publication.

That said, the reviewer would like to offer the following suggestions to further enhance the scientific merit and broader impact of the manuscript.

=> We would like to express our sincerest gratitude to this reviewer for this encouraging comment.

1. This is a purely conceptual suggestion. Could bifacial chirality be regarded as an analogue of tacticity in conventional polymers (*e.g.*, isotactic versus syndiotactic polypropylene)? If so, it may be valuable to discuss whether these concepts could be unified under a broader framework of "polymer tacticity," which may help situate bifacial chirality within established polymer science terminology.

=> We thank the reviewer for this thoughtful and constructive conceptual suggestion. We have carefully considered the relationship between bifacial chirality in the present ladder polymers and the concept of tacticity in polymer science.

Classical tacticity in vinyl polymers such as polypropylene is defined for backbones that do not possess true stereogenic centers; stereoregularity in such systems arises from the relative configuration of substituents along the chain. In contrast, the present bifacial ladder polymers contain intrinsically chiral backbone units with true stereogenic centers, and their facial

orientation is configurationally fixed by ladderization. In this strict sense, bifacial chirality is not equivalent to classical vinyl polymer tacticity.

We note that bifacial chirality bears a closer conceptual resemblance to the way tacticity is discussed for condensation polymers such as polylactide (PLA), where true stereogenic centers are present along the polymer backbone. However, an important distinction remains: in ring-opening polymerization of lactide, the boundaries of the original monomer units are lost along the polymer chain, whereas in the present system the monomer units remain structurally well-defined and their facial orientation is preserved along the backbone. Therefore, the correspondence is not exact.

Because of these differences, we refrain from developing a strict generalization of bifacial chirality within the conventional framework of polymer tacticity. Nevertheless, for intuitive comparison and clarity, we now include a brief discussion in the manuscript noting that LP-(*S*,*S*)-1 and LP-(*R*,*R*)-1 are conceptually analogous to optically active isotactic PLA derived from L- and D-lactide, respectively, while LP-*meso*-**1** is analogous to optically inactive atactic PLA derived from meso-lactide, and LP-*rac*-**1** is analogous to optically inactive atactic PLA obtained from a racemic mixture of L- and D-lactide. Representative references on tacticity and stereoregularity in polylactide systems have been added to support this comparison.

We have added explanatory sentences and a reference to the revised manuscript as follows:

[Page 7, lines 246–253] "For intuitive comparison, the stereochemical regularity of the polymers derived from these monomers can be discussed in relation to the concept of tacticity commonly used for polylactide (PLA) [42]. In this context, polymers derived from enantiopure (*S*,*S*)-**1** and (*R*,*R*)-**1** are conceptually analogous to optically active isotactic PLA derived from L- and D-lactide, respectively. Polymers derived from *meso*-**1** are analogous to optically inactive atactic PLA obtained from *meso*-lactide, whereas polymers derived from *rac*-**1** are analogous to optically inactive atactic PLA obtained from a racemic mixture of L- and D-lactide."

Ref #42) Roy S. S., Sarkar, S. & Chakraborty D. Determination of Polylactide Microstructure by Homonuclear Decoupled ^{1}H NMR Spectroscopy. *Chem. Rec.*, **21**,1968–1984 (2021).

2. Ladder polymers are well known for their exceptional thermal and chemical stability, as exemplified by classical systems such as BBL and more recent ladder-type polyanilines. Such robustness may translate into enhanced stability of chirality and chirality-related properties in the present system. It is therefore strongly suggested that the authors perform experiments, either in this work or in future studies, to demonstrate this stability. For example, does the chiral bifacial polymer retain its spin selectivity and circular dichroism signals after exposure to elevated temperatures? Is the chirality preserved up to the polymer's decomposition temperature? How does the material respond to harsh acidic or basic conditions, or to strong light irradiation? Demonstrating such robustness would substantially strengthen the case for ladder polymers as functional chiral materials with practical relevance.

=> We thank the reviewer for this important and forward-looking suggestion regarding the robustness of ladder polymers and the stability of chirality-related properties. We agree that the exceptional thermal and chemical stability of ladder polymers, as exemplified by classical systems such as BBL and ladder-type polyanilines, represents an important advantage for functional materials.

In the present work, thermogravimetric analysis (TGA) and differential scanning calorimetry (DSC) data for the bifacial ladder polymers are already provided in the Supplementary Information, demonstrating high thermal stability of the ladder polymer backbone. These results establish that the chemical integrity of the ladder polymers is maintained up to elevated temperatures.

At the same time, we recognize that the stability of higher-order chiral organization, which underlies the observed chiroptical and spin-selective responses, is a distinct and important question. To directly address this point, we have now performed additional thermal annealing experiments on thin films of LP-(*S*,*S*)-**1**. The films were annealed at temperatures ranging from 50 to 300 °C for 60 s in air, followed by measurement of the UV–vis/CD spectra. Remarkably, the CD spectral shape and intensity remain essentially unchanged even after annealing at 300 °C, indicating that the supramolecular chiral organization responsible for the chiroptical response is retained under these harsh thermal conditions. These results provide experimental evidence that the bifacial ladder polymer architecture confers substantial robustness not only to the covalent backbone but also to the higher-order chiral organization in the thin-film state. The new data (UV–vis/CD spectra after thermal annealing) and the corresponding discussion have been added to the revised manuscript as Extended Data Fig. 5d.

[Page 11, line 391–397] "To evaluate the thermal stability of the chiral supramolecular structure in the thin-film state, UV–vis/CD spectra of an LP-(*S*,*S*)-**1** thin film were measured after thermal annealing at 50–300 °C for 60 s (Extended Data Fig. 5d). Remarkably, the CD spectral shape and intensity remain essentially unchanged even after annealing at 300 °C. These results indicate that the supramolecular chirality responsible for the strong chiroptical response is highly stable against thermal treatment, consistent with the intrinsic structural stability of the ladder polymer backbone."

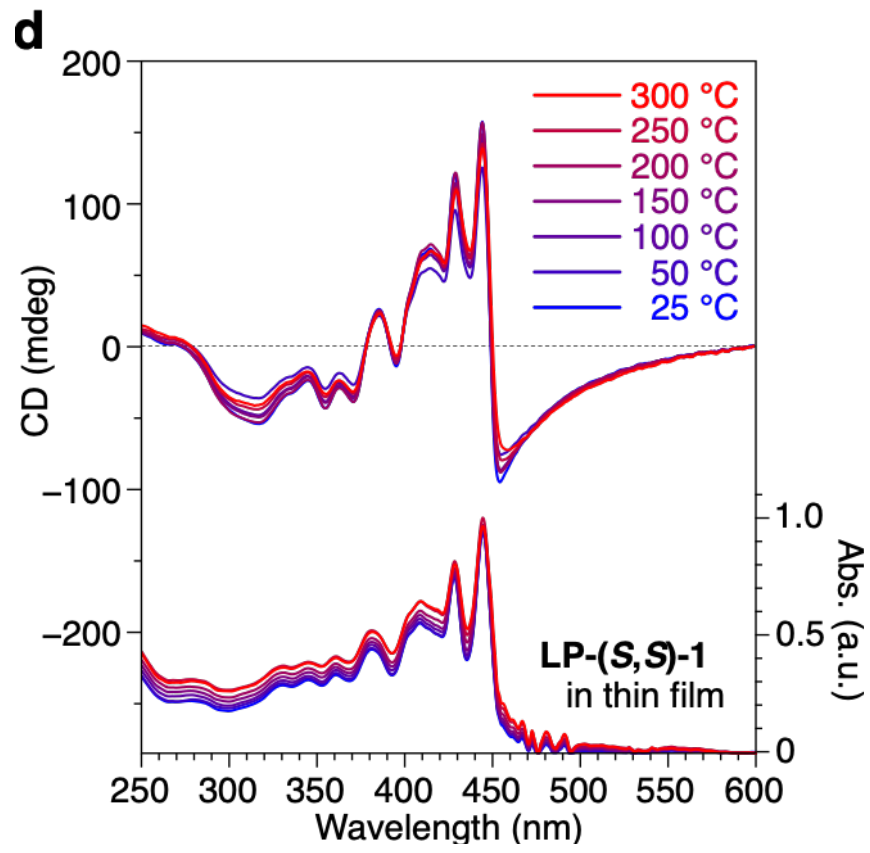

[Page 30] **Extended Data Fig. 5 | UV-vis/CD spectra in the thin-film state. d,** UV-vis/CD spectra of an LP-(*S*,*S*)-**1** thin film after thermal annealing at 50–300 °C for 60 s in air.

For Reviewer #3:

The paper by Ishiwari deals with the synthesis of bifacial ladder polymers, that is rigid double-stranded backbones bearing two deliberately differentiated molecular faces. The design takes advantage of a chirality-assisted synthetic strategy, an underdeveloped yet very important concept, which the authors masterfully develop for polymer synthesis and enantiospecific ladderization. The paper is very well written, and it is, in my view exciting chemistry. The cartoon representation of the chirality processes involved are particularly effective. The efficiency of the ladderization is properly demonstrated with the aid of model compounds. Structure-property-relationship in terms of spin-induced selectivity unravel the added significance of the ladder polymers. Although the authors have presented a similar synthetic approach in other contexts, the paper is very original and represents a huge step forward in the field of chirality assisted synthesis and ladder polymers. I recommend publication upon addressing the following minor points:

That said, the reviewer would like to offer the following suggestions to further enhance the scientific merit and broader impact of the manuscript.

=> We would like to express our sincerest gratitude to this reviewer for this encouraging comment.

a. the ladderization brings a systematic increase in the polydispersity index. I would have expected the opposite, with an increase in polymer rigidity. Can the authors comment on this point;

=> We thank the reviewer for this comment. The PDI values reported here are apparent values derived from SEC retention times calibrated with polystyrene standards. In SEC measurements, non-ideal interactions with the column as well as aggregation can alter retention behavior, leading to apparent changes in molecular weight and dispersity. In the present system, ladderization increases backbone planarity, which may enhance attractive interactions with the SEC column and affect retention times. In addition, increased planarity can promote aggregation, further influencing the apparent molecular weight and PDI. We therefore consider that these combined effects likely account for the observed changes in apparent PDI after ladderization. We have added an explanatory sentence to the caption of Extended Data Fig. 3 as follows;

[Page 29, caption of Extended Data Fig. 3] "The slightly higher apparent dispersity values of the ladder polymers might be attributed to non-ideal SEC behavior associated with aggregation and stronger interactions with the SEC column due to increased backbone planarity upon ladderization."

b. the authors should specify the scale for average batches of enantiomeric separation through chiral HPLC

=> We thank the reviewer for this helpful comment. The enantiomeric separation was typically performed using ca. 20 mg of racemate per batch on a semi-preparative Daicel CHIRALPAK IE

column (2 cm i.d., 25 cm length), with the sample dissolved in 4 mL of solvent. This information has been added to the Supporting Information.

[Supplementary Information, 1. Materials] "Enantiopure (*S*,*S*)-**1** and (*R*,*R*)-**1** were obtained by chiral-stationary-phase HPLC separation of *rac*-**1** using a Daicel CHIRALPAK IE semi-preparative column (2 cm i.d., 25 cm length), providing each enantiomer in >99% ee (Extended Data Fig. 1b). The enantiomeric separation was typically performed using ca. 20 mg of racemate per batch, with the sample dissolved in 4 mL of solvent."

c. please remove the following from the abstract "a new architectural motif in polymer science".

=> We thank the reviewer for this comment. We have revised the Abstract as follows; references to "a new architectural motif" have been removed at both occurrences.

Original (first occurrence):

"··· Here we introduce 'bifacial ladder polymers' —rigid double-stranded backbones bearing two deliberately differentiated molecular faces— as a new architectural motif in polymer science, realized through a chirality-assisted synthetic strategy."

Revised:

"··· Here we introduce 'bifacial ladder polymers' —rigid double-stranded backbones bearing two deliberately differentiated molecular faces— realized through a chirality-assisted synthetic strategy."

Original (second occurrence):

"These results demonstrate that bifacial ladder polymers constitute a new architectural motif, providing a versatile platform for emergent self-assemblies and functions in organic polymeric materials."

Revised:

"These results demonstrate that bifacial ladder polymers provide a versatile platform for emergent self-assemblies and functions in organic polymeric materials."

d. please cite relevant references: 10.1002/anie.202407495; 10.3390/sym16010116.

=> We thank the reviewer for this suggestion. The Angewandte Chemie article by Preda and Pasini (Ref. #20) was already cited in the original manuscript, as it represents an important contribution and is directly relevant to chirality-assisted synthesis and covalent ladder polymers.

The Symmetry article focuses on the synthesis of amino-functionalized chiral triptycenes and does not directly address helical ladder polymers or bifacial polymer design. We therefore did not include this reference in the present manuscript.

Point-by-Point Response to Reviewers' Comments in the 2nd Revision:

We sincerely thank the editor and all reviewers for their positive evaluation of our revised manuscript. As no further reviewer comments requiring additional revision were provided, our responses are given below. We have revised the manuscript, Supplementary Information and related files according to the editorial requests in the Author Checklist. In addition, we have prepared and uploaded a marked Review-Only Material file in which the revised parts made in response to the editorial requests in the Author Checklist are highlighted for ease of assessment.

For Reviewer #1:

The authors have addressed the reviewers' concerns, and the manuscript is ready for publication.

=> We sincerely thank the reviewer for the positive evaluation of our revised manuscript. We are pleased that the reviewer found that the concerns raised in the previous round were satisfactorily addressed and that the manuscript is now ready for publication.

For Reviewer #2:

I have examined the authors' responses to the previous round of reviewers' comments. They have addressed the comments in a satisfactory manner. I recommend the publication of this work in its current form.

=> We sincerely thank the reviewer for carefully examining our responses and revised manuscript. We are pleased that the reviewer found our revisions satisfactory and recommended publication of the manuscript in its current form.

For Reviewer #3:

In my opinion the authors have addressed in a satisfactory manner all comments by the reviewers. Publish as is. This is a truly fine paper.

=> We sincerely thank the reviewer for the very positive and encouraging evaluation of our revised manuscript. We were particularly encouraged by the reviewer's comment that this is "a truly fine paper." We are grateful that the reviewer found that all comments had been satisfactorily addressed and recommended publication of the manuscript.
